# Antennal transcriptome and differential expression of olfactory genes in the yellow peach moth, ***Conogethes** punctiferalis* (Lepidoptera: Crambidae)

**DOI:** 10.1038/srep29067

**Published:** 2016-07-01

**Authors:** Xiao-Jian Jia, Hai-Xiang Wang, Zeng-Guang Yan, Min-Zhao Zhang, Chun-Hua Wei, Xiao-Chun Qin, Wei-Rong Ji, Patrizia Falabella, Yan-Li Du

**Affiliations:** 1 Beijing Key Laboratory for Agricultural Application and New Technique, College of Plant Science and Technology, Beijing University of Agriculture, Beijing 102206, China; 2 College of Forestry, Shanxi Agricultural University, Taigu, Shanxi 030801, China; 3 State Key Laboratory of Environmental Criteria and Risk Assessment, Chinese Research Academy of Environmental Sciences, Beijing 100012, China; 4 Dipartimento di Scienze, Università della Basilicata, Campus Macchia Romana, Via dell’Ateneo Lucano, 10, Potenza 85100, Italy

## Abstract

The yellow peach moth (YPM), *Conogethes punctiferali*s (Guenée), is a multivoltine insect pest of crops and fruits. Antennal-expressed receptors are important for insects to detect olfactory cues for host finding, mate attraction and oviposition site selection. However, few olfactory related genes were reported in YPM until now. In the present study, we sequenced and characterized the antennal transcriptomes of male and female YPM. In total, 15 putative odorant binding proteins (OBPs), 46 putative odorant receptors (ORs) and 7 putative ionotropic receptors (IRs) were annotated and identified as olfactory-related genes of *C. punctiferalis*. Further analysis of RT-qPCR revealed that all these olfactory genes are primarily or uniquely expressed in male and female antennae. Among which, 3 OBPs (*OBP4*, *OBP8* and *PBP2*) and 4 ORs (*OR22*, *OR26*, *OR44* and *OR46*) were specially expressed in male antennae, whereas 4 ORs (*OR5*, *OR16*, *OR25* and *OR42*) were primarily expressed in female antennae. The predicted protein sequences were compared with homologs in other lepidopteran species and model insects, which showed high sequence homologies between *C. punctiferalis* and *O. furnacalis*. Our work allows for further functional studies of pheromone and general odorant detection genes, which might be meaningful targets for pest management.

The yellow peach moth *Conogethes punctiferalis* (Guenée) is a kind of multivoltine and polyphagous insect pest, distributed in the south eastern Asia and Australia^1^,^2^. The adult female feeds, oviposits and develops primarily in buds and fruits of peach, plum, chestnut, maize and sunflowers^2^. After hatching, larvae remain within the reproductive structures of the host plant and use them as food sources and a protected habitat to complete their life cycle. The endophytic behavior of larvae makes this insect difficult to control with conventional insecticides and other cultural practices. Thus, new methods to monitor *C. punctiferalis* population outbreaks and to achieve pest control have been initiated^3^,^4^,^5^. For example, sex pheromone composites of *C. punctiferalis* have been analyzed, synthetized and made into lure to attract male moths and disrupt their mating in fields^1^,^5^,^6^,^7^. At the same time, attention has been given to host plant volatiles usable to synergize response to sex attractant pheromone in the yellow peach moth^4^,^8^.

In insects, chemosensation serves to detect and react to environmental chemical cues, in virtually every aspect of their life cycle^9^,^10^. Olfaction, as a kind of chemosensation, is critical to food source identification, predator avoidance, oviposition site selection, kin recognition, mate choice, and toxic compound avoidance. It is, thus, an attractive target for pest control, for example, several olfactory-based strategies including mass trapping and mating disruption have been developed to control moth populations^11^. Better knowledge on the molecular mechanisms by which an odor generates a neuronal signal could lead to the identification of targets for the development of new control strategies.

Antennae are the primary olfactory sensor of insects and their cuticular surface is covered with several different types of small sensory structures, named sensilla, in which olfactory receptor neurons extend dendrites into the antennal lymph where peripheral olfactory signal transduction events occur. Previous studies reported diverse olfactory proteins, including odorant-binding proteins (OBPs), odorant receptors (ORs), chemosensory proteins (CSPs), sensory neuron membrane proteins (SNMPs), ionotropic receptors (IRs) and odorant degrading enzymes (ODEs) involved in different odor perception steps in signal transduction pathway^12^,^13^,^14^. OBPs are widely engaged in the initial biochemical recognition steps in insect odorant perception and play a key role in transporting hydrophobic odorants across the sensillum lymph to the ORs^15^,^16^. Recently, OBPs have attracted the attention of many researchers^17^,^18^,^19^. OBP family notably includes two sub-families: the pheromone-binding proteins (PBPs), transporting pheromone molecules, and the general odorant-binding proteins (GOBPs), transporting general odorants such as plant volatiles^20^,^21^. As to the procedures of olfaction transmission, the volatile hydrophobic molecules are firstly bound by the sensilla-enriched binding proteins (OBPs and CSPs) to cross the aqueous sensillum lymph that embeds the olfactory neuron dendrites, thus interacting with the membrane-bound chemosensory receptors (ORs and IRs) located in the dendritic membrane of receptor neurons^22^. The chemical signal is then transformed into an electric signal that is transmitted to the brain. Sensory neuron membrane proteins (SNMPs), located in the dendritic membrane of pheromone sensitive neurons, are thought to trigger ligand delivery to the receptor. Subsequently, signal termination may then be ensured by ODEs^14^,^21^,^23^,^24^,^25^.

Lepidopteran species have been widely used as models of insect olfaction because of their highly specific and sensitive olfactory senses and complex olfactory behaviors. The emergence of next-generation sequencing (high-throughput deep sequencing) technology has dramatically improved the efficiency and quantity of gene annotation^26^. Similarly, application of the high-throughput sequencing technology in the field of entomological research has greatly promoted its progress^27^,^28^,^29^. Recently, studies on antennal transcriptomes have led to the identification of olfactory-related genes in several moth species^18^,^19^,^28^,^30^,^31^,^32^, which demonstrated the power of transgenomic strategies for olfactory gene identification. However, in *C. punctiferalis*, only two olfactory-related genes (*CpunOrco* and *CpunPBP*1) with their expression profiling were reported to date^33^,^34^. Hence, little is known about the function of olfactory genes of *C. punctiferalis*, due to the deficiency of the genomic data for this species.

In this study, we used next generation sequencing (NGS) to gain insights into the complexity of the antennal transcriptome and to identify genes related to chemosensation of *C. punctiferalis*. We also report the results from gene ontology (GO) annotation as well as sets of putative OBPs, ORs and IRs in *C. punctiferalis*. Moreover, using real-time quantitative-PCR (RT-qPCR), we screened all the annotated olfactory genes from *C. punctiferalis* antennal transcriptomes. The results will be the basis for further studies of the olfactory mechanisms of *C. punctiferalis* and to select some of the olfactory genes that may be used as targets in management programs of this destructive insect pest.

## Results and Discussion

### Sequence analysis and assembly

Two non-normalized cDNA libraries (SRR2976624 and SRR2976631) of the male and female *C. punctiferalis* antennae were constructed. After a trimming of adaptor sequences, contaminating or low quality sequences, 70.3 and 74.2 million clean-reads comprised of 8.88 and 9.34 gigabases were generated from male and female antennae respectively, and remained for the following assembly.

**Figure 1:**
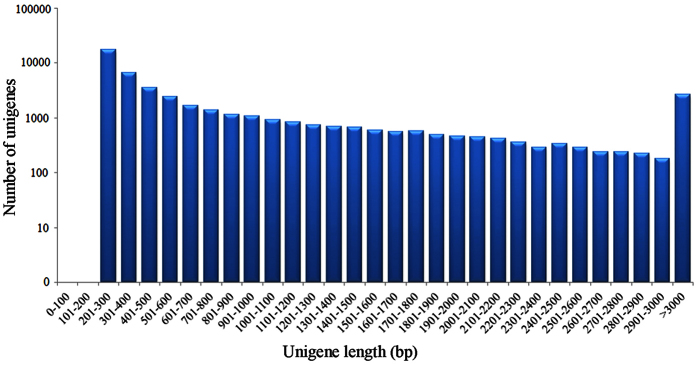
The size distribution of the assembled unigenes from *Conogethes punctiferalis* male and female antennal transcriptome. A total of 47,109 unigenes were generated. Among which, 19,765 (41.96%) were longer than 500 bp and 12,129 (25.75%) were longer than 1 kb. The x-axis represents the unigene length (bp), and the y-axis represents the number of unigenes.

All clean reads from male and female antennae were assembled and a total of 47,109 unigenes were generated. The transcript dataset was 41.82 mega bases in size and with a mean length of 887.83 bp and N50 of 1,808 bp. Among these unigenes, 19,765 (41.96%) were longer than 500 bp and 12,129 (25.75%) were longer than 1 kb (Fig. 1 and Table 1). Compared with the published Lepidoptera antennal transcriptomes, especially the two Crambidae species *Chilo suppressalis* (66,560 unigenes, mean length 761bp, N50 1,271bp)^35^ and *Ostrinia furnacalis* (37,687 unigenes, mean length 818bp, N50 1,022bp)^18^, the assembly quality of our transcriptome was qualified and even better than most of these transcriptomes. These results further demonstrated the effectiveness of Illumina sequencing technology in rapidly capturing a large portion of the transcriptome, and provided a sequence basis for future studies, such as rapid characterization of a large portion of the transcriptome and better reference of the genes of interest^36^. The assembled sequences have been deposited in the NCBI Transcriptome Shotgun Assembly (TSA) Database with the title as BioProject: PRJNA304355 and accession numbers GEDO01000001 to GEDO01000068.

### Functional annotation of the *C. punctiferalis* antennal unigenes

The unigenes were annotated by aligning with the deposited ones in diverse protein databases including the National Center for Biotechnology Information (NCBI) non-redundant protein (nr) database, the Kyoto Encyclopedia of Genes and Genomes (KEGG), the UniProt/Swiss-Prot, Gene Ontology (GO), Cluster of Orthologous Groups of proteins (COG) and the UniProt/TrEMBL databases, using BLASTx with a cut off *E*-value of 10^−5^ (Table 2). The analyses showed that a total of 18990 unigenes (40.31%) were successfully annotated in all above-mentioned databases. Of which, 18924 unigenes (40.17%) had significant matches in the nr database, followed by 11489 unigenes (24.39%) in the Swiss-Prot database. However, 28119 unigenes (59.69%) were unmapped in these databases. The higher percentage of sequences without annotation information could be attributable to the insufficient sequences in public databases for phylogenetically closely related species to date^37^. For example, in the two published Crambidae antennal transcriptomes, the ratio of the unigenes annotated in nr database in *C. suppressalis*^35^ and *O. furnacalis*^18^ was 45.4% and 41.2% respectively, similar to the results in present study. On the other hand, short reads obtained from sequencing would rarely be matched to known species because the significance of the BLAST comparison depends in part on the length of the query sequence^37^. In the present study, more than one third (36.65%) unigenes were shorter than 300 bp, which might be too short to allow for statistically meaningfully matches. As to sequences longer than 1 kb, the annotation rate was 76.08%, whereas for sequences longer than 300 bp, the percentage decreased to 52.95% (Tables 1 and 2). In addition, the low annotated percentage might be due to non-conserved areas of proteins where homology is not detected^38^,^39^. For example, the 5′ ends of genes generally show less sequence conservation than the body^40^. Therefore, partial transcripts, especially unigenes representing the 5′ CDS, may not find matches in the various databases.

**Figure 2:**
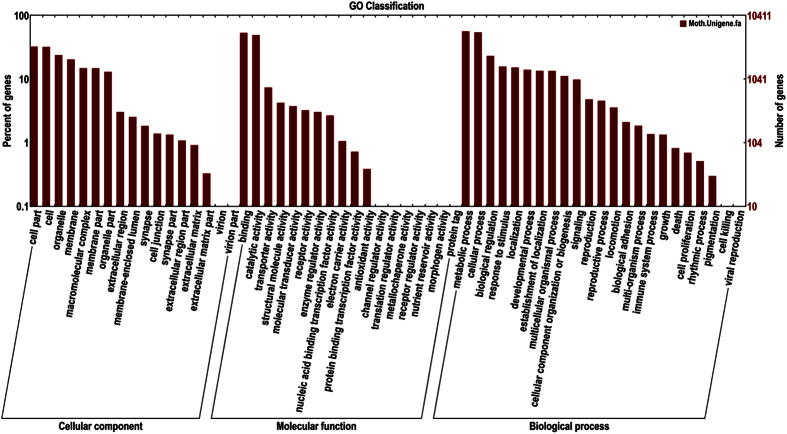
Functional annotation of assembled sequences based on gene ontology (GO) categorization. GO analysis was performed at the level two for three main categories (cellular component, molecular function, and biological process).

For GO analysis, a total of 10411 unigenes (22.10%) could be assigned to three ontologies, including biological process ontology, cellular components ontology and molecular function (Fig. 2). In biological process ontology, the “metabolic process” and “cellular process” were most represented, with 5853 (22.64%) and 5651 (21.85%) unigenes, respectively. In the cellular component ontology, the terms were mainly distributed in cell (3337 unigenes, 19.59%) and cell part (3364 unigenes, 19.74%). In the molecular function ontology, the terms binding functions (5546 unigenes, 41.27%) and catalytic activity (5120 unigenes, 38.10%) were the most represented. These results were also similar to those found in the antennal transcriptomes of *Manduca sexta*^30^, *Spodoptera littoralis*^41^, and *Agrotis ipsilon*^42^.

**Figure 3:**
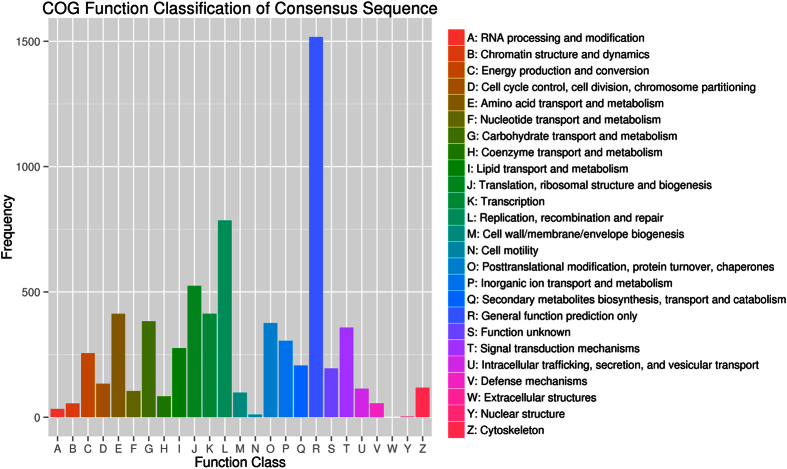
Cluster of orthologous groups (COG) classification. In total, 5076 of the 47109 unigenes with non-redundant database hits were grouped into 25 COG classifications.

In addition, all unigenes were subjected to a search against the COG database for functional prediction and classification (Fig. 3). As result, a total of 5076 unigenes with hits in the nr database could be assigned to COG classification and divided into 25 specific categories. The category of “general function prediction”, similarly to that found in *Dialeurodes citri*^36^, was also the largest group (1517 unigenes, 29.89%), followed by the classification of “replication, recombination and repair” (785 unigenes, 15.46%). The categories of “cell motility” (11 unigenes, 0.22%) and “nuclear structure” (3 unigenes, 0.06%) were the smallest groups.

The unigenes metabolic pathway analysis was also conducted using the KEGG annotation system. This process predicted a total of 197 pathways, which represented a total of 4931 unigenes.

### Identification of olfactory genes and analysis of differentially expressed genes

A total of 68 olfactory genes, including 15 OBPs, 46 ORs and 7 IRs, were identified from antennal transcriptome of *C. punctiferalis*. Analysis of gene expression differences at a single time indicated that the antennal transcriptomes of male and female *C. punctiferalis* were different, mainly distributed in the expression of 1308 genes. Using female antennae as the reference standard, we found 759 up-regulated genes and 549 down-regulated genes. Among which, 3 OBPs (OBP4, OBP8, PBP2) and 4 ORs (OR22, OR26, OR44, OR46) are male antennae-specific expression, whereas 4 ORs (OR5, OR16, OR25, OR42) are female antennae-enriched expression.

### Candidate odorant binding proteins in the *C. punctiferalis* antennae

In the antennal transcriptome of *C. punctiferalis*, a total of 15 OBP genes, including four pheromone binding proteins (PBPs), two general odorant binding proteins (GOBPs) and one antennal binding protein (ABP) were identified (Table 3). The BLASTx results indicated that all of these 15 identified CpunOBPs shared a typical structural feature of OBPs (i.e. having typical six conserved cysteins) with other insects^43^ and twelve of them shared relatively high amino acid identities (62–91%) with Lepidoptera OBPs at NCBI. Thirteen of these presented intact ORFs with lengths ranging from 384 bp to 837 bp, and the other two genes, *CpunPBP1* and *CpunABP*, were represented as partial ORFs with length 483 bp and 432 bp, respectively.

Among the 15 putative OBP genes in the *C. punctiferalis* antennal transcriptome data, the gene of *CpunPBP*1 has been reported in our previous study^34^, but the remaining 14 *CpunOBP*s are reported here for the first time. The number of *C. punctiferalis* OBPs was less than those identified from the antennal transcriptome of *Bombyx mori* (44)^17^, *Helicoverpa armigera* (26)^44^, *Dendrolimus houi* (23)^45^, *O. furnacalis* (23)^18^ and *Spodoptera litura* (38)^19^, but comparable with those identified in *M. sexta* (18)^30^ and more than those identified in *Spodoptera exigua* (11)^46^. Since we used the same methods and technologies reported for previously cited papers we hypothesized the possible reasons of the small number of OBPs identified in *C. punctiferalis* in actually less number of OBPs than other caterpillar or that some OBPs may be larvae-biased ones, some species-specific ones and some ones that low expressed in antennae. For example, some of the genes might be expressed only in the larva^47^,^48^.

The RPKM value analysis revealed that 12 OBP genes (OBP2, OBP5, OBP6, OBP7, OBP8, PBP1, PBP2, PBP3, PBP4, GOBP1, GOBP2 and ABP) were highly expressed in both male and female antennal transcriptomes (RPKM value much higher than 100). The other 3 OBP genes (OBP1, OBP3 and OBP4), however, showed a relative low expression level (RPKM ranged from 0 to 8). Six OBPs (OBP4, OBP7, OBP8, PBP2, PBP3 and PBP4) showed a higher RPKM in the male antennae than in the female antennae (about 1 to 20 times) (Table 3).

**Figure 4:**
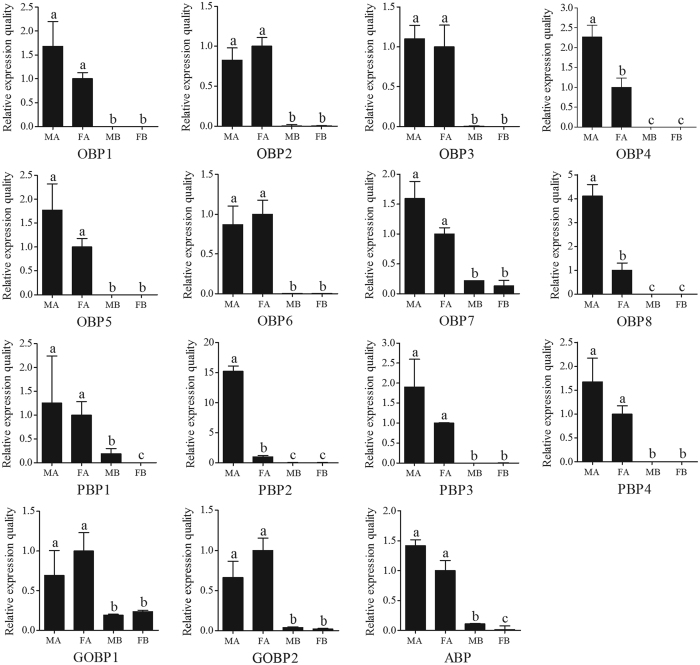
*Conogethes punctiferalis* OBP transcript levels in different tissues as measured by RT-qPCR. MA: male antennae; FA: female antennae; MB: male body with antennae cut off; FB: female body with antennae cut off. The internal controls *β-actin* was used to normalize transcript levels in each sample. The standard error is represented by the error bar, and the different letters (**a–c**) above each bar denote significant differences (*p* < 0.05).

Furthermore, RT-qPCR analysis was performed to compare the accurate quantitative expression levels of these OBP genes among different tissues between sexes (Fig. 4). The results indicated that three OBPs (*OBP4, OBP8 and PBP2*) were significantly overexpressed in male antennae and have male antennae-specific expression, which suggests that these OBPs may play essential roles in the detection of sex pheromones. Comparatively, the expression of 2 GOBPs (*GOBP1*, *GOBP2*) in female antennae were almost twice to three times higher than those in male antennae) (Table 3, Fig. 4), which suggests that these OBPs may play important roles in the detection of general odorants such as host plant volatiles. Especially, three OBPs (*OBP5*, *PBP1* and *ABP*) showed somewhat higher RPKM in the female antennae than in the male antennae (Table 3), lack concordance with the results of RT-qPCR (Fig. 4), which maybe the sequencing depth of Hiseq2500 is not good enough, or may need more repetition to further test in the future study.

In addition, the RT-qPCR results showed that all of the 15 *C. punctiferalis OBPs* were significantly overexpressed in the antennae compared with the bodies (*P* < 0.05) (Fig. 4). The result of high expression in antennae was not only concordant with that from RPKM values in present study, but also same as that in *Anopheles gambiae*^10^, *H. armigera*^44^, *Ips typographus* and *Dendroctonus ponderosae*^49^, *Ag. Ipsilon*^42^ and *Sp. Litura*^19^. For the body parts with antennae cut off, no significant difference appeared between male and female OBP gene expression levels, excepting OBP7 and PBP1 significantly overexpressed in the male body, whereas ABP overexpressed in female body. Up regulation in antennae indicate their participation in moth olfaction during attraction to the host plants and may offer targets for disrupting this activity.

**Figure 5:**
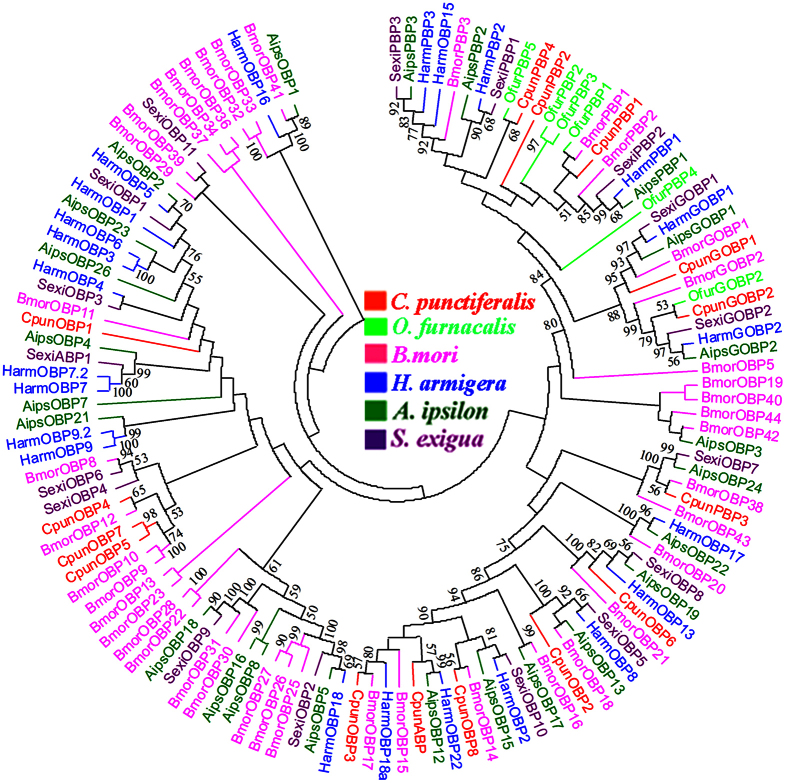
Neighbor-joining dendrogram based on protein sequences of candidate odorant binding proteins (OBPs). The protein names and sequences of OBPs used in this analysis are listed in Supplementary Table 3.

A neighbor-joining tree of 126 OBP sequences was built from six different Lepidoptera species, including *C. punctiferlis*, *O. furnacalis*, *B. mori*, *H. armigera*, *Ag. ipsilon* and *Sp. exigua* (Fig. 5). The OBP trees indicated that the six Lepidoptera species were extremely divergent; however, the GOBPs (*GOBP1* and *GOBP2*) were highly conserved among different species. All PBPs, GOBPs, and OBPs from *C. punctiferlis* were grouped into corresponding branches except *CpunPBP3* clustered with OBP group. No evident specific expansion of OBP lineages was found except *CpunOBP5* and *CpunOBP7* were grouped together.

### Candidate olfactory receptors in the *C. punctiferalis* antennae

In the process of recognizing smells, insect ORs are the most important players in sex pheromone and general odorant detection. In this research, the OR candidates from the *C. punctiferalis* antennal transcriptomes were identified carefully, and a total of 46 ORs (including the full-length or almost full-length OR candidates) were submitted for further analysis. Of which, ten ORs (*OR2*, *10*, *17*, *19*, *21*, *22*, *23*, *25*, *30* and *32*) had intact ORF, whereas the other 36 ORs were represented as partial open reading frames. In addition, 45 of these submitted 46 ORs were first report in *C. punctiferalis* and identified as typical ORs, whereas one OR (*OR23*) has been reported and was identified as atypical coreceptor^33^ (Table 4). The number of *C. punctiferalis* ORs identified in this study was comparable with the numbers identified in *M. sexta* (47)^30^, *H. armigera* (47)^44^ and *Ag. ipsilon* (42)^42^, and more than those identified in *Sesamia inferens* (39)^32^, *Dendrolimus houi* (33) and *Dendrolimus kikuchii* (33)^45^, but less than those identified in *B. mori* (72)^17^ and *O. furnacalis* (56)^18^. Considering that those OR candidates with partial ORFs were discarded in the present study, we speculated that more ORs may be identified in the future.

**Figure 6:**
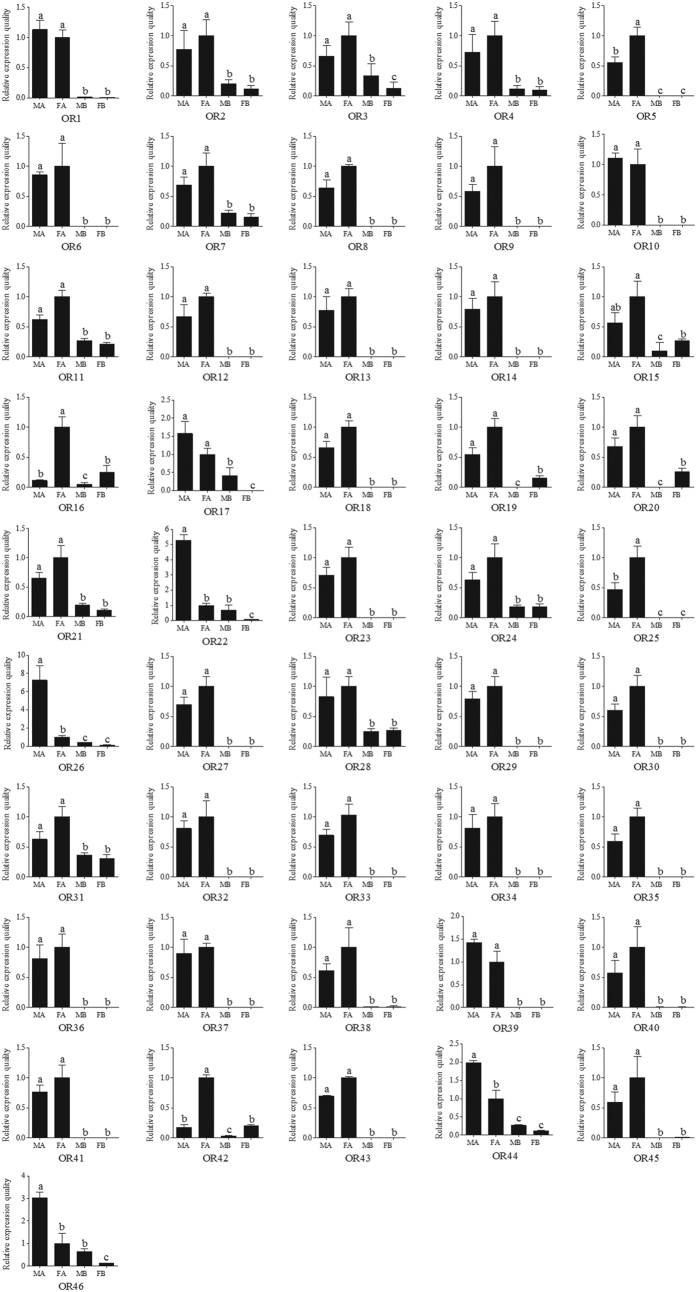
*Conogethes punctiferalis* OR transcript levels in different tissues as measured by RT-qPCR. MA: male antennae; FA: female antennae; MB: male body with antennae cut off; FB: female body with antennae cut off. The internal controls *β-actin* was used to normalize transcript levels in each sample. The standard error is represented by the error bar, and the different letters (**a–c**) above each bar denote significant differences (*p* < 0.05).

The RPKM value analysis revealed that the *ORco* (*OR23*) had the highest expression level among the 46 ORs, with RPKM value of 320 and 531 in the male and female antennae, respectively. The other 45 typical ORs, however, showed a relative low expression level (RPKM ranged from 0 to 233) compared with the *ORco* (*OR23*) and OBP genes. In detail, five ORs (*OR17*, *OR22*, *OR26*, *OR44* and *OR46*) showed a higher RPKM in the male antennae than in the female antennae (more than 10 times), whereas *OR16* and *OR42* showed opposite results, with RPKM from the male antennae almost 20 times lower compared to female antennae (Table 4). The RT-qPCR results indicated that *ORco* (*OR23*) had a significant higher expression level in the antennae than in the bodies of *C. punctiferalis*, which was concordant with previous results^33^. Moreover, 4 ORs (*OR22*, *OR26*, *OR44* and *OR46*) have a male antennae-specific expression, whereas other 4 ORs (*OR5*, *OR16*, *OR25* and *OR42*) have a female antennae-enriched expression (Fig. 6). This male-biased transcription also appears to be retained among the *B. mori* orthologs *OR3*, *4*, *5* and *6*^50^. Comparative genomic analyses suggested that male-biased expression and female pheromone receptor function is retained in OR subfamily in *B. mori*, and female-biased transcription of OR gene family members is predicted among transcripts in both *B. mori*^50^,^51^ and *O. furnacalis*^18^.

**Figure 7:**
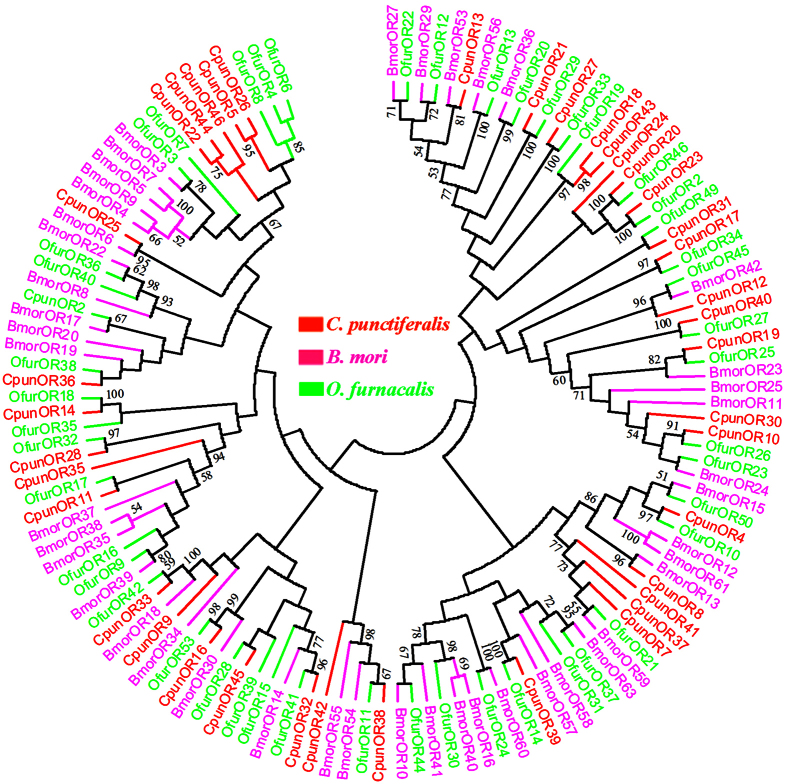
Neighbor-joining dendrogram based on protein sequences of candidate odorant receptor proteins (ORs). The protein names and sequences of ORs used in this analysis are listed in Supplementary Table 4.

A neighbor-joining tree of 130 OR sequences was built from three different Lepidoptera species, including *C. punctiferlis*, *B. mori* and *O. furnacalis* (Fig. 7). The *ORco* (*OR23*) was clustered with other Lepidoptera *ORco* sequences (*OfurOR2*). Most ORs from *C. punctiferlis* and *O. furnacalis* appear in pairs on the dendrogram, according with the fact that they belong to the same family of Crambidae. Especially to be mentioned, four male-biased ORs (*OR22*, *OR26*, *OR44* and *OR46*) were clustered together with *OfurOR4* and *OfurOR6*, which were suggestive of a functional role in male pheromone response^18^. However, the female-biased ORs (*OR5*, *OR16*, *OR25* and *OR42*) were stretched in different branches. Given that several *B. mori* female-biased ORs are capable to respond to host plant volatiles^51^,^52^, it is conceivable that *C. punctiferalis* orthologs may have retained similar functions, but further studies are required to investigate any potential evolutionary conservation of function. However, based on the different expression profiles of these ORs in male and female antennae, we suggest that these male antennae-enriched expressed ORs are involved in sex pheromone detection, whereas female antennae-enriched expressed ORs play important roles in locating suitable host plants and oviposition sites.

### Candidate ionotropic receptors in the *C. punctiferalis* antennae

IRs were recently discovered as another class of receptors involved in chemoreception^53^. Since IRs have been identified throughout protostome lineages, they belong to an ancient chemosensory receptor family^54^. To date, 15 IRs in *Cy. Pomonella*^28^, 24 IRs in *Ag. Ipsilon*^42^, and 12 IRs in *H. armigera*^44^ have been identified. In the present study, 7 IR genes were first identified from the *C. punctiferalis* antennal transcriptomes. Among these, two IRs (IR2 and IR6) had intact ORF, whereas the other 5 candidate IRs were represented as partial ORFs. The BLASTx results indicated that all of these 7 identified CpunIRs shared relatively high amino acid identities (67–81%) with Lepidoptera IRs at NCBI (Table 5). Compared with the number of IRs in above mentioned three species, the scarcity of divergent IRs in *C. punctiferalis* antennal transcriptomes may due to some IRs only expressed in other tissues. For example, the expression of divergent IRs was detected only in gustatory organs in *Drosophila melanogaster*^53^,^54^. It is generally reported that in insects, the antennal IR subfamily constitutes only a portion of the total number of IRs^49^. In particular 15 *D. melanogaster* IRs^53^, 10 *H. armigera* IRs^44^ and 7 *S. littoralis* IRs^55^ were expressed exclusively in the antennae.

**Figure 8:**
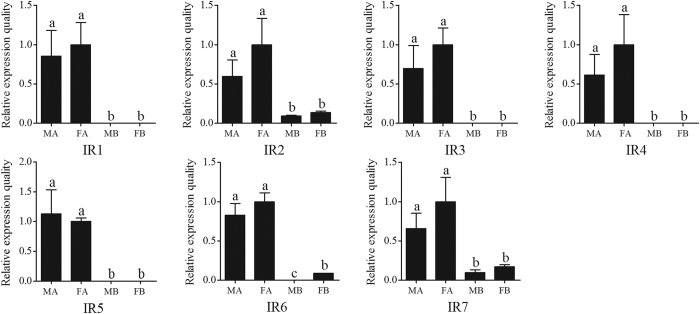
*Conogethes punctiferalis* IR transcript levels in different tissues as measured by qRT-PCR. MA: male antennae; FA: female antennae; MB: male body with antennae cut off; FB: female body with antennae cut off. The internal controls *β-actin* was used to normalize transcript levels in each sample. The standard error is represented by the error bar, and the different letters (**a–c**) above each bar denote significant differences (*p* < 0.05).

**Figure 9:**
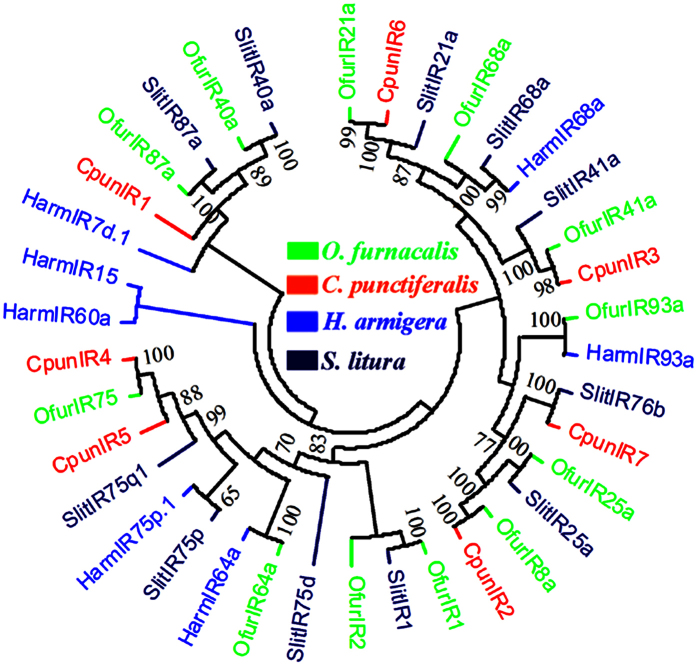
Neighbor-joining dendrogram based on protein sequences of candidate ionotropic receptors (IRs). The protein names and sequences of IRs used in this analysis are listed in Supplementary Table 5.

The RPKM value analysis revealed almost no differences between male and female IRs, which was validated by RT-qPCR results (Table 5, Fig. 8). Therefore we speculated that the IRs were relatively highly conserved. Similarly to the ORs, the RPKM value analysis revealed that all of the 7 IRs showed a relative low expression level (RPKM value ranged from 1 to 54) compared with the OBPs. Our RT-qPCR results also indicated that all of the 7 *C. punctiferlis* IRs were highly expressed in the antennae. The antennae-enriched IRs may play important roles in odorant detection. The IR tree from four lepidopteran insects was similar to that from ORs, with most of IRs from *C. punctiferlis* and *O. furnacalis* appearing in pairs on the dendrogram, concordant with the fact that they belong to the same family of Crambidae (Fig. 9).

## Conclusion

Olfaction is a primary sensory modality in insects. In the present study we performed a comprehensive analysis of the antennal transcriptome of *C. punctiferalis*. As a result, three major gene families (OBPs, ORs and IRs) that encode olfactory-related proteins were annotated for the first time, and their expression levels were measured based on the transcriptomic data, and validated by RT-qPCR. The expression profile analysis revealed that 15 OBPs, 46 ORs and 7 IRs are uniquely or primarily expressed in the male and female antennae. The results from the present study will be fundamental for future functional studies of olfactory-related genes in *C. punctiferalis*. Connection of the molecular information presented here and the available chemical and ecological knowledge will clarify the olfactory mechanisms of *C. punctiferalis*, and provide new targets for pest management in the future.

## Materials and Methods

### Insect rearing and tissue collection

The mature larvae of *C. punctiferalis* were collected from cornfields of the Agricultural Experiment Station of Beijing University of Agriculture on October 9^th^, 2009, and the insects had been maintained for about 25 generations on maize in climate incubators (RTOP-B, Zhejiang Top Instrument Co., Ltd.) at 23 ± 1 °C, RH 75 ± 2%, 16L/8D photoperiod, and 3500 lux light intensity. Adult moths were provided with 5–8% honey solution after emergence^2^. Antennae were excised from 3-days-old male and female moths, frozen immediately and stored in liquid nitrogen until use.

### RNA extraction

200 antennae from each sex were pooled for total RNA extraction using RNeasy Plus Mini Kit (Qiagen GmbH, Hiden, Germany) following the manufacturer’s instructions. During which, the DNA could be eliminated automatically. The quantity and concentration of RNA samples were determined using 1.2% agarose electrophoresis and a Qubit^®^ RNA Assay Kit in a Qubit^®^ 2.0 Fluorometer (Life Technologies, CA, USA), respectively. The integrity of RNA samples was assessed using a RNA Nano 6000 Assay Kit of the Bioanalyzer 2100 system (Agilent Technologies, CA, USA).

### cDNA library construction and sequencing

Firstly, mRNA was purified from total RNA using Oligo (dT) magnetic beads. mRNA was fragmented in fragmentation buffer into 200–700 nucleotides sections. The first cDNA was synthesized using random hexamer primer with the fragmented mRNA as templates. Second–strand cDNA were synthesized using DNA Polymerase I, dNTPs and RNaseH (Invitrogen, Carlsbad, CA, USA). Short fragments were purified using QiaQuik PCR Extraction Kit (Qiagen, Hilden, Germany) and eluted with ethidium bromide (EB) buffer for end-repair, poly (A) addition, then linked to sequencing adapters. The suitable fragments, as judged by agarose gel electrophoresis, were selected as templates for PCR amplification. The cDNA library of *C. punctiferalis* was sequenced on Illumina HiSeq™ 2500 using PE125 technology in a single run by Beijing Biomake Company.

### Sequence analysis and assembly

The raw reads were cleaned by removing adapter sequences, low-quality sequences (reads with ambiguous bases “N”), and reads with >10% Q < 20 bases. Cleaned reads shorter than 60 bases were removed because short reads might represent sequencing artifacts^56^. The quality reads were assembled into unigenes using short reads assembling program Trinity (Trinityrnaseq_r2013-11-10)^57^.

### Functional annotation

The assembled sequences were annotated using BLASTn (version 2.2.14) with an *E*-value < 10^−5^ and BLASTx (*E*-value < 10^−5^) programs against the NCBI nr database^58^,^59^. To annotate the assembled sequences with GO terms, the Swiss-Prot BLAST results were imported into BLAST2GO, a software package that retrieves GO terms, allowing gene functions to be determined and compared^60^. The COG database was also used to predict and classify functions of the unigene sequences^61^. Kyoto Encyclopedia of Genes and Genome (KEGG) pathways were assigned to the assembled sequences using the online KEGG Automatic Annotation Server (KAAS) used to determine pathway annotations for unigenes^62^. Finally, the best matches were used to identify coding regions and to determine the sequence direction.

### Olfactory genes identification and phylogenetic analyses

All candidate OBPs, ORs and IRs were manually checked by the BLASTx program at the National Center for Biotechnology Information (NCBI). For contigs with hits against genes of interest, open reading frames (ORFs) were identified and the annotation verified OBPs, ORs and IRs protein sequences and orthologs in other species of Lepidoptera and model insects to analyze the characteristics of olfactory genes in *C. punctiferalis*. The nucleotide sequences of all olfactory genes that were identified from *C. punctiferalis* antennal transcriptomes were named according to sequence homology analysis and numbered arbitrarily. Of which, the genes of *OBP1*, *OBP2*, *PBP1*, *PBP4*, *GOBP1*, *GOBP2*, and *ABP* were numbered according to blast results, whereas other OBPs, and all ORs and IRs were numbered arbitrarily. In addition, we use the prefix *CpunOBP*, *CpunOR* or *CpunIR* to reflect that the gene is a putative member belonging to yellow peach moth OBP, OR or IR-like family (Tables 3, 4 and 5).

Phylogenetic reconstruction for analysis of OBPs, ORs and IRs was performed with MEGA5.0 software^63^, with construct consensus phylogenetic trees using neighbour-joining (NJ) method. Bootstrap analysis of 1000 replications was performed to evaluate the branch strength of each tree.

### Analysis of differentially expressed genes

To compare the differential expression of chemosensory genes in the *C. punctiferalis* male and female antennal transcriptomes, the read number for each chemosensory gene between male and female antennae was converted to RPKM (Reads Per Kilobase of exon model per Million mapped reads)^64^. The RPKM method eliminates the influence of gene length and sequencing depth on the calculation of gene expression, and is currently the most commonly used method for estimating gene expression levels. Thus, the calculated gene expression can be directly used to compare gene expression between samples.

### RT-qPCR and data analysis

To verify the quantification of gene expression levels in transcriptome sequencing, the RT-qPCR for different tissue and sex samples was performed. Two biological samples each with 80 male antennae or 80 female antennae, and another two samples each with one male or one female moth body with antennae cut off, were used for RNA extraction using RNeasy Plus Mini Kit (Qiagen GmbH, Hiden, Germany) following the manufacturer’s instructions. cDNAs from antennae and other body part of both sexes were synthesized using the SMART^TM^PCR cDNA synthesis kit(Clontech, Mountain View, CA, USA).

An equal amount of cDNA (100 ng) was used as RT-qPCR templates. For each sample, the *β-actin* gene (GenBank JX119014) of *C. punctiferalis* was used as an internal control gene. The primers were designed using the Primer Premier 5.0 program (Primer Biosoft International, Palo Alto, CA, USA) (Supplementary Table 2). The RT-qPCR was performed in an iCycler iQ2 Real-Time PCR Detection System (Bio-Rad, Hercules, CA, USA) with SYBR green dye bound to double strand DNA at the end of each elongation cycle. Each RT-qPCR reaction was conducted in a 20.0 μl reaction mixture containing 10.0 μl of 2 × SYBR Green PCR Master Mix, 0.4 μl of each primer, 2.0 μl of cDNA sample (100 ng/μl), and 7.2 μl sterilized ultrapure H_2_O. The cycling parameters were: 95 °C for 3 min, 40 cycles at 95 °C for 10 sec, and 60 °C for 30 sec to measure the dissociation curves. Blank controls with sterilized ultrapure H_2_O instead of template were included in each experiment. To check reproducibility, each RT-qPCR reaction for each sample was carried out in three technical replicates and three biological replicates.

The Relative quantification analyses among four samples were performed using comparative 2^−ΔΔCt^ method^65^. The comparative analyses of each target gene among different tissues were determined with one-way nested analysis of variance (ANOVA), followed by Least-significant difference (LSD) test using SPSS Statistics 19.0 (SPSS Inc., Chicago, IL, USA).

## Additional Information

**How to cite this article**: Jia, X.-J. *et al*. Antennal transcriptome and differential expression of olfactory genes in the yellow peach moth, *Conogethes punctiferalis* (Lepidoptera: Crambidae). *Sci. Rep.* **6**, 29067; doi: 10.1038/srep29067 (2016).

## Supplementary Material

Supplementary Information

## Figures and Tables

**Table 1:** An overview of the sequencing and assembly process.

Length (bp)	Contig	Transcript	Unigene
201–300	10,879,481 (99.69%)	24,147 (23.82%)	17,266 (36.65%)
301–500	12,435 (0.11%)	18,792 (18.54%)	10,078 (21.39%)
501–1000	9,011 (0.08%)	20.661 (20.38%)	7,636 (16.21%)
1001–2000	6,688 (0.06%)	20.364 (20.09%)	6,467 (13.73%)
2000+	5,710 (0.05%)	17,402 (17.17%)	5,662 (12.02%)
Total Number	10,913,325	101,366	47,109
Total Length	467,980,625	116,001,455	41,824,959
N50 Length	45	2,031	1,808
Mean Length	42.88	1144.38	887.83

**Table 2:** Functional annotation of the *Conogethes punctiferalis.*

Annotated databases	unigene	≥300 bp	≥1000 bp
COG_annotation	5,076	4,676	3,444
GO_annotation	10,411	8,900	5,826
KEGG_annotation	4,931	4,495	3,119
SwissProt_annotation	11,489	10,399	7,245
nr_annotation	18,924	15,781	9,224
Total	18,990	15,803	9,228

COG = Cluster of Orthologous Groups of proteins; GO = Gene Ontology; KEGG = Kyoto Encyclopedia of Genes and Genomes; nr = non-redundant protein.

**Table 3:** Candidate OBP genes in *Conogethes punctiferalis* antennae.

Unigene reference	Gene name	ORF (bp)	Accession number	BLASTx annotation	Score	*E*-value	Identify	RPKM Value	
								male	female
Unigene_32154	OBP1	456	GEDO01000008.1	gb|AGM38610.1|odorant binding protein [Chilo suppressalis]	156	5e–45	51%	2.78	3.96
Unigene_24192	OBP2	417	GEDO01000009.1	gb|AFG73000.1| odorant-binding protein 2 [Cnaphalocrocis medinalis]	251	3e–84	84%	497.19	506.90
Unigene_26427	OBP3	384	GEDO010000010.1	gb|AFG72998.1| odorant-binding protein 1 [Cnaphalocrocis medinalis]	235	6e–78	84%	0.15	0.14
Unigene_33249	OBP4^*^	435	GEDO010000011.1	gb|AGP03455.1| odorant-binding protein 9 [Spodoptera exigua]	111	3e–29	50%	7.80	2.12
Unigene_32695	OBP5	522	GEDO010000012.1	gb|AER27567.1| odorant binding protein [Chilo suppressalis]	191	1e–59	62%	812.60	2059.91
Unigene_11213	OBP6	420	GEDO010000013.1	gb|AGI37362.1| general odorant-binding protein 3 [Cnaphalocrocis medinalis]	228	4e–75	80%	6061.33	6056.06
Unigene_34662	OBP7	837	GEDO010000014.1	gb|AER27567.1| odorant binding protein [Chilo suppressalis]	290	8e–44	49%	1273.48	687.08
Unigene_25150	OBP8^*^	417	GEDO010000015.1	gb|AGI37366.1| general odorant-binding protein 2 [Cnaphalocrocis medinalis]	226	2e–74	88%	15576.65	4538.34
Unigene_33044	PBP1	483	GEDO010000018.1	gb|AGS46557.1| pheromone binding protein 1 [Maruca vitrata]	257	8e–86	75%	5223.90	8946.71
Unigene_31490	PBP2^*^	510	GEDO010000019.1	gb|BAG71419.1|pheromone binding protein [Diaphania indica]	249	1e–82	74%	44872.29	2143.21
Unigene_29089	PBP3	570	GEDO010000020.1	gb|ACF48467.1| pheromone binding protein female 1 [Loxostege sticticalis]	186	1e–57	70%	5402.32	4224.34
Unigene_33607	PBP4	486	GEDO010000021.1	gb|AGI37368.1| pheromone binding protein 4 [Cnaphalocrocis medinalis]	224	4e–73	69%	2130.20	1742.68
Unigene_37211	GOBP1	522	GEDO010000016.1	gb|AFG72996.1| general odorant binding protein 1 [Cnaphalocrocis medinalis]	243	7e–80	83%	2121.17	7257.42
Unigene_33256	GOBP2	483	GEDO010000017.1	gb|AIN41151.1| general odorant-binding protein 2 [Maruca vitrata]	311	5e–107	91%	19358.14	33411.03
Unigene_34301	ABP	432	GEDO010000022.1	gb|AAL60415.1| antennal binding protein 4 [Manduca sexta]	206	2e–66	67%	172.80	305.29

**Table 4:** Candidate OR genes in *Conogethes punctiferalis* antennae.

Unigene	Gene name	ORF (bp)	Accession number	BLASTx annotation	Score	*E*-value	Identify	RPKM Value	
								Male	Female
Unigene_10429	OR1	555	GEDO010000024.1	gb|BAR43480.1| putative olfactory receptor 38 [Ostrinia furnacalis]	173	3e–48	45%	1.36	1.11
Unigene_35486	OR2	1203	GEDO010000025.1	gb|NP001157210.1| olfactory receptor 17 [Bombyx mori]	334	3e–107	44%	10.88	17.06
Unigene_11235	OR3	420	GEDO010000026.1	gb|BAR43481.1| putative olfactory receptor 39 [Ostrinia furnacalis]	99	1e–21	39%	0	1.51
Unigene_38154	OR4	1170	GEDO010000027.1	gb|BAR43452.1| putative olfactory receptor 10 [Ostrinia furnacalis]	471	4e–161	59%	13.85	26.63
Unigene_6365	OR5^*^	501	GEDO010000028.1	gb|NP001296037.1| odorant receptor 13a-like [Plutella xylostella]	172	2e–48	54%	0.4	1.76
Unigene_47068	OR6	282	GEDO010000029.1	gb|ALM26253.1| gustatory receptor 3, partial [Athetis dissimilis]	155	7e–45	77%	0.73	0.60
Unigene_31536	OR7	1185	GEDO010000030.1	gb|AGK90020.1| olfactory receptor 17 [Helicoverpa assulta]	462	1e–157	64%	10.3	19.54
Unigene_37424	OR8	1164	GEDO010000031.1	gb|.XP0143628661| odorant receptor 46a, isoform A-like [Papilio machaon]	483	6e–166	63%	4.37	13.62
Unigene_21797	OR9	978	GEDO010000032.1	gbXP013186820|.1| gustatory and odorant receptor 22-like [Amyelois transitella]	580	0.0	89%	0.84	1.33
Unigene_39046	OR10	1296	GEDO010000033.1	gb|BAR43467.1| putative olfactory receptor 25 [Ostrinia furnacalis]	672	0.0	79%	7.4	8.04
Unigene_39333	OR11	1272	GEDO010000034.1	gb|NP001103476.1| olfactory receptor 35 [Bombyx mori]	388	7e–128	52%	16.46	25.88
Unigene_34286	OR12	1161	GEDO010000035.1	gb|BAR43487.1| putative olfactory receptor 45 [Ostrinia furnacalis]	422	4e–142	57%	6.53	10.24
Unigene_33960	OR13	1230	GEDO010000036.1	gb|ALM26238.1| odorant receptor 53 [Athetis dissimilis]	454	3e–154	55%	1.40	5.45
Unigene_35288	OR14	1368	GEDO010000037.1	gb|BAR43460.1| putative olfactory receptor 18 [Ostrinia furnacalis]	626	0.0	73%	5.94	11.87
Unigene_41196	OR15	201	GEDO010000038.1	gb|BAR43490.1| putative olfactory receptor 48 [Ostrinia furnacalis]	142	9e–40	97%	0	1.42
Unigene_30767	OR16^*^	1322	GEDO010000039.1	gb|BAR43476.1| putative olfactory receptor 34 [Ostrinia furnacalis]	412	7e–137	51%	0.36	13.29
Unigene_36352	OR17	1245	GEDO010000040.1	gb|BAR43461.1| putative olfactory receptor 19 [Ostrinia furnacalis]	290	1e–89	40%	16.96	1.18
Unigene_33377	OR18	1245	GEDO010000041.1	gb|BAR43468.1| putative olfactory receptor 19 [Ostrinia furnacalis]	525	0.0	65%	1.01	4.15
Unigene_36402	OR19	1269	GEDO010000042.1	gb|BAR43488.1| putative olfactory receptor 46 [Ostrinia furnacalis]	706	0.0	79%	5.08	9.87
Unigene_35705	OR20	807	GEDO010000043.1	gb|BAR43491.1| putative olfactory receptor 49 [Ostrinia furnacalis]	401	4e–136	68%	3,71	6.37
Unigene_33043	OR21	1281	GEDO010000044.1	gb|ADB89183.1| olfactory receptor 6 [Ostrinia furnacalis]	315	2e–99	44%	8.43	11.63
Unigene_32177	OR22^*^	1239	GEDO010000045.1	gb|BAR43471.1| putative olfactory receptor 29 [Ostrinia furnacalis]	474	2e–161	62%	197.27	4.26
Unigene_39439	OR23	1422	GEDO010000046.1	gb|AFG29886.1| odorant co-receptor [Conogethes punctiferalis]	951	0.0	99%	319.92	531.37
Unigene_35755	OR24	1206	GEDO010000047.1	gb|BAR43452.1|olfactory receptor 10 [Ostrinia furnacalis]	424	1e–142	56%	1.37	2.80
Unigene_22804	OR25^*^	1245	GEDO010000048.1	gb|ADB89180.1| olfactory receptor 3 [Ostrinia furnacalis]	308	1e–96	37%	0.57	1.98
Unigene_29130	OR26^*^	1278	GEDO010000049.1	gb|AIT71991.1| olfactory receptor 22 [Ctenopseustis obliquana]	303	1e–94	43%	233.32	4.77
Unigene_33708	OR27	1005	GEDO010000050.1	gb|BAR43475.1| putative olfactory receptor 33 [Ostrinia furnacalis]	533	0.0	82%	3.95	5.46
Unigene_36273	OR28	1170	GEDO010000051.1	gb|AII01045.1| odorant receptor [Dendrolimus houi]	354	2e–115	44%	2.08	5.45
Unigene_9909	OR29	306	GEDO010000052.1	gb|BAJ61939.1| odorant receptor [Ostrinia nubilalis]	75.1	1e–13	41%	0	1.84
Unigene_34694	OR30	1203	GEDO010000053.1	gb|BAR43467.1| putative olfactory receptor 25 [Ostrinia furnacalis]	410	3e–137	51%	7.64	12.86
Unigene_29284	OR31	1224	GEDO010000054.1	gb|BAR43494.1| putative olfactory receptor 52 [Ostrinia furnacalis]	491	1e–168	56%	1.99	6.58
Unigene_35553	OR32	1224	GEDO010000055.1	gb|BAR43494.1| putative olfactory receptor 52 [Ostrinia furnacalis]	508	4e–175	61%	3.33	5.20
Unigene_31835	OR33	984	GEDO010000056.1	gb|BAR43484.1| putative olfactory receptor 42 [Ostrinia furnacalis]	414	2e–140	63%	1.30	4.47
Unigene_37901	OR34	1260	GEDO010000057.1	gb|BAR43458.1| putative olfactory receptor 16 [Ostrinia furnacalis]	502	2e–172	60%	5.95	16.00
Unigene_30980	OR35	1230	GEDO010000058.1	gb|KOB74670.1|Odorant receptor 50 [Operophtera brumata]	479	6e–164	53%	1.46	3.59
Unigene_32663	OR36	1194	GEDO010000059.1	gb|BAR43480.1| putative olfactory receptor 38 [Ostrinia furnacalis]	411	1e–137	49%	11.15	14.14
Unigene_32039	OR37	1188	GEDO010000060.1	gb|NP001166611.1| olfactory receptor 59 [Bombyx mori]	363	1e–118	47%	20.16	42.48
Unigene_30358	OR38	1167	GEDO010000061.1	gb|BAR43453.1| putative olfactory receptor 11 [Ostrinia furnacalis]	494	1e–170	68%	6.25	6.33
Unigene_35167	OR39	1248	GEDO010000062.1	gb|BAR43456.1| putative olfactory receptor 14 [Ostrinia furnacalis]	548	0.0	64%	8.24	22.00
Unigene_29815	OR40	1203	GEDO010000063.1	gb|BAR43469.1| putative olfactory receptor 27 [Ostrinia furnacalis]	647	0.0	86%	7.23	8.41
Unigene_34345	OR41	1215	GEDO010000064.1	gb|BAR43481.1| putative olfactory receptor 39 [Ostrinia furnacalis]	248	4e–74	34%	5.11	10.70
Unigene_34297	OR42^*^	969	GEDO010000065.1	gb|AII01110.1|odorant receptor [Dendrolimus kikuchii]	386	4e–129	53%	0.05	9.88
Unigene_37409	OR43	1275	GEDO010000066.1	gb|NP001292415.1|odorant receptor 13a-like [Plutella xylostella]	307	3e–96	40%	6.69	17.05
Unigene_33544	OR44^*^	1200	GEDO010000067.1	gb|BAR43461.1| putative olfactory receptor 19 [Ostrinia furnacalis]	280	4e–86	43%	46.14	4.43
Unigene_36203	OR45	1275	GEDO010000068.1	gb|ADB89183.1| odorant receptor 6 [Ostrinia nubilalis]	342	1e–109	42%	9.31	12.66
Unigene_35759	OR46^*^	1191	GEDO010000069.1	gb|BAR43470.1| putative olfactory receptor 28 [Ostrinia furnacalis]	446	1e–150	50%	86.74	4.77

**Table 5:** Candidate IR genes in *Conogethes punctiferalis* antennae.

Unigene	Gene name	ORF (bp)	Accession number	BLASTx annotation	Score	*E*-value	Identify	RPKM Value	
								Male	Female
Unigene_32271	IR1	1278	GEDO01000001.1	gb|ADR64682.1| putative chemosensory ionotropic receptor IR68a [Spodoptera littoralis]	499	2e–167	77%	1.25	1.91
Unigene_39471	IR2	2727	GEDO01000002.1	gb|BAR64796.1| ionotropic receptor [Ostrinia furnacalis]	1493	0.0	81%	14.74	54.32
Unigene_33510	IR3	1899	GEDO01000003.1	gb|BAR64800.1| ionotropic receptor [Ostrinia furnacalis]	874	0.0	71%	1.25	6.29
Unigene_36510	IR4	1923	GEDO01000004.1	gb|BAR64803.1| ionotropic receptor [Ostrinia furnacalis]	821	0.0	68%	7.07	20.83
Unigene_37845	IR5	1926	GEDO01000005.1	gb|BAR64808.1| ionotropic receptor [Ostrinia furnacalis]	994	0.0	78%	2.35	6.38
Unigene_35392	IR6	2556	GEDO01000006.1	gb|BAR64797.1| ionotropic receptor [Ostrinia furnacalis]	1352	0.0	81%	15.61	17.66
Unigene_30586	IR7	1644	GEDO01000007.1	gb|BAR64809.1| ionotropic receptor [Ostrinia furnacalis]	875	0.0	77%	13.63	45.88

## References

[b1] Xiao W., Matsuyama S., Ando T., Millar J. G. & Honda H. Unsaturated cuticular hydrocarbons synergize responses to sex attractant pheromone in the yellow peach moth, Conogethes punctiferalis. J. Chem. Ecol. 38, 1143–1150 (2012). 22903747 10.1007/s10886-012-0176-9

[b2] Li D. Y., Ai P. P., Du Y. L., Sun S. L. & Zhang M. Z. Effects of different host plants on the development and reproduction of yellow peach moth, *Conogethes punctiferalis* (Guenée) (Lepidoptera; Crambidae). Austr. Entomol. 54, 149–153 (2015).

[b3] Honda H., Ishiwatari T. & Matsumoto Y. Fungal volatiles as oviposition attractants for the yellow peach moth, *Conogethes punctiferalis* (Guenée) (Lepidoptera: Pyralidae). J. Insect Physiol. 34, 205–211 (1988).

[b4] Du Y. L. . Volatiles from involucres of chestnut and their potential applications in control of *Conogethes punctiferalis*. Chin. J. Ecol. 33, 2096–2100 (2014b). (In Chinese).

[b5] Liu M. Y., Tian Y. & Li X. Identification of minor components of the sex pheromone of yellow peach moth, *Dichocrocis punctiferalis* Guenée, and field trials. Entomol. Sin. 1, 150–155 (1994).

[b6] Konno Y. . (E)-10-hexadecenal, a sex pheromone component of the yellow peach moth, *Dichocrocis punctiferalis* Guenée (Lepidoptera: Pyralidae). Appl. Entomol. Zool. 17, 207–217 (1982).

[b7] Du Y. L. . Formulation screening of sex pheromones and field trapping tests for the yellow peach moth, *Conogethes punctiferalis* (Guenée) (Lepidoptera: Pyralidae). Acta Phytophy. Sin. 41, 187–191 (2014a). (In Chinese).

[b8] Dong Y. Z., Chen B. X., Xu S., Li P. Y. & Zhang X. W. Analysis of volatiles from fruit and leaf of insect-resistant and insect-susceptible chestnut cultivars with GC-MS. J. Fruit Sci. 29, 1052–1056 (2012). (In Chinese).

[b9] Field L. M., Pickett J. A. & Wadhams L. J. Molecular studies in insect olfaction. Insect. Mol. Biol. 9, 545–551 (2000). 11122463 10.1046/j.1365-2583.2000.00221.x

[b10] Pitts R., Rinker D., Jones P., Rokas A. & Zwiebel L. Transcriptome profiling of chemosensory appendages in the malaria vector *Anopheles gambiae* reveals tissue-and sex-specific signatures of odor coding. BMC Genomics 12, 271 (2011). 21619637 10.1186/1471-2164-12-271 PMC3126782

[b11] Witzgall P., Kirsch P. & Cork A. Sex pheromones and their impact on pest management. J. Chem. Ecol. 36, 80–100 (2010). 20108027 10.1007/s10886-009-9737-y

[b12] Bruyne M. & Baker T. C. Odor detection in insects: volatile codes. J. Chem. Ecol., 34, 882–897 (2008). 18535862 10.1007/s10886-008-9485-4

[b13] Korsching S. Olfactory maps and odor images. Current Opin. Neurobiol. 12, 387–392 (2002). 10.1016/s0959-4388(02)00348-3 12139985

[b14] Leal W. S. Odorant reception in insects: roles of receptors, binding proteins, and degrading enzymes. Annu. Rev. Entomol. 58, 373–379 (2013). 23020622 10.1146/annurev-ento-120811-153635

[b15] Pelosi P., Zhou J. J., Ban L. P. & Calvello M. Soluble proteins in insect chemical communication. Cell Mol. Life Sci. 63, 1658–1676 (2006). 16786224 10.1007/s00018-005-5607-0 PMC11136032

[b16] Zhou J. J. & Gerald L. Odorant-binding proteins in insects. Vitam. Horm. 83, 241–272 (2010). 20831949 10.1016/S0083-6729(10)83010-9

[b17] Gong D. P., Zhang H. J., Zhao P., Xia Q. Y. & Xiang Z. H. The odorant binding protein gene family from the genome of silkworm, Bombyx mori. BMC Genomics 10, 332 (2009). 19624863 10.1186/1471-2164-10-332 PMC2722677

[b18] Zhang T. T. . Male- and female-biased gene expression of olfactory-related genes in the antennae of asian corn borer, *Ostrinia furnacalis* (Guenée) (Lepidoptera: Crambidae). PLoS ONE 10, e0128550 (2015). 26062030 10.1371/journal.pone.0128550 PMC4463852

[b19] Gu S. H. . Identification and comparative expression analysis of odorant binding protein genes in the tobacco cutworm *Spodoptera litura*. Sci. Rep. 5, e13800 (2015). 10.1038/srep13800 PMC4561897 26346731

[b20] Vogt R. G. & Lerner M. R. Two groups of odorant binding proteins in insects suggest specific and general olfactory pathways. Neurosci. Abst. 15, 1290 (1989).

[b21] Vogt R. G. Biochemical diversity of odor detection: OBPs, ODEs and SNMPs. In: Insect pheromone biochemistry and molecular biology, eds. Blomquist G. J. & Vogt R. G.Elsevier Academic Press, London, pp 391–445 (2003).

[b22] Jacquin-Joly E. & Merlin C. Insect olfactory receptors: contributions of molecular biology to chemical ecology. J. Chem. Ecol. 30, 2359–2397 (2004). 15724962 10.1007/s10886-004-7941-3

[b23] Rogers M. E., Krieger J. & Vogt R. G. Antennal SNMPs (sensory neuron membrane proteins) of Lepidoptera define a unique family of invertebrate CD36-like proteins. J. Neurobiol. 49, 47–61 (2001). 11536197 10.1002/neu.1065

[b24] Leal W. S. Pheromone reception. Top. Curr. Chem. 240, 1–36 (2005).

[b25] Benton R., Vannice K. S. & Vosshall L. B. An essential role for a CD36-related receptor in pheromone detection in *Drosophila*. Nature 450, 289–293 (2007). 17943085 10.1038/nature06328

[b26] Ansorge W. J. Next-generation DNA sequencing techniques. New Biotechnol. 25, 195–203 (2009). 10.1016/j.nbt.2008.12.009 19429539

[b27] Costa V., Angelini C., De-Feis I. & Ciccodicola A. Uncovering the complexity of transcriptomes with RNA-seq. J. Biomed. Biotechnol. 853916 (2010). 20625424 10.1155/2010/853916 PMC2896904

[b28] Bengtsson J. M. . Putative chemosensory receptors of the codling moth, *Cydia pomonella*, identified by antennal transcriptome analysis. PLoS ONE 7, e31620 (2012). 22363688 10.1371/journal.pone.0031620 PMC3282773

[b29] Lundberg M. . Characterisation of a transcriptome to find sequence differences between two differentially migrating subspecies of the willow warbler *Phylloscopus trochilus*. BMC Genomics 14, 330 (2013). 23672489 10.1186/1471-2164-14-330 PMC3660185

[b30] Grosse-Wilde E. . Antennal transcriptome of *Manduca sexta*. Proc. Natl. Acad. Sci. 108, 7449–7454 (2011). 21498690 10.1073/pnas.1017963108 PMC3088587

[b31] Legeai F. . An expressed sequence tag collection from the male antennae of the noctuid moth *Spodoptera littoralis*: a resource for olfactory and pheromone detection research. BMC Genomics 12, 86 (2011). 21276261 10.1186/1471-2164-12-86 PMC3045336

[b32] Zhang Y. N. . Differential expression patterns in chemosensory and non-chemosensory tissues of putative chemosensory genes identified by transcriptome analysis of insect pest the purple stem borer *Sesamia inferens* (Walker). PLoS ONE 8, e69715 (2013). 23894529 10.1371/journal.pone.0069715 PMC3722147

[b33] Ge X. . Cloning and tissue expression profiling of the olfactory receptor coreceptor gene in adults of *Conogethes punctiferalis* (Lepidoptera: Crambidae). Acta Entomol. Sin. 56, 243–250 (2013). (In Chinese).

[b34] Jia X. J. . cDNA cloning, expression profiling and binding affinity assay of the pheromone binding protein Cpun-PBP1 in the yellow peach moth, *Conogethes punctiferalis* (Lepidoptera: Crambidae). Acta Entomol. Sin. 58, 1167–1176 (2015). (In Chinese).

[b35] Cao D. P. . Identification of candidate olfactory genes in *Chilo suppressalis* by antennal transcriptome analysis. Int. J. Biol. Sci. 10, 846–860 (2014). 25076861 10.7150/ijbs.9297 PMC4115196

[b36] Chen E. H. . De novo characterization of the *Dialeurodes citri* transcriptome: mining genes involved in stress resistance and simple sequence repeats (SSRs) discovery. Insect Mol. Biol. 23, 52–66 (2014). 24164346 10.1111/imb.12060

[b37] Hou R. . Transcriptome sequencing and *de novo* analysis for Yesso scallop (*Patinopecten yessoensis*) using 454 GS FLX. PLoS ONE 6, e21560 (2011). 21720557 10.1371/journal.pone.0021560 PMC3123371

[b38] Vogel H., Heidel A. J., Heckel D. G. & Groot A. T. Transcriptome analysis of the sex pheromone gland of the noctuid moth *Heliothis virescens*. BMC Genomics 11, 29 (2010). 20074338 10.1186/1471-2164-11-29 PMC2820457

[b39] ÓhÉigeartaigh S., Armisén D., Byrne K. P. & Wolfe K. H. Systematic discovery of unannotated genes in 11 yeast species using a database of orthologous genomic segments. BMC Genomics 12, 377 (2011). 21791067 10.1186/1471-2164-12-377 PMC3161974

[b40] Ibarra-Soria X., Levitin M. O., Saraiva L. R. & Logan D. W. The olfactory transcriptomes of mice. PLoS Genetics 10, 821–833 (2014). 10.1371/journal.pgen.1004593 PMC4154679 25187969

[b41] Jacquin-Joly E. . Candidate chemosensory genes in female antennae of the noctuid moth *Spodoptera littoralis*. Int. J. Biol. Sci. 8, 1036–1050 (2012). 22904672 10.7150/ijbs.4469 PMC3421235

[b42] Gu S. H. . Molecular characterization and differential expression of olfactory genes in the antennae of the black cutworm moth *Agrotis ipsilon*. PLoS ONE 9, e103420 (2014). 25083706 10.1371/journal.pone.0103420 PMC4118888

[b43] Pelosi P. & Maida R. Odorant-binding proteins in insects.*Comp. Biochem. Physiol*. B 3, 503–514 (1995). 10.1016/0305-0491(95)00019-5 7613772

[b44] Liu Y., Gu S., Zhang Y., Guo Y. & Wang G. Candidate olfaction genes identified within the *Helicoverpa armigera* antennal transcriptome. PLoS ONE 7, e48260 (2012). 23110222 10.1371/journal.pone.0048260 PMC3482190

[b45] Zhang S. F., Zhang Z., Wang H. B. & Kong X. B. Antennal transcriptome analysis and comparison of olfactory genes in two sympatric defoliators, *Dendrolimus houi* and *Dendrolimus kikuchii* (Lepidoptera: Lasiocampidae). Insect Biochem. Mol. Biol. 52, 69–81 (2015). 10.1016/j.ibmb.2014.06.006 24998398

[b46] Zhu J. Y., Zhang L. F., Ze S. Z., Wang D. W. & Yang B. Identification and tissue distribution of odorant binding protein genes in the beet armyworm, Spodoptera exigua. J. Insect Physiol. 59, 722–728 (2013). 23499610 10.1016/j.jinsphys.2013.02.011

[b47] Wanner K. W., Isman M. B., Feng Q., Plettner E. & Theilmann D. A. Developmental expression patterns of four chemosensory protein genes from the Eastern spruce budworm, Chroistoneura fumiferana. Insect Mol. Biol. 14, 289–300 (2005). 15926898 10.1111/j.1365-2583.2005.00559.x

[b48] Xia Y. . The molecular and cellular basis of olfactory-driven behavior in *Anopheles gambiae* larvae. PNAS 105, 6433–6438 (2008). 18427108 10.1073/pnas.0801007105 PMC2359781

[b49] Andersson M. N. . Antennal transcriptome analysis of the chemosensory gene families in the tree killing bark beetles, *Ips typographus* and *Dendroctonus ponderosae* (Coleoptera: Curculionidae: Scolytinae). BMC Genomics 14, 198 (2013). 23517120 10.1186/1471-2164-14-198 PMC3610139

[b50] Wanner K. W. . Female-biased expression of odorant receptor genes in the adult antennae of the silkworm, Bombyx mori. *Insect Mol. Biol.* 16, 107–119 (2007). 17257213 10.1111/j.1365-2583.2007.00708.x

[b51] Anderson A. R. . Molecular basis of female-specific odorant responses in *Bombyx mori*. Insect Biochem. Mol. Biol. 39, 189–197 (2009). 19100833 10.1016/j.ibmb.2008.11.002

[b52] Tanaka K. . Highly selective tuning of a silkworm olfactory receptor to a key mulberry leaf volatile. Curr. Biol. 19, 881–890 (2009). 19427209 10.1016/j.cub.2009.04.035

[b53] Benton R., Vannice K. S., Gomez-Diaz C. & Vosshall L. B. Variant ionotropic glutamate receptors as chemosensory receptors in *Drosophila*. Cell 136, 149–162 (2009). 19135896 10.1016/j.cell.2008.12.001 PMC2709536

[b54] Croset V. . Ancient protostome origin of chemosensory ionotropic glutamate receptors and the evolution of insect taste and olfaction. PLoS Genet. 6, e1001064 (2010). 20808886 10.1371/journal.pgen.1001064 PMC2924276

[b55] Olivier V., Monsempes C., Francois M. C., Poivet E. & Jacquin-Joly E. Candidate chemosensory ionotropic receptors in a Lepidoptera. Insect. Mol. Biol. 20, 189–199 (2011). 21091811 10.1111/j.1365-2583.2010.01057.x

[b56] Meyer E. . Sequencing and *de novo* analysis of a coral larval transcriptome using 454 GSFlx. BMC Genomics 10, 219 (2009). 19435504 10.1186/1471-2164-10-219 PMC2689275

[b57] Grabherr M. G. . Full-length transcriptome assembly from RNA-seq data without a reference genome. Nat. Biotechnol. 29, 644–652 (2011). 21572440 10.1038/nbt.1883 PMC3571712

[b58] Liu M. Y. . Transcriptome sequencing and *de novo* analysis for Ma Bamboo (*Dendrocalamus latiflorus* Munro) using the Illumina platform. PLoS ONE 7, e46766 (2012). 23056442 10.1371/journal.pone.0046766 PMC3463524

[b59] Altschul S. F. . Gapped BLAST and PSI-BLAST: a new generation of protein database search programs. Nucleic. Acids. Res. 25, 3389–3402 (1997). 9254694 10.1093/nar/25.17.3389 PMC146917

[b60] Conesa A. & Götz S. Blast2GO: a comprehensive suite for functional analysis in plant genomics. Int. J. Plant Genomics 619832 (2008). 18483572 10.1155/2008/619832 PMC2375974

[b61] Ashburner M. . Gene ontology: tool for the unification of biology. Nat. Genet. 25, 25–29 (2000). 10802651 10.1038/75556 PMC3037419

[b62] Kanehisa M. . KEGG for linking genomes to life and the environment. Nucleic Acids Res. 36, D480–D484 (2008). 18077471 10.1093/nar/gkm882 PMC2238879

[b63] Tamura K. . MEGA5: molecular evolutionary genetics analysis using maximum likelihood, evolutionary distance, and maximum parsimony methods. Mol. Biol. Evol. 28, 2731–2739 (2011). 21546353 10.1093/molbev/msr121 PMC3203626

[b64] Mortazavi A., Williams B. A., McCue K., Schaeffer L. & Wold B. Mapping and quantifying mammalian transcriptomes by RNA-Seq. Nat. Methods 5, 621–628 (2008). 18516045 10.1038/nmeth.1226 PMC13303166

[b65] Livak K. J. & Schmittgen T. D. Analysis of relative gene expression data using real-time quantitative PCR and the 2^−ΔΔCt^ method. Methods 25, 402–408 (2001). 11846609 10.1006/meth.2001.1262

