# Supplementary material for: Antennal transcriptome and differential expression of olfactory genes in the yellow peach moth, Conogethes punctiferalis (Lepidoptera: Crambidae)
Source: Sci Rep. 2016 Jul 1;6:29067. doi: 10.1038/srep29067 (PMC4929561; doi:10.1038/srep29067)
Supplement: Supplementary Information [file srep29067-s1.pdf]

Supplementary Information for

Antennal transcriptome and differential expression of olfactory genes in the yellow peach moth, *Conogethes punctiferalis* (Lepidoptera: Crambidae)

Xiao-Jian Jia[1], Hai-Xiang Wang[2], Zeng-Guang Yan[3], Min-Zhao Zhang[1], Chun-Hua Wei[1], Xiao-Chun Qin[1], Wei-Rong Ji[2], Patrizia Falabella[4] & Yan-Li Du[1*]

[1]Beijing Key Laboratory for Agricultural Application and New Technique, College of Plant Science and Technology, Beijing University of Agriculture, Beijing 102206, China;

[2]College of Forestry, Shanxi Agricultural University, Taigu, Shanxi 030801, China;

[3]State Key Laboratory of Environmental Criteria and Risk Assessment, Chinese Research Academy of Environmental Sciences, Beijing 100012, China;

[4]Dipartimento di Scienze, Università della Basilicata, Campus Macchia Romana, Via dell'Ateneo Lucano, 10, Potenza 85100, Italy.

[*]Corresponding author:

Yan-Li Du, PhD

Beijing University of Agriculture,

Beinong Road 7#, Beijing, 102206, P. R. China.

Tel: +86 10 80799135

Fax: +86 10 80796917

E-mail: yanlidu@126.com

Supplementary Tables: 6

Supplementary Table 1 | Functional gene annotations of contigs from the combined antennal references assembly.

Supplementary Table 2 | The nucleotide sequences of 15 OBPs, 46 ORs and 7 IRs identified in the present study.

Supplementary Table 3 | Primers for RT-PCR expression analyses of *Conogethes punctiferalis* OBPs, ORs, and IRs.

Supplementary Table 4 | Protein names and sequences of the 126 OBPs used in Figure 5.

Supplementary Table 5 | Protein names and sequences of the 130 ORs used in Figure 7.

Supplementary Table 6 | Protein names and sequences of the 37 IRs used in Figure 9.

Supplementary Table 1.

Gene ID	COG class	COG class annotation	GO annotation	KEGG annotation	Swissprot annotation	Nr annotation
c32154.graph_c1	--	--	--	--	Pheromone-binding protein-related protein 5 (Precursor) GN=Pbprp5 OS=Drosophila melanogaster (Fruit fly) PE=2 SV=2	antennal binding protein [Danaus plexippus]
c24192.graph_c0	--	--	Molecular Function: odorant binding (GO:0005549);;	--	General odorant-binding protein lush (Precursor) GN=lush OS=Drosophila melanogaster (Fruit fly) PE=1 SV=1	odorant-binding protein 2 [Cnaphalocrocis medinalis]
c26427.graph_c1	--	--	Molecular Function: odorant binding (GO:0005549);;	--	General odorant-binding protein 19a (Precursor) GN=Obp19a OS=Drosophila melanogaster (Fruit fly) PE=2 SV=1	odorant-binding protein 1 [Cnaphalocrocis medinalis]
c33249.graph_c0	--	--	--	--	General odorant-binding protein 56d (Precursor) GN=Obp56d OS=Drosophila melanogaster (Fruit fly) PE=1 SV=2	SexiOBP9 [Spodoptera exigua]
c32695.graph_c0	--	--	Molecular Function: odorant binding (GO:0005549);;	--	Pheromone-binding protein-related protein 2 (Precursor) GN=Pbprp2 OS=Drosophila melanogaster (Fruit fly) PE=2 SV=2	odorant binding protein [Chilo suppressalis]
c11213.graph_c0	--	--	Molecular Function: odorant binding (GO:0005549);;	--	Pheromone-binding protein-related protein 3 (Precursor) GN=Pbprp3 OS=Drosophila melanogaster (Fruit fly) PE=2 SV=1	general odorant-binding protein 3 [Cnaphalocrocis medinalis]

c34662.graph_c0	--	--	--	--	Pheromone-binding protein-related protein 2 (Precursor) GN=Pbprp2 OS=Drosophila melanogaster (Fruit fly) PE=2 SV=2	odorant binding protein [Chilo suppressalis]
c25150.graph_c0	--	--	Molecular Function: odorant binding (GO:0005549);;	--	General odorant-binding protein 19a (Precursor) GN=Obp19a OS=Drosophila melanogaster (Fruit fly) PE=2 SV=1	general odorant-binding protein 2 [Cnaphalocrocis medinalis]
c37211.graph_c0	--	--	--	K14026|9e-07|api:100162766|similar to AGAP000615-PA	Endonuclease (Fragment) OS=Sciara coprophila (Fungus gnat) PE=4 SV=1	PREDICTED: hypothetical protein LOC100573199 [Acyrthosiphon pisum]
c33256.graph_c0	--	--	Molecular Function: odorant binding (GO:0005549);; Biological Process: transport (GO:0006810);; Biological Process: sensory perception of smell (GO:0007608);; Biological Process: response to stimulus (GO:0050896);;	--	General odorant-binding protein 2 (Precursor) OS=Antheraea pernyi (Chinese oak silk moth) PE=2 SV=1	general odorant binding protein 2 [Cnaphalocrocis medinalis]
c34301.graph_c1	--	--	Molecular Function: odorant binding (GO:0005549);;	--	General odorant-binding protein lush (Precursor) GN=lush OS=Drosophila melanogaster (Fruit fly) PE=1 SV=1	antennal binding protein 4 [Manduca sexta]
c33044.graph_c0	--	--	Molecular Function: odorant binding (GO:0005549);; Biological Process: transport (GO:0006810);;	--	Pheromone-binding protein (Precursor) OS=Bombyx mori (Silk moth) PE=1 SV=2	pheromone-binding protein 1 [Cnaphalocrocis medinalis]
c31490.graph_c0	--	--	Molecular Function: odorant binding (GO:0005549);; Biological Process: transport (GO:0006810);;	--	Pheromone-binding protein (Precursor) OS=Bombyx mori (Silk moth) PE=1 SV=2	pheromone binding protein [Diaphania indica]

c29089.graph_c0	--	--	Molecular Function: odorant binding (GO:0005549);;	--	Pheromone-binding protein-related protein 4 (Precursor) GN=Pbprp4 OS=Drosophila melanogaster (Fruit fly) PE=2 SV=1	pheromone binding protein 3 [Cnaphalocrocis medinalis]
c33607.graph_c0	--	--	Molecular Function: odorant binding (GO:0005549);; Biological Process: transport (GO:0006810);;	--	General odorant-binding protein 1 (Precursor) OS=Epiphyas postvittana (Light brown apple moth) PE=1 SV=1	pheromone binding protein 4 [Cnaphalocrocis medinalis]
c10429.graph_c0	--	--	--	--	--	odorant receptor 65, partial [Manduca sexta]
c35486.graph_c0	--	--	--	K08471|4e-11|tca:100141798|Or25	Putative odorant receptor 13a GN=Or13a OS=Drosophila melanogaster (Fruit fly) PE=3 SV=2	olfactory receptor 17 [Bombyx mori]
c11235.graph_c0	--	--	--	--	--	PREDICTED: odorant receptor Or1-like [Bombyx mori]
c38154.graph_c0	--	--	Biological Process: intracellular protein transport (GO:0006886);; Biological Process: retrograde vesicle-mediated transport, Golgi to ER (GO:0006890);; Cellular Component: COPI vesicle coat (GO:0030126);; Cellular Component: clathrin adaptor complex (GO:0030131);;	--	Probable coatomer subunit delta GN=C13B9.3 OS=Caenorhabditis elegans PE=3 SV=1	coatomer protein complex subunit delta [Bombyx mori]
c6365.graph_c0	--	--	--	K08471|3e-08|nvi:100328547|Or82, NV17546, NvOr82	Odorant receptor 43b GN=Or43b OS=Drosophila melanogaster (Fruit fly) PE=2 SV=3	odorant receptor 5 [Plutella xylostella]

c47068.graph_c0	--	--	Biological Process: detection of carbon dioxide (GO:0003031);; Molecular Function: olfactory receptor activity (GO:0004984);; Cellular Component: plasma membrane (GO:0005886);; Biological Process: G-protein coupled receptor signaling pathway (GO:0007186);; Biological Process: determination of adult lifespan (GO:0008340);; Molecular Function: taste receptor activity (GO:0008527);; Biological Process: response to carbon dioxide (GO:0010037);; Cellular Component: integral component of membrane (GO:0016021);; Biological Process: detection of chemical stimulus involved in sensory perception of smell (GO:0050911);; Biological Process: detection of chemical stimulus involved in sensory perception of taste (GO:0050912);;	K08471|2e-28|aag: AaeL_AAEL010058|Gustatory receptor 63a, putative	Gustatory and odorant receptor 24 GN=GPRgr24 OS=Anopheles gambiae (African malaria mosquito) PE=2 SV=4	gustatory receptor 24 [Danaus plexippus]
c31536.graph_c0	--	--	Biological Process: signal transduction (GO:0007165);; Biological Process: sensory perception of smell (GO:0007608);; Molecular Function: signaling receptor activity (GO:0038023);;	K08471|6e-35|tca:663463|similar to Putative odorant receptor 94a	Odorant receptor Or1 GN=OR1 OS=Anopheles gambiae (African malaria mosquito) PE=2 SV=1	olfactory receptor 17 [Helicoverpa assulta]

c37424.graph_c0	--	--	Molecular Function: transmembrane signaling receptor activity (GO:0004888);; Biological Process: sensory perception of smell (GO:0007608);; Cellular Component: membrane (GO:0016020);; Biological Process: response to stimulus (GO:0050896);;	K08471|2e-30|aga: AgaP_AGAP002126|GPROR37	Putative odorant receptor 71a GN=Or71a OS=Drosophila melanogaster (Fruit fly) PE=3 SV=4	olfactory receptor 13 [Bombyx mori]
c21797.graph_c0	--	--	Molecular Function: G-protein coupled receptor activity (GO:0004930);; Cellular Component: plasma membrane (GO:0005886);; Biological Process: G-protein coupled receptor signaling pathway (GO:0007186);; Cellular Component: integral component of membrane (GO:0016021);; Biological Process: sensory perception of taste (GO:0050909);;	--	Gustatory and odorant receptor 21a GN=Gr21a OS=Drosophila melanogaster (Fruit fly) PE=2 SV=3	PREDICTED: gustatory and odorant receptor 22-like [Bombyx mori]
c39046.graph_c0	--	--	Molecular Function: transmembrane signaling receptor activity (GO:0004888);; Biological Process: sensory perception of smell (GO:0007608);; Cellular Component: membrane (GO:0016020);; Biological Process: response to stimulus (GO:0050896);;	K08471|2e-50|aag: AaeL_AAEL012254|odorant receptor	Putative odorant receptor 92a GN=Or92a OS=Drosophila melanogaster (Fruit fly) PE=3 SV=3	putative chemosensory receptor 12 [Heliothis virescens]

c39333.graph_c0	--	--	Biological Process: sensory perception of smell (GO:0007608);;	--	Lachesin (Precursor) GN=LAC OS=Schistocerca americana (American grasshopper) PE=1 SV=1	odorant receptor 50, partial [Manduca sexta]
c34286.graph_c0	--	--	Molecular Function: transmembrane signaling receptor activity (GO:0004888);; Biological Process: sensory perception of smell (GO:0007608);; Cellular Component: membrane (GO:0016020);; Biological Process: response to stimulus (GO:0050896);;	K08471|9e-57|aag:AaeL_AAEL012254|odorant receptor	Putative odorant receptor 92a GN=Or92a OS=Drosophila melanogaster (Fruit fly) PE=3 SV=3	olfactory receptor 42 [Bombyx mori]
c33960.graph_c0	--	--	Biological Process: sensory perception of smell (GO:0007608);;	K08471|1e-23|tca:100141798|Or25	Odorant receptor Or2 GN=OR2 OS=Anopheles gambiae (African malaria mosquito) PE=2 SV=1	putative odorant receptor OR28 [Cydia pomonella]
c35288.graph_c0	--	--	Molecular Function: transmembrane signaling receptor activity (GO:0004888);; Biological Process: sensory perception of smell (GO:0007608);; Cellular Component: membrane (GO:0016020);; Biological Process: response to stimulus (GO:0050896);;	K08471|2e-34|aga:AgaP_AGAP002558|GPROR40	Putative odorant receptor 83a GN=Or83a OS=Drosophila melanogaster (Fruit fly) PE=3 SV=2	olfactory receptor-like [Bombyx mori]

c41196.graph_c0	--	--	Biological Process: detection of carbon dioxide (GO:0003031);; Molecular Function: olfactory receptor activity (GO:0004984);; Cellular Component: plasma membrane (GO:0005886);; Biological Process: G-protein coupled receptor signaling pathway (GO:0007186);; Molecular Function: taste receptor activity (GO:0008527);; Biological Process: response to carbon dioxide (GO:0010037);; Cellular Component: integral component of membrane (GO:0016021);; Cellular Component: dendrite (GO:0030425);; Cellular Component: neuronal cell body (GO:0043025);; Biological Process: detection of chemical stimulus involved in sensory perception of smell (GO:0050911);; Biological Process: detection of chemical stimulus involved in sensory perception of taste (GO:0050912);;	--	Gustatory and odorant receptor 22 GN=GPRgr22 OS=Anopheles gambiae (African malaria mosquito) PE=2 SV=1	PREDICTED: gustatory and odorant receptor 22-like [Bombyx mori]
c30767.graph_c0	--	--	Biological Process: sensory perception of smell (GO:0007608);;	K08471|4e-16|nvi:100463083|Or93, NvOr93	Putative odorant receptor 92a GN=Or92a OS=Drosophila melanogaster (Fruit fly) PE=3 SV=3	TPA: TPA_exp: odorant receptor 30 [Bombyx mori]

c36352.graph_c0	--	--	Molecular Function: signal transducer activity (GO:0004871);; Cellular Component: membrane (GO:0016020);; Biological Process: single-organism process (GO:0044699);; Biological Process: response to stimulus (GO:0050896);;	K08471|3e-42|aag: AaeL_AAEL000391|Odorant receptor 85d, putative	Putative odorant receptor 67c GN=Or67c OS=Drosophila melanogaster (Fruit fly) PE=1 SV=2	PREDICTED: LOW QUALITY PROTEIN: putative odorant receptor 85b-like [Bombyx mori]
c33377.graph_c0	--	--	--	--	Putative odorant receptor 92a GN=Or92a OS=Drosophila melanogaster (Fruit fly) PE=3 SV=3	PREDICTED: putative odorant receptor 85c-like [Bombyx mori]
c36402.graph_c0	--	--	--	K08471|3e-44|aag: AaeL_AAEL000391|Odorant receptor 85d, putative	Putative odorant receptor 92a GN=Or92a OS=Drosophila melanogaster (Fruit fly) PE=3 SV=3	olfactory receptor [Bombyx mori]
c35705.graph_c0	--	--	Molecular Function: olfactory receptor activity (GO:0004984);; Molecular Function: odorant binding (GO:0005549);; Cellular Component: membrane (GO:0016020);; Biological Process: detection of chemical stimulus involved in sensory perception of smell (GO:0050911);;	K08471|1e-08|dme: Dmel_CG13158|Or49a	Putative odorant receptor 49a GN=Or49a OS=Drosophila melanogaster (Fruit fly) PE=1 SV=3	olfactory receptor [Danaus plexippus]
c33043.graph_c0	--	--	Molecular Function: catalytic activity (GO:0003824);; Biological Process: metabolic process (GO:0008152);;	K08471|3e-13|tca:661659|odorant receptor	Putative odorant receptor 82a GN=Or82a OS=Drosophila melanogaster (Fruit fly) PE=3 SV=1	PREDICTED: uncharacterized protein LOC101736708 [Bombyx mori]

c32177.graph_c0	--	--	--	--	Putative odorant receptor 13a GN=Or13a OS=Drosophila melanogaster (Fruit fly) PE=3 SV=2	odorant receptor 6 [Ostrinia nubilalis]
c39439.graph_c0	--	--	Molecular Function: olfactory receptor activity (GO:0004984);; Molecular Function: odorant binding (GO:0005549);; Cellular Component: membrane (GO:0016020);; Biological Process: detection of chemical stimulus involved in sensory perception of smell (GO:0050911);;	K08471|0.0|aga:AgaP_AGAP002560|GPROR7	Gustatory and odorant receptor 7 GN=GPRor7 OS=Anopheles gambiae (African malaria mosquito) PE=2 SV=3	odorant co-receptor [Conogethes punctiferalis]
c35755.graph_c0	--	--	Cellular Component: anaphase-promoting complex (GO:0005680);;	K03348|2e-77|phu:Phum_PHUM603310|anaphase-promoting complex subunit, putative	Anaphase-promoting complex subunit 1 GN=anapc1 OS=Dictyostelium discoideum (Slime mold) PE=3 SV=1	PREDICTED: anaphase-promoting complex subunit 1-like, partial [Bombyx mori]
c22804.graph_c0	--	--	--	--	Odorant receptor 43b GN=Or43b OS=Drosophila melanogaster (Fruit fly) PE=2 SV=3	odorant receptor 51, partial [Manduca sexta]
c29130.graph_c0	--	--	--	--	Putative odorant receptor 13a GN=Or13a OS=Drosophila melanogaster (Fruit fly) PE=3 SV=2	odorant receptor [Ostrinia nubilalis]

c33708.graph_c0	--	--	Molecular Function: olfactory receptor activity (GO:0004984);; Molecular Function: odorant binding (GO:0005549);; Cellular Component: membrane (GO:0016020);; Biological Process: detection of chemical stimulus involved in sensory perception of smell (GO:0050911);;	K08471|1e-15|aag: AaeL_AAEL010669|olfactory receptor, putative	Putative odorant receptor 13a GN=Or13a OS=Drosophila melanogaster (Fruit fly) PE=3 SV=2	olfactory receptor-like [Bombyx mori]
c36273.graph_c0	--	--	--	--	--	PREDICTED: odorant receptor 46a, isoform A-like [Megachile rotundata]
c9909.graph_c0	--	--	--	--	--	odorant receptor 6 [Plutella xylostella]
c34694.graph_c0	--	--	Molecular Function: signal transducer activity (GO:0004871);; Cellular Component: membrane (GO:0016020);; Biological Process: single-organism process (GO:0044699);; Biological Process: response to stimulus (GO:0050896);;	K08471|1e-46|aag: AaeL_AAEL012254|odorant receptor	Putative odorant receptor 92a GN=Or92a OS=Drosophila melanogaster (Fruit fly) PE=3 SV=3	putative chemosensory receptor 12 [Heliothis virescens]

c29284.graph_c0	--	--	Molecular Function: signal transducer activity (GO:0004871);; Cellular Component: membrane (GO:0016020);; Biological Process: single-organism process (GO:0044699);; Biological Process: response to stimulus (GO:0050896);;	K08471|2e-22|nvi:100463087|Or101, NvOr101	Putative odorant receptor 85d GN=Or85d OS=Drosophila melanogaster (Fruit fly) PE=3 SV=1	putative odorant receptor OR17 [Cydia pomonella]
c35553.graph_c0	--	--	Biological Process: sensory perception of smell (GO:0007608);;	K08471|7e-14|dpo:Dpse_GA25554|Dpse_Or13a	Putative odorant receptor 13a GN=Or13a OS=Drosophila melanogaster (Fruit fly) PE=3 SV=2	olfactory receptor 20 [Helicoverpa assulta]
c31835.graph_c0	--	--	Molecular Function: olfactory receptor activity (GO:0004984);; Molecular Function: odorant binding (GO:0005549);; Cellular Component: membrane (GO:0016020);; Biological Process: detection of chemical stimulus involved in sensory perception of smell (GO:0050911);;	K08471|4e-22|nvi:100121399|Or96, NV16979, NvOr96	Putative odorant receptor 30a GN=Or30a OS=Drosophila melanogaster (Fruit fly) PE=3 SV=4	olfactory receptor 18 [Danaus plexippus]
c37901.graph_c0	--	--	Biological Process: sensory perception of smell (GO:0007608);;	--	Putative odorant receptor 85d GN=Or85d OS=Drosophila melanogaster (Fruit fly) PE=3 SV=1	olfactory receptor 35 [Bombyx mori]
c30980.graph_c0	--	--	--	--	Putative odorant receptor 85d GN=Or85d OS=Drosophila melanogaster (Fruit fly) PE=3 SV=1	odorant receptor 50, partial [Manduca sexta]
c32663.graph_c0	--	--	--	--	Putative odorant receptor 7a GN=Or7a OS=Drosophila melanogaster (Fruit fly) PE=3 SV=1	olfactory receptor 20 [Bombyx mori]

c32039.graph_c0	--	--	Biological Process: signal transduction (GO:0007165);;	K08471|4e-29|tca:663463|similar to Putative odorant receptor 94a	Odorant receptor 2a GN=Or2a OS=Drosophila melanogaster (Fruit fly) PE=2 SV=2	olfactory receptor 59 [Bombyx mori]
c30358.graph_c0	--	--	Molecular Function: olfactory receptor activity (GO:0004984);; Molecular Function: odorant binding (GO:0005549);; Cellular Component: membrane (GO:0016020);; Biological Process: detection of chemical stimulus involved in sensory perception of smell (GO:0050911);;	--	Putative odorant receptor 19b GN=Or19b OS=Drosophila melanogaster (Fruit fly) PE=3 SV=1	olfactory receptor 54 [Bombyx mori]
c35167.graph_c0	--	--	Biological Process: single-organism process (GO:0044699);; Biological Process: response to stimulus (GO:0050896);;	K08471|3e-17|nvi:100328552|Or92, NvOr92	Putative odorant receptor 94a GN=Or94a OS=Drosophila melanogaster (Fruit fly) PE=3 SV=1	olfactory receptor 64 [Bombyx mori]
c29815.graph_c0	--	--	Molecular Function: olfactory receptor activity (GO:0004984);; Molecular Function: odorant binding (GO:0005549);; Cellular Component: membrane (GO:0016020);; Biological Process: detection of chemical stimulus involved in sensory perception of smell (GO:0050911);;	--	Putative odorant receptor 85c GN=Or85c OS=Drosophila melanogaster (Fruit fly) PE=3 SV=2	putative odorant receptor OR12 [Cydia pomonella]

c34345.graph_c0	--	--	Biological Process: single-organism process (GO:0044699);; Biological Process: response to stimulus (GO:0050896);;	K08471|3e-36|tca:661659|odorant receptor	Odorant receptor Or1 GN=OR1 OS=Anopheles gambiae (African malaria mosquito) PE=2 SV=1	putative odorant receptor OR3 [Manduca sexta]
c34297.graph_c0	--	--	Biological Process: single-organism process (GO:0044699);; Biological Process: response to stimulus (GO:0050896);;	K08471|8e-20|nvi:100328547|Or82, NV17546, NvOr82	Putative odorant receptor 92a GN=Or92a OS=Drosophila melanogaster (Fruit fly) PE=3 SV=3	putative odorant receptor OR31 [Cydia pomonella]
c37409.graph_c0	--	--	--	--	Putative odorant receptor 92a GN=Or92a OS=Drosophila melanogaster (Fruit fly) PE=3 SV=3	PREDICTED: putative odorant receptor 85c-like [Bombyx mori]
c33544.graph_c0	--	--	--	--	Putative odorant receptor 13a GN=Or13a OS=Drosophila melanogaster (Fruit fly) PE=3 SV=2	odorant receptor 6 [Plutella xylostella]
c36203.graph_c0	--	--	--	K08471|2e-13|dvi:Dvir_GJ14423|GJ14423 gene product from transcript GJ14423-RA	Putative odorant receptor 30a GN=Or30a OS=Drosophila melanogaster (Fruit fly) PE=3 SV=4	olfactory receptor 20 [Helicoverpa assulta]
c35759.graph_c0	--	--	--	--	Putative odorant receptor 13a GN=Or13a OS=Drosophila melanogaster (Fruit fly) PE=3 SV=2	odorant receptor 6 [Ostrinia nubilalis]

c32271.graph_c0	[RTKL]	--	Molecular Function: acyl-CoA dehydrogenase activity (GO:0003995);; Molecular Function: acyl-CoA oxidase activity (GO:0003997);; Molecular Function: protein serine/threonine kinase activity (GO:0004674);; Molecular Function: ionotropic glutamate receptor activity (GO:0004970);; Molecular Function: extracellular-glutamate-gated ion channel activity (GO:0005234);; Molecular Function: ATP binding (GO:0005524);; Cellular Component: peroxisome (GO:0005777);; Biological Process: protein phosphorylation (GO:0006468);; Biological Process: fatty acid beta-oxidation (GO:0006635);; Biological Process: ion transport (GO:0006811);; Cellular Component: integral component of membrane (GO:0016021);; Biological Process: ionotropic glutamate receptor signaling pathway (GO:0035235);; Molecular Function: flavin adenine dinucleotide binding (GO:0050660);;	--	Aurora kinase GN=aur OS=Asterina pectinifera (Starfish) PE=1 SV=1	putative chemosensory ionotropic receptor IR68a [Spodoptera littoralis]

Supplementary Table 2.

>CpunOBP1

ATGATGAAGTTAAATATTTTCGTGAGCGTCGCCGTTCTAGCGGCAGTTTTGGGCAATGCCAGGGC
CTTAACCAAAGAAGAGTTGGGAGTCATCGAGAGTGACATGATCGCTCATGTGAAGAAGTGTGGC
GAGCAGTTCGGGGTCTCTGATGAGGAAATCAAGGCAGCCAAGGAGAAGAAGGACATCGACGGC
ATCGACCCCTGCCTGATCGGATGCGTGTTCAAGAGCACCAAGCTGATCAATGATGAGGGCGTGTT
CGACCCTAAAGTAGCACTGGAACACAGCGAGAAATACCTCAGCAGCGACGATGACAAGGCCAA
GTTCAAGGACATCGCTGACGATTGTGCTAAAGTGAATGACGAAAGTGTGTCCGATGGGAAGGAG
GGCTGCGAGCGAGCGAAGTTGCTTCTGTCCTGCTTCGCGAAACACAAGGATGAACTGCGTCCAT
CTCGTCGT

>CpunOBP2

ATGCTCCTTGTGTTGATAGCTAAGTTCTTGATGTTGTTGGCAACTTGCGAAACGATGACAATGAAA
CAAATAAGAAATACCGGCAAAATGATGAGGAAATCCTGCCAGCCGAAAAACAACGCTACTGACG
AACAACTCGATCCCCTAAATGAAGGCGTATTTATCGATGAAAAAGAAGTCAAGTGCTATATGGCTT
GCATCATGAGGATGGCCAACACGATGAAGAACGGGAAACCGAATTACGACGCGGCCGTCAAACA
AGCTGACTTGTTACTGCCCGAAGAGATGAAACAACCTGCCAAAGAAGCTTTATTTGCCTGCAAG
AAAGTTCCGGATGACTACAAGGACCCGTGTGACGCGGCCTTCCACGTCACCAAGTGCATCTTCA
ACCACAATCCAAGCATTTTCTTCTTTCCA

>CpunOBP3

ATGTTCGATTTGAGTTTTGCGATGACCAGGCAACAGCTTAAAAATTCCGGTAAACTTATGAAAAA
GTCGTGTATGCCAAAGAATGACGTCACCGAAGAGCAAATCGGAGATATATCGTCAGGAAAATTCA
TTGAGGAGCGCAATGTTATGTGCTACATCGCCTGTGTTTATACAATGACCCAAGTTGTAAAGAATA
ATAAAATAAGCTATGACGCTGTTATCAAGCAAGTGGACATGATGTTCCCACCGGAGATAAAGGAT
AACGTCAAAGCTGCGGCCGCACACTGCAAGGATGTTTCCAAGAAGTACAAAGACTTATGTGAGG
CATCATATTGGACAGCAAAATGTATGTACGACTTTGATTCCGAAAGCTTTATTTTCCCA

>CpunOBP4

ATGTTTAAGTCTGGAGTATGTTTCGTTTTCATTGTCAGCTTATTGGAATTTTCAATGTCGTTATCGG
ATGAAAAGAAAGCGGAAATCCTAGCAAAGTTTATAAAGGTCGGAGAAAAATGTATTATTGACTAT
CCGCTAACGAAGGAAGAAATTGCTGCGTTTAAGGAGGGCAAGTTCCCGGATAGCAGGGGAGCCG
CTTGCTTCAGCGCTTGCATCCTGACCAAAATTGGACTCATGGACGATAAAGGAGAAATATCAATC
ACCGCCGCATTAGAGCGTGCTAAAACCATATTCAAGGACGAAGAAGAACTTAAAATTGTTGAGG
ACTTCTTAAATACTTGCGCTAAAGATGGTGGAACCAAAGGAGAGGACAAGTGTGACCGAGCCAA
AGAAATATTTATATGCTTCATAAAAAAATCTAAGAAGTTCGATCTC

>CpunOBP5

ATGATTTCCAACTACAAATACATTCAGTGGTTCGGACTGTTCGGTACTTGTAGTTGGAAATCATTG
AAAATGATCCGTGCGGTGACATTGTTCTGTGGCCTTTTCCTTATGGCGTTGACCCCAAATGTGGAT
GCAATGACAGAGGAACAAAGAGCCAAGATCCGGGAACACTTTGAGACTGTTGGAATGCAGTGTA
TTGGTGACAACCCTCTCTCCGAGGAGGATATCACCGCTCTTCGAAGCAAAAAGGCTCCGTCCGA
CTCTGCTTCCTGCTTTCTGGCCTGTATGATGAAAAATGTTGGCGTGTTGGATGACAGCGGTATGCT

GCAAAAAGAAACGGCGTTGGAGCTCGCCCGCAAAGTCTTCCAAGATGAAGAGGAGTTACAGAT
AATTTCAGATTACTTACATTCTTGCTCTCCAGTCAACTCGGCTGCAGTCAGCGACGGTGCTAAGG
GCTGCGAGCGAGCCATGCTGGCCTACAAGTGTATGATTGAGAACGCATCTAAGTTTGGCATCGAC
GTC

>CpunOBP6

ATGTCCTGGACGGGTCTGGCCATAATAGTCACAGTTTTAACTGTTTGTCACGGAAAAGATACTTTG
GAACTGAGCGATGAAATCAAAGAAATAATACAGCATGTTCACAACGAGTGCGTCGGTAAGACCG
GCGTCGCCGAGGAGGATATAAGGAATTGCGAAAACGGCATATTTAAGGACGACAAGAAATTGAA
GTGCTATATGTTTTGCCTGATGGAGGAAGCGAACCTCGTGGACGATGATGACAACGTGGATTACG
ATATGTTAGTCAGTATAATCCCAGAGGAGTACACAGATAGGACAACTAAGATGATATTCTCCTGTA
GACATCTTGATACACCTGATAAAGATAAATGCCAGAGGGCTTTTGATGTCCACAAATGCTCATATG
GAAAGGACCCGGAATTCTACTTTTTGTTC

>CpunOBP7

ATGATTCATGCTGTGATTTTGTTTTGTGGTCTTCTCTTTGTGACATGGATGCCTTATTCTGATGCAG
TCACACCGGAACAAAGGGCCAAGATTGGACTGCACTTCCACGCGGTAGGGATGGAGTGTATCGT
CGACAATCCCCTCGCTGAAGAGGACGTGCTCGCCTTCCGGAACAGGATGCCCCCTCCCGGGCCC
AACGGGGCCTGCTTCCTGGCTTGCGTCTTGAGACGCGTTGGTGCTATGGATGATGCCGGTATGAT
GCAAAGAGATTCGCTGCTAGAACTCGCTCGTACAGTCTTCCACGATGAGGCCGAGTTACGGTCTA
TTTCAGATCTCTTACTCTCATGCTCGTCAGTGAACTCCATTGAGGTGAGCGACGGGGAGAAAGGA
TGCGAGCGAGCTATGTTGGCTCTGAAATGTATGCTCGAGCAGGCATCTAAGGCAAACTCCCTAGA
TGAAAAGAACATGATTCGAGATAAATTTGAGTCTGTCGGGGAGGAGTGTATTGAGAAGCATCCGC
TCAGTGATGAGGACATCGCTGCCCTCGAAAACAAGATGCCTCCTCCGGGACGCGTCGGCGCCTG
CTTCGTGGCTTGCGTTATGAAAAACGTTGGAGTGATGGATGACGCAGGTATGCTGCAAAAAGAG
ACTGCTCTAGAACTCGCTCGCGAAGCCTTCGACGATGAAGAGGAGTTAGAGTCGATTGCAGACT
TCTTACACGAGTGCTCATCATCAATAAACTCCGTTGCAGTGAGCGATGGCACGGAAGGCTGCGAC
CGGGCGATATTGGCTCTGAAATGTATGAACGAGCACGAATCTAAGTTCGGCCTCGATCTC

>CpunOBP8

ATGGATGGACGGATTTGTCTTCTATTGGTGTTTCTTGTTGGTGGTAGTGATGCGATGACCAGAGCA
CAATTAAAAAATTCAGCGAAAATGCTGAAAAAGAATTGCATGGCGAAAAATTCAGTTACTGAAG
ATCAAATAGGAAATATCGAGAAAGGCCAATTCATAGAAGAAAAACCTGTCATGTGCTACATAGCT
TGTATTTATCAGATGATGTCTATCGTGAAGAATAACAAATTGAACTACGAAGCATCGATTAAGCAG
GTGGACATGATGTACCCGAACGATCTAAAGGAGTCTGTCAAGAAATCTATCGAAAACTGCAAGTC
TGTTTCCGACAAGTACAAGGACATATGCGAGGCTTCGTACTGGACAGCCAAATGTATTTATGAAG
ATAATCCGAAAGACTTTATTTTTGCT

>CpunGOBP1

ATGATGAACTCCGCCAGCAGCATCTCCATGGAGGGCGCGAGGCTGCGCTGCAGGCACGAGTCGC
GGATGCACTCGCCGATGCGCAGCGTGCGCATGCAGTGGTCCTCCACCTCCTCGAACTGCTTCTCG
CACTCGTGGATCAGGGAGACCAGCTTGGAGGCCAGCACCTCGCCGTTGGGGAAGCTCCTGATGA
ACTTGTCGGTGTTCTCGTGGTGCAGCTTGTTGGCGTCGGTGATCAGGTTGAAGTACTTGCTCATG
CAGTGCAGCGCGCAGCCCAGCTCGCGGTCCACGAACTTGAACTCCTGGTGCCAGATGTGGAAGA

ACTCCTCCATCTTGTCCTCCGTCAGCCCCGACTCCTCCCTGCAGTGCTCCAGCGCCTGGCCGAAG
CCCAGCGTGACGTCGGTCATGATCTTGTGGTCGCCGGCCACCAGCCCGGCCGCGGCCACCACGC
ACGCCGCCACAACCAACCTCGCGTCCGAGACCATCGTGCACTATCCGCTCACCCGCCAAGCTCA
CCTTCCC

>CpunGOBP2

ATGCTGCCCATTTGGTTGTACTTCGGTTTGGTGATGGCCGCGGTGTCGTCGGTGAAGAGCACTGC
TGAGGTCATGAGCCACGTGACTGCTCACTTTGGAAAAGCATTGGACGAATGCAGGGATGAGTCC
GGGCTCTCTCCTGAAATTCTGGAAGAGTTCAAACATTTCTGGAGCGAAGACTTCGAGGTGGTGC
ACCGCGAGCTCGGCTGCGCGCTCATCTGCATGTCCAACAAGTTCTCTCTGCTGCAGGAGGACACC
AGGATCCATCACATCAATATGCACGACTATGTCAAGAGTTTTCCTAATGGCGAGGTTCTCTCAGCA
AAAATGGTGGAACTACTTCACAACTGTGAGAAGCAGTATGACGCCATCACTGACGACTGCGACC
GAACTGTGAAGGTGGCTGCTTGCTTCAAGAATGACTGCAAGAAGGAGGGCATCGCTCCGGAGAT
CACCATGATCGAGGCTGTCATGGAGAGATAC

>CpunPBP1

ATGGGTATGCTGGTCAAGTTACTTTTGGTGATAGTGGCAAGTGTGGGGGTAGAATGCTCGCAAGA
TATACTGAAGAAAATGACTGTGAATTTCGGAAAAGCATTAGAAGCTTGCAAGAAAGAGATAGAC
CTTCCGGATTCTGTGAACGCGGAACTGTACAACTTCTGGAAGGAGGATTACCAGCTGACGAACC
GGCAGGCGGGCTGCGCGCTGGTCTGCATGTCCACCAAGCTGGACCTGGTGGACCCCGACGGGAA
CATGCATCACGGCAACGCGCACGAGTATGCTAAGAAGCATGGAGCTGATGACGCCACAGCCAAA
CAGTTGGTAGAGATGCTCCACACCTGCGAGAAGTCGGTGGGCAAAATGGACGACAACTGCGAG
AGGGCGCTCGCCATCGCCCGCTGCTTCAAGGCCGAGATACACAAGCTCAAGTGGGCGCCCGACC
CGGAGGTCGTGCTGGCAGAGATCTTGGCTGAAGTT

>CpunPBP2

ATGGCCTTTAAAATGTGGTTGAAAAATATAATGGTCGTGGCAACGGTCGTGATGATGAGCGTGAA
GGTAGATTCATCACAAACGATGATGAAGGATATGACGAAGAATTTTCTGAAGGCGTATGGTGAAT
GCCAGCAAGAGCTTCATCTTACCGATGATACTGCTAGAGATTTGATGTTCTTCTGGAAGGAAGACT
ATGAGGTCACCAGCCGGGAGGCCGGCTGCACCATCTTGTGTCTCTCCAAAAAACTGGAAATCATC
GACCCTGAGGGGAAACTGCATAAAGGGAAAACGGCGGACTTCATCAAGCAACATGGAAGTGAT
GAGGAGACAGCTCAAAAGGTGATAGACGTTCTGCACGCTTGCGAAGCCAGCGCTGTTCCCAATG
AGGACCACTGCATAATGGCTCTGGGCGTGGCCACCTGCTTCAAGAAGGAGATACACAAGCTGAA
CTGGGCTCCGGACACTGAAGTCTTGTTGGAAGAGCTCATGGCTGAGATGTCAGAACGA

>CpunPBP3

ATGTCGAGAGTTGTATCAGTGACTCTGTTAGCTTTGTTAGCCCTCTCCGTTGGGCTGTCAGTGGGA
GCTTCTACGGGTACCAGCGGGTCGTCAGCTACCACTAGTGGGACTACGAGCAATGACCTGTCAAG
ATCCACAGACGATGTGAAGGGTAAACTGATCACTCCAGCTGACGACCAGGGCAACATGACCTCC
TCGGAGGAGAGAGCCCTGGATGTACCGGACCTGATGGCTGTCATGGTCGAATGCAACGACTCCTT
CAGGATCGAGATGGGTTACTTGGAGTCATTGAATGAAAGTGGAAGTTTTCCTGATGAGATCGATA
GAACACCAAAGTGCTACGTCCGATGCGTCCTCGAGAAGACAGGGGTGGCGTCAGAAGACGGTC
TGTTCGACCCTGCGCAGGCGGCTGCGGTGTTCGCCGGGGAGAGGAACGGCGTGCTCATGACCAA
CCTGGAGGACTTGGCATCCAGGTGTGCAGCTGACCGAAATGAAAAATGCAAATGCGAACGGTCC

TATAACTTCATCAAATGCCTGATGGAAGCCGAGATAAAGGAATATGTATCTAAT

>CpunPBP4

ATGAAAGGGTTCTTTGTTACTCTTTTGGTGGTTTTCATGGGAGGAAAGGAGGTAGAGATGTCGTC
TGATGGAATGAAGCAGTTGACTACTGGCTTTTTGAAAGTTCTTGGTGCTTGCAAAACTGAGCTCG
GTCTATCAGACGGCATCCTGAGCGACATGTACCACCTGTGGAAGGAAGAGTACGAGCAGGTGAG
CCGCGACTCCGGCTGCATGTTCAGCTGCATGAGCAAGAAGCTGGACATCCTGGACGGGGACGGG
AAGATCCACCATGACCACACCAAGGAATACGTGTTGAGCAACGGCGGCGGTGAAGACCTAGCAC
GCCAGCTCATAAACGTAGCTCACGACTGTGAGAAGCAACAAGAATCTCTGGAGGATGAATGTGA
CCGAATGCTGGAGATAGCCAAGTGTCTCCGGAGGAACATCAAGGAGATACAGTGGACGCCCAAA
GTGGAGGTCATCATCACCGAAATTGTGGCTGACATG

>CpunABP

ATGTTTCTAGGACATTTTTATATTTCGTGTCTTCTTTTAATTATTTTCGACACTTATCATGTTATGTCG
ATGACCAGACAGCAAATGAAGAATTCTGGCAAGATTCTGAAAAAGACATGCATGCCGAAAAATG
ACGTCACAGAAGATGAAGTGGGAAATATAGAAAAAGGAAAATTTATAGAGACGAAAAATGTCAT
GTGTTATATTGCTTGTATATATTCTATGGGACAAGTCATAAAAAACAACAAAATAGCTCACGACGC
CATGTTCAAGCAAATTGACATGGTGTTCCCACCAGAAATGAAAGAGCCGGTCAAGGCAGCTGTA
GAGAAGTGCAAACCAGTTGCAAAGAAATATAAAGACATCTGTGAGGCCGCCTACTGGACTGCCA
AATGCATATACGACGCAGACCCGGAGAACTTTGTATTTGCT

>CpunOR1

ATGAGAGCTGGTGGTTGGTGTATTCATTCGAAGCATCTCAGCGCCAATATGACGCGCCCATTTCAT
CTAACTAACAAAAGCACGAGATATTTCTATAAAATGTGCAATATCGTCTATCTTACAGGGTTCCCA
AACTTCTGGACGGAAGATCTGGGTTACTCTGAAACATTCGTCAAATATTATAAATATTTTACGAAA
CTCGTTACCAGTATTCTCTATGTATTCATAGTTACTGAGTGGGCCGCCTTCTTCACGCAACACAATA
TGACACCAAAACAGAGTTCCGACAGCATACTCTTCAACTACTCCCATCCATTGCTGACCTCGTAC
AGACCGATCATGATTTACCACCAGGACAAGTTCCGCGAACTGATATTCAACCTGTGTGTGAAACT
GAAAGAGGTCTTCAACGACGAAAAGATGGAGCAGGAAATGATCAAGAAGTCGAAAATGTGCGT
GGCTAGTTTTACGACTATATGCAGTATGTCACTCATATTTTATGGTTTTGAAGGAATTTACCAGGTT
TTGATGTCAGATGCCACATTCACCACGGTA

>CpunOR2

ATGGACGCCTCCGAGAATCTGAAAACTAATCACCCTCAGAAATATTTCCTAAAACTGATATGTAGA
TCCCTTTACTACATAGGGATGGGAGACTATTGGTATGAGAAGACAGAGAGAACCAAAGTCCATAA
GAAAATGTATATCGTTTACGCTGTTCTTATAAACGGGTATCTCTTATTGTCCACTCTGAATCAGCTC
CTAGCCCATTTTAGGACAGACCTGACTTTGAAGGAGAAGAACGATTTAATTCAATTCTCCTTGGC
CCACCCATCCCTTTGCGCCAAGTTCATCTGCTTGTTTCTCCAGAAAGATCGGCTTAAAGTGCTATT
TGAGAAACTGATTGAAGGGGATTCGTATACGTTTAGATCTTTGGACGTGGAAAAGGTATCTGTTA
AAAAGGCGATTCGGTATTTTGTGGCGTTGTCCACCGGAACGTACGCGGCTGTGCTGTTTTCGACG
ATAGATGGATTTTTATCTTCTAAAAGAGACGGTACTCCATTCCAAACAGAAGTGTCTCTAATCCCA
ACAGGATCTCAACTTGGTCCTTTCTGGTCCTTTATTAGATGCCTTCACGTATTGCACTGGTGGTGTA
TTGTCACGAACATGTTATTGGTAGACGCGATCTCTTTTACCTCACTGATCTTCCTTGGATATAAGTT
TAGACTGGCTTGCATGTACTTCGAACAGTTGAGGGTTAAAACTAAGGACAACTGCAAGAGTAAG

AGTGACAAAGACTGTATGGAGGAATATGAGAAGGGTTTAATTACTGGGATAAAGCTGCACCAGGA
TGCTTTATGGTGCGCACGCACCGTGCAGTCCTCTCTAGGAATTATATATGGGGTTCAAATTATTGAA
ACCACTTCAATACTTGTTATGTGCATGATAAAACTTGTTGCTACTAGAGGGAGTTTCACGTTTCTC
TTGGCCAACTTCTTTTACATAGCTTGCCTTTTGATACTGAACGGTGTGTATATGATAGCAGCGGGA
GATATAACATTTGAAGCTTCGCCTCTCTCCACCGAAATCTTCCACTCCGGGTGGGATCTGTTGAAA
AGTAGGAGGAGGTTCCGTACCCTGATTGTGCTGGCCATAGCCTGCAGCCAGAAGCCGGTCTACAT
GACCGCATTTGGTGTCATCACGTTGTCTCATGAGAACTTCATCTCGGTGCTGCGTTCATCATACTC
ATTCTTCGCAGTGACATAT

>CpunOR3

ATGCACGCGTTGGGCAAGTCTATATACTACGGGTTTTCTGGGGCTTCCTTTGTATGGATACTTGTTT
TTATATTTGAACGAATCGAAGACCCTCTAAACGCTTCAGTGCCATCCTATGTACCTTTTGATACTAC
ATCGACGGCTGGTTACTCACTGTCCGTTTTCCTTGAAGTGGTGCCAATTTTTTGGGCAGGTTATGG
ACATCTTGCAGCGGATTGCGTTGTAGCGTGCTACTACTCTCAAGCTAGAACACAATTAAAGATCAT
TAAGTACAATTTGGAACATATGTTTGACAATGAAGATAAAGAAAATGTTCTTATAGAATGTGACAA
CATCTCGAACGGTCGATTTAAATACTTAGACGAAGTCAATAGCAATATAAGAACAAAGTTCATTTA
TTTCATTGAGAGATATAAGAAG

CpunOR4:

ATGGAACCTACTCAATCGCAAGTTTTTGATCTAAACATTTTTCTATGGAAAATTTTCGGAATGTGG
CATCACGAAACTTCTACAATTTACTACAAATATTACTGTGTTACTTTTCAGATGTTATATTCAGTTAC
ATATACGTTTCTTTTTACCCTAAGTTTGATGTTCACACCGAATGATCTCGAACTTATTATATCACAAT
GTATGTTCTATTTTACGCAACTGGCAGGTGTTACTAAAATAGCAATGATCATACTACGAAAAAAGT
ATATATTTGAAGCTTTCCACATATTGGACTGCATAGAATTTCAAGGAGATGACGTGGAAACCAGAA
AAATTGTTAACGAAAATAAAATTTTCTATAAGAAGTACTGGAAAGCTTGTTTTGTGTGTTATAACT
CTGGTGGTTTTTTTATCTTATGTTTACCACTTATCAATTACGTTGTCAGAAGAACTGAGTTGGATCT
GCCACTTTGTCAATATTATTTTTTAAGCGATCACACCCGTGAAAAATATCATAGCGGTTTGTTTTTT
TATCAGTTTACTGGATTAGTGGTGATCATTTTAAGTAATATCAGCACAGATACTTTCATCATTGGGTT
ATTAATGATGGCAATAACACAGTTTAGAGTTTTGAATTGGAAATTAACGAATTTAAATTTTTCATCA
CTGGACGAAGAACTCGTAACAATGGTCGACAAAGAAGTTCTATTTGTAGAGAAACTGAAAAAAT
GTCTCGTACATTACGACCTTCTTCAGAAGTATTGTAACATCATACAAGACGTGACGAGTTATTCAA
TATTTGCCCAGTTTGGAACAGCAGCAGTTACTTTATGTGTATCCATGTGTACTTTTCTCAGGCCGAT
GACGAATAACGATTTTATGACTTCCGTGACTTACATGACTTCTATGGCTATTGAAGTATTCTTGCCG
GCATATTATGGAGCGGAATTAACTCATGAGAGTGAAAAGATTGTCATGGCTGCATATAGCAGCGAT
TGGATACCACGTTGGGAAAGTTTTAAGGGCAGCTTGCTTCTGGTCATGGAGCGTGCCAAAAGGC
CCGTTGTGATCACCGGCCTTAAGATGTTCACACTCTCACTGGAAACCTTCGCTGCGATAATCAAG
ATGGCATATTCCATCTTTACACTTCTGAAAAATGCACAGAAT

>CpunOR5

ATGAACCTAGTACAAAGGAAAAGTAACTACCAAGCAAATTCTTTTAATGGAGCATACAACAGGAA
AGAAAGCGATTTAATAAGAAAACAACTCATAAAATGCATTGATCATCATAATACTATTGTCAGTTTT
GCAGGTTGTGTGTCAGAGTTATTTGGCCCCGTTCTTGCTTTTTCCTATCTAAACCATCTAGTCTGCG
GCGCTCTCATGCTGTTGGAGTGCACAATGGGGGACAGTGATACCGTCATGCGTTGTTTACCTATTA
CCATAATTACATTCAGTCAGCTTGCTCAAATATCTGTGACGTTTGAAATCGTAGGATCTGAGAGCG

AGAAATTGATAGATGCAGTGTATAACACACCGTGGGAATGTATGAACACTAGTAATCGAAGGCTA
GTGTCCATGTTTCTGGCGAAGGTACAAACCCCAATACGCGTCACAGCCCTAGGGATGGTAGACGT
GGGCGTGGAAGCTATGGCTGCGATTTTAAAAACAACATTT

>CpunOR6

ATGGTAGCGTATGTGCTCTATCTATCACTTCATAAAGTCCAGATCTTGCGAACCGCTGAGGGCAAG
TTCGAGGAAGCCGTGATTGAGTACCTGTTTACGGTATATTTGTTCCCAATGGTCGTTGTTCCAATAA
TGTGGTATGAGGCCCGCAAAATAGCTGGAGTGCTCAACGAATGGGTCCATTTTGAGACGGCGTAC
AAGCAATTATCTGGAAAAGCTCTACCAATTGGCTTGTACAAGAAAGCCCTAACCCTCGCCGTCGT
AATCCCCTTACTGTCAACG

>CpunOR7

ATGACGTCGCTGGCGGGCGGCAGCGTGGCGCCGCACCTGCGCGTGCTGCGGCTGGTGGGCTTCT
GCCGGCTGCGGGCGCCCGCGGGCCGCGCCGCCCCGGGCCTGGCGCGCCGCCTGCACGGGCTCTA
CTGCTGGTTTGCGCTCGCCGCCACCTCCTTTTACGTACTTCAGCAGGGAATTTATGCATATCAGGA
GCGGCACGACATGGTGAAGCTGTCGCGCGTGCTGTTCGTGCTGCTGTGCCACTGCACGTGCATCA
GCAAGCAGGTCGTGTTCCACGCGCACGCGCCGCAGATCGACGCGCTCATCCGCAGCCTCGACCA
GCCGCTGTTCAACCAGGGCGGGGCGGCGCGCGAGGCGCTGCTGCGGCGCGCGGCGTCGCGCGC
GGCCCGCGTGCTGCGCGCCTACTACGGCTGCGGCGTCGCCACCTGCGTGCTCTGGACCGCCTTCC
CCGTCATCTACCGCCTGCAAGGGCACTACGTTGAGATGCCCTTCTGGACCTTCATCGACTACCGTA
AGGCGTTCATGTTCGTGGTGGTGGTGAGCCACTCGTTCTACGCGACCAACCTGGTGGCGGTGGG
CAACACCACCATGGACGCGTTCATCGCCACCGTGCTGTACCAGTGCCAGACGCAGCTGCGCATCC
TGAGACTGAACTTCGAGTCTCTGCCAGAAAGAGCGAGGATCGCATCAAAGGAGTTATCCAAAGG
CGACTATGAGGCGACATTAAATAAATTACTGTTGGAGTGTATACTGCATTTTCAGAAAATTGCCGA
CACCATGGAGCTGCTGCAGCGCGTGTTCGGGCTGGCGGTGCTGGTGCAGTTCACGGTGGGCGGC
TGGATCCTGTGCATGGCGGCCTACAAGATCGTCAGCGTGGACATACTCAGTTTTGAGTTTGCGTC
GACGACGCTTTTCATCGTTTGTATTCTAACAGAGCTATTTCTCTATTGCTACTATGGGAATGAAGTT
ACGGTTGAGAGTGACCGCGTGGTGGAGTCGATCTACAGCATGGAGTGGCTGCACGCGCCGCTGC
GCTTCAAGAAGTCGCTGGTGCTGATGATGGAGCGCGCCAAGCGGCCGCTGCGCCCCGCCGCCGG
CCGCGTCATCCCGCTCTCGCTGGACACCTTCGTCACGATTCTAAAATCATCGTATAGTTTCTATGCT
GTCCTTCGACAAACAAAA

>CpunOR8

ATGGAGTTTAAACAAATTGATTGTTTTGATAGAAATAAAAAATTTTGGAAATTTCTTGGGTTGTAC
CCTGATATCGATATCTGGCCATATTATCATTATTACTCAATTTTTTTCATTTTTTTTGTAATCATATTGT
ATGATTTTTTGCTGTCTATTAATTTTTACTTTTTACCGAGACAACTGGACAGCTTCATTGAAGAAAT
GTTGTTTTACTTTATGGAGCTCGCTGTAACGTCAAAAGTGTTTACTTTTTTCTTTCAGCGAGGCAA
AATATCGAAAGCTCTAGCAAGCTTAGAAAGTGATATATTTCAACCAACCACTAATGAAGGAGTGG
AAATAATAAATAAAGCAAAGAAGTTTAATGTTAGATACTGGAAAATCGTAGCCGTGGTTTCGACA
ACATCAAATTTTACACATGTATTAACCCCATTTTTCGTACACCTATTTTTGCCTGTAAAATTGGAGTT
ACCAGTGTGTTCTTATTCCTTCCTTTCGGAAGATACCATTCAGAGATTTGTGTATCCTATATTTGTTT
ACCAAGGTATTGGAATGCATTTTCATATGTGGTGCAACGTGAACATTGATACTTTCTTTTTGGGCG
TCATGATATTTGCAATAGCTCAACTGGACATCCTTGATATCAAACTGAGAAGTTTAACAGATAATAT
TGCTGAAGATACAGACGTTGATAAGAATCTTAATCTTAAGCTCAATGAAGCAATCAATCATTATAG

TGAGGTGGTAAAGTTTTGCTATTTGATTCAGGATATTTTCAGTGTGACATTATTTGTGCAATTTAGT
ATGGCTTCATGCATAATATGTGTTTGCCTTTTCAGGTTTACTTTACCCGCAACAGCAGATTACTATG
TATTTCTGGCCACTTACATGTTTATAATGATAATACAAATTATGGTACCTTGCTGGTTTGGAACTCG
AATTATGGATAAAAGCTGCTTGTTGGCCTCAGCTATTTACAGCACCGATTGGACGCCAAGGTCAA
AACGTTTCAAGAGCAGTCTCCGAATCCTCGTGGAGAGGCTGAATCATCCAATATCAATTGTCGGT
GGGAAAATGTTCCCTCTCTCGTTAGAAACTTTCACTTCGATAATGAATTCTGCTTACTCATTCTTCA
CGCTACTTCGGCACATGCAAACGAGAGACAGA

>CpunOR9

ATGGTCGTGTTGTACGTCGGCTACAAGCGCGTCATGATCCTGCGCTCCATCCGCAAGTTCGACGA
CTACATCTACGCCATTATATTCGTCGTGTTCCTCGTTCCGCATTTCTGGATCCCTTTTGTTGGGTGG
GGAGTAGCCCATCAAGTCGCTATCTATAAGACTAGCTGGGGAAAGTTTCAAGTGAGATACTACAG
AGTTACCGGGGAAAATCTCCAGTTTCCTAACCTAAAATCCTTGATTGTAGTAATTAGCATTGGCTG
TTTACTTCTCGCTATAGGCTTCTTATTAAGTCTATGTGCACTCATGGACGGGTTTTTACTGAAGCAT
ACTGCGGCGTACTACCACATTATAACAATGATTAACATGAATTGTGCTTTGTGGTACATCAACTGC
AAGGGCATCAAAATTGCATCCCAAAGTCTTTCGGATTGCTTTCGCAGGGACGTGAGAAGAGAAT
GTTCTGCTAAATTGATTTCCCGTTACCGATATTTGTGGCTGAACCTGTCAGAACTACTTCAACTATT
AGGCAATGCGTATGCGCGGACATATTCCACTTATTGCCTTTTCATGTTCACCAACATAACAATCGC
AGTCTACGGCGCACTCTCAGAAATTGTCGACCACGGCTTTGCATTCAGCTTCAAGGAGATAGGCC
TATTCGTCGACACTGTCTACTGCTCCACTCTACTCTTCATCTTCGCTGACTGCTCTCATAAGTCTAC
GCAAAAGGTGGCAGATGGAGTACAGGAAACCTTACTGACGATAGATATTTTAGCCGTGGATAGAC
CGACTCAAAAGGAGATTGACCACTTCATTCAAGCCATAGAGATGAATCCCGCCGTGGTGAGCTTA
AAAGGATACGCCGACGTTAACAGAGAGTTGCTGACATCGGCCATAAGTATGATAGCTATTTATCTC
ATAGTGCTGCTGCAATTCAAGATCTCGTTGCCCAAGGATCCACAATCTCCGACGCCG

>CpunOR10

ATGTCGGATCATACAGAAAAACCAAACGGCATCAGTAGCTGTAACTTGAATCAAAATTTGGACAA
TGTTGAAGCAAAAGAAGGGATGCAAAAAGAAATTCTATTCGATCAATCACTACAAAAAATAAAAT
TTGCATTCCGTCTGACAGGCCTGAACATACAAAATGAAAAAAGATCAATGAAACAAAATTGTGTG
TACCTATTCAACTTCTTATGGCTAAATACAGACATAATTGGAGCCCTATTCTGGGTCATCGATGGTA
TGTTAAGTGGCAAAAACTTTACGGAACTAACCTATGTGGCCCCTTGTCTAACACTCAGCATCCTA
GGTGATGTAAAAGCTATATATCTAGTCCTAAACGAATCAAAAGTGCACACTTTGATTGCAAATTTG
CGTAAACTGGAGCAAAAAGCGAACGAATTCGAGAATTTTGAACAAGACAAATTGATAGAACCAG
ATATACGACTTTTCAATGTTGTAATTAAGGTTTTGAATGTGTTAAATTGTCTCATGATAGTCGTCTTC
GATCTGAGCCCTCTTATAATAATAGCAGTTAAGTATTTCACAACCGGAGAGTTGGAGCTTATGCTC
CCATTTTTGGACGTTTATCCGTTCGATTCGTTTGATCTTAGGTATTGGCCTTTTGCATATATTCATCA
GATATGGTCAGAATGTATCGTCCTACTGGACATTTGCGCCACCGACTACTTATTTTTCACCTGCTGC
ACCCACATCAGGATACAATTCAGGCTTCTTCAGCACCAGTATCAGGAGATCATTGAACCTGTCAA
CTTGTCGGCAGTCGGCACAATTGATAGGTCTCGAATCAAGACCAAGTTCAAAGACCTGGCTGTTT
GGCATCAGGAGATCATTAATTCAGCAAGTATGTTGGAAGGTGTATATGCTAAATCGACGTTATTCA
ACTTTATGACTAGTTCATTGGTCATTTGCCTCACAGGATTCAATGTTACGACTATAGACGACAAGG
CGTTTGTGATAACTTTCATTATATTCTTGTTCATGAGTATGCTGCAAATATTCTTCTTATGTTACTTTG
GCGATATATTAATGAGTTCGAGCATGGAGGTGACAAATGCAGTTTACAACAGCAGATGGTACCTA
AGCGACGTTGGTATGGGCAGAAACGTACTGCTAGTACAGACTAGGGCTCAAAAACCTTGCAAGG

TGACTGCTGCAGAGTTCGCTGATGTTAATCTTCGAGCTTTTATGAGGATATTAAGTACTGCGTGGT
CATACTTCGCCCTGTTGCAAACTGTCTACAGTTCAAGATCTAAA

>CpunOR11

ATGCAATCAGATTTGTCAAAAGAACAATATAAGAATAGCATGATGTTACAAATACGAGAGTTTGGG
CCTGCATACAGCGATTTACCAACAATGATGCAAAATGTAGCCAAGCTGCTGAGAGTACTAACGCT
TAATATTGACAACGAGTATAATAAACCAATCCCAATCCACTGCTACATTCTGACCATCGCGACAGC
CGCAGCTTACACGTACGTGTTTGCGATCTCTGCCGGATGGTTCGTTGTCAAGAGGTGTATCCCCAA
TCATGACGTCATAGGTGCCATCATCCTGTTCTCTCTCTCCATCTCCAGCGAAATCGGCAGCGCCAA
GTTCTTTTATCTATATCTGTACAGAGACATAGTTAGAAAAATAGTGCGGCAAACCCTCGAATTTGAT
GCAGGCATCGTTCCAGGAAGTAGATATTCTAAGAGTGTGTTGCAGACGATGAGGAAAGCCAAAG
TGCGCGCCATCGCTTTCTGGTTCATCATCATGGGGAACGGAGTCGTGTACGTTTTTAAATCCCTCT
TGACGCCTGGCAGACATTTTATGGACGACGTGTATACGATATTTGGCCTAGAACCAATGCAGAAAT
CACCCAACTACGAGATAGGCTACGCGCTCGTCTCTGCGGCCGTCTGGTTCCTGTGCTACGTACCG
GCCAACGTCACTGCCCTCCTCATCGTCGTGGCCGGCTACGTCGAGGCCCAGATGGTGGCCCTCAC
ACAAGAACTCCTGCATCTCTGGCCCGACGCCGTACAACACTACCAGGACGTACAATTTTCAACAT
TCACCAGCAGAACTAACACCAAAAGCCGTCAACGAATTCTGAACGATTTCATCAACATTAAACTG
CAGGACGTAATAAAAAGACATTCAAAGAACGTCCACCTTCTACAGATGCTGGAAAACGTGTTCA
AAGGTGCTATAGCGTTAGAATTCACGCTGCTCGCATTGGGTTTGGTAGCAGAACTTTTGGGTGGT
TTGCAGAATACATACCTGGAGATTCCTTACGCGTTCGTTCAGGTAGCGATGGATTGTTGGACTGGG
CAAAGGGTGATTGATGCTAGCACTTCCTTTGAAGCGGCGGTGTACGCTTCAAACTGGGAGCACTT
TGATGTGGCCAACATGAAGATTGTGCTGCTGATGCTGGGCAGCGCTCAGAAGACCATGACGCTGT
CTGCGGGAGGAGTGACCATGCTCAGTTTCGCGAGCCTAATGAGTGTTGTGAAGTCGATTTATTCA
GCTTATACTGCGCTGCAGTCTACAATAAAG

>CpunOR12

ATGGACGTACCCACTTTTGAGGAACTCTTTCGACAGTTAAAATTCAACATGTGGTTATTTGGAATA
TTGTTCGACGACTACGAAATTGCATTCCGATTCTATTGTTTTGTCCTTTCTATGTTTTCCATGTTAGT
CGAGGAGACGTCATTTTTCATTTCAAGATACGCCCCGGAGAATTTTCTTGAGCTCACCCAATTAGC
CCCTTGTCTAGGCATCGCACTACTGTCCGTTCTCAAAATTCTCCCAATCGCAGCTAAACGACAGA
AGATATCCGTATTAACAAAGTCTCTCAATAAATTATATAATGCTATATTAACGGACCCCGAAAAGAG
AGAAGTGGTTAAAAAAGAAATTCTACTCGTCCATACCATCATTAAATACTTTTTCATACTAAATACG
ATTCTTATTACGGTTTACAACTTTGCACCGTTAGCGTTCATGCTATATGCATATCTAAAGCATAACA
GAATAGAGTACAAAGTGCCGTATGCTGTAAGTCTGCCATTTGCCTTAGACTCTTATCCAAAGTGGT
TCATAGTATATTTCCATTCTATTATTAGCGGGTTCGTGTGCATATTGTTCGTCACTTCAGTAGATGCT
CTTTACTGTATGTTGACTTCGCAAATTTGCAGCAATTTTACAGTTATTAGCAACGAGATACTACATC
TTGATGAATCTGGTGTCCATCGATTGAAAGATTTAATCATTTATCATCAGCAAGTTCTTAAACTATC
CGATGACCTAGAAGAGATATTCAAATTGGTCAATCTATTTAACGTCGTTGTGGGTTCCACTGAAAT
ATGCGCCCTCGGGTTTAACATAACGACTGGAGATCTTGGCCATTTACCAGGATACATCTTATTTTTA
TCTTCGGTACTGCTCCAGATATTATTAATGAGCGTATTTGGAGAAAAACTTATACGTGAGAGTACA
AAAGTAGGCGATGCAGCTTTTAACTGCAAATGGTATGATATGGATTTGAAATACAAGAAGATGATA
GTCTTTATTATAGCCAGATCACAAAAGCAGCAACAATTGACTGCCTACAAATTTTCAGTTATTTGC
CATAGGAGTTTTACAAAAATAATAAGCACATCATGGTCTTATTTCACAATACTCAGGACTGTATACA
CTGCTCCTCCGGAAACAGTGCAATTGGAG

>CpunOR13

ATGCTTTCGATAAAAAAGAAAATAATTTCTTGGCTTAAAAAGCTTGAACACCCAAAACACCCACT
GCTTGGCCCAAACATAAAAATTCTTTATTTATTCGGGATCTGGCAAACGCCTCGTACTAAAATCAG
GAATAGATTATACAATATCGTTCATGTTAGCACCTTCTTCTATGTTTTATCGCAATTCATTGATCTTTA
TAAGCAGCGAAATGATTTCAATAAGGCATTGAACAATCTTTCATTAACCAGCATTGGCGTAATTTG
CTCTGCAAAGTGCATCGCATATGTGCTTTGTCAGCCTCAGTGGCAAAAACTCGTAGCGAAAATAT
CTGAAGAAGAATTAACACAAATGAAGAAAAGAGATGTCAAAGTAGTTAAAAAGATGGAAGAATA
TAAGTTATATGCAAGAGTAGTATCTTACATGTTCTGGGTGTTAGTGTTGTTAACAAATATAACTTTG
ATCGGGACGCCGTTGTTAAAACTGCTCATATCGTCGACGTTTAGAGAAAATATTCACAAAGGTTTA
GAAGAATACCCGCAGATAATGAGTTGTTGGTTTCCCTTTGACTACATGAAAATGCCTGGTTATGCG
TACTCTTGTGTGATTCAAATAGTCATGGCTTTGCAAGGTTCTGGCGTGCTTGCTGGTCACGATGCT
AACGCTATAACCATAATGACGTTTATGAAGGGACAGATGCAAATACTCAGGGAGAAGTGCGTGAA
AATATTTGACTCAGACGAAAATGAGGGTCCGAATGAATTCTTCATTAGGATGAAAGAATGTCATAG
ACATCATAATTTTTTGCTCGAGCAGTCAAACCTTTTTAACTCGCTCTTGTCTCCGGTTATGTTCGTC
TACATACTGATCTGTTCAATGGCGATTTGCTGCAGTGTTGTACAATTTTCGTCAGAAGAGGCTACA
TCTGAACAAAAATTATGGGCAGTTCAGTACACTTTGGCACAGATTGCACAATTATTCCTGTTCTGT
TGGCACGGCAATGAAGTTTTTGTTGAGAGCAAAGATGTAGACATGGGAGTATACTCTAGTGATTG
GTGGAAAGCAGACGCGCGTTTGCGAAGACATGTGGTCCTTCTAGCAGGAAAGCTCAACCGTCCC
ATATTGTACAGTGCTGGACCATTCTCCGATCTGACTTTGCCTACCTTCGTCAGTATCATGAAAGGC
TCTTACAGTTTCTTCACTCTATTTTCACAAATGCAAGAGGATAAT

>CpunOR14

ATGCGGTTCAATTTCCTCCGCGCAGCCGCGGAACCCAAAGAGACATCCACATTGGACGGTGACC
CCATCAGAAATTACATACACTTCTTAGAGACGCCTCTGAAGATCGTAGCCTGCTGGGATTGGTACA
GACGCCCGCAAACAGACCGCGAAATAATCATTAACAATGTGTACCTTTGTTTGGTGCTCTTCGTTT
TAGTGAACGTTCCAACAAGCTTATATGTCCACTTGTACCTCGATTGGGTAGACGTGATGACCAGTT
TACACAAGTTGGCAGATTGCCTCCCTTTCATCGTCTCTATAGTCATTGTAATATACTTCAGCATATAC
AGGAAAGAGTTGTATGAGTTGACGGATTTCATGATGGAGAACTTCAAGTACCGGTCGGCGAACG
GGCTGACCAACATGACAATGCTGAACAGTTACAAGAAGGCGAAGGGCTTTGCCCGTTTCTACAC
GGCATGTACGTTGTTCAGCGTCACGATGTATACTCTCCCCGAGATCGTCAATTGGTGGACCGACCA
GCCGCTCCTGAGCCACCTGTACATGGACGTGACCAGGTCACCGTTCTTCGAGTTCACCTTCATGC
GCCAGTTCCTGTCGCAGGCTTTCGTTGGCTTGGCCATGGGGCAATTTGGAGTGTTCTTCGCCGCG
AACTCGATCCTCCTGTGCGGGCAGCTGGACCTGGTCTGCTGCAGCCTGCGCAACGCGCGCTACA
CGGCGCTCCTGCGCAGCGGCGCGGCGCACGCGGCCCTCGCGCGCGCGCACGCCGACGTGCTGCT
CGACGAGCGCCACAGCTACATATACAACGTGGCGGAGATGAAGGAGTCTGCCTACCATTATGATC
AGAAGATGAGTCGCATTTTTACCGATAGGAGGACACATTTCGACCTATACAGCGCGGAGTTCGAC
GCGGCGACGTGCGCGGCGCTGCGCGAGTGCGCGCGCGTGTGCGGCGCGGCTAACCGCTACCGG
GAGCGGTTCGAGCGGTTCGCGTCGCCGCTGCTCGCGCTTCGCGTGGTGCAAGTCACGCTCTACCT
CTGCATGCTGCTGTATGCGGCCACGGCGAAATTCGACATGGTCACTGTGGAGTACTTGGCTGCGG
TCGCTTTGGACACCTTCGTCTACTGTTTTTACGGCACCCAGATTATAATTCAGGCGGACCGCGTCA
CGACGGCGGCGTACCAGAGCGCGTGGCCGGCCATGGGCGCGACGCCGCGCCGCTTGCTGCTCAA
CATCCTGCTGGCCAACAGGAGGCCCGTGGCGGTGCGCGCCGGCATGTTCCTGCCAATGGACTTG
CACACCTTTGTAGTGATTATCAAGACTTCTTTCTCTTATTACACACTGCTAGTTAACGTTAATGAAA
AG

>CpunOR15

ATGGTCGGCCTCCAGAGCCTTGTGGAGGTTCATGGCGAGGCCCCGGGAGGCCGTGCCGAACGCG
TTACAATTTATGTACCACAGAGAGCAGAAACCATCCAACATAGCGATGATATGGTAGTACGCAAAC
GAGTGCCACAGTTCGAAGTCGTCTTGAAGGTAATGTTGCGACAAGATCACCGCAAAGCTGATGC
CCCACGA

>CpunOR16

ATGTGGCCCCTTAAGAGACGAACAAAAGTTGCCCCAAACAATGAGCCCAAAGTATTCTCTGCGG
AAGACTACGATGGCACCTTTTTTCATATAAAAAGGGTGTTGCGCCTTCTCGGTATACGGTTATTGT
GCGAGGATAGACCTTTAGTCAAGATGCTATGGGATGTGTTTTACTGGTTCGAATATGTAGCCATAG
CTTCAACGGCCATGCAGGATATACTTCTGGTACGCGATGTGATGATGGAAGAGAGGAATTTCGAA
GGTGCATTGAAGATCTTTAGGATGATACCGTGCATTGGTTATTTACTCCAAGCCATGCTAAAGACA
CATAACATCGTGTACTATCGATCCACTTTTGAGAACATGGTGAATGAACTACGAAGTATGTGGCCA
CAGGGCACTGTGTCCGAAGAAGAAACGACTGTTCTGACTGAATCATTGAAGGAAACTAAGACCT
TTGTGAAAGTTCTATTCTACAGCAACTACATACTAGTTCTCAGCATCGCCGTGCCCCCATACGTGG
ATGTGGTCAAACGATTCTTTTGGAAGGACACTCCTAAGATACTGCCCTTCACCAACTATTTGCCCT
ATGACCCTTCACCGCCAGTACTGTTTGAGCTCACCCTGACTTTACAGATTTGGGAAACTATAATCA
CAGTTTGGTGCGTATTACTCGGAGATCTACTGTTTTGCGTGCTCCTTAGCCACGTCACGACACAAT
TTGAACTGCTATGCATTAGAATTCAGAGGCTTTTCCGCGTTTCAATCGACGACCAACTTATTGCCA
CATACCCTCTTGCTGAAAATAACCATGAAACTTCGGCAACTAACAATGACGAATACACCTCGTTT
GAGCGAAAGATGATGTATCAAAGAGAGGTAGCTGAGATCGTCACTAGGCATACTGCCTTGATTAG
ATTAGCCGGCGACATAGAACAAATGTTTAGCTTCGCGATGTTATTCAATTTTTTTTACAACTCTATC
ATAATTTGCTTCTGTGGATTCTGCTGTGTGTTAATTGAAAAATGGAACGTTTTTATGTACAAAACAT
TTTTGATAACCGCCTTCATTCAGACTTGGCTTCTGTGCTGGTACGGACAGAAATTGTTCGAATCTA
GTCAGAAAGTTGCAGATGCGTTATACAACAGCGGTTGGTACGCAGCCCCAAAAGAAGTTAAAAA
ATCTGTATTAATCATGATTCAAAGATCGCAGAAAAGTGTGAACGTTACTACATACGGATTTTCTATC
ATCTGTTTGATTAACTACACTACAATTATCAAGACTGCTTGGTCGTACTTCACCCTTCTTCTTAATG
TCTATACGCCTGGCGAC

>CpunOR17

ATGGACGGATTTGAAATATACCACAAGGAGTTCTTAAAACCCCTTATAGTCACTTTGAAAATGCTC
AACCAGTTTAACGTAAGGTTCTGTTCTTCTGAAGTACCCTTCTTGACGAGAAACTGGAGGTTTTT
CTACCTAGTCCCATTATGGATTCTGCACATCATAAGTTTGATGTCGTTTATAGTAAATTTGATAATGG
AGGGAAATGATCCCTTTGAGGAGGCCTATATGATACCTATCTGCATAATCAGCTTTGAACATAGCAT
TAAGATGTGCATTTTAATAAAGAAACGAGAGGAAATCCGAGATGTTATCACTAATATTGGCAACAT
ATGGAGGTCTACTGGGTTAACAGATGACCAAAAGAAGACGAAAGAAGAAATCCTAAAGAAGCT
GTATAATGCAGGATTAGTATTCAACAACATCGGCATAGCTGTTACCATCCAGTACCTAATGGTGCC
GCTCTTCGAAACCTTAGTCCGCAGACTGATCCTAAAACAAGAGGTTGAGCTCATGCTACCATTGA
CCATTATCTACCCCTTTGAGATAAAAACCTGGCCTCTTTACTTGGTTATATATGGCTTTCAAGTCTA
CAGTCTGCTCTGTGCCGCTTGTGTGTACCTAGGCTTCGGCTGGCTATTAGTCGTCTTAACGTGTTA
CCTCAACATACAATTCTTACTTCTGCAAGAAGACTTGATACATGTGAAACCAGAAACCTCCAATA
GGTTGAGTTATCTGCCAGAAGAAGAGGGAGAAATAACGCAATATTGGGAGCCCTCTCCGTTTGGT
ATAGACGATTTCGTCAGGCAACATCAAACGGTTATACTATTAACTGATAAACTGAATGCTGCCGTC
AACAAAATTACTTTCACAATCATACTATTTGCTACGCTGAACATTAGCTTCTTCGCGATTGCAGTTA

AAGCTTCCGCTGGCGCAGTAGATATGGTCAATAATTTCGGAGCTATCATAGTCATAATGATGAATAT
ATTTATATTGTGCTATTGTAGCCAACTGCTAAGTTCGTCCAGTAGCGGCATAGCCTTAGCTGCAGG
AAAAAACCTATGGTACAAAGGTGATTTAAGCTATCAGACCAAAATACGTTTCATTATGATGAGATC
TCAGAAGCCGTGTTCACTGACCTCATTAGACTTTTCGCCAATTGGTCTGGAAACCTTTAATAAGGT
GCTGAAAACTACATGGTCATATTTTTCACTCGCCAGTCAAATGTATGATGAAAGATAT

>CpunOR18

ATGACGGATAAAGAAGATTTCGATAAAAGTATAAAACTATCGAAAACCTTTCTGCTTCTTTCGGGG
ATAAGACTAACGGAAATTAAATGGAATAGATCCATAGTGACGTTCATAGATCGCTATTTATTCTACT
TCAATTTATTTTGGCTCTACACTGATGTATGGGGTGAAATAAACTGGCTTTGCGAAGGACTATTAA
AAGGGACCAGTTTCATAGACTTATCAATCACCGTACCTTGTATTACCATCAGTTTGCTTGGCACTT
CAAAGGCTTTATTTCTAATAAACAACCGTGAAACTCTATTGAAAACAGTGGACAAACTTCGCAAC
ATCCATCCAGTAGACAACGACGATTTTGATGAGAACAGCAACCATTTCCGCTCGTTTGATGTATGG
AACAGCGATGTAACCAAAAATGAAGACGTGGTGAGGAAAATAGTGAGGGAATCGGTTAAATTCG
TAAACTTCATTGGTACGCTGATGTATTACATATGTTCTGTGGTCATCTGCGCTTTCCCTCTGATGCC
CGTGACATCCATGGCATATGATTACTATACGACGGGTCAGACCGAATGGAAGTACCCCTTTTTGGT
GAACTACTTCTTCGATGTAAACAATGTGTATATGTGGCCACCGGTGTATGTCCATCACGTAATTTCC
ACGTGCATAGTCGGAGCGAACGTCATCGGCCCAGACAGCCTGTTCTACGCGTTGTGCATCTACAT
ACAGATGCATTTCCGGCTGCTGTGTCATCGGCTCGAGAACGTTGTTGGTGCGGGGACACAGATCA
GGAGGGAGCTTATAAGTGCCGTGCAGAGACACCAGGAGTTGATTGCTATCGTGGACCAAATGGA
GATTATGTATTCGAAATCGACCCTGTTCAACATCTTAACAAGTTCCTTGCTTATCTGTTTGAGCGGT
TTCATTATCACCGTGCTGACGGACTTCAGCGTAGTGATCGCATTCGGCTGCTTCTTAATCATGAGC
CTCACTCAGATATACCTTCTGTGCTTCTTTGGAGATTCGCTCATGAGATCAAGCACGCAGATCAGT
TCGGCCGTCTACAACTCTCTATGGTACGAGACAGACCAGAGATCGAAGAAGATCATGCTCTTGAT
TCTGATGAGAGCGCAGAAGCCTTGCAAGCTAACCGCTTGTAAATTCGCCGACCTCAACCTGACC
GCGTTTACAACAATACTAAGCCGATCCTGGTCCTACTTTGCGTTACTAAGAACAATGTACAAC

>CpunOR19

ATGTTGTTAAAAAAATTTAAAGCTTTTTACAATAAAGATGGTTTTGATTACTCCGTGGGGTATGTG
GATCCCTTCGGATATCATCATACATTCATCTATATGATGAGGGCATTTCAGGTTTCAGGCGAGCCAT
TCAAAACTTGGACATATGTCTCGAAAATATTCACTCTTATTTGCGGCATAGGTGTTCTAACAGATG
CTTGTCTATCATTCTACCATGCCGTAGATATTTTTGACATGGGATTAATCACAGAAGCTGGGACTTA
TGTACTTATGTTGCTGTACAAGATGATGAATTTGATCATCACCAAGGTCAACTTGTCTGAGTATATC
AAACTTATGCAAGCTATGAAGGATGATTTCCAGTATATTAATACTAAGAACGAGAAGTATAAAAAA
GCGTTTTTCAAAACACAACACGACACATTCAAGGCGTGTGTCGTGACGTGCACATTTATGTTCGT
GCTAGCCACGAGCCTTGTGCTATTTGCCATCGGCAGCCTCTTATTCTACCTGGCGACTCACACACC
AGGGGACGGAACCCACAAGCCCTTGGTGTTTCCCTTCTGGGCCCCTGGAGTAGATTACACTACAT
CCCCAGCATTTGAATGCGCATTTACCTTTGCCAATATTGGAGTTATGGCTTGCACGTATAATTACAC
TTTTGTACTTCAAACAAATATAGTATGGGTCTGCCAAATCGCATCAAAGGCCGAATTGATATGCAT
GTGTATCAAAGACCTTTTGGATGGCATTCAGCCAGCGTGTAACGAAGAGGAAAAAAGGCACTAC
GCAAGTGTGATTAATTTCCGGATGAGGGAAATAATATCACTACATCAGTCAATGAACAGGTTGATA
GACAGCTACGCGTGCGTATATAGGAAATGTTTGATGTTCGAGCAGTTTGTGTCTAGTGGTGTAATA
TGCATGCTGGCCTACTGTTCCGCTGAGAAAATCGATGCAGGTGACATACATGTGGTAATGATGGTA
CTATGCGTTGGTGCTATAGTTATATTATACGTACCCTGCTATTTATGCACCTATATGCGGGAAAAGGT

CACCCTTATATGTGACGCTTGCTGGGATATCAGGTTTTGGGACGCGGGCCCCAACATACGCCCTTA
TTTGATACTGATCATGCAGAGATGTCTTCGCCCTTTGCCGCTTCAAGCGCCCGGCTTTCAAGAGGT
TTCGGTCAAAACATTTTCTAGCAAAATAACATCAGCGTATTCTCTCTTCAATATGCTCAGACAAGC
AGATCTTGATTTG

>CpunOR20

ATGTACTTCGCTGTGACATTGGTGCCACTTCTGTATCTGACCTTATTCGCTAATCAAGATGAACAC
ACAGTTCGACCGCTGATTTTCCCCATGTGGTTGCCTGAAGATGATCCGTACAGGACGCCCAACTA
TGAAACATTCTTTATCCTGCAAATAAATATCTGTTTCATTTTCGTCCAGGCGTTTGCGGTGTATGTC
TACATTCAGTTCCACGTGCTACTGCACAACTATTACATGTTGGACTTGGTGATCATGCACTTCGGG
GTGATATTCGAGGGCCTGGACGAAACGGTGGCGTACTTGCCGGTCCGAGACGAGCGCCGAGTCG
AGACACAGCGCATACTCAACAAGAGAATGGAAAGGATTGTTACCTGGCACAACTCTGTTTTTAAA
TTAACCGCTATGATATCGAAAGTACAAGGAGCACCCTTAGTATACCAAGTGATGGTGACTTCCATG
GCGGTTTGTTTGATGATGTACCAAATAGCAGAGAAACTTAATGAAGGGTCAATCGACATAATGTTC
TCTATGTTAATAACGGCGACTACGATCCAGCTGTGGGTGCCGTGCTACCTCGGTACTATGTTGCGG
AATAAGGCGTTCGATGTGGGCGAGGCTTGCTGGCTCTGCGGCTGGCACGAGACTTCGCTGGGCC
GTCTCCTGCGGCAGGACATCGTCATTGTCATCCTGCGCTCGCAGCAGCCGCTCTCAATCAAGTTC
ATCGGCCTCCCGTACTTGTCGCTGGAAACATTCTCCTCGCACATGAGCTCTGCATATTCGTACTTT
AATATGTTAAGACATTATAAC

>CpunOR21

ATGAATGGTAACGAAATTAAACCGGACCCCATGAGCCTACCGTACATGCGGTACACTCGCTTGAT
GCTGCAGTCCGTGGGCGCGTGGCCCGGCGACGCCGTGGCGGGCCGCGCGCTGCGCCCGCGCCTC
TACGCCTACTACCTGATGCTGGAGACCGCCGTGCTCATCGCCGGCGAGCTGCTCTTCATCCTCAA
CCAGTTCCACGTGCTGTCCTTCTTTGTTCTGGGCGACGTGTATATAGCGCTGTCTTTGTCCTTCTTG
GTCATGTTCAGGGCTGTCCTGTCGTGTTTCAAAAGATATGGAACGATATTCCGAAAGTTTATAACA
GAATTTCATTTGATGCATTTCAAACACAAAAGTGAATACTCAAATCAGATGTACGAGAAGGTGAA
CAAGATCTCGCACTACTTCACGCTGTTCGTGATCGGGCTGTCGGTCTCCGGCGTGGTCTCCTTCA
ACGTCACCCCCCTTTACCGCAACTACCGGCTCGGGGTGTTCCGGGACGAGCCCCCGGAGAACGT
CTCGGCGGAGTTCTCAGTCCACTACCTGTTCCCGGGCTTCCGGCAGGAGGACTTCTACCTGCCCT
CGACCCTGGCCAACGTGTTCATCTCGTACGTCTGCGCCTTCATCATCTGCACCATGGACCTGTTCC
TGTGCCTCATGATCTTCCAGATCATCGGCCACATCAAGACGCTGATACACACCTTGAGGAACTTCC
CGGCGCCGAAGAAGATGAAGGAGCTGCAGGGATTTCGGCAGGGGTCTATGAACAAAGCCCCTAT
AGAGGTGGAAATTGTGAAACAGTTCGACGATCAAGAGAACGAAGTTATTAAGAAAATTATCATCG
AGTGTGTCGAGCATCATCTTTTCATCGTAAGTTTCACAGACGACATATCGAACTTCTTCGGGCCGA
TGCTGGCCTTCAACTATCTGTACCATATGTTCAGCTTCAGTTTGCTACTCGTGGAGTGCATGCAAG
GGCAAGTCGCCCTGATGCGCTACGGACCCCTCACCATGATAACACTCGCGCAGCTGACACAACT
GTCTGTCACTTTTGAGATAGTAGGATCAGAGAGTGAGAAGTTGAAAGACGAAGTGTATTACATCC
CGTGGGAGTCCATGAACAAGAGCAACCAGAAGTCGGTGGGGATCTTCCTGAAGCGCGTCCAGAC
GCCCATCCGGGTCACCGCCATGGGCATGACGACTGTCGGCGTGCAAACTATGGGCGGGATTCTCA
AGACGACATTTTCGTATTGCGCTTTCTTGAGATCGTTGAATCCA

>CpunOR22

ATGAGACAAAGGAAAGGGGCTACTTATCATAAAGCTTCCCCGACATACAATTTACGAAAGCAGAT

AACACAAACAATTAACAATACCGGGATAACATTCGAAGATGACAGAATAAAGTGGCATTGGATTG
CCTCAGTATCCGTTATTTGCTTTGTCATCGCCGATGTGGCAATGATCATTGCTTTGTTCAGTGTTGA
CGACGAAGAACGCTTTTTTGAATGCTTCAGTGTTCTTTGCTTCTGCTTCGTTGGGTTTCTTAAGTT
TTTATCACTAAATTGCAACCACAAACAATGGAGTTCTATTTTGAGCAATATAACAGCGTTAGAGAA
AGAACAGATCAGCTATGAGCCGGCGAGTGTTGAATACGAAAGTGACGATGAAAACGAAACATTT
AATTTTCCTCAATACATCAACTCTTACACCGCTCAGTTTCAAGCAGTTTCGACGTTTCTCTTCAGG
ATGTACATTACGACATTGATCATTTTCGCCGTATCACCTTTTGTGGAGTACGCCTTTCTCAGAATAT
TGGGGGAGCACGTGGAATGGCTACGTATACTACCGGGTTGGGAACCGTTTGCTAACAAAAGTTTC
GTCGGTCACGTTATCAATATTATTATAGAGATAATCGGCTCCTGTTATTGCGTTATCCTTCACGTAGC
ATTTGATCTGACGTCGATTGGAGTAATGATATTCATTTGTGGCCAGTTTTCTTTATTGCGTGTTTAC
AGTGAAAATATAGGAGGCAAGGGAAGACATTGGCAGTTGTCGAAAAGAAGGGATGAAAGGGCA
CATCAAAGAATTATCAGATGTCATAAAATTCACGGGCTGTTGATGAATACAAGTAACGAATTGAGT
AAATTGTTGACGAATGTACTGGGCGTATATATTTTTTTGGCCACACTCACATTATGTTCGGTTGCGG
TACAATTAAATTCGGAACTGAGCAGTATGCAACTGGTCTCCTTGGTCCAGTACACATGCGCGACG
CTCACGCAGCTCTACTTGTATTGCCGCTACGGGGACAACGTTCTACACGAGAGCTCCATAGGCAT
GGGACAGGGCCCGTTCGGCGCGGCGCACTGGTGCCTCAGCCCGCGCACGCGCAGGGAGCTGGC
GCTGCTGGGCGCGGGCATGATGCTGCCGAGATACATCCGTGCAGGCCCCTTCACGAGGCTGGACT
TGCCGTCTTTGATCAATGTTGTGCGCACTGCATACAGCTACTATGCAGTGATCAGGCAA

>CpunOR23

ATGATGACCAAAGTGAAAGCCCTGGGCCTCGTGTCCGATTTGATGCCAAACATCAAATTGATGCA
GGCAGCTGGGCACTTCTTGTTCAACTATCACTCAGATAACTCTGGCATGGCAATGCTTCTACGCAA
AATCTATGCCAGTGTCCACGCGTTCCTCATCGTGATTCACTACTTGTGCATGGCAGTGAACATGGC
CCAGTACTCCGAAGAAGTCAACGAGCTGACCGCTAACACGATCACAGTATTGTTCTTTGCGCATT
CTGTCATCAAATTGCTGTTCTTCGCGCTCAACTCTAAAAGCTTCTACAGGACCCTGGCAGTATGGA
ATCAGTCCAACAGCCACCCACTCTTCACCGAGTCAGATGCGCGCTACCACCAGCTCTCGCTAACC
AAGATGAGGAGACTGCTGTACTTCATCTGTGGGGTCACTGTCTTGGCTGTCGTCTGTTGGGTGAC
CATAACGTTCTTCGGTGAATCAGTCCGCATGATAGCCAACAAGGAAACCAATGAAACTCTAACTG
AACCAGCCCCTAGGCTGCCTCTGAAAGCCTGGTACCCCTTCGATGCTATGGGCGGCACTATGTATG
TGGTCGCTTTTGTTTTTCAGGTATACTTTCTCTTCTTCTCCATGGCGATTGCTAACCTGATGGACGT
CATGTTCTGCTCGTGGCTGATCTTCGCCTGCGAGCAGCTGCAGCATCTGAAGGCCATCATGAAAC
CCCTGATGGAGCTCAGCGCCTCCCTGGACACGTATAGGCCTAACACTGCTGAGCTGTTCCGAGCT
TCGTCCACAGAAAAGTCAGAGAAAGTACCAGATCCTGTGGACATGGACATACGCGGCATATACTC
CACGCAGCAAGACTTCGGCATGACTCTGCGCGGAGCCGGCGGTCGGCTGCAGAACTTCGGCGGG
AACCCGACCAACAATCCTAACGGGCTGACGCAGAAACAAGAGATGCTAGCGCGCTCCGCCATCA
AGTACTGGGTGGAGAGACACAAGCACGTTGTGAGACTAGTGGCGTCCATAGGGGACACTTATGG
TACCGCTCTACTATTCCACATGTTGGTATCTACTATCACTCTCACTCTACTGGCCTACCAAGCGACT
AAGATCAACGGCATAAACGTATACGCCTTTAGTACCATCGGATACCTGAGCTACACACTTGGTCAA
GTATTCCATTTCTGCATATTCGGGAATCGTCTCATTGAAGAGAGCTCCTCGGTTATGGAGGCAGCA
TATTCCTGCCAATGGTACGACGGCTCCGAGGAGGCCAAGACCTTCGTTCAGATAGTCTGCCAGCA
GTGCCAGAAAGCCATGAGCATCTCTGGAGCCAAGTTCTTTACAGTGTCACTGGATCTGTTCGCTT
CTGTACTCGGAGCGGTGGTAACTTACTTCATGGTGTTGGTACAACTGAAG

>CpunOR24

ATGAAGTCATATCGCCTAGGGTTCGATATGGAATCGACCGCTCAATCGCGAGTGTTTAAATACAAC
ATTTTCTTTTGGAAATGTTTGGGATTGTGGTCAGATGAAACATCTGGATATTGTTACAAATATTACT
CTGTTGCTTTTCAGTCATTTTATTCAGGAATGTTTACGTTTCTTTACACATTGAATTTAATGTTCACA
CCATTTGATCTCGAAATTATCATATCACAAAGCATGTTCTATTTTACTCAATTGGCTCAGATTACGA
AAATAGCAATGATTGTTTTACGGAGAAAATATATATTGAGAGCTTTTAATATGTTGGATTGTACAGC
ATTCCAAGGAGTTGACGAAGAAACTAGAGAAATTGTGAACAAAAATAAATGTTCATATACAAACT
ATTGGAAAGCTTGTTGCGTGTATTATCATATTAGTTGCTTTTCAATCATTATTTTGCCGATTATTAAC
TATTTTGTTAATGGAACTGTAATAGAGTTACCACTTTGTCAATATTATTTTTTGGGCGACAATATACG
GGAAAAATATTATAAAGTTTTGTTTGCTTATCAATTTACTGGTATAATAATGATCATGTATAGCAATC
TCAACACAGATACTTTTATCAATGGGTTCCTTATGATGGCAATAACACAATTTAAAGTGTTATATTG
GAAATTAGCTCATTTGGATTCGACATCGTTGGATCAGGGTGTTATAAACGAAAAAGATAAAGAAA
TCTTGATGATAGATAAACTTAAGCAGTGTCTTATACATTACGACCTCATTTTGCAATATTGCAATATC
ATACAAGATGTGATGAGTTTTGCGATATTTGGTCAATTTGGAACTGCAGCAGTTACTATATGTGTTT
CCATGTGCTTTTTCCTTAAGCCGATGACAAATGAAGATTTATTTATAACTACGTTTTACCTGCTTGC
TATGATATTTCAAATTTTTTTGCCGACATATTTAGGAGCGGAATTAACTGAGCAGAGTGAATTGATA
GTAACAGCTGCGTACAACAGCGACTGGATACCGCGGTCGGAAAGCTTCAAGCGAAGTCTTGGTC
TGCTCATGGAGCGCGCCAAGACACCCATAGTAATCACTGGCCTCAAGATGTTCACACTGTCTTTG
GAAAACTTTACTTCGATCATGAAAATGGCGTATTCTTTATTCACGCTTCTTAAAAATTCACAAGTG
GAAGCT

>CpunOR25

ATGGATGGGTCTTCCGAAAAAAAAGAGGACCCCTTGCAAAGGAACCATATACGTGTCCTACGCG
CCATTCTTATGACCCATGGCGCCTGGCCGATTGGTGAATCGTTTATGAGAACGTATTTCACAGCTG
CGGTTGTCGTATCTGTAATCATAAATGTCTTTGCAGCAGGGCACTACATTATTGTAAATTTTGCTGA
ACTTGATTTCATCGACATAGGAGATCACTGTATGACGGAGTGTCTAGCTATTCTGACATTAATGAG
GTCCATATTTGTTAGGGTGAAAGATTATGGAACTGTCACTGAAGAATTTTTAACGAAAATTCATTT
AGATCATTTTAGACATTGTGGCGAAAATTATGATAAGATATGCCGAAGGGTAAACCAATTTTCGTA
TTATGTTACTCTGCTAATGGTAGTAATTGCAGCTCTGGGCCTAAACTTCTTCAACATAGTACCTATT
TATAACAATTTTCGGAACCGTAATGATGAGGACGCGGTTACTCAATATTCGATCTATTTGTCACTTC
CGGGAGTTAACCAAGACATGATATACACATTTTCGACTATCTACAACTATTACATAAGTGTAATGTG
TGCAGTTCTTGTATGTGGCATTGATTTACTCATGTTTGTAATGGCATTTCACGTAGTCGGTCACATA
ATGACCCTTAGGGACGATATAAATAATTTGCCCAAGCCAAAAGGTAAAAAAGATCCTGTTCATATG
AAAAATAACTACCAAACTAATTTACATGTGGATATATACGACAATGAAGAGAATGCTAAAATAAGA
GAAGAACTCCTCAAATGCATTGATCATCATGCAACTATTGTCAGTTTTGCAGGAGCTTTATCAGAC
TTATTTGGCCCGATACTTGCTATTTCGTATGTAAATCATCTAGTCTGCGGTGCCCTTATGTTGTTGGT
GTGTACAACGGGGGACCGGAACAGCGTCGTACGTTGTATTCCTGTTACGATAGTAGTGTTCAGTC
AGCTTGCTCAAATATCAGTGATTTTCGAAATCATGGGATCAGAGAGCGCAAAATTGATGGATGCA
GTGTATAACGTACAGTGGGAGTGTATGGGCGCTAATAATAGAAAACTAGTGCATATGTTCCTTACC
AAGGTACAAACTCCAATACGCGTCACGGCTCTAGGAATGGTAGACGTGGGAGTGGAAGCCATGG
TTGCGATTTTGAAAACAACATTTTCTTACTATACTCTACTTCAATCATTGGGCGAA

>CpunOR26

ATGAAAATAATTAAAAACGCACAGCTCGTAAATAATAAAAGAGAAAACATAGATATAACCCCACC
GAGAGATCAGTTAATGTACAAGATCCTCGCAAACGTGTTAGAACTCTATGGTCTCGCATCTATGAA

GCTATGGAACATACCGCCATACCACGTATATTTAAACAAGTTCTACGCAATAAACAGTCTATTGATC
TATACCTCTCCGTTATGTATACTATCTCAGTTATTGTATTTGTATGTTCATTTTAATGATATGTCTTTCT
ACACGCTTGGTGTTATGTTTTCTGTATTACCAATAGGGATTACGGTTTTAATCAAAATAGCATCGAC
GAGAACTAATGCTTACAAAAAGATCATGGTCAACTTTATGAATAAGATTCACGTCCATAATCAACA
TTTGGAAGAACCAGATAACGAATTTATTAAATCGGTAGTTAAGCAAACGGAAAATTTCACTCACT
TGGCCACTTACTTCCTAGCTTTTTGCCTGCTCACGGACTGGACTGCATGGACCGCAATTCCTGTAA
TAGATTATTTAAAAAACAAAGAACTTATAGAACGGAAAGAAAAAAAACTCGAAACATGTTTATAT
CTGTACACGCCATTTGATTACAGTTATGATGTCGGCAACTGGTTGATAGTGCATACATTTGGTTCTT
ACCTGAATTTCGGTGGAATAACTATAATAATAATATTTGACACATTGTGTTTTATCTTTGTATTCAAT
TTGGTATCTCATATGAAAATTTTGCAACATAAAATGGAAAGGATTTGCAAAATTGATGAAATGACT
AACGAAGACATGCGTAAGAAACTTATTGAGATTATTGAGTACCATGCGCAAATAATTAATTACTTT
GACGATATACAAGCCGCATTTGGACTTAACATTACTGCAGTGTACGCGCAAAATTTATTCGTAGAC
AGTCTTTTGCTATACCAAATAATGGTTGGTGTAAATGGGGAAAAGACTCATGTAATAATATTCGGG
CTAATGTTCGTGGCGTACATGGGAGGGCTTACTTTCATGTCTTTTGTACTAGAAGAAATAAGAAGG
CAGAGTGAGACATTCAGTGATCTAATCTATAGCCTTCAGTGGGAAGACATGTCCAAATCAAATCA
AACTACGGTGATTATGATTCTCGCAAAAATGCAGCCCCCTTTAACTTTTACTGGCGCTGGAGGTCT
ACAGACAGGAGTCCGTCCTCTGGTTTCAATTATCAAGTCTACGTTTTCTTACTATGTTATGCTCAAT
AATAGAACTGCTACACGT

>CpunOR27

ATGACCATATCCCTTGGCGTGTGCTTGGTATTCAGAAACAAATTCAGAGTTTTCTTAACCGAAATG
GCGTTTAAGGACGAGATGTTAGAAATGCCTTTAATCAAGTACGCTTTCAAGTTCGGGGAGGGAAA
AAAGCTGATGGAGCTGAAAAATATGGTGATGGAGTCTCAGGAGAAGCTGTTGAAGTATACCAGG
GTGTTGTTGAAGAGTTATGTGATCAGTGTATGGCTGGTAGCTACTTTGTATCTTTGCAGTCCGATTT
ATGAGATGTTCGCCAAGGGAGACCCTACGCTGAGACTCCTAGCTTTTGATATGTGGTTCCCGTGG
AGCTTAGAAAAGCTCGGAGTGTACATCGCATCATTCATATTTCATGCTTACGCTGGATATTTGTGTT
GTATTGCGTACCCCGGCTTACAACTAACGATCATACTGCTGATTGGGCAGATAATACGTCAGCTTC
GCATTGTCACCTTCATCATGAGGAATCTCAACGAGCTGGTGGTGGAAATAATCAAGGACAGAGAG
AGTAAATGGCAGATATGTTGCACTGCTGTGTTGGCACAATGTGTCGACCATTATATTAAATTGAAA
AGGTTCAGCAACCGCCTAAATGTGATATGCCAGCCATTTTACCTAACTTTGATACTGGTCGCCATTA
TGCTGGTTTGTATGTGCTCAGTTAAAATTGCTATCTCGAACAAATTATCTCCAGATACAATCAAATA
CTACGTGCATGAATTTTGCTTCATAATGGTGGTTTTGATGTTTTGTCTGTTGGGTCAACAAGTTGAT
ATTGAGTGCGAATTGTTGGAGCTAGCTGTTACAGAAAACTGGTATATATTTGATAAAGAACACAA
GATGAACGTTCGTATATTCAAAATGGCGCTCAGTCAGCGGATGCCTATTTTCATATTCGGTTCTATT
AAATTGTCCCTGCCGACGTTTACTTGGTTCATTAAAACAGGGATGTCTTTCTTCACACTGGTGATG
TCTGTATTAGAAGAA

>CpunOR28

ATGAAATTCTCCATTGAAAATTATACTCACACAGTTGGCAATAAGAACGAAATGAACGGTATAATG
CGTATAGTTCTTGTATTACAGAGATTTTTCGGCCAACATATCCTTAATCCCGATTGGACGTGGAAAC
GGTTCTGCTTGTATCAATTGGCTACATTTGCTTTGTTTTCATACGTACTCGCCGGCACGTTTGCGAT
ACTTCAAGAGACGGATGACATAAAAATGATAGCCGAAGCCACGTATACAGTCGTCGTGACGGGT
GTATCATTCGTCAAATACTGCATAATCACGAGCAAACGGTTTGTTTTCAGAAGACTGTACGTGGAA
TTGAAATCCAGTTTATATGATATCGTTAGGGATGACTCCGAGGAGAAGATGAAGAAAGTATTCGAT

GATGGGAAAAAATCAGTGTATTATATATTTGTCTTTACTCTATGCCCTATAATAATGTATATAGCCAG
AGTTCTGTGGTATAACCTGCATGGACAAAAGGTCACTTTGTCCCTAACGACATCAATTTTAATGCC
GATGGAGACACCCTACTACCAGCTAGGGTTGATCTTACACTCCATATACTTTATCGAGGTGTCCTTT
ATAGTAATACAGGTCGATATGTGGTTAGTGCTATTTGTGTTTTTCTTCTGCGTAACTTCTGACATAA
CTTTGAAGATTTTGACCGTTGAAAAGAGGAAGCAGGGAGAAGATAGAATTCAATACGCAGTTCG
CCTGAATGATAGCTTGAAAAGATTCTATAGACTCCACGTGAAGCAAGTCAATTTTCTTAGCATGTT
AAATGGCACATTCAAATGGATTACACTGGTGACTCAAGTCAATATATGTATATGCATATGTATAGTA
CTTCTACTTGTGCGAAAGGGTGCAGAATCGGCATTTGCCCTGAACATACTGCCCTCTATCGCAGA
GTTAATTGGCTTCAGCTGGCTCGGAGAACAAATTAAGACAAAGACAAACAATTGGAAAATGGCT
TTACTGGATTTCGATTGGATTAACTTGCAACAGAAGGACAAGAAAAACTACTACATATTGATGTGT
TACATGAACCAAGAGTTTGGTCTTCAGTCCGCCATTGGTGGCGATTTAGTGCTTATTACTGTCAGC
AAAGTGCTCAAATTCAGTTACCAGGTCTACACTGTGTTGCAGAGTATG

>CpunOR29

ATGGTAGCGAATATGACAATCGGGGTAAATTTTTTTAATGTTGTACCATTTTATAACAATTTGAAAA
ATGGCATGTTCAATAATAATAAGCCGAGTGACTATAAGCCTCAGTATTCGATTTATATGGGAATTCC
AGGGCTCAAACAAAATGAATATTTTTTAATATCAACAGTTCATAACTATTTCTCGAGCTACTTTTGT
TCAGTTTTGATATGTGCCATTGATTTACTTATGTTCCTAATGGCATTTCACTTGATTGGTCATATCGC
TGCCCTTAAACACGATCTACATAATCTGCCCAAACCG

>CpunOR30

ATGGTCGACCTACCGGTGGACCAATCATTGAAGAAGATCAAGTTTTTATACCGTCTATCCGGTATG
AATATAGAAGATCGAGATAAAAATACAACTGAGAAAGCAGTTTATATGTTCAATTTCCTCTGGATC
CTGACTGATATAATCCTAGCCACCGAGTGGGGTATAGTAGGCTTCGTGAAGGGATTAGACTTCATA
GAAATTACGCATGTTTTACCGTGCTTGAATATGGGCATGGTAGCGGAGATGAAAACGATATTTCTA
GTTGTTTACGAGGAGAAACTCAAGCAGCTGTTGAGAGACCTTCGTGACTTAGAAAGGATTAGGT
TACCGAATAATCCTTACGCGAAGAAGATAGCGGATACTGACGCGAAATTTTTGCACAGTCTGATA
AAGATGACATTCACAGTAAACATATGTTTACTGATACTGTTTAATTCGGGGCCGTTGGTGCTTATAG
CTGTCAAATACTATTTGACTGGAAAATTGGAGCTGTTCCTGCCCTTTCTTGATATATATCCGTTTGA
TTCATATGACCCTAAATATTGGGCCTGGGCGTATTTACATGAAGTGTGGACAGCTTGCATAGTCCT
CTCAGAGATATTCGCGGTGGACTTCTTGTTCTACGTCTGCTGCACCCACATTGGCATACAGTTCAA
GATGCTGAAGAGGGAGCTAGAACACCTCATTACTGGCAAAGGGCTTCTGGCAGACCACGATGAT
CAGCTGAAGGAAAAGCTAGCCGACATTGTGAAATGGCACCAGCAAATAATAAGTTGCGCAGAAA
TGCTAGAAGTTATATACAGTAAGTCGACGCTTTTCAACTACATAAGCAGCTCTTTGATCATCTGTCT
CACTGGATTCAATTCGATGGTAATCGATGACATGGCGATAGTCATCTCGTTCTTCATGTTAGCTGTG
GTCGAGGTGTTGCAGATATTCTTCTTGTGCTTCTTCGGAGGCAAGCTGATAGATTCGAGTACAGAT
GTGAGCTCCGGCGCTTACAACTCAAAATGGTATTTAGCTGATATACCCACGAGGAAAACGATTCTT
CTGATACAGACCAGGGCACAAAATCCATGCAAATTAACAGCAGCAGGCTTTGCTGATGTTCATTT
GAGGGCATTTATGAAAATATTAAGTACTTCGTGGTCCTACTTTGCTTTACTGCAAACAATGTACGG
GTCATCCAAAGTTAGCCAT

>CpunOR31

ATGCCTGATAATCATGCATATTCGTACAACGTTATGCAAAAGAAAATGTTGCACGAGATAAAATTT
CTGAGTTTATTAGGGGTCGCAACATTTGTCTACCCCTTTATTGGGAGATCCAAAATTGTTCTCGGT

TGCTACAGCTTCATTTATCTTTTGGTACTTTTAACGGCAACACAACTCATAATCACACTTTATTTAA
AAGATTTCAGTGATTGGGTTGAAATTATTAACGTTGTGCCCAATTTAGCTGTAGTCCTTATGGCAG
TGTTGAAATACAGCAAAGTACATCACAATCAAAGGTTTTATAAGAAACTGTTTGATCATTTTCGTA
ACGACTTGTGGGATGCGGTATCTGACTGCGAGCAACATAGGAAAATTGTTACTAAATACACGGAT
ATTTCGAGATATGTGACAAGATTTCTGTTCTATTATTCTGTCGTCCTTGTCATATTCGTGTTTTCATT
TCCGAGATTCATAATGTACTTGCAAAAAATAATAACTGGCGAAGAATGTCACCTGTACCCGTTTGA
CGGATGGTACCCATTTGACAAAGTTTCTTGGTACTACGTCGCTTATATTTGGGAATGTTTCATGACT
TTCGTTGTTGTTTGCATATACGGATTCGCCGGTATTTTCACATCAGCTATAACAATATTCATTTGTAT
GGAGCTAAAGGTTTTGGGAAGCAGTATACAAATGCTAATAAGTCCTAGTGATGCTGCTAAGCTAA
CAAAATCTCCGAACGACAAAAAAATTCATCAAGACATAAGAAAAAGATTGAGAAGTATTATAATT
AGGCATCAAGTTTTGGCAAAGTTATCCGCGGATTTCGATGGTGTATTAGGCGATATCATGTTGATTA
ATTACGTGTTTAGTTCAGTGTTCATAACGCTAACTATATTCACAGCCACGGTAGTAGAAAACTTATA
TATGCGTATGCGTTACTTCTTCATGTTCTGCTCCTTGATGGTGGAAGTGTTCCAACAATGCATGATT
GGACAAATACTTAGCAATCATAGTGAGGAACTATCGGAATCAACATACTTCGCCGATTGGACTTAC
GCTGATAATAACACGAAGATAATGCTCTTGATACTCATGACCCGTACTCAAAGACCATTTGAGTAC
ACGGCGAATAATTATTTGGCTATGAACTTACAGAGTTTTAGTAGCATTTGCAGCATGTCATATCAGT
TTTTTAATTTACTTTATACGGCTTACAAT

>CpunOR32

ATGATTTTCGGTTCCAAAAAAAAATAAGCAAGAGAATGACATCGAGGAGCAAACAGAGTACACTT
TCAGACCATTCCACGAGACGTACAGGTGGATCGCGACTACTCTCACGCTCGGACTGATGTACCCC
AACCCGGCTACTGACAGGACCAGGCTCATACTCATTGTAGCGTCGTTGCTGATGATGCAGCCGGT
GATCGTGTTCATCTTGATAGACATGTACATGTGCTGGCTGAAGCGGGACATCTTCAACATCATCAG
GCACAGCACCATCATAGGACCGTTCCTTGGAGCCTTTTTCAAGATGCTCCTAATGTACTACAAGA
GGGTCCAAGTGAAGGAGCTGATAGACGAGATGAACCGCGACTACAGCAGTTACAACCAGCTGCC
GCGGCACTGCCGCGCCGTTGCCGCCGCCGGCGTACGCGCGAGCGTCACGCACACCGAGCGGCTG
TGGGCGCCGCTCGTCGGCATCGCCATCATGGCCTTCCCGGGCGTGGCCGTGGTGGGCACACTGTG
CAGCCACGTGTTCGCGGCGGCGCCGCAGCGGTACATGGTGCACGACCTCAACCGGCCCTTCCGC
GAGCCCGAGGCGCGCTTCGACTCGCCCTACTTCGAGACGCTGTTCCTGTTAATGTTCGCGGGCGC
CATCATCTGCGTCTTTAATTATACTTCATATGACGGGCTGTTCGGGCTGATGACGCGCCACGCCTG
CCTCAAGATGAGCATCTACTGCCTGCGCCTGCACGACGGCTTCCGCAGCGACGAGCCCGACGAG
CTGTACCGGCGGCTGATGGAGTTCATCAGGGAACAGGGGAGGATGTTCAGATTTGGCGCATTAAT
TCAGGACGCTTTCAACATCTGGCTGGGTACAATTCTGATCAGCACCATGATACAAGTAGGGTCAC
TGCTGTTTCACATTTCAGCGGGGTACGGGTTCGATCTCCGGTACATGCTCTTCAGTGTATGTTCCG
TGGTCCACATCTTGTTGCCGTGCAAAAATGCTGCGAATTTGAAGATGATGTCCATGGACACGGCC
ACTCTGGTATATTGCTGCGGTTGGGAGCGCACTAGTTGCAAGCGGGTACGACGAATGATACCATT
CCTCATTGCGCGCGCGCAGCGACCCATCCGCATCATGGCGTTCTACATGTTCCAGTACGATATGGA
GCTATTCGTCAATATAATGAGAACTTCGTACTCTATGTTCACATTATTGCGGTCA

>CpunOR33

ATGGCTTTTGTTTTCTTTGTGGTCACTGGAATCGGCATGTACATGAATCAGGATGATCTCATATCTT
TGCTGTCAGATCTGGATAAAACCATCGTCTCTTACAATTTCTTCTTCAAAATCATCATGTTTCTTATT
AAACGGAAACAAATAAGGATTCTTATCGACGATATACTTCATTCTGGTGATAAAATCAATGAAGAG
CGTGAAAGGGTGATGAAAATCCATGTGATATTGGTCTCTGTATTGATCACGACGATCATCGGTGGT

TTCCAAATGCTGAGTCAGATAAAAGGAGAGCTGGTGATCAGCTCTTGGATGCCGTTTGATGCTGT
CAAGAACCAGTGGACCGTCTTCATGGCTGGCCAGATCCTGGACGGGTTGTATGCAGTGCCTGTGA
TGTATAGAGCAATCGCTATACAAGGCATAGTATGCAGTATCATAATGTACATGTGCGATCAGTTGGT
GGAACTCCAGCAAAGACTGAAGACTTTGACTTACAATGAGGAAAATGGTCATAATACGCGAAAA
GCATTCAAGAGAATTATTAAGAAACATATACGCCTAGTTGGCTATTCACGGTCAATAAAATCAGTG
TTCAAAGAGTATTTCTTCATACAAAACGTAGCCATAACAGCGGAACTATGTCTCAACGCCCTAATG
ATGACCATTATGGGATTGGAACAGAAGAAACATCTTGCAACTTTCATGGCGTACCTGATGATAGCT
CTGTTCAACGCTTACATTTTTTGTTATTTGGGTCAAGAGCTGATGGATCAAAGTGCAGGTATAGCT
CTTGCTGCCTACGAGTCAGACTGGACTTCATGGCCAGTAGACATGCAGAAAGACCTGCTTCTACT
CATCACAGTAGCCCAGAAGAGCTTCACCTTGAGCGCTGGTGGAATAGCCGTGATGTGCATGGAG
ACTTATGCTCTGGCTTTATACAACGGATACTCGATATTTGCTGTTTTACGTGATGTAGTTGAT

>CpunOR34

ATGTGGCAGTCAGCGCGCAAGTTCGGCCTGGAGCACTGCGACCTGGAGACCATGCTGCAGAACG
TCAACCTGCTCTTGCGCACGCTCACGCTCAACATCGACAGCCAGAACAAGAAGCCCATTCCGTG
GGTGTACTATCTGGTGACGCTGGTGCTGGGGCTGTGCTACTACTACGTGTTCCTGTTCTGCATGGC
GTGGGTGGTGGCGTGGCGCAGCCGGCAGACCGGCGACTGGCTCGGCGCCACCATCGTGGCCTCC
CTCGGCATCACCAGCGAGATCGGCACCACAAAACTGCTCTACATGATCTTGTATATCCGCCAAATT
CGGGAACTTGTCGACTTGTACCGAGACTGCGACAAGTTGGTGTCGCCTGAAAGCCGGTTCGCAG
ACAATCTCCTCAAAACGCTCAAGGTCGTGAAGAAACGAGCTATTTTTTTCTGGATGGTGCTCGTC
GGGAATGGCGCAGCTTATATATGCAGGCCTTTCTTGAGGCCCGGAAGACATTTATTGGAAGACAG
CTTCACAATTTACGGTTTTGAACCAAAATTCGAATCGCCAAATTATGAAATTTCGAACCTCCTACT
ATCGGGTGGGATTGTGTTCGTAGTCTACCTTCCATCGAACATCAGTGCCTTCCTGATTATCATCACC
GGGTACACCGAAGCCCAGATGATTGCTCTTTCTGAGGAGATGTTGAATCTATGGGACGAGGCTCA
ACGACATTACCAGCATAGAGCCGCGACCTTTGCCACCAGCTCTGTTTTGATACAGAGCAGGGAAG
CAAAGTGGCAGGAATACACGGCAAAGAAAGAAATCGTTAATGAGTACATCAGGTCACATCTGCG
CGAAATCATCAAAATGCACAGAACGAACATTTTCATTGTGCAGAAGGTAGAAAACGTCTATCGGA
ATTTGATTGCTGCTGAGTTCATACTGCTGACGTTCGGGATCACAGTGGAGCTGCTGGGGCGCCTC
GAGAACACCTACCTGCAGATACCGTTTGCGCTGATGCAGGTGGCGATGGACTGCTACTGCGGGCA
GAAGGTGATGGACGCGAGCGTCGTCTTCGAGAACGCGGTGTACGCGTGCCAGTGGGAGAACTTC
GACGTGGACAACATGAAGACGGTGCTGGTGATGCTGCAGATGTCGCAGAAGACGATGCGGCTGT
CCGCGGGCGGCGTGACCATGCTCAGCTTCACCAGCCTCGCGTCCGTCATTAGAATCATTTATTCTG
GATACACTGCTTTAAGGCAGCTCTAT

>CpunOR35

ATGTTGGAGAAACTGAAGAGGTTCGGCCTTGGGCACTGCGATCTGCCCACCCTGCTGTGGAATGT
AGCCTTCATGCTGAAAGGGCTTATGCTCAACATCGATAAGAGATATATGAATCGCATCCCAACGAT
ACTATATATCATTAATGTGATACTCGCCGTCAGCTACCTGTACACATACTTCTTTTCAATGATATGGT
TCGTGTTTGTCCGCTGCGTTCAGACTAAAGATCTTGCTGCTGCAATGATTGTCTTCTCGCTCGGCA
TTACTAGTGAAATCGGTGTTGCAAAATTTATCAACACCTATATTTATAGAGACGAGATCCGAAATCT
GCTGCAAGACTGTCTCGAATTAGACTCTACAACTGTATCTGGGAGCCGCTACTCCATGAATTTACT
ACAAACTCTCAGAACAGTGAAGAAGCGCGCACTAACGTATTGGATAATCATCATCAGTAATGGAG
TCCTGTATATTCTGAAACCTATTGTGATGCCTGGTCGCACCGCTATGGAAGATGTCTTTATTCTTTA
CAAACTTGAACCAGAGTTTGAAACGCCAAACTACGAACTGGCTTATTTTCTCAGCGCTACCGGGT

CTGTCCTCACCTGCTATTTGACCTCAAACATGGCTGCCTTTCTGATCATCGTAGCTGGTTACTTGG
AAGCCCAACTGCATGCTCTTAGTGAAGAACTCTACAATCTCTGGGATGATGCAGAGCTTGAATAC
TTAAATCGCCATTTCAGCGCCGATATCATAGATGTAAATGATGTTATAGACGAAAAGGATATCAATA
GACTGATCCACATAAAACTTAGAGATATAACTATAGCTCACACCAAGACAATAACTCTAATGCTTA
AACTAGACGATATATTTAGAATTTCCTTTGCTTTTGAATTCTTTATTCTATCCATAGGTCTCATAGCT
GAATTACTTGGCGGCTTAGACAAAACTTACATGCAAGTTCCCTTTGCTATTATCCAAGTGGGAATG
GACTGTTTGACAGGACAACGCGTCATTGATGCATCAAACACTTTTGAAAATGCAGTGTACGCTTG
TAACTGGGAAAGGTTTGATTCGTCCAATATGAAGACGGTTTTGGTAATACTCATTAACGCCCAGAA
AACTTTATCATTATCTGCGGGTGGTATTGCTATCCTCAGCTTTGCAACTCTTATGTCTGTATTTAAGT
CAATTTATTCTGCCTTTACCACTTTACAGTCGATGATG

>CpunOR36

ATGTTGCGAACATATAGCTCGGCTAACAATGGCACGAGATATTACCATAAATTGTGCAACATTGTCT
ACCTTGCGGGATTGCCAAACTTCTGGATGGAAGATCTGGGCTACTCTGAAACATTCGTGAAATAT
TTTGACTATTTTACTAAACTCGTCACCGGTATCCTTTATATGTTCATGGTTTCCGAGTGGGCTGCAA
CATTCACGCAACACAATTTAACACCAAAGCAGAGTTCCGACAGCCTTCTCTTCAATTACGCCCAT
CCATTATTGTCCTCATACAGATTAATTATGATTTATCACCAAGATCAGTTCAGGGAACTGATATTTAA
TCTGTTTGTGAAACTGAAAGAGGTTTTCAATGAGAAAAATGTGGAACAGGAAATGATTAGAAAG
TCCAAAACGTTCGTCGTTTCGTTTACGATTATTTGCAGTATGACTCTCGTATTTTACGGTTTCGAAA
GCTTTTTGCAGGTGTTAACGACAGATGCCACATTCACCACGGTAATCACAGCCTGGCCAGACGTT
GAGGACAAGAGCACCGCTGCCGGTGTCGGCAGGGTCATCGCCTACCTGATCTGGTGGATGCTGA
TGTCACGAATCTTCGCGTCCTACATCGTCGTAATGTCACTGATGGTAGCCCTTGAGTATCAGTATA
AGAACTTAAGGGAATATTTCCGAAGTCTGCAAGAAATCTTTGAGGAAGATTTGAGCCAATCGGAG
ATGGAGATGAAGTATGAGGAAGGCGTCAAAATAGGGATTAGGCTGCATTCGGAGACTCTTAACTG
CGCCCGGCTAGCTCAAGAAACTTGCAGCACAATCTACTGCCTTCAAATTATATTGAACATAACCGT
TCTCGTATCGCTTATGTCTCAAATGCTGGACTCTGGTCGTACTGTGAGCACAGTGTTTACTATAGT
GGTCACCGCTGTCGGTACGCTCCTGAGCACCGGAGTCTTCATGTGGACAGCTGGTGACGTCACT
GTGGAGGCAGCATTGCTGGCGACCGATATGTACGCGTCCGGCTGGCAGAACTGCTACGAGCGCG
CCGTCAGCGTAAGGAAGCTGCTCACTGTCGCCATGGCGCAGGCGCAGAGACCCGTAACCATCAA
AGGTCTGGGTATTTTGGAAATTTCTTACAGCGCATATCTTCAGATTGTGAAGTCGTCATACAGTTT
GTTCACAATGTTGTAT

>CpunOR37

ATGTCCAAGGCCGGAGTGAGCGTCAGGCCGCACCTGCGCGTGCTGCGCCGCCTCGGCTTCTGCG
GCCTGCTGCCCGCGCAGCCCGGCGCGCCAGGCCGCGGGCCGCTCGCGCGACGCCTGCACAAGAT
CTACTGCTGGTTCACGCTCTGCGGCACCACGCTATATGTCATACAGCATCTCATTTGCATTTACCAG
GAGCGGCACAACGCGGCGCGCGTGGTGCGTGTGTTGTACCCGATGTTCAGCTTCTGCACGTGCG
TCTCCAAGCAGGTCTCGTTCCGCATGGGCCAGCCGCGCGTCGACGACCTCATCATTGGCCTCAAC
GATGAGTTGTTCAACCAAGAGGGCGAGAGGTACGCGCAGCTCCTGAAGCAGACTGCGCAGCGC
TCGTTCTACCTGATGCGGCTGTTCTACAGCTGCGGAGTGTCCACGGTCAGCCTGTGGACGCTGTT
CCCGGTGGTGCAGTACTTCCAGGGCAAGGAGGTGGAACTGCCGTTCTGGACTCCCCTGGATATTG
AGAAGCCTTCGATGTTTGCTATAGTAGTTGCGCACTCCTCCTACATGGTACTTCTGCTGTCCATGG
GGAACACCACTTCGGATGTCTTCATCGGCGCCATGCTCTACCAGTGCAGTACACAGCTGCGTATC
TTGAGGGCAAGCTTCGAAACTTTACCGGAACGAGCAAAAAAACTGTCGGAAGAGTTAAACGAA

AATTATGACATGATTTTACACGAACTGCTTGTGAAATGTATTCTGCACTTTCAGAAAATAGCTGAC
ATGGCGGAGGTAATCCTACAAGTGTATGGTTGGGCAGTGCTGGTGCTGTTCGGAGTGGGCGGCTG
GATGATGTGCACGGCGGCCTACACCTTCATCACTGTGGATATACTAAGCATGGAATTTGCTTCGGC
AGTGTTTTTCATCATATGCATTCTGATAGAATTATACATCTATTGCTACTTGGGAAATGAAGTTACAC
TCGAGAGCGACCTCGTGGTGGAGTCGATCTACTCGATGCACTGGCTCGGCACCTCGCACCGGTTC
CAGAAGGCGCTGGTGCTGGTGATGGAGCGCGCCAAGCGCCCGCTGAGGCCCACCGCCGTCGGC
CTCATACCGCTATCGCTCGACACCTACGTCAGGATTTTAAAGTCATCATACAGCATACTGTCGGTT
CTTCGTCAAACTAATAAA

>Cpun0R38

ATGGATTTGTTTGAACATGTGAAAAATACATTTTCGAACGTCAAAACTCGTTTAAAGCATAACACC
TATGCCGATTTGCTTTGGCTTGTAAATATTGTACCAAGTTTCGCTGGCTTTTCGATTCTGGGGGACA
CGCTATGGGCACCTTTTTGGATAGCGCATCTGTCGCTCCTGATGTATATCTACGGAGTGGGCTGTG
CAGTGTACCAAATCCGGGACGCCCAAAACACCGGAGATTTTATCAAGAGCTTTGTGAACGTTTCT
CTTTTTGTTCTTATCGCTAACAATAGTTATTGGTTCATGATGAAAAGACCCTTATTAAAATCTACCTT
GGCTGACATAAGTAAAAGTGATGAGATGGCAACTGTTAACGTTATTTTCCGTGAAAAACATGAAC
GTTACTTGATGAGGATCAAATGTATCTTGTTAATTTTTTATGGATTCAACGTAGGTAATGCCGCGTT
TGTTTACCTGCCGCATCGTGTTGACGTCTTGAATGACTATGCCATGACTCCTTGTTATGGTATGGAG
CCTTTATCGTCTAGTCCAAATAAAGAGATCTGCATGACTCTTCTCTGCCTGCAAGAGTTATCTATAA
TGACGGTTGTCCTCAATTATCAAGCTTTGCTCTTACTTCTGATAGCTCACACTGCTCTTTTATACCA
TCTGCTCTCCGACGAGATAATGACTCTCAATAATTTCAACAAAGACGAGCTTTACAATAACCCGG
CAGCTAAGGAATTTCTGCCTGTGATTATACATCGCCACTCAATAATACTTAGCGTTATATTTAAGTT
GAAGGCTCTATACAGCGTGCCTATAGGAGTCAACTTCGGCTCAAATGCTGTATGTATATCATTATTT
TTCTACCTACCCCTTCAGGAATGGTTGCAGTTCATGCCAATACTGGTATATTGCTTCTTGGTATTTT
TCTTGTACTGCTTCTTATGTCAGCGGCTATCGAATGCGGCAGAAGTGTTTGAGATGGCAGTGTATG
CTTGCGGATGGGAAAATTTTGATATAAATGAAAGAAAAGCGGTTTATGTGATGCTAAGACAAGCG
CAGAAACCTGTGGAGCTGCTAGCCGCAGATATAATACCAGTGAATATATCAACGTTTGCCACAACT
TTGCAGGCGATGTTTAAGTTTGTAACTGTAGTAAAGTTT

>CpunOR39

ATGGTTACCTTAAAACATTTTTGGAGGAAATTAACTCACACTAAGGCCCTAGAAAACTCTAGTGG
CCAATTGGAAATGGCGTTCTTTGAAACTGTTTATCGAGTTTCTTATATAACAGGTTTTTCAGCTGCC
GATCATGATATACCATACATGATTTATAGTACCACAGTAAAGTTACTGATAACGATGCTGGTCTGCG
CTGAGATGTGGTACGCCTTCACGGAGACGTCCAGCTTGGACGGTATCGCTGCGTGCATCAACACC
ACCCTCATACAGTTGATTACTATGTATCGATATGGGAAAATGTTATACCACAAAGATGTATACAGAA
AATTGGCGATGTCTATGGAGTCACCATACTTTGATATTTCGACGGAGCAGAGAAGGAATCTAGTCG
ATTACTGGGCTAAGAAGAATGATAACTACTTGAAGTTGTTATTGTTTCTGGGCAATTGTAGCCTAG
CTTTTTGGTTCGTTTACCCACTACTGGATGAGTTGGAGTACAACCTGTTCATAGCTATCCGGCTGC
CTTTCGATCACAGCAGCCCCAAAAACTACGTGTTGGCCTACTTCTTCGCAATCACCACCTTCGCTT
ACATGTCCCACTTTGTGATGGGAAATGATCTTCTGATGCAGGCCCATCTGCTTCATCTGGTCTGTC
AGTTCACCGTACTGAGCGATTGCTTTGAGAACATCGTGGAAGACTGTACAAAGGAATTCAAAGAT
GCTGATATTAATTCATTAATAAGAAATGAGATGTTCCAAAGAGTGTATAAAAACAGACTCCGTGAT
ATGGTCAACCAACATCGATCTATTCTTAGCCACGTCATGGAGCTAAGAGACGCGCTGAGCGGCCC
AATGCTAGGCCAGTTGGCGGCCAGCGGCATCCTTATTTGCTTTCTTGGTTATCAAACCGCTACGAC

CGGAGTAGGAAATGTTACCAAATTTTTGATGAGCCTGCTCTTTTTGGGATACAATCTGTTTGAGTT
CTACATTATATGCCGCTGGTGTGAGGAGATCACAGTTCAGAGTGAAAAGGTAGGCGAAGCCGCAT
ACTGTTCCAACTGGGAGTGTGGTTTGACGGTGATACCTGGAGTGAAGTCATGTCTTCTGCTCGTC
ATCGCACGGGCCACCAAGCCTGTGGTCATGACTGCTGGAGGGATGTACAACCTCTCACTTATGTC
TTATTCTTCGCTCGTAAAGGCCTCCTACAGTGCTCTAACTGTCCTTCTGCGAACTAGACAGGAC

>CpunOR40

ATGTCGGAATTAACTTTAGCAGAGGCAAAGCAGGAAATAGCAGAATCTTTGACTTTAAACACCTT
TTGCATGAACCGTATAGGGCTATCGTTCGAGGAACCAAAGAATACCACGGCATATTTGCTGCAGA
AATGTATGTTCGTGTTATCAGTGATGGGCATCTGCTACCACGTTTTCAGCGAAATCGTTTACATTGG
CCTGACCTTATCGAATTCCCCTCGTGTGGAAGATGTTGTGCCGTTATTTCATACTTTTGGCTATGGT
GCCCTTAGTATTGCAAAAGTATTCGTGCTTTGGTACAAAAAGGACGTGTTCAAACAACTGATTTAT
GAGCTGGCAGGAATCTGGCCCATGCCTCCTATGGCAGAGGAAGATACGATGATAAAAAACGACA
GTCTGGCCGCTCTTCGCATGGCTCATCGCTGGTATTTCGCTGTGAACGTGCTGGGAGTATGGTTCT
ACAACGTGACACCGATCATAGTGTACTTCTACCGGGTGTATCAGGGGCAAGACGTGACCGTGGGC
TTCGTGTGGGTCTCTTGGTACCCTTTCGATAAACACAAGCCTATTGCCCACGTTTTTGTTTATATCT
TTGAAATTTTTGCCGGTCAAACCTGCGTATGGATCATGGTAGGTACAGATCTCCTGTTCTCTGGGA
TGGCGAGCCATATAGGACTCTTACTGAAACTCCTACAGCGACGCTTGGAAACCTTGGCCTCTGCT
GAGCAAACTGAAGAACAGGATTATAGGGAGATATTGTCGAATATCAAACTACACCAGCGTCTCAT
AAGATACTGCAATGATTTGGAGGTCGCATACTCGCTGTCAAATCTAGTGAATATTGTTCTCAGTTC
TGTTAACATTTGCTGCGTCGTCTTCGTAATTGTTTTACTGGAACCATTCTTAGCTATCAGCAACAAA
CTGTTCCTTGGATCTGCATTGATTCAAATAGGAATGTTGTGTTGGTATGCTGATCAGATCTTGCATG
CGAACGCGGACGTGGCCGCAGCGGCATACAACAGCAGCTGGTACCGTACCAGCGCTCGCTGCCG
GCGCGCCCTGCTTTTCCTTATCCAGAGAGCCCAGAAGCCGATCGCATTCACAGCAATGGGCTTTA
CTGATATATCGCTCGTGACTTATTCGGCGATCCTGACCAGATCGTATTCTTATTTCGCTCTTCTATAC
ACTATGTACAATGACAAG

>CpunOR41

ATGGAACAAAGCGAAAGCCCCTTTGATTCCTTTCGTGTAATAATAAAATTGCTTACAATAACTGGC
TTTTATGAAATCGAATCAAGCCGCAAATATGTACGGGCTATCCACCAAGCCTACAGAATTTTTGTG
ATATTTATAATGTTAGCGTTTGCCATTCAACATTATATCTATACTTTTCAGGTAGCTGACCAAGATGA
CAAGCTGTATGGACTTATTATTGGATCACCACAAATCAATTTCATGGTGAAAATTTTAACCGTACAT
TTTAAATATGGAAGAATTCGAGAACTTCATGTATTAATCAAGGATCCTATTTTCTCTTCTACAAACA
AGAAAGATCTGGAATATATTCAACGGAACAATGTAGCCATGCAGATGCTGGGCAAAAGTGTTTAC
TACTGTATTACTTTAGCCGCCGTGACGTGGTCACTTGTATTTATATTTAAACGGATCGAAGACCCTG
TAAACGCTTCAGTACCATCTTATGTGCCTTTTGATACTACATCGACGATTGGATACTCAATATCGGT
TGTTCTTGAAGTGACACCAATCTTTTGGATAGGCTTTGGACAATCTGCGGCGGATTGCGCTGTAGC
CTGTTACTACTCGCAAGCTAGAACACAACTAAAGATCATAAAGCACAATTTGGAGCATATCTTTGA
CAATGAAGATGAAGAAAGTATTCTCATGGAACATGATACTAGCTCCAATGGTAGACCAAAATACAT
AGACGAAGTTAATGGCAATATAAAAACGAAGTTTATTTCCTTTATTGAGAGATATAAAATGGTGGA
CTGGTATGTAACTGAAATAAGCAGTATATTTGATACAAGTATAACCTGTCAGCTTATTTCGTCGACA
CTGGCGACTTGTTTGGTGTCTTACATGTTAAGTTTGTTGGAGATATTTTCTGTAATGTTTTTGCATT
TGTTTGTTTGTCTTATCTACTTTAAAATGCAAGCATTTATATTCTGCTTATTCGGACATCTAGTCGAG
GACGAGAGTAAGTCAATAAATGACGCTATGTACTTCAGCGACTGGTTGTCGGTGTCACCGCGGTT

CCGACGCTACATCATAATCTCGATGACCCGTTGGACGAAACCTTTAACGCCACGAGTTTCTACAAT
TGTTCCTATTAATTTTGTTACATTCGCATCGATAGTGAATTCATCATACAGATTGTACACTTTTATGA
AATCTTCTCATATTGCC

>CpunOR42

ATGAACACAATGTTTTTGTTTCTAACTAATACTGACTGTATTTACAAGTTAATTGTTTTTTGGGTGA
AACCTGAACAAATCGATGAGATTCTAAGTGTATTTAAAGGACCGATTTTTAATCAAGAAGAACCG
GAACATCGCAGTATTTTAATGAAAACAGTACGAAACTCTCATCTGATGTATGGAATATTAAAGAAC
ATGGCTTTATTTACTTGTTTCCTGTGGGTCGCCCACCCAACCGTGTTTCACATACAAGGGAAGTAC
ATAGAATTTCCCGTCTGGTTGCCATTCGATGCCAACAAAAATCCCCAATTTTACATAGCAGTATTCT
ATGTATGGCTACAAACATCTTGGCTGGCATACTGCAACACAACATCGGATATTTTCATCACGTTTTT
ACTTGAGCAATGTAGAACACAAATTTCTATTCTAAGATTAGATTTAGAAAACGTAGTCGAAAAGA
GCAAAAATGAAGCAACAGCGTTATCAATGAGTTTCCAAAAGGTTTTAGAGAGACGATTCAAGAA
AATCTTGTTACATCACAAAACGATCGTCAATACGGCAGACAAAATTCAAGACATATTTGGTGGAG
CTGAGTTTTATTTGTTCTTCGTAAGTGGATGGATTCTATGTACTTCTGCGTATCGATTAGCTAGTGC
CAATCCAGCTTCAATTGAGTTTGTGTCAATGATAATGTATGTGATTTGTATCCTGATGGAGATATTT
CTCTATTGCTATTACGGCAACGAAATAACACACGAGACTGATATGCTGATGGAGTCTGCCTACGCA
ATGGACTGGTTAGAAATGCCTGTAAAACTGCGTTTGTCTTTGATCATATTCATGGAACGCATAAAA
ATACCAATTAGACCGATGGCGGGATCCCTGATACCGCTTACTAATGCCACGTTTATTTCGATTGTAC
GCAGCTCTTACACATTTTTTTCGTTATTAAAAAACTCACAACAT

>CpunOR43

ATGAAGAGGAAACTGGATCCTATGTCGTTAAATCACTTGAGATGGGTACGTATGGCGCTGAAGAC
GGTGGGCGGGTGGCCGGGCCACGCCATCGACGGGCGTCCCCACAAAGTGCTGTGCTTCACGCTC
TTTGTGGCCATCGAGTGTGCGTTGGTCATCGTCGGAGAGATCTTGTTTATAATTAACCGTTTCCAC
GTGGTTTCTTTCTTTATTCTAGGAGACGTCTATATTTCAATGGCTCTATCTTTCGTACTTTTAGTAAG
AGCTTCTATACCCATTTTTGAAAGATATGGAATTATAATGAGAGAATTTATACGTCACTTTCATTTGA
TTCATTTCAAATATACAGGTGGACATTGGGAAATAATTTTTGAAAAAATAAACAAACTATCCCATT
ACTTTGCTTTGTTCAATATTACATTGACAGCGATAACCGCAATAAGCTTTAACATCCCTCCGCTATA
CAACAGCTACACTAGAGGGGCGTTTAAGAAGAATAGGTCGGAAAACATAACATTACAGTTTTCTG
TACATTATGACTGGCCAGGCTTTGAACAAGAGAAACACTTCATAGTAGCAAGCATCTTAAACTTTT
GGTTAAGTTACGCCTGTGCCTTTATTATATGTATTATGGATTTGCTTTTATGTCTCATGGTGTTTCAA
ATAATTGGTCACATCCAAGTTCTAAAGCATTCTTTGAGAAACTTCCCTAAGCCTCAGATACAGACT
AATCTACAAGAATTATCGACGGGGGAGATGAATGAAACACGCATCCTTATAGAAGTCATGCAGCC
CTTCAGTGGTGAAGAGAATGAATGCATTGAAAATAAAATTAAGGAGTGTGTTGATCATCATTTGTT
TATTGTAAGTTTTGCAGAGGACATGTCGCAGTTTTTCGGGCCTTTGCTTGCTGTTAACTATTCATAC
CATCTGTTCGGGCTAAGTCTGCTCCTGGTCGAGTGTATGCAAGGGGAGGAAGGTGCATATACTCG
CTACGGGCCTCTTACACTAATAACAATTGCTCAACTCATGTTGTTGTCTATCACATTTGAAATAGTT
GCATCTGAGAGTGAAAAATTAATCAACGAAGTGTACTATGTCCCTTGGGAGTACATGAGCGTCAG
TAACCAGAAGTCAATGTGCATTCTGCTCGGGCGAGTCCAGAGGCCTATCCACGTGACTGCGATGG
GGATGGCGGATGTGGGTGTTCAAACCATGGGTCAGATTTTGAAGACTACGTTGTCATATTATGCGT
TTCTTAGAACACTAAATAAT

>CpunOR44

ATGAGCTTGGATATGTTGAAACGAAGCAACGTCATGTTCTGCGGACCCGAAGTATCCTTTACGAA
GAAGTACTGGCGTTTCTTCTATATGATACCTCTACTGGTTTTGCACTACATAAGTCTGTCGGCATAT
ATTTTAAAGCTGTTTACTGAGGGACTTGATCCTTTTGAGAAAGCTGACATGCTTCCTTTGTGGCTT
GCTACTGTAGAATATTGGATTAATACTGTTATTCTAATAATAAATCAGGACGAGATAAGGAACTTCA
TCGTCCACTTGGGTTCTATTTGGAGGATTACAGGACTTAATGAAAAGCAGATGACTATAAAAACT
GGTATACTCAAACGTCTCTACTACGTAGGAATCGCTGTTAATAAGGTCGGCCTAGCAATGTCTTGG
CAATACCTACTAGCGCCTCTCTGTGAGACGGTGATACGAAGGTTGTTCTTGAAACAAGAGGTGGA
ACTGCAGCTACCCTTCGACTGTGTCTACCCTTTCGAGACGAAGGACTGGCCTATTTATCTAGCGGT
GTACGCCTTTCAAGTGTATTGCATCTTTCGCACCATCTACGTATATCAGGGCGTTGGATGGCTTCTA
GTTGTTTTAACTACACACATGCACCTCCAATTCTTACTTCTTCAAGAAGATCTGGTGCAAGTTAAG
CCTGAAATCAAAGGTCGCCCGATTAAATCCGTGTCCGAAGATGCGGATGAGATAACGCAATATGT
TGACAGACCTTATTTACGTATAGACGACTTTGTGAGACGTCATCAAGTTCTTATATCATTGGCAGAT
AAATTGGACTCATCGTGCAACAAACTGACATTCACAATCATGCTGTTTGCAACACTCATCATTTGC
TTCTTCGCTGTAGCAGCAAAAGCATCAAAGGGAGCTGCTTATGCTTTGAACAACTACGGTGCAGT
TGTGGTGATACTGATGAGTATATTGATACTATGCTACTGCTGTCAATTACTGAGCTCTTCTAGTAGC
GGAATTGCAATGGCAGCAGCTAAAAATTTATGGTACAAAGGAGACCTGCGGTATCAAACCAATAT
TCGTTTCATAATGATGAGATCACTGAAACCATGTTCTTTGACATTATTGGGATTTTCACCAATTGAT
TTAGGGACTTTCAATAAGGTTCTAAAAACTACATGGTCATATTTTTCTCTGGCAAGTCAGATGTAT
GACGAACGTGAT

>CpunOR45

ATGACCGTTAAAATGCAGAAAGGACAGCCCTTACTATTAACTCATATACGAATTCTGCGGCTGTCA
CTTATGTCCTGCGGGGCGTGGCCAAATGAAGTTTTGGAAGGGAGTTCTCGTAGAAGATTCAGCTT
GGCATTTATAATAATCATAATATCATGCATAAATACTTGCGGCGAACTTAACTACCTTATAAAGAATT
ACAACATATTGCCATTTTTCGACTTGGGCGACTTATACATGACATTTTTTTAACCATCTTGACTTT
AATTAGAGCGATTATACCAACCGTGAAAAGTTATGGAGATGTAGTATGCACCTTTTTTATGGAATTT
CATTTAGAACATTATAAACAAAAAGGGGAATACTACGAAGAGATTTGCAATAAAATAAATAAGTTT
TCTCACTACTTCACGTTGTTAACGGTAGCGAATATGACATTAGGGCCAATATTTTTTAATCTGGCCC
CCTTATACAACAATTATAGGCACGGCGCTTTCACCAATAACAGAGAAGAAAATTACACACTTCAG
TTTTCTGTATACTTTCTTTATCCTTGGTATGACCAGGAAGATCATTTTATCACAACATCACTTATAAA
TCTGTATTTATCATACTTCTGTGCACTTTTAGTGTGTTGCCTTGATTTACTTATGTCGCTAATGGCAT
TTCAAATAATTGGCCATATAAAGACACTTATATACGACCTTCGACATGTGCCTAGGCCTAAGCGCC
ATATCTCTATGGACATATCGTTCCGTGAAACAAACACTAAGGCAAATATTTATGCTGATATATACGA
TGAACAGGAGAATATTGTGATTCGGGCGAAAATTATAGACTTTGTCAACCATCACAGGCAAATTG
TAAATTTTGCAGGAAGTATGTCAGAACTATTTGGACCGATGCTAGCTTTTACATATCTTTACCAGTT
AATCAGCTGTGCTCTCTTACTTTTGGAATGTTCTCAAGGCGAACCTGCCGCGTTAGCTCGATACGG
ACCTCTTACAGTAATAATATTTTACCAACTTGCACAGATATCATTTACATTTGAATTTATTGGATCGG
AGAGCGATAAATTAAAAGACGAGACATATGACATACCTTGGGAGTGTATGAGCGTCAAAAATCAA
CGATTGGTCTGGATGCTCCTTAACCGCATACAAATCCCCATACGAGTGACTGCTTTGGGAATGGTC
GAAGTGGGCGTTCAAACCATGGTCGCGATATTGAAGACAACGTTTTCATATTTTGCTCTTCTTAAA
TCAATTAATGAG

>CpunOR46

ATGACGATACAAGGCAAGAAGCAATGGGAATTCACATCGTTCCCCGAAACGTTTTTGATAACCAG

TTTCTCGATGAGTATCGGTATGATATATCCTAACCCCGCTACTGACAAGTGGCGGTTGCTGGCTATA
CCATTTGCACTGTTGTCTGTTAGTCCTGTATCAGTTTTTATTTCTATTGAGATGTTGAAACTATGGA
ACAACGGAGAAATTCTTGAACTTATGAGACATACTGCTATGTTCCTTCCATTTTTTGCGGGGTTTAT
GAAGATGTGCCTAATGTACTACAGAAGAGTGGAAGCGAAAAAAATCATTGACATAATCAACCATG
ACTACGCGTATTACAACAACTTACCAGACGACTATAAGGCCATGGTGGCTGCCAGTATCAAGAGC
TCCCACATCTACATCAACATCTGGGCTGCTTGCGTGATGGTCATCTTCGCTGTCTTCGTGGGCACT
GCCAGCTTACTCAACATCGTCAGCCATCTGTTCATGCAAGAGCCGAAGAGGTATATGATTTATGAC
ATCAATATGCCGGGGAGGGACCCAGAGGAGCGGTTCAACTCGCCGTATTTTGAGATCATGTACTT
CTACACAATTTACGTGGCGCTGATGTACGTACTAAGCTTCACGGGCTACGACGGGCTGATGATCG
CGTCGGTCAACCACGCCTGTCTGCGGCAGAACATCTTCACGAGGCAGGTGCAGGAGGCGATGGG
CTTGAGGGGGGAGCGGCTACGGAGGAAAATGGCCGCCGCTGTCAAGGATCAGGTTGAAGCTTTG
AGATTAATAGACTATGTTCAAACTACATTTAACATTTACTTGGGATATATGTACGTGATTGTATTAGT
TGAAATGGTGATATGTATATATCTTACTAATGAGGACTACAACTTCGATTTCCAGTATACTTGCCTC
AGTTTCGGCACCATTCTGCACATATACGTGCCTTGCATGATCGCTGACAAGCTCAAGTGCACGTG
CGAAGACACCGCAGTGAGGCTGTACTGCTGCGGCTGGGAGAACATGTACGACCTGCGCGTGCGC
CGGTGCCTCCCCTTCATGATCGCGCGGGCGCAGTGCCCCGTCACGCTGAAGGCGTTCGGCTTACT
CACCTATGAGATGGATTTGTTCGTCGGGACTATGAAGAGCGCCTATTCTATGTACACGCTGCTTAA
AACACAACAA

>CpunIR1

ATGAAGAAAATAATATTGTTATGTGTGTTGTTGTGTGCTAACACTTTAGCAGACTTAACCCCTATCC
TGAAAGGTCTGAACGAGCGTAAGGACCTGGAAAAGATTCTAGTTGACCTGCTAAATGAGATGAC
TGGATCAGACGACGAGATCACCTGCATGACCTTCGTGTGCGACGCGGTGTATCTGAACGTGTTCG
ACGGAGACCTGTTCAAGAGGACCATGGCTGTGCCTATTGTCATGATTGTGGTAGAAGATTACGAA
GACCTGCTATCTCCAAACTTTGATACGCTGGAATCTTTGAGAGAGGCAAGGAAAGATGGTTGCAA
CGTATACGTTATTCTTCTAGCAAACGGTCTGCAAACTTCTAGATTGTTGCGTTTCGGAGACAGATA
CCGGATCCTTGACACGCGGGCTAAATACATAATGCTGCACGACTACAGACTGTTCCACAGCGACC
TGCATTACCTGTGGAAACGCATCATCAACGTGCTCTTCTTGCGGTACCACAGCATATCTGTGGGAG
CGCCTAAGAGCAAGGCCTGGTTCGATCTCTCAACTGTGCCGTTTCCGAATCCTATCAAGAGCGTT
TTTGTGTCTAGAAGAGTGGACATCTGGAACAACGGGAGGTTTCACTACAACAGAACATTGTTCGC
GGACAAGACTAGCAACTTGAACAACGAAGTCTTGAACGTGGTGTATCTGGACCACGTACCGTCC
GTGGTGGTCACGAAAACGAACGAGACGAATAAAGTTGGCGGCGTGGAGATCGAAATTCTCAACA
CCCTGGCTAAACAAATGAACTTTCAACCGAAGTTGTATCAGCCAATCAACGCGGATCTATACAAA
TGGGGTCAAGTGCAGGCCAATGGCTCCTTCTCCGGACTTCTGGGAGAGATGGTGAATGGCCAGG
CCGACGTCGCGCTCGGCAACCTGCAGTACAACCCTTACCACCTGGAGCTAACTGACCTGAGCATC
CCTTACACGTCACAGTGCTTCACCTTCCTGACTCCGGAGGCTTTGACAGACAACTCTTGGAAAAC
TTTGATTTTACCTTTCAAAGTCCGAGAAGCCGGCGGGCCTGTATCTGTTTGGCGAGATAATAAACA
GCATTCTCTACACTTACGGAATGCTGCTCGTCGTGTCTTTACCCAAGCTACCCACAGGATGGTCCC
TGAGACTTCTAACTGGGTGGTATTGGTTATACTGCATTCTGGTCGTCGTATCCTACAGAGCCAGCA
TGACCGCTATTCTCGCAAACCCTGCCCCCAGAGTCACAAT

>CpunIR2

ATGGAGATTCCATTGTTTTTGTTAATTTTCTTCATTAATTTGGGCTGTGTCGTTTCGGAGCTGTCTC
TGAGGTTTGTGTTCATAATCGAGAACCACGAGCCGGAGCTGGGTCAGTTGGTGGGGAGGGCGCT

GAAGGTGGCCGAGGAGCAGCAGGACGTGAGGGTCGATGACTCCATCGTACTACTGGACCGGGA
GAACGAGGAGGAGAGCTATGGGAGATTCTGCTCCGCAATCTCTAAAGGGGTGTCCCTCATCGTG
GACCTCTCCTGGTCTCCGTGGGAGCTGGCCGAGAGCGTGGCCAGTGGGGGCGGAGTACCCTTAG
TTCGGACTGCTCTGAGCATGCAGAGACTCCTTTCAGCTGTCGCCAGCCACTGCGCCTCGAGGAAT
GCGACTGATGCCGCGTTGATTACTGAGAGCGAAGCGGACGTCGACAGAGCTCTATACGAGCTGCT
AGGTCGCTCCAACATCCGTCTCTGGGTCCACGCAGGGCTCACGAGAGACTCGGCCCGCGCCCTC
AAGAACATGCGACCAGACCCAAGCTTTTATATCATCGTTGGGGAGAGTGGTTTTGTCATGGATACT
TATAGACGGGCAGTTAAGGAGAAGTTAGTCCGCCGAGACTTCCGTTGGTATCTGCTCCTGACAGA
CTACTCCGGGGACAGTTTTGACACCAGTCAACTTGTACTGCCGACTATGATGTTACACGTGGACG
CCAACGAGTGTTGTAAGCTGCTGGGCTCGAAAGATGACTGTAGCTGCCCTTCTGATCTGAAGAG
AAAGCAATACATTTTGAGCGGCCTCATGACATATCTAGCGGAGACTTACTCCAAATTAGAGAATGA
TTTAAGCGTCGTGACGGCTAAAATGGACTGTGACAATATACAGGCTTCAGAAATGAACGTGACAA
GAGAGAGGGTCGTGAGACAGTTCGCTGATGATGAGCAGATCAGCAATGACACACTGTTCTATTG
GGACGGTGAAAGGTCAGCTCTATATCTTCGTTCCACCTTCGTCCTCTCCACCTTCAAGCCTGACTC
CGGGCTGGAGACGGTAGCGAGCTGGTCTGCCAACGAGGAGTATAAGCTGTTGCCAGGAGTCACC
TTGGAGCCTCTGAGACCTTTCTTCAGGGTCGGAACCTCTCCGGCCGTACCCTGGACCCTGCCCAA
GCTGGATCCCGACACGGGGGAGCAGGTCTATAACGAGGATGGCCAGCCGGAGTACGAGGGGTAC
TGTATAGACCTCATCGCAAGGATTGCCGAGACTATGGAATTTGACTACGAGATAATAACGCCTAAG
TCTGGTACGTTCGGCAAGAAACTACCCAACGGATCCTGGGACGGCGTGGTCGGCGATCTGATGC
GAGGGGAGACGGACTTGGCAGTGGCTGCGTTGACGATGACAGCTGAGAGGGAAGAGGTGATCG
ACTTCGTGGCTCCTTACTTTGAACAGACTGGTATACTCATTGCCATCCGCAAGCCAATCCGGAAG
ACCTCCCTCTTCAAGTTCATGACCGTCCTCCGGACTGAGGTCTGGCTGAGTATCGTGGCCGCGCT
GGTCCTCACTGGCCTCATGATCTGGCTCCTGGACAAATACTCGCCATACTCGGCGAGGAACAACC
CTACTGCTTACCCGTATCCTTGCAGAGAGTTCACGCTAAAGGAGAGCTTCTGGTTCGCCCTAACC
TCGTTCACCCCCCAAGGTGGGGGGGAGGCGCCCAAGGCCCTCTCGGGGCGGACGCTGGTGGCG
GCCTACTGGCTTTTCGTGGTGCTCATGCTGGCCACCTTCACAGCCAACCTGGCTGCCTTCCTGAC
CGTCGAGAGGATGCAGACGCCAGTATCCTCCCTGGAGCAGCTCGCCCGTCAGTCCAGGATCAAC
TACACCGTAGTGGAAGGGTCCACGATACATCAGTACTTCATCAACATGAAGTTCGCTGAAGATAC
CTTGTATAGAGTCTGGAAAGAGATAACCCTGAACGCAACATCAGACCAGGCTCAGTATCGAGTAT
GGGACTACCCAATTCGGGAGCAGTATGGCCACATACTATTGGCTATCAACGCATCAGGTCCAGTTC
CTGATGCCAAGACCGGGTTTGAGCAAGTCAACGAGCATACCGATGCAGACTTCGCTTTTATACAC
GACTCTGCTGAAATAAAGTACGAGGTGACTCGTAACTGCAATCTGACGGAGGTCGGCGAGGTGT
TCGCTGAGCAGCCCTACGCGCTGGCCGTGCAGCAAGGGTCCAGGCTGCAGGAACAGCTGTCCAG
AGCCTTGTTGGACCTGCAGAAGGAGAGGTTCTTGGAGCAGTTGACATCCAAATATTGGAACGAG
TCTGCGCGACAAGCGTGCCCGGACGCAGACGAGTCGGAAGGCATCACTCTGGAGAGTTTAGGTG
GAGTTTTTATAGCAACTCTCTTTGGTCTAGGTCTAGCAATGATAACTCTAGCCTGGGAAGTGTTCT
ACTACAAACGAAAAGAGAAAAACAAAGTGCAAACCATAGACGCAAAAATGGAGAAAGCCGCTT
TTACCGAACCGAAAAATGCGGAAAAAACCGGTGTGCGGTTTAGGAAGAAGGAGAAGAAAAGTA
AATTGGGTAAAATTAGTAAGTTGGGTAAAGTGGAGGAGGGTAAATTGGGTAAAAGGGTGACTATT
GGGGACAGTTTCAAGCCGGCTTCTGAGGGAGCTGGAGTTTCATATATAAGTGTTTTTCCTAAGGG
GGAGTATAGACCC

>CpunIR3

ATGATAAATATATTGCGCATAATATTCACAATACAAATAGATTGGGTTAATAAATTTATTCTGTTTTTT

AAGTATAAGAACTTTATTAGTTATTTATTTACAATGATGTTGGACTCAGTGTTATCGTTAATGCCAAT
AGAAATATTGCTCCAAACAATATTCAATGAATATTTAAGTAACTCCTATTGTTTGACTGTCGTGTCG
GAAAAACCACTCGATTTGCATGTTAATATTAGTTATGCATACATTTCTGTAGAAAACGGTGAATTAT
CTCCAGATCAGATGTTAAAGCTTTCTGAAAATGGATGTTCAGATTATATTGTGCAAGTCAAAAATC
CTCAAAAATTCATGGGAGCTTTTGAAACAGTTAATCTTTTAGGAAACGTAAGACGTGGTGATCGA
AAAATTGTGTTTTTGCCATATCGAGAAGATAATGCTACCACAACTCTTTTGCTGGAGATACTAACA
TTGAAGGAGACTAGTTTCATTGCAAATATACTTTTGATATTGCCGTCGCCAGAACAATCGACGTGT
AGCTATTATGACTTAGTTACGCACAAATATGTTGGTCAAGACAATGAAATCAATCAACCTTATTATA
TAGACAGGTGGAACGCATGTACTTTAAATTTTGAGAAGAATGTTTCTTTGTTTCCTCACGATATGA
CAAACTTGTATGGTAAAACTTTAAAAGTAGCTTGTTTCACCTACATGCCATATGTATTATTAGATCT
TTCTGAAGCACAAGAACCGTTTGGAAGAACTGGTACGGAAATAAAGATTGTTGATGAATTTTGCA
GATGGGTCAACTGTACCGTTGAATTGGTACGTGAGGATGAGCATATGTGGGGAGAGATATATGATA
ATTTAACCGGAGTTGGTGTGATTGGAAATTTAGTGGAGGATCGTGCTGATATTGGTATAACTGCTT
TATATTCATGGTATGAAGAATATGTTGTATTAGACTTCTCGGCTCCTGGGGTTAGAACTGCTGTCAC
ATGCATAGCGCCATCGCCAAGGTTATTGGCCAGTTGGGAAGAGCCACTGCTCCCTTTTAGCTGGT
ACATGTGGCTGGCGTTGATTTTCACATTCGTGTACGCTAGTCTAGCCCTTACGATAGCCCAAGGCT
TTACAACAGACAACGCATTCTTGACCACTTTTGGGATTATGATCGCACAATCTCAGCACGATGTTG
GCGCATCATGGCGCGTGCGAAGCGTGACGGGCTGGATGCTGTTGACAGGGCTCGTCATAGGTAAT
GCCTACGGTGGAGGCCTAGCCTCCGTGTTTACTGTCCCGAAGTATGAGAAGTCTATAGATACTGTT
CAGGATATTGTGGATAGAAAAATGGAATGGGGAGCTACTCACGATGCCTGGGTGTTTTCACTTAC
ATCATCAAATGAGCCCTTAATCAAGAAATTAGTAAACCAATTCAAAGTGTATCCAGCAGACGTTCT
GAAGAAGAAAAGTCTTGATCGATCTATGGCTTTTTCAATAGAACGACTACCTTCAGGTTATTACGC
AATTGGTGATTACATAACTAAAGAAGCGATGCTGGATCTGACCGTAATGCTAGAAGACTTTTATTT
CGAACAATGTGTGGCGATGCTGCGTAAGAGTTCACCCTACACGAAAAAGATAAGTCAGCTAATAG
GGCGATTGCATGAATCCGGATTGCTATTAGTGTGGGAGACACAGATGGCGTTAAAATATTTGAACT
ACGAAGTACAGCTAGAAGTTCGATTATCACGATCCCAAAAGGACATAAATACGGAAGCGTTAAGT
CTTCGACATGTTGTGGGAGTCTTCATACTGTATTTGATAGGAATGATTTTTTCAGTCATTATTTTTAC
TTTGGAAGTAATGAATGTAAACAAAAAAAGAAACACTTCATCTTTT

>CpunIR4

ATGTTAAATAAAATGCAAGATATGAAAACCTACGAAGTGTTATTTATTTTGTTAATGAGTTGTTTCT
CCCTGATAATTGCCAATCCAATCAATGAATTCAGAATGATAGCAGATGTAATAAAAGATTCGAATA
AGTCTACTTCAGTAGTTGCACACCTTTGTTGGAATCCGTCAAAGCAAATCCAAATGGCGTCTTATT
TGCATAACTCGGAGTTAACACAGCTAGTTCTTCTTGTGAATGAATCCTGGGCAGACATAAAGGAA
CCACAACATAGGGAACGATTGTTACTAATAGCAGACATTGACTGTCCATCTACAACAGCTTTCTTT
AAAATGGCTAATGAAACTAAAAAGTTTAGTTTGCCATATCGCTGGCTGATCATCGGAAAGGCAGT
TAATAAGAGCACAGACGTGACTGCTGATTTTGATGGCTTGCATTTGTTACCGGACTCCGATGTAAT
CATCGCTCAGAAGAACGACAGTAACTCATTCTATATGAACATGATTTACAAAATAAAAATAAAAA
GCAAATGGATAATTGAAGATTTTGGAACTTGGACGACAAATACCGGCCTTATAAAATCTGATCTAG
CGCAATACTCAACCTCGACCAGAAGAAAGAATTTCCATGGAGAATCATTTACTACAGCAATGGTT
ATATTTGACAACAAGACTATATCTAATCTGTTTGATTTGAGTGATATACTAACTGACGTCGTAACTA
AGAGTTCTTTTCGACAAATCGTGCCACTTTACGGTTACATGAATGCGTCTCAACAACACATATATT
CTAAAACTTGGGGTTACTACAGAAATGGCACTTTTGATGGAATGATAGCTGAATTGACTGTAGGC
GACGCGGACCTTGGAGGAACAGTGTTAATAGTCACATGGGATCGTATGCAAGTAGTTGATTACCT

TTCAAAACCCGGTTCTATAACTGTCAAGTTCGTATTTCGGGAGCCACCACTCTCATACCAAAACA
ACCTGTATTTACTACCTTTCAAAGTGACGGTTTGGTACTGTATGGGAGCGTTCGTTCTTGTGATGG
GTTTCATCTTGTACATCACTGCGCTATGGGAAAACAAGAAAATGGGGGAAAATCAAGAGATCTCA
AATGATCCTACTGTCTTGAAACCCAATGTAAGCGATATTGCGATTTTAATAATTAGCGCAGTATCTC
AGCAAGGAACTACGTTGGAATTAAAAGGAACTCTTGGTCGGATTGTAATGATAATACAGTTCATT
GCGTTCCTGTTGCTTTATGCATCATATTCTGCCAGCATAGTGGCGCTACTTCAGTCCAGCTCCAAC
CAAATAAGAACCTTTTCAGACTTACTAAATTCCAAGTTAGAGCTAGGAATCGAAGACACGCCGTA
CAACCGATATTTCTTCCCGATAGCCGTAGAACAGGTTAAGAAGGAAATCTACAAATCAAAGACAC
CACCACGTTGGACAGAGCCCAAATTCATGAGTCTAGAAGACGGAGTGAAGAAACTACAAAAGA
AACCATTCGCCTTTAACATGTTGCAAGGTATTGGGTACAAGCTAGTGGAGCGATATTTCCATGAAC
ATGAAAAATGCGGTCTCCAAGAAATAGAATTACAGTATGGAACCAAAACATACATAGCCAGTCGA
AAGAATTCACCTTACAAAGAGATTTTCAAAATTGGCTTATTCAGGATCCAGGAACACGGGATCAG
CGACCGCGAGTTCCGGCTCCTCTACGCGCGCAAGCCCACGTGCCAGGTTCGCGGCGGCAACTTC
GACTCTGTCAACATGGTTGACTTCCATCCCGTGCTCCTCATGTACCTCTACGGCATATTACTCGCTA
TTGCTCTTCTTGTCATAGAGATTCTCGTGTTTAAGAAACAGCAGCTCATGTGTAGTGCTGCGAGTA
GGCGGCAAAGAAGCGGGAGCTGT

>CpunIR5

ATGAAAGCGACTGTATTGCTTTTGTTCTGTTTAAAATATCTTAATGTTAAAAGCCATGCGAATACTA
ATTCTGTAATGCATATGGTGGGCGATATAATTCGCGCCATGGAAAAGCCGTCAAGCGTAGTGGCCA
CGCTTTGTTGGCTAACTGATGAAAAAGTGCAGTTTTATTATGCAGTTACGGCTAGTGATAGGTTTT
CGAGAGTCAACACTGCGCAGTTTGTTGATATGCGTCATGTGAGCGAGGATCATGGTCAGGAGCAA
CATATTGTATTTGTAGCAGATCTCAGTTGTCCAAACATTTCCGCGTATTTTGACGAGAAAAGAGCA
CAGAATTACTTTCGTGGACCTTTCCGTTGGATTTTAATTGGTAATGTGGTAGAAGAAGATATAGTA
CCGAACTCAATAGCTCATATTGATGCTCTGCCGGATTCTCAAGTAATCGTCGCACGACAGATTGAT
GAGGAATCCTATGATCTATATACGATTTATAAAATAAATGCCAACGATGATTGGCGAACGAAACTAT
ATGGAAAGTGGAACCAACAAACAAGATTTACTATTACAAACCCTCACATGGAATCAATTGCTTTG
GAAAGGCTAGACTTGCTTGGATCCGAAATCAGTGTTTGCTACGTATTAACTGATAAAGACAGTATC
AATCATCTTACTGACGAAGTAAATGACCACATTGACACCATTACAAAAGTAAATTTTCCTACAACA
AACCATTTGTTGGATATTGTAAATGCTTCAAGAAAATATATTTTCGCCGATACATGGGGTTATCGAG
TTAATGGGACTTGGAATGGTATGACGGGATATCTCATAAGAGAAGAAGTCGAAATAGGAGGTTCA
CCCATGTTTTTTACTTCAGAAAGAATATCCGTTGTGGACTACATCGCAAGTCCTACACCAACTCGT
TCAAAATTTGTATTCCAACAACCTAAATTGTCATACGAGAACAATCTTTTCCTTCTGTCTTTCCGCA
CTTCTGTTTGGTACAGTAGTACGGGCCTAATTTTCTTATTATTATTGGCTCTATTTGTAGTGGCAGCT
TGGGAGTGGAAAAAGCATACAAATGATAATCAGGTTTCTTTTCAGAGAGAAAATGATGCAGGGA
CTCTTAGACCAAATTTTGTTGACGTTATAGTTTTAATATTTGGTGCTATCTGTCAACAAGGAAGCCC
AGTGGAACTAAAAGGATCACTAGGACGAGTAGTGATGCTCATCCTCTTCTTGGCACTGATGTTTC
TTTACACCTCGTACTCAGCTAATATAGTCGCTTTGCTGCAGTCGAGTTCCACTAAAATCCGCAATT
TGGATGACTTGCTACATTCCAGACTAAAATTTGGTGTCCACGATACAGTCTTCAACAGATACTACT
TTTCGACAGCTACAGAGCCAGTAAGGAAAGCTATCTACGAAAAGAAAGTGGCTCCACCGGGAAC
TACTCCCCGCTTTATATCAATGGAAGAAGGAGTAAAGAAAATGAGGAAGGGTTTGTTCGCATTTC
ACATGGAAACAGGAGTTGGATACAAGTTTGTGGGCAAATATTTCGATGAAGGTGAAAAAATGTGGA
TTGCAAGAGATACAGTATCTGCAAGTTATAGACCCCTGGCTGGCAGTTAGGAAGCACACGCCTTA
CAAAGAAATGTTCAAAATAGGAATGAAACGCATCCAAGAACATGGCCTTCAATCCAGGGAGAAC

TTGTTGTTATACGAAAAGCGTCCGAAATGCTCTGGTAGAGAGTCCAATTTCGTATCAGTCAGCATG
GTCGACTGCTATCCTGCGCTCTTGATCCTGTCCTATGGAATACTCGTGGCTTTGTTCTTTTTAGCTT
TCGAATTGTTAATTCACAAAAGACAAACTATTGTTCATAGATTATCGCATTGTCGGCGTAATAGCAT
TGATAGTAGGTTCATA

>CpunIR6

ATGTCTCACAGTATGATACATCTACGATGTATTGTCGCTTATACGTTGCTTTTGTATTACACTGTGTG
GTGTGAAGATGTGGAATACTATCCTTCACAGTATACTATTGATAATCACAATATTGCTAAACGGTCA
AGCAATTATTTGCAAAGTACCGACAACAAATGGAATATTAAGGAAAAAGTGACTCAAATGAAATT
AAGATATTTCAATGATAATGATATAAAAGCCAAAGATAATAAAACTAAAAGAGCAGTTGATCCAGT
ATTCCATGGACATCCTAAAACCAGAGAACAATTGTGGCATGAACATTTTCTAAATAAGAGTACTGC
GTTCGACCAAAATCCTTCGCTAATAAAACTAATTCACAAAATAACGTTAAAATATCTTAATGATTGT
ATACCAGTAATACTCTACGACAGCCAAGTAAAGTCAAAGGAGAGTTATTTATTTCAAAATCTCCTA
AAAGATTTTCCAGTGTCATATGTGCACGGATATATTGATGATAGCAACAATCTAAAAGAGCCAGAA
CTTCTTGTACCGGTTAAGCAATGTCTTCATTATATAATATTTTCGACAGAGGTCAAAAGTAGTGCGA
AAGTACTTGGTAAGCAATCGGAAAGTAAAGTTGTTGTTGTAGCTAGATCTTCTCAATGGGCTGTG
CAAGAATTCTTAGCAAGTCCTGAATCTAGGATGTTCATTAATTTGCTGGTCATTGGGCAGAGCTTT
AAGGATGATGACGATGAAACAATGGAAGCGCCATACATTTTGTATACGCACAAATTGTATACTGAT
GGATTGGGCGCCAGCAAGCCTGTAGTATTGACATCATGGACACATGGAAAATACTCTCGTGAGGT
CAACCTATTTCCAGCTAAGATGACTGAAGGTTATGCGGGACATAGATTTATAGTATCAGCATCTAA
CCAACCGCCTTTTGTTTTTAGAAGAATAAAATCAGATTTAGACGGAGGAAATCCACGAGTTGTGT
GGGATGGAGTTGAACTGAGATTGCTTAGCATGTTGGCAGAGAGAAATAATTTTTCAATCGAAATC
AAAGAGCCACAGGAGCCTTCTCTTGGTCCTGGTGACGCCGTATCGAAAGAAGTAGCCATGGGTA
GAGCAGATATTGGAGTCGCAGGGATGTATTTTACTAGTGAAAGAACCTATGGTTTAGACATGTCGT
TTTCCCATTCACAAGATTGTGCTGTGTTTATAACATTAATGTCCACTGCGCTACCACGATACCGTGC
AATCCTAGGGCCTTTCCATTGGCACGTCTGGGTGGCTCTTACATTTACTTACCTAATTGGGATATTG
CCTCTAGCTTTCTCCGACAAACACACCCTTCGTCATCTATTACACAACAGTGGTGAAATAGAGAAT
ATGTTTTGGTACGTCTTTGGTACATTCACGAATTGTTTCACTTTTCTTGGTAAAAATTCTTGGAGTA
AAACTACAAAAATAACCACAAGATTACTAATAGGTTGGTATTGGATTTTCACTATAATTATCACAAG
TTGCTATACTGGCTCCATTATAGCATTTGTGACATTGCCTGTATTTCCTGAAACAGTCGATACTATAC
AGCAATTGCTTGCTGGTTTTTACCGCGTTGGTACATTAGACAGAGGTGGCTGGGAGAGATGGTTT
TTCAATTCTTCAGATCCTAATACTAATAAGCTTTTCAAGAAGCTAGAACTAGTTCCAAATGTAGAA
GCAGGAATAAGAAATACAACCAAAGCGTTTTTCTGGCCTTATGCATTTCTCGGTTCGCAAGCTGA
ACTTGAGTACATCGTTCAAGCTAATTTCTCAATGGCAAAATCTAAACGAGCTATGCTCCACATATC
TGATGAATGCTTTGTACCGTTTGGAGTGTCAATGGCTTTCCCAAGTAATTCACTGTACTCCTCCAA
ACTGAGTGGTGACTTAAGAAGAATGTTCCAAAGTGGCTTAATATACAAAATAGTAGACGAAGTCC
GCTGGGAAATGCAACGGAGCAGCAGTGGAAAATTGTTATCAGCCGGAGCAGGCAGTTTGAAAAT
TGTATCAGCAGAAGAAAAGGGATTAACACTAGAAGATACTCAAGGAATGTTCCTCTTACTCGCAG
CGGGATTCCTATTAGCCGCTTCAGCTCTCATTTCCGAATGGATGGGCGGAATCGGCAGACGGTGTA
GGCAACTAAGAAACAAACTACCATCAAGTGCTAATTCTAAGGAGCAGTTGGTGATTTCTTCACCG
GACCTAGAAAGTGAAGTGAATGATGGTACTGAAAGCAGGTTACAGTTCGGAACTAGATCTACAA
GTGCCGGTTCCAGAGATACTTTAGATGGTCAAGTGATCAATGTTACTGAGGAAAATATAATAGTCC
ATGAGTTAATGGTTGAGGGGTTGGATTCGCGGCGGTCGAGCTCAGTTGATTTAGATAGAGAAGTA
CAGGAGATATTTGAAAGAGATTTGCGAAGACGTAAGATAGTTACAGGGGACAGCATAGAAGTGT

CTGAGGAGAAGAGAGAGCCTACGGCATCAAAAGGTGCTTTTGGAGATCCGTTAAGC

>CpunIR7

ATGGCAACCGGATTAGAATTGATCTTGTCGTCTATTTGCAATGCCACATTTTGCGAGCCGATATTTG
ACAACCCACTTTTAGGAAGACAGGATTCGCCAAAAGATGTAAAATACAATGATATGGTCAATGAA
ATAAACGGAAAGCATCTCAAAATTGCAACCTATGATAACCGGCCTATGAGTTGGGTGGAGAAAGG
AGAAAATGGAACGATTATTGGCAAAGGAGTGGCGTTCGTCATCGTCAACATACTGCAGAAAAAG
TATAACTTCACCTATGAAGTGGTGGTTCCGGAGAAGAACTTTGAGATGGGCGGCGACAACCCAC
AGGATTCATTAGTTGGTCTGGCGAATAGCAGTTTAGTGGACATGGTGGCTGCGTTCCTGCCGAAG
GTGAATAAGTACCGGGAGAAGGTATCCTTCTCCTACGACCTGGACGAAGGTGTCTGGATGATGAT
GCTGAAGCGGCCCAAAGAGTCGGCAGCGGGGTCAGGGTTGCTGGCACCATTCGACAACGCTGT
GTGGTACTTAATCCTCATAGCAGTGCTATCGTTTGGTCCCTGCATCACTTTGCTCACCCGGCTGCG
CAACAAGATGGTCCCCGACGGCGAAAAGTTCATCCCCTTGTCGCCCAGCTTCTGGTTCGTGTACG
GAGCCTTCATCAAGCAGGGCACCAACTTGGCCCCTGAAGCTAACACGACGAGAGTCCTCTTCAC
GACGTGGTGGATATTCATAATCTTGCTCTCCGCGTTCTACACGGCAAACCTGACTGCGTTCCTCAC
TCTCTCCAAGTTTACCCTGGACATCGAAACTCCTCAAGACTTGTACAAGAAGAACTACCGTTGGG
TGTCGCCAGAGGGCAGCGCTGTTCAGTATGTTGTTAATAGCCCCAACGAAGACCTTTACTACCTC
AGCCGCATGATAGGCACGGGTCGGGCGGAGTTCCGAACGGTGCCCAACAGCCAAGACTACCTGC
CGTTGGTGGACGGCGGAGCGGTGCTGGTCAGGGAACAGATTGGGATCGACGAGCTCATGTATGG
CGATTACCTAAAGAAGGCGAGAGAGGGCGTGGCGGAGGCAGACAGGTGTACATACGTAGTCGCC
CCAAATAATTTCATGACCAAATTGAGGGGGTTCGCGTATCCCAGAGACAGCAAGCTTCAGTACTT
CTTCGATAGTATATTGACATACATCTTACAAGCGGGCATAATAGACTTCCTTGAGAAGAAAGACTT
GCCGAGCACGAAGATATGTCCCCTGGATCTTCAGTCGAAGGACCGGCAGCTGCGGAACAGCGAT
CTGATGATGACTTACATGATCATGGTGACTGGGCTCGCTGCGGCGGTCGCGGTGTTTATAGGCGA
GCTCTTCATCAAACGCTACATTTGCAAAACCAAGGACGAAGTTACCAAACCGAAGCGTAAAAAG
ACCAAGTTTGAAAAGCGCCTGCGAATTCATACCTACGACGACAGCCAGCCCCCGCCTTACGACG
CAATATTCGGTCGGAACCCGAAGATCAAAGTCACGGAGAGAGCTCAGAGGAAGATCATAAATGG
GAGGGAGTATCTGGTAATAGACGTGTCTAATGGGGAGACCAGACTTATACCCGTGAGGACGCCGT
CCGCTCTTCTATATCAGCTGGACAAA

Supplementary Table 3.

Primer	Forward primer (5'-3')	Reverse primer (5'-3')
OPB1	TGACGAAAGTGTGTCCGATGGG	CGACGAGATGGACGCAGTTCA
OBP2	CTGCAAGAAAGTTCCGGATGACTAC	GAAGAAAATGCTTGGATTGTGGTTG
OBP3	GTAGCACATAACATTGCGCTCCTCA	AAGTCGTGTATGCCAAAGAATGACG
OBP4	GCCATTGGCAGATATGTCAGTAAAG	CAATGAAAACGAAACATACTCCAGA
OBP5	GAGCTCGCCCGCAAAGTCTTC	TCGCAGCCCTTAGCACCGTC
OBP6	CCTGATAAAGATAAATGCCAGAGGG	GAACAAAAAGTAGAATTCCGGGTCC
OBP7	AGGCCGAGTTACGGTCTATTTCAG	CCTTTCTCCCCGTCGCTCAC
OBP8	CCCGAACGATCTAAAGGAGTCTGTC	AATACATTTGGCTGTCCAGTACGAA
GOBP1	TTCCCCAACGGCGAGGTGC	AGCGTGCGCATGCAGTGGTC
GOBP2	TGGCGAGGTTCTCTCAGCAAA	CAGTTCGGTCGCAGTCGTCAGT
PBP1	ACGGCGGACTTCATCAAGCAA	CAGTGGTCCTCATTGGGAACAGC
PBP2	TGTTCCCAATGAGGACCACTG	TCAGCCATGAGCTCTTCCAAC
PBP3	GAAGACAGGGGTGGCGTCAGAA	CTCCAGGTTGGTCATGAGCACG
PBP4	AGGATGAATGTGACCGAATGCTGG	CTCCACTTTGGGCGTCCACTGTA
ABP	AGCCGGTCAAGGCAGCTGTAGA	GCATTTGGCAGTCCAGTAGGCG
OR1	TACTCCCATCCATTGCTGACCTCGT	TCCTGCTCCATCTTTTCGTCGTTGA
OR2	AGGCGATTCGGTATTTTGTGGC	GACCAAGTTGAGATCCTGTTGGG
OR3	TATAGAATGTGACAACATCTCGAAC	ATATCTCTCAATGAAATAAATGAAC
OR4	TCAGAAGAACTGAGTTGGATCTGCC	CCGCTATGATATTTTTCACGGGT
OR5	GTGTATAACACACCGTGGGAATGTATG	TGGGGTTTGTACCTTCGCCAGA
OR6	TGCTCAACGAATGGGTCCATTTTG	ACGACGGCGAGGGTTAGGGC
OR7	CTGCAAGGGCACTACGTTGAGAT	CTCACCACCACCACGAACATGA
OR8	GTGTGTTCTTATTCCTTCCTTTCGG	AGTATCAATGTTCACGTTGCACC
OR9	GTACATCAACTGCAAGGGCATCA	CTAAAGGGCAATGGCTATAAACACC
OR10	CCCTTGTCTAACACTCAGCATCCT	GTTCGCTTTTTGCTCCAGTTTACG
OR11	GCCATCGCTTTCTGGTTCATCATC	CACGTCGTCCATAAAATGTCTGC
OR12	TACAACTTTGCACCGTTAGCGTTC	GGCAGACTTACAGCATACGGCAC
OR13	GACGCCGTTGTTAAAACTGCTC	GGCATTTTCATGTAGTCAAAGGGA
OR14	GACGCCGTTGTTAAAACTGCTC	GGCATTTTCATGTAGTCAAAGGGA
OR15	GAACAGTTACAAGAAGGCGAAGG	GAACAGTTACAAGAAGGCGAAGG
OR16	TTACTCCAAGCCATGCTAAAGACAC	GGCCACATACTTCGTAGTTCATTCA
OR17	GGATTAGTATTCAACAACATCGGCA	AATGGTAGCATGAGCTCAACCTCT
OR18	ACCTAGTGGTGCTGCTGCAGTTC	GGCTCCTCGTCTTCTAGCTGTC
OR19	TCCATCCAGTAGACAACGACGAT	CGATTCCCTCACTATTTTCCTCAC
OR20	GCAAGCTATGAAGGATGATTTCCAG	GAATGTGTCGTGTTGTGTTTTGAA
OR21	ACTCAACAAGAGAATGGAAAGGAT	GGAAGTCACCATCACTTGGTATA
OR22	CTCAAATCAGATGTACGAGAAGGTG	TAGTTGCGGTAAAGGGGGGTGA
OR23	CGAAAGTGACGATGAAAACGAAAC	CCTGAAGAGAAACGTCGAAACTGC
OR24	GACTGCTGTACTTCATCTGTGGG	GTTTCATTGGTTTCCTTGTTGGC
OR25	ACAGCATTCCAAGGAGTTGACGA	CACGCAACAAGCTTTCCAATAGT
OR26	GCGAAAATTATGATAAGATATGCCG	ATGTTGAAGAAGTTTAGGCCCAGAG
OR27	TCTGTATTACCAATAGGGATTACGG	GGTTCTTCCAAATGTTGATTATG

OR28	GTATTGCGTACCCCGGCTTA	CCACCAGCTCGTTGAGATTCC
OR29	TTCTGTGGTATAACCTGCATGGAC	GGAGTGTAAGATCAACCCTAGCTG
OR30	GCATTTCACTTGATTGGTCATATCG	GGTTTGGGCAGATTATGTAGATCG
OR31	ACTGACGCGAAATTTTTGCACAG	GACAGCTATAAGCACCAACGGC
OR32	GGTATCTGACTGCGAGCAACAT	CGGAAATGAAAACACGAATATGAC
OR33	GAGGGTCCAAGTGAAGGAGCTG	TGTGCGTGACGCTCGCG
OR34	GTTGTATGCAGTGCCTGTGATGTA	AGTCTTCAGTCTTTGCTGGAGTTC
OR35	CGCCTGAAAGCCGGTTCG	CCCGACGAGCACCATCCAGA
OR36	ATATTCTGAAACCTATTGTGATGCC	CTGAGAAAATAAGCCAGTTCGTAGT
OR37	GAGAAAAATGTGGAACAGGAAATGA	CGAAACCGTAAAATACGAGAGTC
OR38	CATCATTGGCCTCAACGATGAGT	ACAGCCGCATCAGGTAGAACG
OR39	CATGAACGTTACTTGATGAGGATCA	TGGCATAGTCATTCAAGACGTCA
OR40	GATATTTCGACGGAGCAGAGAAG	GCTAGGCTACAATTGCCCAGA
OR41	GTTGTAGAACCATACTCCCAGCAC	TGGCAGAGGAAGATACGATGATAA
OR42	AACTCGTGGCGTTAAAGGTTTCGT	TTCAGCGACTGGTTGTCGGTGT
OR43	CATCGGATATTTTCATCACGTT	ACGCTGTTGCTTCATTTTTGCTC
OR44	CTGGTTACTGACGCTCATGTACTCC	AATTGCTCAACTCATGTTGTTGTCT
OR45	GGTCGGCCTAGCAATGTCTTG	GTAGCTGCAGTTCCACCTCTTGT
OR46	CAACATCGTCAGCCATCTGTTC	GCTCCTCTGGGTCCCTCC
IR1	CTAGCAAACGGTCTGCAAACTTC	CATTATGTATTTAGCCCGCGTGTC
IR2	GCGTTGATTACTGAGAGCGAAG	CAGAGACGGATGTTGGAGCG
IR3	TTAGGAAACGTAAGACGTGGTGATC	CCAGCAAAAGAGTTGTGGTAGCAT
IR4	GTTAATAAGAGCACAGACGTGACTG	CGATGATTACATCGGAGTCCGG
IR5	CGTGGACCTTTCCGTTGGA	TGTCGTGCGACGATTACTTGAG
IR6	CTACGACAGCCAAGTAAAGTCAAAG	TCATCAATATATCCGTGCACATATG
IR7	GTGTCTGGATGATGATGCTGAAG	CACACAGCGTTGTCGAATGG

Supplementary Table 4.

>SexiPBP1

MAGAKWQFVCVVFALYLTSAALGSQELMMKMTKGFTKVVDDCKAELNAGEHIMQDMYNYWRED
YQLINRDLGCMILCMAKKLDLMEDQKMHHGKTEEFAKSHGADDEVAKKLVSIIHECEQQHAGIADD
CMRVLEISKCFRTKIHELKWAPNMEVIMEEVMTAV

>SexiPBP2

MAFCRSATMSVRVALVVAASMLVVVQASQDVMKNLAINFAKPLDDCKKEMDLPDSVTTDFYNFWK
EGYELTNRQTGCAILCLSSKLEILDQELNLHHGRAQEFAMKHGADETMAKQIVDMIHTCAQSTPDVA
ADPCMKTLNVAKCFKLKIHELNWAPSMELIVGEVLAEV

>SexiPBP3

MGSHNVFVALVLLAVGMRVAEPSKDAMKYITSGFVKVLEECKQELNMNDHIIADLFHFWKLEYALL
SRDTGCVIICMSKKLDLLDANGRMHHGNAQEFAKRHGAGDDVASKIVQIIHDCEKKHERDDDECLR
VLEVAKCFRTGIHDLDWQPKVEVIVSEVLTEI

>SexiGOBP1

MLFLLRALPLLAAVLPLRADVNVMKDVTLGFGQALDKCRQESQLTEEKMEEFFHFWRDDFKFEHRE
LGCAIQCMSRHYNQLTDSSRMHHDNTEQFIKSFPNGEVLARQMVELIHSCEKQYDHEDDHCWRILH
VADCFKQGCVQRGIAPSMEMMMTEFIMEAEAR

>SexiGOBP2

MTAEVMSHVTAHFGKALEECREESGLSAEVLEEFQHFWREDFEVVHRELGCAIICMSNKFSLLQDDT
RMHHVNMHDYVKGFPNGHVLSEKLVELIHNCEKRFDSMTDDCERVVKVAACFKVDAKAAGIAPEV
AMIEAVMEKY

>SexiOBP1

MSKFTCLVLCVVAGCLSGVHATAEEKAALIEAVKPYIQECSKEHGVTPEDIKSAKEAGNADGINACFL
RCVYNKAGVINDKGEYDADKALEKLKKFVSNEDDYAKFAEIGKKCASVTETSVSDGEAGCERAALL
TSCFLEHKSEVHA

>SexiOBP2

MKSFVVFCIVLVVGVCANEKGNKLDRPFASECIKETGVKNELLEEAKKGIISEDPAFKAFTYCFFKKI
GIVGEDGLLNRDVAIAKLPSGVDKSEAEKLLDSCKSKTGKDAVDTVFEIFKCYQQGTKSHIMFAS

>SexiOBP3

MVKLTCVVFCAVAMALSVFVAGEDANSVFQGAIKPLIAECAKEYKLSDEELLKNRGLAGLSNLPPCF
IGCVLKKFDIINDKGLYDAEAGIAKIEKLLPNNEFLDKISGVLKSCESANEKSVGDGDAGCERAVLVAT
CYLEHKTAVIA

>SexiOBP4

MWNFLVVFLAICSCVYGLTEEELKMEFTKLIMKCNKDGKVDMTELVQLQNYVVPTKQTTKCVLAC
AYKAAEVMNAKGEYDIDHAYKVAEMMKNGDEKRLVNAKKMADLCVKVNEQSVSDGEKGCDRAA

MIFKCTVENAPKFGFKL

>SexiOBP5

MTMKQIRNTGKMMRKTCQPKNNVEDEKIDPIAEGVFIDEKEVKCYMACIMKMANTIKNGKLNYDA
AIKQADLLLPDDIKEPAKEAITACKKVADAHKDICDASFHITKCIYNHNPGIFYSP

>SexiOBP6

MLGSLLFVFAFSVFSLGAEALLIDDLKQKYADSILQCSQQYPLDRADAELLQNKVMPDKESTKCLFA
CVYKVTGVMSDQGELSVEGVNALSQKYLADDPEKLKKSEEFTEACRTVNDAPVSDGARGCDRAAL
IFKCTIEKSPDFSFV

>SexiOBP7

MTKVLFAIVLMMITFAVTLSASTKEAMTTTMTDQVNSIEVDVLAVMDMCNDSYRIDPTYLQALNES
GSFIDETDKTPKCFIRCVFENVGIVSEDGMQLNPARAAVIFAGERNGKPMEDIADMTALCATDRQETC
PCDRSYKFLRCLMSMEIERYEKS

>SexiABP1

MSVVRYSSFVVALFCLVSVNAMSGDEEAGVRDALRPYVQECADEYGITEEQFEEAKKKASADDIDP
CFMSCFLKKAEFFDAQGKFDVDSTMAFAKEHLSSEPAMKFVEAVGDECVKINDEDVSDGDKGCDR
AKLLFDCIAETKKKMD

>SexiOBP10

MDRKRICLFVIAMFLASGSDAMSRQQLKNSGKMLKKNCMNKIGVTEDQVGSIDKGKFIEDRKVMC
YIACIYELTNVIKNNKLNYEASIKQIDLMYPPDIKESAKAAVEKCKDVQKKYKDICEVSFYAAKCMY
EFKPEDFIFA

>SexiOBP8

MARRQQGAMFTETLPLFVILVAVTHGGKDKPVFSDEIKEIIQTVHDECVAKTGVAEEDITNCENGIFKE
DAKLKCYMFCLLEEASLVDDDDTVDYDMLVSLIPDEYYERTTKMIFACKHLDTPDKDRCQRAFEVH
KCSYEKDPDLYFLF

>SexiOBP9

MKTLFVFAACILLAQALTDEQKEKLKKHRTECLSETKVDEQLVNKLKGGDYKTESEPLKKYALCMM
MKSELMTKEGKFKKDVALAKVPNPADKPTVEKLIDACLANKGNTPHQTAWNYVKCYHEKDPKHAI
FL

>SexiOBP11

MKEGNRYSHERRITNDSGDQLMVINATDDDYSGYGSGNMGEKLLTSVPRPASSSNNINKNNTRRTRR
NEPFLNRPDSDQCLSQCVFANLQVVDSRGIPREAELWNKVQTSVTSQQSRSALHDQIRACFQELQSE
AEDNGCSYFNKLERCLMLRFSDRKVDGKGNPKKSSTEQT

>BmorGOBP1

MWKLVVVLTVNLLQGALTDVYVMKDVTLGFGQALEQCREESQLTEEKMEEFFHFWNDDFKFEHRE
LGCAIQCMSRHFNLLTDSSRMHHENTDKFIKSFPNGEILSQKMIDMIHTCEKKFDSEPDHCWRILRVA

ECFKDACNKSGLAPSMELILAEFIMESEADK

>BmorGOBP2

MFSFLILVFVASVADSVIGTAEVMSHVTAHFGKTLEECREESGLSVDILDEFKHFWSDDFDVVHRELG
CAIICMSNKFSLMDDDVRMHHVNMDEYIKGFPNGQVLAEKMVKLIHNCEKQFDTETDDCTRVVKV
AACFKKDSRKEGIAPEVAMIEAVIEKY

>BmorPBP1

MSIQGQIALALMVYMAVGSVDASQEVMKNLSLNFGKALDECKKEMTLTDAINEDFYNFWKEGYEI
KNRETGCAIMCLSTKLNMLDPEGNLHHGNAMEFAKKHGADETMAQQLIDIVHGCEKSTPANDDKCI
WTLGVATCFKAEIHKLNWAPSMDVAVGEILAEV

>BmorPBP2

MKLQVVLVVLTVEMVCGSRDVMTNLSIQFAKPLEACKKEMGLTETVLKDFYNFWIEDYEFTDRNTG
CAILCMSKKLELMDGDYNLHHGKAHEFARKHGADETMAKQLVDLIHGCSQSVATMPDECERTLKV
AKCFIAEIHKLKWAPDVELLMAEVLNEVSWKS

>BmorPBP3

MARYNIVVAVLVLGVVGARGSSEAMRHIATGFIRVLDECKQELGLTDHILTDMYHFWKLDYSMMTR
ETGCAIICMSKKLDLIDGDGKLHHGNAQAYALKHGAATEVAAKLVEVIHGCEKLHESIDDQCSRVLE
VAKCFRTGVHELHWAPKLDVIVGEVMTEI

>BmorOBP5

MKQRLRVLLLRFCILQTVLSESGVDVVKNLSLSFARFFLECDEERHFQPEVRLKVMTFWYSESSTWD
RDVGCAFLCIFKKMEIDNPQDPSYRTHLELLSFANSEDNKIANQMVEIFYACGENTETDPCLWALEQV
KCYKNRINQLGLTPTF

>BmorOBP8

MLRVVVICVCFLVIAPYGINASSLDDLKMVYKNVIKECVGDYPITAADLKLIKARQIPNDDIKCVFAC
AYKKTGMMTEEGMLSVEGIKDMSQKYLSDNPEQLRKSKEFAEACSSVNDQQVSDGTKGCERAALIF
KCSTEKITNFGFEL

>BmorOBP9

MLRVVVICVCFLVVAPYGINAVSYEQKIKIRDQLDRAGFECFKDHKITEDDIKNLRANKPATGENVPC
FIACVMKKTGVMNDQGVIRKGPVLELAKKVLADDKDIKKLQDYIHSCSHVNSETVHDKGKGCEFA
MQAYTCMSANASKFGFNI

>BmorOBP10

MLRVVVICVCFLVIAPYGINAVSDEQKIKIREQIDKSGFECFKDHKITEDDIKNLRARKPATGENVPCFI
ACVMKKTGVMNDQGVIHTEPVLQLAKKVLTDDKDIKKLQDYIHSCSHVNSKTVHDKGQGCEFAIQ
TYTCMSANASKFGFDV

>BmorOBP11

MSANSFVVLAFCALAVGVNALTEEQKAEITKSSLPLIAECSKEFSVNQGDIDAAKKLGDPSGLNSCFV

GCFMKKAGIINASGLFDVAATIEKSKKYLTSEEDLKAFEKLTETCAPENDKPVSDSDKGCERAKLLLD
CFVANKGSFSVFSL

>BmorOBP12

MTSFMVFFVLSVLTLKYSDALTDEQKNKIQSKFIEIGAECIVEHPISIDDINSFKNKKFPSGVNAGCFVA
CIFNKIGLFDDKGNLSHNSALEKAKGIFNADEEVKNLEEFLNRCAKVNGEAVGDGVKGCERAKLAY
NCLIENSLEFGFNIDF

>BmorOBP13

MLKIHVLLCFGMAILYFGSAKAVTPEESKAFEAFAKPVIEQCQKDFGMDKESFAQKNLDEIDECLIAC
VVEKFGITNDEKIDGDALKALVTKFVGNEEERNKINKIVEECTEDANKSGDGTCNTSTILFLCLLKNG
KDLWGF

>BmorOBP14

MSRQQLKNSGKMLKKQCMGKNDVTEEEIGDIEKGKFIEQKNVMCYIACIYQMTQIIKNNKISYEASI
KQIDLMYPPELKESAKASAGRCKDVSKKYKDICEASYWTAKCMYEDNPKDFIFA

>BmorOBP15

MFLKNIFIECVLLYFVMLNTSFVNTMTKQQIKNSGKILKKACISKNDVTEDQISDIDKGKFIEDKNVM
CYIACVYSMSQVVKNNKFVHDAMVKQVDMMFPTEMRDAVKASIANCRGVAKNYKDICEASFWTA
KCMYEFDPANFVFA

>BmorOBP16

MRISFLFLISVTIITFDSVFAMTRAQVKKTMTIMKNQCMPKNGVTEDQVGKIEEGIFLENHNVMCYIA
CVYKTIQVVKNDRLDKDLISKQIDVLYPQEIRESTKKAVGDCINLQEKYDDWCEGIFRSTKCLYEKDP
ANFIFP

>BmorOBP17

MTRQQLKNSGKIMKKTCMPKNDVTEEEIGQIEQGKFLEQRNVMCYIACIYTVTQVVKNNKLSYDAV
IKQVDVMFPAEMRPAVKAAAENCKDISKTFKDICEASYWTAKCMYDFDPKNFVFP

>BmorOBP18

MILIVIAKFLILISLCETMTMKQIKNTGKMMRKSCQPKNNVDDEKINPINDGVFIEENEVKCYIACIMK
MANTMKNGKLNFEAAMKQADLLLPDEMKEPTKEAIVACRKVADSYKDVCDASFHVTKCIYNHNPS
VFFFP

>BmorOBP19

MTSAKTDVEIKAWFLGQAVECSKDHPVTTEELRMHKHELPDSKNAKCLMKCVFRKCNWLDSKGM
YDINAAYASSTKDFSDDKTKQENANKLFDTCKSVNEENVGDGEEGCDRSLLLAKCLTKAAPQVSIY
YS

>BmorOBP20

MAVHIFLILASYMALAAHGQLDDEIAELAAMVRENCADESSVDLNLVEKVNAGTDLATITDGKLKC
YIKCTMETAGMMSDGVVDVEAVLSLLPDSLKTKNEASLKKCDTQKGSDDCDTAYLTQICWQAANK

ADYFLI

>BmorOBP21

MITASLHVIFALLAFVYGGKDKPVLSEEIKEIIQTVHDECVGKTGVSEEDITNCESGIFKEDVKLKCYMFCLLEEAGLVNDDGTVDYEMFTSLIPEEYFDRATKMIFSCKELDTPDKDKCERAFEVHKCSYEKDPDFYFLF

>BmorOBP22

MLKVFVVVVCTLGASQLCAALYTQKVAVSFPKDKTTIVVEAMKSCIAKTGANPNVIEVISSGKVSEDEKFKEFFYCACNDIGVVNPDGHIKVKECIELFPKETQPLVEPVIKNCDKEGVNKYDTLFKYLKCFQETSPVRVTLA

>BmorOBP23

MTSKVLLSCVVLAVLATTVLAEDSRKLVSFAPEVAKKLKVLIQECLNENGLGEDAIEVIRAGEYREDEPFQNLVYCAYKKFGALDENNRIISQVAAASFPKDIDVVTVIESCGKEDGNTPVEQVFKYFKCFQKNSPVRMQLY

>BmorOBP25

MKSVVLICLAFAVFNCGADNVHLNEDEREKANWYTAECGVETGVSTEVINAAKIGKYSKDKAFKKFVLCFFKKSAILNSDGTLNMVVALAKLPSGVNKSEAQSVLEQCKNKTGQDAADKAFAILQCFHKGTKTHILF

>BmorOBP26

MKSVVLICLAFAVFNCGADNVHLAETQKEKAKQYTSECVRESGVSTEAINAAKIGKYSKDKAFKNFVLCFFNKSAIFNSDGTLNMDVALAKLPPGVNKSEAQSVLKQCKNKTGQGAADKAFEIFRCYYKGTKTHILF

>BmorOBP27

MKSVVLICLAFAVFNCGADNVHLTETQKEKAKQYTSECVKESGVSTEVINAAKTGQYSEDKAFKKFVLCFFNKSAILNSDGTLNMDVALAKLPPGVNKSEAQSVLEQCKDKTGQDAADKAFEIFQCYYKGTKTHILF

>BmorOBP28

MLKVFIVTFFAFQLSAIARLQANGCVAVPFPKDKTIIIVEAMKSCIAKTGANPNFIDVIRSGKVSEDEKFKEFYYCTCNDTGFVNPDGHIKVKECIELFPKETQPLVEPVIKNCDKEEGVNKYDTLFKFLKCFQETSPVRVALA

>BmorOBP29

MTGPAAAAVLLALLAAAGQATTGCKNCVILGKEERAMFRSHSDACLAQSRVEPRLLESMMNGELIDDAALRKHVYCVLLSCKMIGKDGKLLKAAILGKLAARPAGRDVTKVLEACAEQPGASPEDVAWNIFRCGYNRKAVLFDYMPAGGASSGNTENHP

>BmorOBP30

MRSFVILLNYGLLCCGQFMAEDYYYDIVTRDPDDLMREKENEVRALRAFQADCAEDVQVKPDLVV

NLKSGDWQTEDVSLKKWALCVLMKLGLMTAQGVFKMNEAMSKIPDMNDKIIAEKLIDDCLSLQAT TPHDAAWNYIKCHHQKDPEGNFSSLNIF

>BmorOBP31

MKTFIVFVVCVVLAQALTDEQKENLKKHRADCLSETKADEQLVNKLKTGDFKTENEPLKKYALCM LIKSQLMTKDGKFKKDVALAKVPNAEDKLKVEKLIDACLANKGNSPHQTAWNYVKCYHEKDPKHA LFL

>BmorOBP32

MYSHKYLNDFTNIPEILIILLSSVALMSYGYNTKLFSHSLGSEPSLSILYARDKKSDKVTNECLMEMYP KNLYKYPLRIDRNDIPCIIHCVLKKFGIISNDGFINIKNYYRRVQAIHRYDPRILISDVGETCAQNINGM NLDHDVCKKAKVFNDCTQLYAISYREPEDW

>BmorOBP33

MYAHDKLSDMIADQCLNEMYPRSKRLEIEESDEPCIIFCVLKKFGIMSPTGVINLEAYRKRVQLPEQL AQRNSINDFGSACLESAEATQHKQDVCKKAKVFNECTHLYKILLK

>BmorOBP34

MEKMILLNVFAVVLPCVLASRTRGSSGTLVDFTDPKVQGHLDALVRMAQSCVIKVRATPKDVRAYFT NSSPVSRSGQCFATCMLEQSDIINHGKVNRDLLVHLAGLVNGKNSRVVRKLNSVSRLCLDSISGMTD RCQLASTYNDCLNENMIEFAFPLDIAEEAVRKMPFHLIQPK

>BmorOBP36

MAVSEISRILTFLTIVSFIYIVYSFKPLTKDEHIERYNKMNEDIEPFRKNLTECARQVKASMADVEKFLK RIPQSNMEGKCFVACILKRNSLIKNNKLSQENLLEVNRAVYGDDSEVMSRLKTAILECSKIVEDIFEIC EYASVFNDCMHMKMEHILDKITMERRMEALGQMSSNPDEWSEEEDEMLKLVKDEL

>BmorOBP37

MFYPFRFTLLFYGLFVIYLVRAEPEKENHFTLALKKTLFSTARSCMSHVNANETDLEYLRKDPPFPDK AACIIKCLLEKIGVVKNNKYSKMGFLTAVSPLVFTNKKKLDHYKSVSENCEKEINHDQTTECELGNE VVSCIFKYAPELHFKT

>BmorOBP38

MANLVLLLTFVLMTLSMARLKSTEAPKSKTALFNDQDNMGYEELDMEEIMSACNESFRIEYAYLESL NDSGSFPDETDKTPKCYIRCVLEKTEILSENGVLNPATAALVFAGERNGKPMSDLEEMAVACADRHE KCKCEKAYNFVKCLMYMEIDKYEKKN

>BmorOBP39

MVRKISALLCCFCVLGISMCDSAISTDNEQRCKNPPTAPQKIERVITLCQDEIKLSILREALDVIKEEHT MPAERKRNKREVPFTHDEKRIAGCLLQCVYRKVKAVDGFGFPTLEGLVGLYSDGVNERGYFMAVLE ASRECLMKNHDKFSRTTPMDNGRNCDVSFDIFECISDRIGEYCGTSGL

>BmorOBP40

MSEFIQPSWRTQCNFRLNWDNRNRLSIDISHGAATTQTPVPTTKPKALRDFMVVPQSCDKTTCVFKK

LNIVSDKGVVDVKSFIKLLDKFTNSYPVWNSAKARVITTCLRKSLIAYDGGCELNNILACTFDVLSENCPLNGNNQTC

>BmorOBP41

MLTILFLLPIVVGVLSGNIPEQPRVYCGELPNTIYSCLGNPKIIQPEVSEKCNKPISECDKTRCIFKESGWAKNNVIDKKKVSDYFEQFAKDNPDWSAAVQNFKTTCLSDSLKPQGVDTNCPAYDIIHCALISFIKFASPSQWSTSEQCVYPRQYAGACPVCPERCFAPSVPNGSCNACLALLRTP

>BmorOBP42

MMGYACVFVILAVLQAISAEDPPGLPPFLKDAPEKCKSPPRVKNPNECCISEPFFKEADFIECGIEKPGSERGPPDCSKQNCLLKKYNLLKNDETPDIEAIKSLLDKYIEKNPSFKSSVEKAKECLREDLPGPPQICLANRMTLCIGTVLLMECPDEKWNTTDDCKAFKDHMTECQKYFPK

>BmorOBP43

MKVCVLFAIFTVAQAAKATLKPISACCNIPELGNPEPLAECSNPKLPGPCKDIQCVFEKSGFLTENKTLIKEAYKTHLRQWAKEHEGWSVAVEKAISDCVDKDLRQYLEFPCSAYDVFTCTGIAMLKKCPNEHWTC

>BmorOBP44

MSRLVLFFTILVVLQEFIINLYFNFITEIDSCCVKKYPKLFDSEFITECYNTQRKANDKCERDMCVARKLNLLTEEDSINKDALLRFVEEGFKTEIDLVNAIKKKCFEEDISNIGKPEMCEVAKYKICITSRMAEDCPKWDSKGICSSAQQKVENFMKMLS

>HarmPBP1

MEFHRSTMMSVRLALVVAVCLFIRVDASQDVIKNLSMNFAKPLEDCKKEMDLPDSVTTDFYNFWKEGYEFTNRQTGCAILCLSSKLELLDQELKLHHGKAQEFAKKHGADDAMAKQLVDLIHGCAQSTPDVADDPCMKTLNVAKCFKAKIHELNWAPSMELVVGEVLAEV

>HarmPBP2

MAASRWLFARAFCLVLMMGSAMSSKELLTKMTGGFTKVVDACKTELSVGDHIMQDMYNFWREEYQLVNRDLGCMIMCMTAKLDLIGDDQKMHHGKAEEFAKSHGADDALAKQLVGLIHGCETQHQAIEDHCSRALEIAKCFRTKIHELKWAPSMEVIMEEIMTAA

>HarmPBP3

MGSRHVFFALVVLAVSVRKAEPSKDAMQYITSGFVKVLEECKHELNLNEQILADLFHFWKLEYSLLGRDTGCAIICMSKKLDLLDANGRMHHGNAAEFAKKHGAGDEVASKIVTIIHECEKKHEQDGDECLRVLEVAKCFRTGIHELNWQPKVEVIVSEVLTEI

>HarmGOBP1

MPGVLRALLVLAAAAPLLADINVMKDVTLGFGQALDKCREESQLTEEKMEEFFHFWRDDFKFEHRELGCAIQCMSRHFNLLTDSSRMHHDNTEKFIQSFPNGEVLARQMVELIHSCEKQFDHEDDHCWRILHVAECFKGSCVQRGIAPSMELMMTEFIMEAEAR

>HarmGOBP2

MTSKSCLLLVAMATLTASVMGTAEVMSHVTAHFGKALEECREESGLSAEVLEEFQHFWREDFEVVH
RELGCAIICMSNKFSLLQDDSRMHHVNMHDYVKSFPNGHVLSEKLVELIHNCEKKYDTMTDDCDRV
VKVAACFKVDAKAAGIAPEVAMIEAVMEKY

>HarmOBP1

MSKFTFFVLCVVAVSLSKVYASDEDKAKLHEALKPLVEECMKDHEVSLDDLKAAKEAKSADGVKPC
FLACVYKKAEVLNDKGEFDADHALEKLKEFVSDEDVLAKVAEVGNTCKAVNDKAVSDGDAGCER
AALLTACFLEHKAEILV

>HarmOBP2

MMDRKRLCLLIIAMFLAQGSDAMSRQQLKNSGKMLKKNCMNKNQVTEDQIGSIDKGKFVEDKKV
MCYIACIFEMTNVVKNNKLNYDASIKQIDLMYPPDLKESAKAAVEKCKDVQKKYKDICEASYWTA
KCMYDFKPEDFIFA

>HarmOBP3

MSKFTCFVLCVLAVSLGEVRSNALEKAAIRAAVYPLIVDCAKEHAVTLEQLKAAKASHSAEGINPCF
QSCVYKKTGIFNDNGEYDVANAKTKLQKFVTDEDEYARIAEVGKTCASVNDKSVSDGAAGCERAA
LLTACFLEHRAQIII

>HarmOBP4

MSKLTCVVFAAVAVVFSNVNADDETRASFRQVLGPLVMECRNEFGITEDDLKKAQQERSPDALKPCF
IACVFKKFGIITSAGKYDSDASISRIKDVVKNDDLLAKLKSVGEKCNSVNDASVSDGDAGCERAALL
AKCFIENKSELSI

>HarmOBP5

MSKFTCLVLCVVAASLSQAYASEEEKAAFREAIKPIVEECSKEHGVSHDELKSAKDNQNADNIKPCFL
GCVYKKAEVFNSKGEYDVDKALEKLKKFVSNDEAYAKFAEVGKKCASVNDKAVSDGDAGCERGA
LLTACFLEHKAEVPL

>HarmOBP6

MSKFTCLLLCVVAVSLSKVHATEEEKEAIRAAVRPIMQECGKEHGVTLDDLKAAKAAHSADGIKPCF
QSCVYKKAGIFNDNGEYDIANAKTKLQKFVTNDEEYARIAEVGKMCASVNDKPVTDGAAGCDRAA
LLTACFLEHRAQIII

>HarmOBP7

MFRFGVLSFVVLLFCMESSYALSSEEELSIKEALHPFVVECAEEYGMTEEMFEEAKKKGSAEDIDPCF
MSCFLKKTGFFDDSGKFDAEKSISFAKEHITSESAIKFLEAGAGECVKINDEDVSDGENGCDRAKLLF
DCLTELKKKMSE

>HarmOBP7.2

MSRFGVLSFVVLVFCMENIYALSSEEELSIKEALHPFVVECAEEYGMTEEMFEEAKKKGSAEDIDPCF
MSCFLKKTGFFDDAGKFDAEKSISFAKEHITSETAIKFLEAGAGECVKINDEDVSDGDKGCDRAKLLF
DCLTDLKKKMSE

>HarmOBP8

MLLIEIVKFLTLVAMCEAMTMKQIRNTGKMMRKSCQPKNNVADEQIDPIAEGVFNEDKEVKCYMACIMKMANTIKNGKLNYEAAIKQADLLLPDDIKEPAKEAITACRKVADAYKDICDASFHITKCIYTQNPGIFYFP

>HarmOBP9

MCKFSVLFLYSAVMAVNIWSASCISEEDKAAIITAIAPLAQNCGSECGLDNDDFEKYKEDGSDMDPCFKACLMTQMGVLDKEGKYDGKGLHKAMEEADYPGDKDDAQKFLDELDRCFDAKGDNSGSDEEAKMKRADVLFRCMQDMKEK

>HarmOBP9.2

MCKCSVVFLYLAVMAINIWRASCLSEEDKAAIITAIAPLAQNCGSECGLDNDDFEKYKEDGSDMDPCFKACLMTQMGVLDKEGKYDGKGLHKAMEEADYPGDKDDAQKFLDELDRCFDAKGDNSGSDEEAKMKRADVLFQCMQDMKEN

>HarmOBP13

MFTGTLPLVVFLATFAYGGKEKPVFSDEIKEIIQTVHDECVAKTGVAEEDITNCENGIFKEDPKLKCYMFCLMEEASLVDDDDAVDYDMLVSLIPEEYVDRTTKMIFSCKHLDTPDKDKCQRAFEVHKCSYEKDPDLYFLF

>HarmOBP18

MKSFVVFCVLVAGAFAANVSLPPKQNEKANQIATECMKESGLKPEVLAEAKKGHISDDEHLKKFTFCFFKKAGIVSEDGKLNTEVALAKLPPGVDKAEAEKLLETCKGKTGKDVTDTVFEIFKCYHHGTKTHILLGF

>HarmOBP15

MGSRHVFFALVVLAVSVKKEKPSKHPMPYITSRFVKVLEECQHELKLNEHILEHLFHFWKLEYSLLGKDPGCAIICMSTKLDLLDLYGRMHRGNAAEFAKKHAAGDEVPSKIVTIIHFCQKKHEQDGDECLQVLEVATCCRTGLHDLNWQHQVEVIVPDVLTEI

>HarmOBP16

MFKLCVVLAFIVATCHGGTLERTSSTCGQIPRELTACLDLQPAVSPEIQEKCRRANECERLTCVFREYNLLDGAEVNKERTAAFLDNFVKQYPSWEVAIDVAKTSCLRSSGLKPQGVFLDCPAYDIIQCVFANLVKNALPSQWSSMSQCNHAREFAAACPICPDACFAPLVPIGTCNACSAARRSS

>HarmOBP17

MRAWSVTLVALLGALGAARAVAMDEDMAELARMVRENCAAETGADVALVERVNAGADLMPDDKLKCYIKCTMETAGMMADGEVDIEAVLALLPPELAEHNAPSLRACGTVRGADHCDTAFRTQQCWQNANKADYFLI

>HarmOBP18a

MTRQQLKNSGKLMKKSCMPKNDVTEEEVGDIEKGKFIESRNVMCYVACIYTMTQVVKNNKLSYEAVIKQVDMMFPAEMRDAVKAAATSCKDITKKSKDLCESAYWTAKCMYDYDAENFVFP

>HarmOBP22

MTREQIKNSGKLIKKTCMAKNDLSEDQVKDVDKGKFIEEKPFMCYIACVYKMGQTIKGNTVNHDM
MIKQVEMMFPNEMKAPMKAAIEHCRPVVKKYKDVCEVSYWTAKCIYEFDPPNFMFP

>AipsPBP1

MAPHPSVTMYVRLALVIIAGLFITVECSQEIIKNLSLQFAKPLEDCKKEMDLSDTVITDFYNFWKEGY
EFTNRQFGCAILCLSSKLELLDQDLKLHHGKAQEFAKKHGADEAMAKQLVDMIHSCTQSTPDVADD
PCMKTLNVAKCFVAKIHDLKWAPSMDLIMGEVLAEV

>AipsPBP2

MAASRWCIACLVCVLFAARSVMTSQEVVASFSKGFTNVVEHCKAEVNAGEHIMQDIYNFWREEYQL
VNRDLGCMVLCMANKLGLIGEDQKMHHAKAEEFAKSHGADEAVAKQLVAILYECETKHAAVEDEC
GMALEIAKCFRTKMHELKWAPSMEVAMEEIMTAV

>AipsPBP3

MGTYNVFFAFVLMAAGVREIEPSKDAMKYITSGFVKVLEECKQELNMNDRIIADLFHYWKLDYTLL
NRDTGCAIICMSKKLDLLDDTGRMHHGNAQEFALKHGAGEEVASKIVTIIHDCEKKFERDDDECLRV
LEVAKCFRTGIHDLDWQPKVEVIVSEVFTDM

>AipsGOBP1

MTQPGQVLVLVLLAAAALADVNVMKDVTLGFGQALDKCRQESDLTEEKMEEFFHFWRDDFKFEHR
ELGCAIQCMSRHFNLLTDSSRMHHVNTEEFIQSFPNGEVLARQMVALIHGCEKQFDHEDDHCWRILH
VAECFKHACVAHGVAPSMEMMMTEFIMEAEAR

>AipsGOBP2

MTLRCCLLLVVVAAVTRSVVGTAEVMSHVTAHFGKALEECRDESGLSAEVLEEFQHFWREDFEVVH
RELGCAIICMSNKFSLLQDDSRMHHVNMHDYVKGFPNGEVLSGKLVELIHNCEKQYDTLTDDCDRV
VKVAACFKVDAKAAGIAPEVAMIEAVMEKY

>AipsOBP1

MDISKRRSKNAFRRLLVNTWLRLVQIFTCLSAPPVVSADVTSKCQGSKYENECDKLTCVFRKAKWL
DGNAVDKAKLITYFEQFEKDHPEWAPAMQNVKTSCLGAELKTQGVFLNCPAYDVMHCVLGSFIKHA
TPTQWSTSASCSYPRAYAAACPICPEDCFSAQVPFGSCNACYLPPRTP

>AipsOBP2

MSKFTCLVLCVVAASISRVHADDDANKAAFREAFKPILDECSKEHGVSNDDIDAAKKAGSADAIKPC
FFGCIYKKAEVFNAKGEYDVDSALSKLKKFVPDEAKFAKYAEIGKKCASVNEKPVTDGDAGCERGA
MLTACFLENRAEMLI

>AipsOBP3

MIRSCRCLVFAAVFQVVLGQGLTGTDSGPPGFQRPQSYVPKHCFAPPPGVDLHTCCPIPQLFPDEDME
SCGIQKLTKEQYENPSPARIPCQESICLLRNANLLKQNNSIDYEKMGDFVDNWAKMDPDFTIPITNAK
KVCLIEGGPPAPPVCEPDRIFTCLTSYVLWNCKLRLDSGEGCKILKEHMDGCRPFLAGP

>AipsOBP4

MFGYQFLSFAAALICFGSSYALTSEEEANIKEAFHPFIMKCAEEYGITEEQFEEAKEKHSAEGIDPCFM
SCFMKESGFFDSAGKFDADKTKEFVDAHLTSERAITFMEAVGSECAKVNDEEVTDGDKGCDRAKL
MWGCIQDLKEKMEGSE

>AipsOBP5

MKYFVLFVALVAGIHANVTLPPEQSEKALKTASECIKETGVSKEVLAEAKKGHIADDEGLKKFTLCFF
KKAGIVDNDGKLNLETALAKLPPGVDKAEAKKVLEGCQAKSGKTPQDTAFEIYKCYHAGAKTHIAL
AGI

>AipsOBP7

MSKFTCVLCVVALSLSSVYVTRAHKPNLRDAWRSELDECAKEYPVTNDEIDTAVRSGDSSNLNPCFN
FCVFNKTGFFTENGEYDLKNGLIKLRKAIRDDEEYTKFEEVATECTEDKNTSCDEKAKCDSANRLSL
CFLRFKDKVRI

>AipsOBP8

MYLRSTNGGVRSFPLGESAYTTKIVEICSKETGLKKQVPPEEKEIKFSQRKGLREFNDCYLAKTGVTT
SDGKLNIDEALEKLPPGFAKPFVEHCQANIILGYIEENVNDFSTCFHQEVQNHLLSFYGFENYWVMLV
LGTSFDKTRFTTLFFDKHFDFWLAERAGFVNL

>AipsOBP12

MYSGTIFLFSFILLIVSNVTFVSSQMTREQVKNSGKLVKKTCSAKNDLTEDEVKDVDKGKFIEEKKFM
CYVACVYKMGQAVKGNSLNHDMMIRQVDMLFPADMKAPVKAAIEHCRPVAKKYKDICEASYWTA
KCVYEFDPPNFMFP

>AipsOBP13

MVLIYIVKFLILVAMCEAMTMKQIRNTGKMMRKSCQPKNNVEDEKIDPIAEGIFIDEPEVKCYMACI
MKMANTLKNGKLNFDAALKQADLLLPDDIKEPAKEAIIACKKAAEGHKDICDVSFHVTKCIYNQNP
GIFYFP

>AipsOBP15

MDHNRLCLLVIAMFLATGSDAMTRQQLKNSGKILKKNCMNKHQVTEDQIGTIEKGKFVEDKKVMC
YIACIYELTSVIKNNKLNYESSLRQIDIMYPADLKESAKAAVENCKDVQKKYKDICEASFHTAKCMY
DFKPEDFIFA

>AipsOBP16

MFPGSIPFISGCVHLGVSNYFRSTQSNLVVHYEDDQIVDAIYNCQDENGFDEVLSNSTNLEENFPEKE
GLKKSNDCFLKKTGFVTSDGKLNIDKTLEKLPPSFVKPIVEHCQANIALNYTTESVENFSSCYHDGIL
NHIFAATEVGIFPFIQTWKFFVPGTSFADTILILN

>AipsOBP17

MNQLLVFVLIVACVRISNGMTREQVKKTMTVIKKQCMPKNSVTEDQIGKIEQGVFNEDRNVMCYVA
CVYKSLQVVKNERLDLGLISKQIDALYPPELKEPTKKAVSQCINIQDSYNDLCEAVFHSVKCLYEKDP
ATFIFP

>AipsOBP18

MKTLFVFAACILLAQALTDEQKEKLKKHRTECLTETKVEEALVNKLKGGDYKTESEPLKKYALCMMTKSELMTKDGKFKKDVALAKVPNAADKPSVEKLIDACLANKGNTPHQTAWNYVKCYHEKDPKHAIFL

>AipsOBP19

MFTGTVPFVLCLVAVAFGGKDKPVFSEEIKEIIQTVHDECVANTGVAEEDITNCENGIFKEDPKLKCYMFCLMEEASLVDDDGTVDYDMLVSLIPDEYYERTTKMIFACKHLDTPDKDKCQRAFEVHRCSYEKDPDLYFLF

>AipsOBP21

MLKFSVVCLYFSVAAVNFWNVHCISEDEKKAFIEAMKPMVEECGSDCGLTEEDYKKHSKGEDMDPCFKKCMMQKLGFLDEDGKYNRKQLHESISEYTGDKDEAKRVQEQLDSCFDANGDNDGDDEESQMKRVDVLFKCLKEIKE

>AipsOBP22

MSMWFRAMVVVGALAAARCGVVMDEDMAELARMVRESCVDETGADVKLVEAVNGGADLMEDDKLKCYIKCTMETAGMMSDGEVDIEAVMALLPPEMAEHNGPALKSCGTQRGADDCDTAWKTQVCWQNANKAEYFLI

>AipsOBP23

MSKFTYLVLCFVAVSRVYANEDERAAFHEAAKPILVECSKENGVSFDKLKAAKEAGSADGIDPCFFSCVFKKTGVFNSKGDFDLDNSLTKLKEFVSNDEDYAKVAEVGKKCESVNEKDVSDGEAGCERASLLTACFLEHRAEIPV

>AipsOBP24

MAKLLLAMILTVMTFALTMSATTKDAGTKEAIMTTTVANQDSSIDSNDVDVLAVMNVCNESFRIEMSYIQALNESGSFVDETDKTPKCFIRCVFENVGIVSEDGRMFNPARAAVIFAGERNGKPMDDIADMTALCAADRKETCPCDRSYQFLRCLMSMEIERYEKS

>AipsOBP26

MSKFTCIVLFVVAASLTKVTQAVSEEEKAVAREAMAPILAECSKAEGVSDEDIEEAKKNPSVDAVNSCFIRCVMRKTDALNEKGLFDSDAALAKIRPFVKSDEDFAKFEEIGKACMSVNDKEVSDGEAGCDRAKLLLACFLEHKAEMLY

>OfurPBP1

MGLSLRLLVVVAAAIFGAESSQDVMKQMTINFGKALDTCRKELDLPDSINADFYNFWKEGYELSNRHTGCAIMCLSSKLDLVDPEGKLHHGNTHEFAKKHGADDSMAKQLVELIHKCEGSVADDPDACMKVLNIAKCFKAEIHKLNWAPSMDLIVAEVLAEV

>OfurPBP2

MWLSKTLVVIAVMCSMSVVVHSSQAVMKDMTKNFIKAYEVCAKEYNLPEAAGAEVMNFWKEGYVLTSREAGCAILCLSSKLNLLDPEGTLHRGNTVEFAKQHGSDDAMAHQLVDIVHACEKSVPPNEDNCLMALGISMCFKTEIHKLNWAPDHELLLEEMMAEMKQ

>OfurPBP3

MWLPKTLVVMSVMSSMSVVVHSSQTVMGEMTKNFIKAYEVCAKELNLSEATGLQLINFWKEGHEL
TTRETGCAILCMSTELNLLDVQGSVHRGNTVEFAKHHGSDDAMAHQVVDILHACEKATPNEDKCM
LALSIAMCFKAEIHKLDWAPNNELMFEELVLDMWNS

>OfurPBP4

MADATKWRVAAILVICFTVNLNTVMSSEELMTKMGVTFFNVLEECKKELKVTTNINEGLVRFWSQG
AAPERELGCVFLCMAHKKDLLEDQKRIHHENAHQFARGHGAEDDKATEIVSLLRECEQQFITITDDC
LRALEVARCFQAHMQRLQWAPSMEVMVEEILAGMA

>OfurPBP5

MKGFAGVPVTLMLVLIGVSEIEMVPEAMKQLTGGFLKVLDQCKKELNLSDGVISDLYHLWKEEYDQ
ISRDAGCVIHCMSQKLELLGGDGRMHHVNIKDFALKHGAGDEIATQLVTLAHECEKQKAAIEDDCER
TLEMSKCFRSDVKQVDWTPKMEVIITEVIEV

>OfurGOBP2

MVCSGFYLGLVVMAAVTSVKGTAEVMSHVTAHFGKALEECRTESGLSPEILEEFQHFWSEDFEVVH
RELGCALICMSNKLSLLQDDTRIHHVNMHDYVKGFPNGEVLSEKMVNLLHNCEKQFDDITDDCQRT
VKVAACFKVDAKKEGIAPEVAMIEAVMERY

>CpunGOBP1

MVSDARLVVAACVVAAAGLVAGDHKIMTDVTLGFGQALEHCREESGLTEDKMEEFFHIWHQEFKFV
DRELGCALHCMSKYFNLITDANKLHHENTDKFIRSFPNGEVLASKLVSLIHECEKQFEEVEDHCMRT
LRIGECIRDSCLQRSLAPSMEMLLAEFIMQSE

>CpunGOBP2

MLPIWLYFGLVMAAVSSVKSTAEVMSHVTAHFGKALDECRDESGLSPEILEEFKHFWSEDFEVVHRE
LGCALICMSNKFSLLQEDTRIHHINMHDYVKSFPNGEVLSAKMVELLHNCEKQYDAITDDCDRTVK
VAACFKNDCKKEGIAPEITMIEAVMERY

>CpunOBP1

MMKLNIFVSVAVLAAVLGNARALTKEELGVIESDMIAHVKKCGEQFGVSDEEIKAAKEKKDIDGIDP
CLIGCVFKSTKLINDEGVFDPKVALEHSEKYLSSDDDKAKFKDIADDCAKVNDESVSDGKEGCERAK
LLLSCFAKHKDELRPSRR

>CpunOBP2

MLLVLIAKFLMLLATCETMTMKQIRNTGKMMRKSCQPKNNATDEQLDPLNEGVFIDEKEVKCYMA
CIMRMANTMKNGKPNYDAAVKQADLLLPEEMKQPAKEALFACKKVPDDYKDPCDAAFHVTKCIFN
HNPSIFFFP

>CpunOBP5

MISNYKYIQWFGLFGTCSWKSLKMIRAVTLFCGLFLMALTPNVDAMTEEQRAKIREHFETVGMQCIG
DNPLSEEDITALRSKKAPSDSASCFLACMMKNVGVLDDSGMLQKETALELARKVFQDEEELQIISDY
LHSCSPVNSAAVSDGAKGCERAMLAYKCMIENASKFGIDV

>CpunOBP6

MSWTGLAIIVTVLTVCHGKDTLELSDEIKEIIQHVHNECVGKTGVAEEDIRNCENGIFKDDKKLKCY MFCLMEEANLVDDDDNVDYDMLVSIIPEEYTDRTTKMIFSCRHLDTPDKDKCQRAFDVHKCSYGKD PEFYFLF

>CpunOBP3

MFDLSFAMTRQQLKNSGKLMKKSCMPKNDVTEEQIGDISSGKFIEERNVMCYIACVYTMTQVVKN NKISYDAVIKQVDMMFPPEIKDNVKAAAAHCKDVSKKYKDLCEASYWTAKCMYDFDSESFIFP

>CpunOBP4

MFKSGVCFVFIVSLLEFSMSLSDEKKAEILAKFIKVGEKCIIDYPLTKEEIAAFKEGKFPDSRGAACFSA CILTKIGLMDDKGEISITAALERAKTIFKDEEELKIVEDFLNTCAKDGGTKGEDKCDRAKEIFICFIKKS KKFDL

>CpunABP

MFLGHFYISCLLLIIFDTYHVMSMTRQQMKNSGKILKKTCMPKNDVTEDEVGNIEKGKFIETKNVMC YIACIYSMGQVIKNNKIAHDAMFKQIDMVFPPEMKEPVKAAVEKCKPVAKKYKDICEAAYWTAKCI YDADPENFVFA

>CpunOBP7

MIHAVILFCGLLFVTWMPYSDAVTPEQRAKIGLHFHAVGMECIVDNPLAEEDVLAFRNRMPPPGPNG ACFLACVLRRVGAMDDAGMMQRDSLLELARTVFHDEAELRSISDLLLSCSSVNSIEVSDGEKGCERA MLALKCMLEQASKANSLDEKNMIRDKFESVGEECIEKHPLSDEDIAALENKMPPPGRVGACFVACV MKNVGVMDDAGMLQKETALELAREAFDDEEELESIADFLHECSSSINSVAVSDGTEGCDRAILALKC MNEHESKFGLDL

>CpunPBP1

MGMLVKLLLVIVASVGVECSQDILKKMTVNFGKALEACKKEIDLPDSVNAELYNFWKEDYQLTNRQ AGCALVCMSTKLDLVDPDGNMHHGNAHEYAKKHGADDATAKQLVEMLHTCEKSVGKMDDNCER ALAIARCFKAEIHKLKWAPDPEVVLAEILAEV

>CpunOBP8

MDGRICLLLVFLVGGSDAMTRAQLKNSAKMLKKNCMAKNSVTEDQIGNIEKGQFIEEKPVMCYIAC IYQMMSIVKNNKLNYEASIKQVDMMYPNDLKESVKKSIENCKSVSDKYKDICEASYWTAKCIYEDN PKDFIFA

>CpunPBP2

MAFKMWLKNIMVVATVVMMSVKVDSSQTMMKDMTKNFLKAYGECQQELHLTDDTARDLMFFWK EDYEVTSREAGCTILCLSKKLEIIDPEGKLHKGKTADFIKQHGSDEETAQKVIDVLHACEASAVPNED HCIMALGVATCFKKEIHKLNWAPDTEVLLEELMAEMSER

>CpunPBP3

MSRVVSVTLLALLALSVGLSVGASTGTSGSSATTSGTTSNDLSRSTDDVKGKLITPADDQGNMTSSEE RALDVPDLMAVMVECNDSFRIEMGYLESLNESGSFPDEIDRTPKCYVRCVLEKTGVASEDGLFDPAQ AAAVFAGERNGVLMTNLEDLASRCAADRNEKCKCERSYNFIKCLMEAEIKEYVSN

>CpunPBP4

MKGFFVTLLVVFMGGKEVEMSSDGMKQLTTGFLKVLGACKTELGLSDGILSDMYHLWKEEYEQVS
RDSGCMFSCMSKKLDILDGDGKIHHDHTKEYVLSNGGGEDLARQLINVAHDCEKQQESLEDECDR
MLEIAKCLRRNIKEIQWTPKVEVIITEIVADM

Supplementary Table 5.

>BmorOR3

MIFVDDAVIGIKDPREYRHLRVLRTSLRLLGAWPGHYLGEETGSKYECAPMFLLMFIKIACLYLTIVYL
RNNADVLGFFELGHVYLTIFMTFVTLSRGFSLTWNPNYHKVVKKFITEMHLLYFKDNSEYAMKTHR
RVHKISHFYTVFLKVQMIAGLTLFNVIPMYNNYRQGNYASDRPANITYDLSIYYETFDILNTPNGYIFI
CVFNWFASYICCSFFCSFDLILSLMISTVSGHFRILIHNLLTFPLPEAITASKKFVDKHRCNGNRSEFVLE
EAKLYSPAEMWQVTDRLRQCIDYHRKLVEFTGDISEAFGPMLFVYYLFHQVSGCLLLLECSQLNTAA
LVRYGVLTVVLYQQLIQLSVIVESVGTVTGRLKDAVYEVPWEYMDTSNRKTVAIFLMNVQEPLHVN
ALGLAKVGVQSMAAILKTSFSYFTFLRTVSE

>BmorOR4

MFKIIKNIIVENDALKQVEKPQEFQYMKWVQYHLKYIDGWPNMDMNKKNVSKIRFHKRHLLVVEQ
TITFLSQMFYIVKNYGKLSFFEIGHSYITALMTIVIFSRSVVTALGRYRKIARYFVSSLHLYHYKDISEY
ALQTHLLVHRLSHYYTVYLISLVVTGMLLFNITPLYNNISSGVFNSPRPENMTFQHAVYLGLPFDYTT
DIKGYFVVFILNWHLSHIAASYFCTFDLFLSLLILHLWGHLRIILNNLKTFPKPYTNNSMYTEEENQVV
LLKLQECIRYHNFIISFTVMMSNVYDVVIIVYYLFHQVTGCLLLLQCSTLDWESLSRYGPLTLIIFQQLI
QVSMIFEILGFLSDKLPNAVYSIPWEAMNVTNRKLVQVLLQKSQKPIQFKAMNMMSVGVQTMASIIK
TSISYFIMLRTIARD

>BmorOR5

MLLYYPNTQVKEKVNNVEEFTYIKFLKSFCKIMDFWPEREEKNSKTRIFRLRYILVLQFCFTLVAGVL
YLTNSVGKQTFYDLGHTIITVLMNVVSLSRLILRCFKKYDVVGQQFINKIHLYHYRNDSEYAMKIHTV
VHKISHNMTYIFSFCIIFGTVTFNLTPIFNNIGSDAYKNPRPDNVTLQQCVYYALPFDYTGNFKWYLLV
AIFNVQKTFFCTSLFILFELSLSLMIICLWGHLRIFIHNLNHIPAPRNSFEYTKEERQEVDDTLKKCIQHH
TLIIGFVRIMSETYGLAVLIYYAFQQVVGCLLLLQCSQMELKTVTRFGFLTLVLNQQLIQISVIFELLGY
MSDKLQDAVYCVPWEYMDTSHRKMVYMMFRQSQIPLQLKAMNMLSIGVKTMVSILKTSVTYYLIL
KTVTTD

>BmorOR6

MKEEYYLQHPRTQLFYKVLAHVSTIESTIDLTWWGYTFPKYVGWFYHLQCNVVRLFGKCVVVSQIL
FIILNYQTIDKSVFIIAITITPLGALVGIKAESAKAECYVNLMKNFMDKVHIHSIYRKNENNEFVKKKVI
QIERVSRFTAYFLVILIAINCLSWMLKPTLHNIKHFEEIMNKSMEFQYYIYFWTPLDYKYNLRDYIIIHT
LCIYLGATAVTVIVTFDIFNFIAVFHVVAHIQILKNNVKSNWSDDFNESEKKGYLVSILEYHAYIIRIFGE
VQSAFGLNVASNYLQNLIEDGLFLYQIMNGEKENVLMYGLMIILYLGGLIFLSIVLEEIRRQNYDLCEY
VYALPWEGMSLENQKIFVVFLQRTQPDLEFETVCGMKAGVKPAFSIVKSMFSYYVMINSRF

>BmorOR7

MLLYHPNTQVEEKVNNVEEFTYMKFLKSFCKIMDFWPEREEKNSKTRIFRLRYILVLQFCFTLVAGVL
YLKNNFGKKTFYDLGHTIITVVMNVVSVSRLILRCFKKYDVVGQQFINKIHLYHFRNDSEYSMKTYK
AVHKISNNMTYIFSFSIFVCVVTFNLNPVFNNIGSGAYKNPRPDNVTLQQCVYYALPFDYTGDFKWY
MLVAIFNVQKTFFCTSLFILFDLLLSMMIIHLWGHIRIFIHNLNHIPAPRNSLEYTREERQEVDNTLKKCI
QHHTLIIGFVRIMSETYGLAVLIYYAFQQVVGCLLLLQCSRLDLKTITRFGFLTTMVNQQLIQISVIFEL
LGYMNDKLQEAVYCVPWEYMDTSHRKMVYMMFRQSQIPLQLKAMNMLSIGVKTMASILKTSVTY
YLMLKTITANEA

>BmorOR8

MSLSTRCLLKDFCKYVVYYAGAGNFWYEDIYKETVPYKMYVVISFFTYTVMIFLENLAAFGKLPEV

EKNSAVMFAAIHNIVLTKMFLLLYHKRSISKLNCEMAAVGENLEEASIMRRQFRKMRLGTALYFISVY
LSLVAYGVESARRTIVEGAPFYTVVTYLPDYDNTTVLASFLRIFFYITWLYMMLPMMSADCMPIAHLI
TMTYKFVTLCRHFDQIREKFQINVKIMAKTEATEILKLGFIEGIKMHQKLMYLADEIHRVFGIIMALQ
VCESSAVAVLLLLRLALSPHLDLTNAFMTYTFVCSLFLLLALNLWNAGELTYQASLLSNAMFYSGWY
FCDFEKDWCRDIRRLVLIGCAQAQKPLILKAFGVLDLSYETFVSVARMTYSVFAVFYKRGD

>BmorOR9

MVARRPLQFHQGRNVDNVEDFKYVKWLRNHLKTVDAWPVHSKSKRKIQKRYVLPIFSAACFISQTV
YLKNGIGTLSFVVLVHSYICFLINGSCLCRGILIATERYKRLATCYLKTVHLFHHKNRSEHAMKIHVIV
HRLSHYYTIYLISLVFVGMVLFNFMPIYNNINSGAFKSPRPENVTFQHAMYLALPFDYTTNIKGYFVV
FILNWYISLVTTSHFCTFDLFISLMIIHLWGHIKILMCSLEDIEGFVPGSSFKFTIEQNRKIYLILQECIRH
HQFTIDFTNEMSSTFGLVILFYYFFYQVSGCLLLLACSQMDIESLSRFGPMTFILFQQLIQLSIVFELISS
LSENLPNAVYNVPWEFMDKNNRKMIQVLLLQSQKLIQFKATSMMNVGVQAMATILKTSVSYFIMLR
TMYQEH

>BmorOR10

MRTNAKSFLFVPSKVLTLCGVWPVEKTSIFSLIYRSIMLSSQFCFLVFNGIYIGLMWGDLKAVSDALY
MFFTQTTCCSKAIGFYFNFMKIKRIVASMDDVLFTAMSIEDQATIFSHSRTVNKLYKGVLGFTGFTLVQ
WTVLSLIGSGRTLPFNEMWVPTDISKSPNYEITFVVELWMMVISAALFMSVDTITVATMMFSCAQLDI
IMKKTQQIQEIPLSPDLSSRNRSELHEKNNGILIDCIKQHQAIVRFSELCEGTFQVHSFFHLGGIVFMIC
VIGFRMAGESPVSAQFWAALSYLVIILGQLYLYCWCANELTTKSEQLRDKLYLTPWYDQDVKFKRNL
CIAMECMAKALTFRAGSYIPLSRAMFVSILRSSYSYFAFLNQANEQ

>BmorOR11

MDEHSHFETSLNKIKVLFKYSGMNLENTVTNTYEFLNHRWVYILNHAWTLAAVTFICIGISNGQNFIE
MTCIAPCVAMTVLAVSKSFFHYINENAVKSLLENLIELERTDFERTKSVQRTEIVATEKQLLNMVINVL
YVLNCSMILVFDMTPLIIIAIKYWTTNKFVRLLPYLDIFVFVPYKFEYWVMAYILQIWAECIVLLFIGA
ADCLFFTCCTYIRIHFRLLQYDFERLTSSRRESDGLRDDEDFRETYTNLVKRHQGLIESSSILEMIYSKS
TLSNFVLSSLVICLSAFNVTVVNDVTIVMTYLIFLAMSLMQVYFLCFFDMLMSASEEVGNAVYNCSW
YTEKASTGKDLLFTITRAQKPCELTAAHFAYVNLKAFMRVSFTSASITTLPTI

>BmorOR12

MTRITDVFSLNFIFWKFLGLWGKSAPSKYNMAYTVFYLFASLFVYDIFLTLNLIHTPRKLETLVRETM
FYFNHLVAVTKILMMFIMRKKILVIFDLLDCEEFKPNDENSQEIMKRKTDFYYIYWRIVAVTSNLSCFM
LVIGPLIKMLIWKIELGLPVCKFYFMSDELRNKYFVIWYIYQSFGIYNQMVNNLNLDTFNCGMLWMA
VGQLQILKTKFVNLKLNDFENGLDLKSRDDMQIERLRKYLTHYEIILKYCAIVQDILNITIFVQLGMSS
IVICVGLCGFVAMPSNTETAIFMFSYLTTMTMQIFVPSWMGTQISFECGELMSAAYSCEWIPRSKLFKR
SLILFVERAKTPVRITGLKIFTLSLDTFTSIMKTTYSFFTLIRQLQVDEVN

>BmorOR13

MAPKQIDCFEINWKFWKFLGIWSENKPHRYYKYYSKIFITFFVILYDVLYTINFYFVPRQLDLIIGEML
FYLTELSVLSKVFTFIIMRHKLKIIFEILESDAFQTDTEEELKILHRAKVFIKRYWKIVALVSITANLTHIS
SPLLKNLIFKVELVLPVCSYSFLSESFLKTFEYPLYFYQIVGIHFHMLYNLNIDTYFLGLMILIIAQLDIL
NVKFRNLKSGKDHTQLNESIMGLNKNLDHYNEIERFCSLVQNIFSFTLFVQFSMASCIICVCLFSFTLS
VPVEYYIFLATYMFIMIIQIMVPCWFGSRIMDKSILLSSAIYNCDWTSNSKDFKINMRLFVERANKPLS
ITGGKMFSLSLATFTSIMNSAYSFFTLLRYIQTRE

>BmorOR14

MSNYIFKPFHETYRIITFTMIAAMIYPNPATEKRRLIYIGLMLLSVIPLAFMIVTEMYEFFMASDLNNTI
RHSTVIGPFIGGFVKVALMYYKRRQANELVSEINRDHLAYNGLKGEDREIAASSIRNCQIYCELGWTL
IVMSCGLSFPVIAILLKIHSFTFKLDSTKHMIHDINNPFTDDPEDRFESPFFEIMFVYTFFSSFIYIINYVG
YDGFFGLCINHACLKMKLYCRALEDAMRSDSRRHEKIVAVIEEQRRTYEYIALIQDTFNIWLGLIYVA
TMIQMCTCMYHIVQSFNIDVRYIIFVISIIHIYLPCRYAANLKCMAAETPTLIYCCGWESVSDLRIKRM
MPFMVARSQVIVEITAFNMFAFDMELFVWIMKTSYSMFTLMRS

>BmorOR15

MMTLVYQTDIFKPNVFFWKMFGIWADRKSSKTYKYYSFVFLFITLIMYNSLLAINLLYTPLKIELLIRE
VIFCFTEITVTTKVLMILFKRNKILDAFDLLNKNEFRGNSEESSAIIQKNNSAYKTYWKLYAILSNFAYS
SQVLGPLIVKLIWKTKLELPICNYYFLNEELRHDFFSGWYIYQSFGMYGHMMYNVNIDTFISGLLMM
AVTQLKIIQTKLLSLKLNPRERKMDRGLMNITEVLKLNEILKHYELVLKYCSTVQSILDVAMFVQFGV
ASAIICVAMCGLIMVRSSTETLLFMVTYLFAMTLQIFVPAWMGTQLHFQSQELVFAAYNSEWIPRCQS
FKRSIIIFVERAKIPITITGLKMFPLSLATFTSIMKTAYSFFTLIRNMQTLQEE

>BmorOR16

MSFNSEDLYLNRAKFVMKYLGVWVPPENENFARKFYKIFMMSLQHLFLFFQIIYIVEVWGDLEAVSQ
ASYLLFTQACLCFKITVFQINMNKLKELLKQMNGYVFQPKNINQQNIIKVQATRIKRLLFAFMISSQLT
CGMWALKPLFDDVGSRKFPFDMWMPVSPERSPHYHLGYSFQLVTICMSAYMYFGVDSVAFSSVIFG
CAQIGVIKDKIMSIKPLGIYRNHKTYTKISRYNRKTLIECVKHHQAVISFTELVEDTYNSYLLFQLVGS
VGIICMSALRILVVDWRSVQFFSILCYLSVMISQLFVCCWCGHELSATSEELHTILYNCAWYDQDVKF
KRDLNFMMARARRPILLRAGYYISLSRQSFVSILRMSYSYFAVLDQTNK

>BmorOR17

MREDKMEINNSQKFYTKMIFRYLYSVGLGDWWYQHEDRSDSHRKLYCLWAVISNAYIFLNICNELLA
NFRKDLTDVEKNDAIQFSFAHPLIFAKIASFFFNRKKIREVFGRLLEENRSVYSCGELEKESMKQIKRY
SLAFIGVSYMTLVMSTIDGLRAHFKEGIPIRTEVTYYPSPSNSGVIVNILRFLVEFHWWYIVSVMVAID
SLAVASFVFVTFKFKLLQRYFKDMGLTVRRDQSNMTDEALADKFRRDFIVGVKLHENALWCAENVQ
KAFGWVYSVQVFETVALLVMCLVKLVTTNHNMIFLLANFAFMLCVIILNGSYMMPAGDVTYEASEV
PTSIFLCGWELVRQTDLRFLVVVAIQRSQVPVIMKAFGIMTLSYSNFIAVSLFKFYVQFQINLF

>BmorOR18

MGDRMVTRGHFFDFNIKYLFYVGLWPSNEAKRIEKIAYKIYEYQLHVLSLIFLVTTGIGTYKNHKDII
ALLTNLDKTLVAYNFVFKVIVFVYKREELRKLIEQIVQSGDQITEDRKALMAKLVIVLTGISTVIITAFS
CLALFEGEMTIDAWMPFDPMKSKMNLFAASQILAATFVVPCGYRAFAMLGIVCSLILYLRDQLVDLQ
NKIRDLRFATGNVEKLRDDFKLIVKKHVRLLGYSKVIEMIFKEYFFIQNMAVTAELCLNAMMVSVVG
LEQKTLAASFLAFLSVALLNAYIYCYLGNELIVQSEGIAMAAYESSWILWPVDMQKDLLIVITAAQKP
MKLSAGGMAVLSVQTYSQTLYNGYSIFAVLNDIVN

>BmorOR19

MHEFVINVQNETTKLYDQLNIILYILGLQGIWVDEIKLSRRFHVFFKVVTFILHIMCGMFAGLQFFAIFT
QNSLNSQQKSDVIVIGISNPMAYIFCINFIRNRNEIKDLFYHLAVVLKIYYNDVEIEKSMVNKIKSYLST
YVFASITILVSNGIIAFFQTINSDEPFLGIITAWPDKTDTSKTASYARIGFYLFWCIHFFRISTVFAVIVCILI
SIKYQYKFLCSYFESLNKIFDDETSSHEVKEAEFENAFCNGIKIHTQIIWCVRRCQIMCRTVFSANIML
DTFVLVILMLAMVNSENDFYGLCSQMSSVLVTVVLMAFFMWTAGDINVQASQLPDAIYGSGWYNC
RGKSSARIRSLVTISMNKAQQPILMWALGFVELSHKNFVAIIKSAYSVFSVFY

>BmorOR20

MIQASKYPNSKTKELFRKIAHIAYICGLPNFWIEELNLPKSFIRVYDKIVRIFNVATYFFLGIEIAAHFTQ
HHLTNKQKFDLLLYSISHPILNGYGVIVSRQVGNVKKVLLDLIVNLKVKYNDPVIEEAMIKISMTYSV
SFITNCVLSMLTYTFDALLMVYKKGVTFNVIITAWPDVEDTTTEASIGRIGFHIFWWLFVTRPFAVYVL
VINLTTCLSHQYMNLQSYFFHLEDIFKENLSQNEKEAKYEAEYKIGVMLHANTLRCTRRCHMVWNG
VMSGQIIFNISLIVIIMAQMMNSDRTLVNTFGTVLTASAILISTGFFMWNAGDVTVQASRLATAMYCS
GWQNCRGKSSVSIRNMVMNTIAVAQRPLVLRGLGVIDLSYQSYLSIVKASYTVFSVIY

>BmorOR22

MNKNHYILKTYCDKIFLVGSGNFWHQKTESRNDKTLLYKIYSCVLFFTYGFMTVLEIMAATMGDFPE
DEKRDSVTFATSHTVVMIKFISIIKNKELLKTLNRKMMMICEAHEEQTLMDEMYRTVKINVVAYCVA
VYGSATFYVFEGLRKFYNGSHFVTIVTYYPSNDDDTMLASIVRIATTLVLLMMLLSMIISVDTYTMAY
LIMYKYKFITLRHYFKRLRENVDELVAAGKARLAAEKLAQGLVEGIKMHNELLSLSKDIHKAFGTV
MALQLCQSSGSAVSLLLQIALSDQLTFTMGMKIFFFLAAMYLLLALFLCNAGEITYQASLLSDEIFYC
GWHKCNSPVLSTQRNIRDIVLIAILRAQSPLVMKAFKMVELTYATFILVVRSTYSVFALFYAQNK

>BmorOR23

MRAKTEFEKTIKLTKTALFLSGINIFLGEWNHWTRTFVDSIAYYLNIVGLYFVLIGEMYWLIDGTITGK
SFVELSLIVPCLTISVLATAKVHYLYHNKESLLDVVDKLREIYPDEIEETANDNDQCLNDKKETVYDN
DVTEVGIVNEANELLKFVNFLLSTVSFVVTMTFCTMPLFGMAGEFMETGKFVVLYPFAVKYPFDVY
NTSFWVIVYVNQFWATIIVCTNIFGVDTLFYALCSYIGMNFRLLSYKFEHLEIKRNDRIINEIIVLIKRHQ
ELIELVNKTQSLYSLSTLFNIVTSSLLICLSGFNITILSRSWSYFALLKTIYS

>BmorOR24

MPEELFLDRSIKKIESYFRWMGINIRSGDNNNKKDVFKIRCIYFINFVLLNTDVLGAIFWFRSGLEQGK
TFTEVTYNAPCLTFSFLANFKMLSLIFYEKTVHELIAALQKLEIKHFLRQNCAEELKMLKDEKNFLHA
VFKGSKIVNYASILTFGCSPLVLIASNYYKTGRMDYLLPLIVLYPFDVDNITVWPIIYVRQIWSVITAVI
GVCATDYLFYTFCVYISTQFRLLGHSIERVVPNNGLSVRTRLNGNLRMKFVENLKWHQELIRAASLL
EQIYTKSTLYNFVTSSVIICLTGFNVAVVEDFAVILSFLFFLFMSLLQIILLCFFGDKLMKSSTNISDAVYN
SKWYLTEKNVGKVLLMVQIRSQRACRLTAYGFAEVNLRAFMKILSTAWSYFALLQSLYSSHE

>BmorOR25

MFEKALRSANFYMRVIGIPTDIRDGNRTLMERLRNRWFYCINFLWLNTDVAGEITWFVKGLLSGSST
LIENTYLIPCLTLCILGNVKTFFTIKYANHIIDLVAILKDLEIKNNAARKNETEIVKERLKFLTTSNKFLLF
VIGTGIIAFGIGPLMLTASIYFSSGDMKLKLPFLIWYPFDSSDIRYWPFVYVHQVWSACIACCAVYGPD
CFYFTSCTFIHIHFIHLQNDITNVIVESSRARRNGLYRGCHQAFLELTNRHKDLIRCVNLLEIIYSKSTLV
NVVSSSLLICVTGFNVMAIDFLPLIAPFTSFLALGLVQTYLLCYYGDTIMCSSTEVSDAVYNSTWYGT
NISQMRDYLFVMKRAQKPCKLTAYGFSDVNLRTFSRILSTAWSYFALLITIYRGNGQQ

>BmorOR27

MPSSFFLPNLENPDYPSLGPTLKGLKYWGMWQSGGIKRILYNSIHAFATFFVITQYVELWIIRNNVELA
LRNLSVTMLSTVCVVKAGTFVCWQKYWSGIIGFVSNLEKEQLSKNDAATQAAIVKYIKYSRRVTYF
YWSLVTATVFTVILAPLVGFLSSPERELIANGTLPYPEIMSSWVPFDRSRGFGYWVTALVHTLICFYGG
GVVANYDSNAVVLMSFFAGQMKLLSINCSRLFDDGNEVISNNEAMKRIKECHYHHVFSTIFNSLMSP
VLFLYVIICSLMLCASAVQLTTDGTSNMQRIWISEYLMALIAQLFLYCWHSNQVLYMALEDRLGGLF
EACLESGRFPSKWKTGRLVLLRKDGRPADSPAGYRPIVLLDEAGKMLERIVAARIVRHLTETAPDLSA
E

>BmorOR29

MFDFLQNLEDSERPLLGPNFWLINKTGLLLPKTNFGKLAYILVHEIVTFFVVTQYVELYVIRSDLDLVL
TNLKISMLSIVCIVKVNTFVFWQTSWREVLEYVNEADKFERNQTDETRGKMIETYTKYCRRLTYFY
WSLVFTTFLTTTNTPLMRYWSSPIFRENLRNGTEDFPHIFSSWMPFDKNHSPGSYCTIVWHVLLCAYG
AAIMAAYDTCIVVIMVFFGEKLNLLRERCKKMLANDLYNHAFVIGQLHDIHVQLIKQSRLFNSLLSP
VMFLYILMCSLMLCASAYQLTSATSTAQKLLMAEYLIFGIAQLFVFCWHGNDVLFKNANVSLGPYES
NWWSSSPRVRADVLLLCGQLRVRHVFTAGPFADLTLSTFIKILKGAYSYYTLLRK

>BmorOR30

MSVSNLKFEVLFKPTTMSLHMNRSHPSIKRNKIWLLQFISLMTLTAFCATGLITSLLFHDLKFGKYME
ASKNGTIAMLSFTTTFKYSLLLYLQKSLNRLIAKIDMDYEIAKGLTPQEKVTVLNYAKKGVIVSKFWL
FTAFAITFCFPLKAFIIMGYRFIIKDEFRLEPMFDMTYPEPIESYKTSFPVYFILFVVCFLFGCYASSLYVA
FDPLVPIFVLHACGQLDLLSVRITKLFSDTKNPRIIAKELKVIIIKLQELYSFVNFIKVNFSILYEYNMKIT
TISMPLSAFQVVESLRRGEFNIEFTYFFFGCILHFFMPCYYSNLLMERSENFRFAIYSCGWENHNDKNI
RQMLLFMLTRAAEPLGIATVFTNISLDTFAEVNTFDTVLLA

>BmorOR34

MELNFDKIFRIAIISQKFSGTYPYTKRDKKWATHFILMHGELTIICMLFIYNIIEFDLKAANYSQMCRN
MCLSFLYMVITLLYINMLYYQSKLKMLIETMKAEYEIAKTMSEEEQNVILEYAKKGRWLCRAWAILT
TCGMAQFFLKSIVCTIYSAIQGNFRIVQYYEVICPEVIERHRNNPVIFITMYFCTFFYSLYTSALYTSVLP
LGPIFLLHGCAKLEIVRLNIKNLFDNDDYVVQERLKKTVLQMQDIYW

>BmorOR35

MVVLSLGISSEIGTLKFFYTFIYIKKVQRIVREYLECDHMVVPGSRFADNVLKTMRNVKKRAILYWV
VVIGNGVVYVTKPLFMSGRHHMEDRYIVYGLEPMFESPNYEVAYFLMMFGLCFICYPPANVTVFLIV
VVGYTEAQMIALGEEMLRIWEDAVAHYNNKYHTVGALTNSSEKNKIINQYVKFRLTEIIKMHTTNIQ
LLRQVEFVFRSAIAMGYVFLVLGLIAELLGGLENTYLQIPFALIQVLVDCYTGQKVMDASSLFEQAVY
DCKWENFDKSNMKTVLLILQNSQKSMRLSVGGITVLGFSCMMSVMKSIYSAYATLRTTMS

>BmorOR36

MVFNSKKNIISLFSLLEDSRHPSVGPHLRLLSLTGIWYPNSKTNITLLKRACFYVIVLFFVSQYLKCIIK
FKIDSLQLILEYAPFHMGIVKTCFFQKDYNVWQDLVSFISKTERDQIAKKDPKSIKTIQSYISRNRKITY
SFWALAFIANIGVFSKPYQNNQSDVNGTVTYNHLFDGYTPFSEEPPGYYFSMGIETILGHVVSFYVLG
WDTLVVSIMIFFAGQMQMSRLQCSRMINGSPERTHKNIIKCHKFHTDLIKYQKQFNSLISPVMFVYLF
VSSINLSVCIVQIAEIEDDFATVLSSFIFLLACLIQLLLFYWHSNEVTVQSELVSYSTFESNWTSTQNKL
QKEVALLGLTTSKTLVFTAGSFNHMTLATFISIIRASYSFYALLNSTKY

>BmorOR37

MELGCSRHLKLPCSLHPIGISKHGNTLSELLIYFPAIPKITYAILAVLLTVYYYIYLCSITWFVFVRCPQT
GDLAAASIVFSLGVSSEIGAIKLFIMYVYRAKLRDITGEYLQCEADMAPGRLRARVGRSLRTVRRRAF
VYWLVLVVNAFAYDLMPAFLPGRHLSEDVFVIYGFEPMFESPNFEIASTLMGVSVVFICYTAGSISAFL
IVIVGYSEATMLALSDEISCVWDDACASECQQPNDFIRARLGKIVAIHTKQIRLIREVEVVFRGALAGG
FACVAFGLIAALLGGLENTFLQLPFCVIQISVDCFVGQRLRDANVAFETAVYNCKWEYFDKSNMKTV
LLILQNSQKTMGLTAGGVAALDFTSLMTIFKSVY

>BmorOR38

MVVFSLGISSEIGSTKFFNTIIYIKELRKLFKDYLLYDATCPAQGRLRLHLLTTLRYVKRRAIIYWLVIIG
NGFIFAIKPLLVEGRHLAQDDLVLIGLEPMRQSPNYEIAYAIMTMGVCFICYPPAHVTMFLIIIVGYTEA
QMLALSEELKHLWNDAIEHYEKHSRTEREADAAMKSKILNSFVNFRLVQIIKSHSTNVNLIGRVENVF

RGSLAVGYVFLIVGLIAELLGGLENTYLQVPFALIQVAIDCFIGQRVNDANIDFEKAVYDCKWENFDK
RNMKIVLLLLQNAQKTVSLSAGGIAKLNFSCFMSVIKSIYSAYTTLRTTMK

>BmorOR39

MLWSVFSYFTRADDVLAGIVIFSLGVSSEIGLVKLCFMYANIDKIQKITEGYLKSDAASARNSRFSKNI
LHTMQSVKKRGVIFWLVIISNGVVYLVKPIVTPGRHFMEDQFIILGLEPKYETPNYEIGFFMMAVGVC
VTCYLPANITAYLITVAGYSEAQFLALGHELANLWPDAQLHCRAMNLSQSVNEQANEYVKMRLREL
VKIHSTNVNLLRDIEGAFRGAIAVEFLLLIVGLIAELLGGLENTYMQVPFALIQVSVDCLTGQRVMDA
NLALERAVYDCRWEEFDASNRRVVLLLLQNAQKVATLSAGGIATLNFSCLMAVIKSIYSAYTTLRTTM
K

>BmorOR40

MTGAGAGTFRTGAGPGRGDGVARRGESGETTTLGRDAFAALGCFGAADGSTARARFFPRVTVLNPS
EVPGSGLAADSNSISDSESEPELDAAQDAIDAGAGVGGDIGESRARTVFGIQGHDASDSALRMHNNV
AIYAKTTMSGNSQLTFATAATIFLKNASGPNGVAIGTDYAICVVSLSLFFCYRFTELVEDTYNSYLLFQL
VGSVGIICMSALRILVVDWRSVQFFSILCYLSVMISQLFVCCWCGHELSATSEELHTILYNCAWYDKD
VKFKRDLIFMMARARRPILLRAGYYIGLSRQSFVSVSIPRIRFNAILVI

>BmorOR41

MMGNSTDLFLDRTKSILNFFAMWRSFEKPIPLKVYMAFIMTTQYLFLIFEIIYIVNVWGDMAEVSEAS
ILLFTQASVCYKITSFISKTNNFVILLGLIESEIFSAQTELHEKILILKARKIKRLCMFFLVNAVTTCSLWA
VIPLLDISSKMLPFKIWMPASTGESPHYELGYLYQMITIYISAFLFIGVDSVPLSMIMFGCAQLEIIMDKI
GKVKSRPLDQQPMQRQAVLNSNYELLVECVRRYQSVVRFIELTEKTYHANIFFQLSGSVLIICNIGFRI
AIVDSNSLQFYSMLTYLVTMLSQLFQYCWCGHELTIRGEELRETLYQSPWHEQDIRFRKVLIITMERM
KRPIIFKAGHYIPLSRPTFVAILRCSYSYFAVLNRVRNE

>BmorOR42

MDIPKFEELLKQIKMNFWLMGIPFDNPKIQIRYYVLLLPLSLMLIEEIAFFGSRMSSENFLELTQLAPCI
CIGVLSVLKILALTAKRQKIYELTQNLECLHKIILNDTRKTELVRKNLVLIKFITKYFFVLNAVLIFVYNF
SSPVIIAYNYIVSNEVQFVLPYAVLLPFKTDSWIPWLIVYVYSIFCGFTCVLYYATVDVLYCVMTSLVC
NNFSLISFKLQKVNRNTAHLLKEVVKEQQYVLKLAEDLENIFTAPNLFNVLIGSVEICALGFNLMIGD
LTQIPGCILFLSSVLLQILIMSVFGENLISESSRIAEAAFLCKWYEMDQKSKKTILTIMIRSHKPKKLTAY
KFSVISYGSFSKIISTSWSYFTILRTMYTPPGTKFQDDL

>BmorOR53

MALKKMLALTKGLEDPTHPLLGPTLKALSVFGLWQTGSQKSTVIYNTFHFLTFLFVITEYIDLYTVRK
ELSKMLNNLSVTVLSTICMIKTLSYVCRQSHLKVLVREISELELELMKTTDKNIVKRLRQYTVYTRAV
TYVYWFLVVGINVVLLTSPLLKYASSEIYRSEIKNGTEPPPLILCSWFPFDSARMPGYFWATMVHIIMSI
QGCGVVATYDMNAVAVMSYLKGQTSILKDKCKAIFDETASSRDVLNRIRDCHRHHNILLRHYYMFN
SLLSPIMFVYMLICSFTICCSIIQLDSSETTISQRIWIIQYSIGQISQLFLYCWHSNEFAAKVKKKHFPLFPI
NLF

>BmorOR54

MGLNTIKEFFVNVKRRFQDVSIDSLLWIVNIVPSLAGFSIRSDRVSAPFWIVHWSLLVYVYAVGNAVY
QWKFANEAIDYITSFINVSLLILIGNNSWWFLANRRLLKSVLHKIEVNDELSRRSEQSRLKHKKLLKII
KRIVLVFYMSNYVNASFIYLPNRVDVLNNYAMTPCVGMEPLTVSPNRELCLTILCMQEFSIMTVVLNF
QALLLCFIAHTAVMFQILADEIMALNNYENLEEHQAYVKEMLPIFVKRHSLTLSAVDNYKSLYSVPLG
VNFGSNALTILLILYLPVLEWFKFIPIFVFCFMLFFLYCFLCQKLVNASEAFETAIYCCGWENFALREM

KMIYVMLHQAQKPVELLAADIVPVNMNTFATTLQAMYKFVTVVKF

>BmorOR55

MCFLKIKQQIIDIQKHFKDYSLNGSLWIVNLLPRLMGFNLRADKVGVFFWTIYILLLVYVFGIGIFVYL
WKHVDTMSGLMKSYLNLSLILVIVNNSCWFLSKRSLLNKVLKKIHLIEDLSCESEHALAKYRRVFKI
VTHLLLASYVLFYFTEIYFMFLFRNYDLLEDYSLAPCVGLEPLSSSPNSEICLIIVLIHEFISTTVMMSFA
ALFLVLIAHTAVMFLVLAEDMTKLTDLINLADHRKMIRESLRSLIHRHSLLLQIVYELRLLYSVPLGINF
ISNAMSILVLLCLPIHEWPSFLHIIGYCFFAFFLYCFLGQNVINASEKFIDAIYCCGWEHFGVAEKKLVH
VMLRQAQKPVEIIALGMISVNMNTYVEALQLIYKFVTVLKI

>BmorOR56

MKLLEKLEDPDRPLLGPNVKALKFWGLLLPESRSKKYFYLFMHFAVTVFTATEYIDVWFVKSDLALL
LNNLKITMLATVSVLKVTTFLLWQNAWRDLIGYVSRADLEQRATSDSRKLALINGFTGYCRKITYYY
WFLMYTTVAIVTVQPIFKFFSSAAYRLDVQSGNGTYLQVVSSWIPWDKNTLPGYLLASIYQTYAAIY
GGGWITSFDTNAIVIMVFFRAELELLRIDCAALFDDEKSFGDMAFMRRLKECHRRHTELVKHSRLFD
SCLSPIMLLYMFVCSVMLCVTAYQITIETNPMERFLMTEYLVFGVAQLFMYCWHSNDVLYASQDLSR
GPYESAWWSRDVKYRKNLYILVAQFNKVIVFSAGPFTKLTVATFIRILKGAYSYYTLLSQSQMNKT

>BmorOR57

MPSLIKNRIFGLTLTLNTLSWAGLILRDDYTKTQRIIMKVYGGLVFLYLFVFTAYVQIADLVVIWGNID
FMTETSLILFMQLAVSAKVLTLMLKSKKIMEVTNEADAILISEKKVEGQRIIASIDKNTTLFLKYYGFF
VAFTIICWFMGENTSTFFIRSKYPFNELKSPGREFAFVHQCIVVIFTGSFDFNVDIIIISLVAVCRCRLKLV
ALSLRNLCLDIPMNKRNLITSDEEKVITERLRNIISQHKRALDAAEAIKHYLSGALLVQLMVSIVVICT
TAYQLAVKKSTTMQSLTMAGYLFGTSLEVFLFCYQGEFLRESSEEIADAAYECPWYTLTRPLKKTLLII
MTRAQRPATLTAGGFVTLDITEYMAVSLISNT

>BmorOR58

MKLVFDNFIFALKVTLNWCRYFGIFIPDELTGRRQKLLVQAYSVFMFMLFIGFFIITQIILFILVWGDLSL
MTDVGLVLGTNLALSAKIAVFFFKREELASILKKNDDTLRFETREEGKKIISEYPCDTKRSPAYEIIMIH
QTIAVAVIASLAITADLLMLSMIAVCRCRVKLVGLYLQTICDDLPCNVKNKLTSDEEVIVAKRIREYVIE
HQAVLDCISELQNHFSPALLVQLLTSVVIICVTAYQLAVEKSSDMLRKFTMASFLFGMSTEMFMFGYQ
GGHLSHDSMEVATAAYSCPWYTFPTSLKRSLLVIMIRAQQPALLTAGGFTTLSLETFVTVS

>BmorOR59

MDTNPSAAGDSVAPHLRRLRQVGFCQLDPTSQSRRPILALMHRVYHRLVLAATVLYIFEQLTYAYQA
RNDMERLSRVLFLMLCHLTCIAKQFVFHSDADKINQLVVGLDDALCNQPVETHRLLLLLETSRRAARL
LMLYSGCAVSTCILWAVFPLLDQLRGRTVEFAFWIPIDYRHNAFQFAVVLAYAFYSTSLVAVANTTMD
AFIATVLYQCTTQLRILRMNFESLPERAYALSRKTRQDYHTVTHELLVDCLLHYKKITETCNLLEQIFG
KAILVQFGVGGWILCMAAYQIVDMEILSIEFASTALFMGCILTELFLYCYYGNEVTVQSGLVSESVYA
MSWLSLCPRERRALVVVLERARRPLRPAAGRVVPLTLNTYLKILKSSYSFYAVLRQTK

>BmorOR60

MVRPCRYFAIHFILLRFLGLGWWHHPHENETRNYPGLYLYYSILTQLVWVVGLVGLETIDPFVGEKD
MDRFMFSLSFVITHDLTLIKLYIFYFRNVEIQDIVRTIEIDLYRYYQNDDKIRATIRISRIFTAAFLFFGWV
TIGNANIYGIVQDLRWKDIVKNLNETTSKPLRTLPQPIFIPWPYQEDKHYILTFILETMGLLWTGHIVM
TIDTFIASVILHMSTQFAILREAIVTAYDRTMIALSEGALQSGVLCENSNGNEENNQIFLESFYSKEHIES
VLESTLLSCIRQHQLLIGCVEKFSKTYSYGFMTQLLSSMAGICVVMVQVSQGASSFKSVRLVTSLAFF
FAMVIQLAIQCFTGNELTIQAERIADAVMESKWEKMPVRLRRLLLVTMMRAQRPLHLTAAGFAYIDN

TCFLSILKAAYSYYAVLSQKQG

>BmorOR61

MARITDVFRLNFIFWKFLGIWGKSAPSKYNMAYTALYLSASLFVYDIFLTLNLIHTPRKLETLLRETMF
YFNHLVAMTKILKMFIRRKKILVIFDLLDCEEFKPSDEDSQEIMKRKNEFYYIYWRIVAVTSNLSCFMQ
VVGPLIKMLIWKSELGLPVCKYYFMSDEFRNKYFVIWYIYQSFGIYNQMVNNLNLDTFNCGMLWM
AVGQLQILKTKFVNFKLNDIENSLDLKTRDDMQTERLRKYLTHYEIILKYCATVQDILNITIFVQLGMS
SIVICVGLCGFVAMPSNTETAIFMSSYLITMTMQIFVPSWMGTQISFECGELMSAAYCCEWIPRSKLFK
RSLILFVERAKTPVRITGLKIFTLSLDTFTSIMKTTYSFFTLIRQLQVDEVN

>BmorOR63

MKLWIRNANFTISLSLTLLRCLGFWSPDGLAGNKRLLYNCYSFVFFMFLLGIYILIQVVDMIKIWGDL
PLMTGTAFLLFTNFAHATKVINIVIRKNRIQRVIQQANAVLMGVQSEEARRIVKSCDFETSIQLCLYFLL
TFVTTVGWATSAEKHQLPLRAWYPYDTSKSPAYELTYIHQVAALLIAAYINVAKDSLVSSLIAQCRCRL
RLVGLALASLGQDLKIDYQSQLSPAQENILNLRLKTCVLEHQTVLAAVTELQACFSKPTFAQFTVSLIII
CVTAFQLVSQTGNLVRLLSMGTYLMNMIFQVFIYCYQGNKLSVESSEIAGSVYFSPWYLGSVKLRRA
LLIVMVRSRRVAKLTAGGFTTLSLASFMAIIKASYSLFTLLQQVKQKK

>CpunOR1

MRAGGWCIHSKHLSANMTRPFHLTNKSTRYFYKMCNIVYLTGFPNFWTEDLGYSETFVKYYKYFTK
LVTSILYVFIVTEWAAFFTQHNMTPKQSSDSILFNYSHPLLTSYRPIMIYHQDKFRELIFNLCVKLKEVF
NDEKMEQEMIKKSKMCVASFTTICSMSLIFYGFEGIYQVLMSDATFTTV

>CpunOR2

MDASENLKTNHPQKYFLKLICRSLYYIGMGDYWYEKTERTKVHKKMYIVYAVLINGYLLLSTLNQL
LAHFRTDLTLKEKNDLIQFSLAHPSLCAKFICLFLQKDRLKVLFEKLIEGDSYTFRSLDVEKVSVKKAI
RYFVALSTGTYAAVLFSTIDGFLSSKRDGTPFQTEVSLIPTGSQLGPFWSFIRCLHVLHWWCIVTNMLL
VDAISFTSLIFLGYKFRLACMYFEQLRVKTKDNCKSKSDKDCMEEYEKGLITGIKLHQDALWCARTV
QSSLGIIYGVQIIETTSILVMCMIKLVATRGSFTFLLANFFYIACLLILNGVYMIAAGDITFEASPLSTEIF
HSGWDLLKSRRRFRTLIVLAIACSQKPVYMTAFGVITLSHENFISVLRSSYSFFAVTY

>CpunOR4

MEPTQSQVFDLNIFLWKIFGMWHHETSTIYYKYYCVTFQMLYSVTYTFLFTLSLMFTPNDLELIISQC
MFYFTQLAGVTKIAMIILRKKYIFEAFHILDCIEFQGDDVETRKIVNENKIFYKKYWKACFVCYNSGG
FFILCLPLINYVVRRTELDLPLCQYYFLSDHTREKYHSGLFFYQFTGLVVIILSNISTDTFIIGLLMMAIT
QFRVLNWKLTNLNFSSLDEELVTMVDKEVLFVEKLKKCLVHYDLLQKYCNIIQDVTSYSIFAQFGTA
AVTLCVSMCTFLRPMTNNDFMTSVTYMTSMAIEVFLPAYYGAELTHESEKIVMAAYSSDWIPRWESF
KGSLLLVMERAKRPVVITGLKMFTLSLETFAAIIKMAYSIFTLLKNAQN

>CpunOR5

MNLVQRKSNYQANSFNGAYNRKESDLIRKQLIKCIDHHNTIVSFAGCVSELFGPVLAFSYLNHLVCG
ALMLLECTMGDSDTVMRCLPITIITFSQLAQISVTFEIVGSESEKLIDAVYNTPWECMNTSNRRLVSMF
LAKVQTPIRVTALGMVDVGVEAMAAILKTTF

>CpunOR7

MTSLAGGSVAPHLRVLRLVGFCRLRAPAGRAAPGLARRLHGLYCWFALAATSFYVLQQGIYAYQER
HDMVKLSRVLFVLLCHCTCISKQVVFHAHAPQIDALIRSLDQPLFNQGGAAREALLRRAASRAARVL
RAYYGCGVATCVLWTAFPVIYRLQGHYVEMPFWTFIDYRKAFMFVVVVSHSFYATNLVAVGNTTMD
AFIATVLYQCQTQLRILRLNFESLPERARIASKELSKGDYEATLNKLLLECILHFQKIADTMELLQRVF

GLAVLVQFTVGGWILCMAAYKIVSVDILSFEFASTTLFIVCILTELFLYCYYGNEVTVESDRVVESIYS
MEWLHAPLRFKKSLVLMMERAKRPLRPAAGRVIPLSLDTFVTILKSSYSFYAVLRQTK

>CpunOR8

MEFKQIDCFDRNKKFWKFLGLYPDIDIWPYYHYYSIFFIFFVIILYDFLLSINFYFLPRQLDSFIEEMLFY
FMELAVTSKVFTFFFQRGKISKALASLESDIFQPTTNEGVEIINKAKKFNVRYWKIVAVVSTTSNFTHV
LTPFFVHLFLPVKLELPVCSYSFLSEDTIQRFVYPIFVYQGIGMHFHMWCNVNIDTFFLGVMIFAIAQL
DILDIKLRSLTDNIAEDTDVDKNLNLKLNEAINHYSEVVKFCYLIQDIFSVTLFVQFSMASCIICVCLFR
FTLPATADYYVFLATYMFIMIIQIMVPCWFGTRIMDKSCLLASAIYSTDWTPRSKRFKSSLRILVERLN
HPISIVGGKMFPLSLETFTSIMNSAYSFFTLLRHMQTRDR

>CpunOR9

MVVLYVGYKRVMILRSIRKFDDYIYAIIFVVFLVPHFWIPFVGWGVAHQVAIYKTSWGKFQVRYYRV
TGENLQFPNLKSLIVVISIGCLLLAIGFLLSLCALMDGFLLKHTAAYYHIITMINMNCALWYINCKGIKI
ASQSLSDCFRRDVRRECSAKLISRYRYLWLNLSELLQLLGNAYARTYSTYCLFMFTNITIAVYGALSEI
VDHGFAFSFKEIGLFVDTVYCSTLLFIFADCSHKSTQKVADGVQETLLTIDILAVDRPTQKEIDHFIQAI
EMNPAVVSLKGYADVNRELLTSAISMIAIYLIVLLQFKISLPKDPQSPTP

>CpunOR10

MSDHTEKPNGISSCNLNQNLDNVEAKEGMQKEILFDQSLQKIKFAFRLTGLNIQNEKRSMKQNCVYL
FNFLWLNTDIIGALFWVIDGMLSGKNFTELTYVAPCLTLSILGDVKAIYLVLNESKVHTLIANLRKLEQ
KANEFENFEQDKLIEPDIRLFNVVIKVLNVLNCLMIVVFDLSPLIIIAVKYFTTGELELMLPFLDVYPFD
SFDLRYWPFAYIHQIWSECIVLLDICATDYLFFTCCTHIRIQFRLLQHQYQEIIEPVNLSAVGTIDRSRIK
TKFKDLAVWHQEIINSASMLEGVYAKSTLFNFMTSSLVICLTGFNVTTIDDKAFVITFIIFLFMSMLQIF
FLCYFGDILMSSSMEVTNAVYNSRWYLSDVGMGRNVLLVQTRAQKPCKVTAAEFADVNLRAFMRIL
STAWSYFALLQTVYSSRSK

>CpunOR11

MQSDLSKEQYKNSMMLQIREFGPAYSDLPTMMQNVAKLLRVLTLNIDNEYNKPIPIHCYILTIATAAA
YTYVFAISAGWFVVKRCIPNHDVIGAIILFSLSISSEIGSAKFFYLYLYRDIVRKIVRQTLEFDAGIVPGS
RYSKSVLQTMRKAKVRAIAFWFIIMGNGVVYVFKSLLTPGRHFMDDVYTIFGLEPMQKSPNYEIGYA
LVSAAVWFLCYVPANVTALLIVVAGYVEAQMVALTQELLHLWPDAVQHYQDVQFSTFTSRTNTKSR
QRILNDFINIKLQDVIKRHSKNVHLLQMLENVFKGAIALEFTLLALGLVAELLGGLQNTYLEIPYAFVQ
VAMDCWTGQRVIDASTSFEAAVYASNWEHFDVANMKIVLLMLGSAQKTMTLSAGGVTMLSFASLM
SVVKSIYSAYTALQSTIK

>CpunOR12

MDVPTFEELFRQLKFNMWLFGILFDDYEIAFRFYCFVLSMFSMLVEETSFFISRYAPENFLELTQLAPC
LGIALLSVLKILPIAAKRQKISVLTKSLNKLYNAILTDPEKREVVKKEILLVHTIIKYFFILNTILITVYNF
APLAFMLYAYLKHNRIEYKVPYAVSLPFALDSYPKWFIVYFHSIISGFVCILFVTSVDALYCMLTSQICS
NFTVISNEILHLDESGVHRLKDLIIYHQQVLKLSDDLEEIFKLVNLFNVVVGSTEICALGFNITTGDLG
HLPGYILFLSSVLLQILLMSVFGEKLIRESTKVGDAAFNCKWYDMDLKYKKMIVFIIARSQKQQQLTA
YKFSVICHRSFTKIISTSWSYFTILRTVYTAPPETVQLE

>CpunOR13

MLSIKKKIISWLKKLEHPKHPLLGPNIKILYLFGIWQTPRTKIRNRLYNIVHVSTFFYVLSQFIDLYKQR
NDFNKALNNLSLTSIGVICSAKCIAYVLCQPQWQKLVAKISEEELTQMKKRDVKVVKKMEEYKLYAR
VVSYMFWVLVLLTNITLIGTPLLKLLISSTFRENIHKGLEEYPQIMSCWFPFDYMKMPGYAYSCVIQIV

MALQGSGVLAGHDANAITIMTFMKGQMQILREKCVKIFDSDENEGPNEFFIRMKECHRHHNFLLEQ
SNLFNSLLSPVMFVYILICSMAICCSVVQFSSEEATSEQKLWAVQYTLAQIAQLFLFCWHGNEVFVES
KDVDMGVYSSDWWKADARLRRHVVLLAGKLNRPILYSAGPFSDLTLPTFVSIMKGSYSFFTLFSQM
QEDN

>CpunOR14

MRFNFLRAAAEPKETSTLDGDPIRNYIHFLETPLKIVACWDWYRRPQTDREIIINNVYLCLVLFVLVN
VPTSLYVHLYLDWVDVMTSLHKLADCLPFIVSIVIVIYFSIYRKELYELTDFMMENFKYRSANGLTNM
TMLNSYKKAKGFARFYTACTLFSVTMYTLPEIVNWWTDQPLLSHLYMDVTRSPFFEFTFMRQFLSQ
AFVGLAMGQFGVFFAANSILLCGQLDLVCCSLRNARYTALLRSGAAHAALARAHADVLLDERHSYI
YNVAEMKESAYHYDQKMSRIFTDRRTHFDLYSAEFDAATCAALRECARVCGAANRYRERFERFASPL
LALRVVQVTLYLCMLLYAATAKFDMVTVEYLAAVALDTFVYCFYGTQIIIQADRVTTAAYQSAWPAM
GATPRRLLLNILLANRRPVAVRAGMFLPMDLHTFVVIIKTSFSYYTLLVNVNEK

>CpunOR16

MAKSFNIINRADFTFKEVFFITEYAMLINRSHPFIKRNLFWLFQYLIIFTLSISASLLLINSILFYDIPSSRY
TDASKNGTMAIVALTVTIKHAFLLYYQADIKNLINIVNKDYELALNFETVKKEIVMKYAKRGKKVTL
FWLVAAAVTSIVFPANALLKMAKTYWHGAFEFIPMFELQYPDCINAVKDTPTFFYLLFVYNFLFGFYA
TTVYIGFDPLAPIFLLHICGQLDILSYRMLTLFDDAEVQDVNVKLKFINMKLQDLYSLVNNIKSMFTV
LFEFNMKTSTFLLPLSLFQVTESLKEKQLNVEFFSIFAATIMHFYMPCYYSDVLLERSDNLRRAIYFCG
WEKQTDIRARKTILLMMTRTTAPLVLSTVFYPICLDTFAEMCRQSYAIFNIMNAAWA

>CpunOR17

MWPLKRRTKVAPNNEPKVFSAEDYDGTFFHIKRVLRLLGIRLLCEDRPLVKMLWDVFYWFEYVAIAS
TAMQDILLVRDVMMEERNFEGALKIFRMIPCIGYLLQAMLKTHNIVYYRSTFENMVNELRSMWPQG
TVSEEETTVLTESLKETKTFVKVLFYSNYILVLSIAVPPYVDVVKRFFWKDTPKILPFTNYLPYDPSPP
VLFELTLTLQIWETIITVWCVLLGDLLFCVLLSHVTTQFELLCIRIQRLFRVSIDDQLIATYPLAENNHE
TSATNNDEYTSFERKMMYQREVAEIVTRHTALIRLAGDIEQMFSFAMLFNFFYNSIIICFCGFCCVLIEK
WNVFMYKTFLITAFIQTWLLCWYGQKLFESSQKVADALYNSGWYAAPKEVKKSVLIMIQRSQKSVN
VTTYGFSIICLINYTTIIKTAWSYFTLLLNVYTPGD

>CpunOR18

MDGFEIYHKEFLKPLIVTLKMLNQFNVRFCSSEVPFLTRNWRFFYLVPLWILHIISLMSFIVNLIMEGN
DPFEEAYMIPICIISFEHSIKMCILIKKREEIRDVITNIGNIWRSTGLTDDQKKTKEEILKKLYNAGLVFN
NIGIAVTIQYLMVPLFETLVRRLILKQEVELMLPLTIIYPFEIKTWPLYLVIYGFQVYSLLCAACVYLGF
GWLLVVLTCYLNIQFLLLQEDLIHVKPETSNRLSYLPEEEGEITQYWEPSPFGIDDFVRQHQTVILLTD
KLNAAVNKITFTIILFATLNISFFAIAVKASAGAVDMVNNFGAIIVIMMNIFILCYCSQLLSSSSSGIALA
AGKNLWYKGDLSYQTKIRFIMMRSQKPCSLTSLDFSPIGLETFNKVLKTTWSYFSLASQMYDERY

>CpunOR19

MTDKEDFDKSIKLSKTFLLLSGIRLTEIKWNRSIVTFIDRYLFYFNLFWLYTDVWGEINWLCEGLLKG
TSFIDLSITVPCITISLLGTSKALFLINNRETLLKTVDKLRNIHPVDNDDFDENSNHFRSFDVWNSDVTK
NEDVVRKIVRESVKFVNFIGTLMYYICSVVICAFPLMPVTSMAYDYYTTGQTEWKYPFLVNYFFDVN
NVYMWPPVYVHHVISTCIVGANVIGPDSLFYALCIYIQMHFRLLCHRLENVVGAGTQIRRELISAVQR
HQELIAIVDQMEIMYSKSTLFNILTSSLLICLSGFIITVLTDFSVVIAFGCFLIMSLTQIYLLCFFGDSLMR
SSTQISSAVYNSLWYETDQRSKKIMLLILMRAQKPCKLTACKFADLNLTAFTTILSRSWSYFALLRTMY
N

>CpunOR20

MLLKKFKAFYNKDGFDYSVGYVDPFGYHHTFIYMMRAFQVSGEPFKTWTYVSKIFTLICGIGVLTD
ACLSFYHAVDIFDMGLITEAGTYVLMLLYKMMNLIITKVNLSEYIKLMQAMKDDFQYINTKNEKYK
KAFFKTQHDTFKACVVTCTFMFVLATSLVLFAIGSLLFYLATHTPGDGTHKPLVFPFWAPGVDYTTSP
AFECAFTFANIGVMACTYNYTFVLQTNIVWVCQIASKAELICMCIKDLLDGIQPACNEEEKRHYASVI
NFRMREIISLHQSMNRLIDSYACVYRKCLMFEQFVSSGVICMLAYCSAEKIDAGDIHVVMMVLCVGA
IVILYVPCYLCTYMREKVTLICDACWDIRFWDAGPNIRPYLILIMQRCLRPLPLQAPGFQEVSVKTFSS
KITSAYSLFNMLRQADLDL

>CpunOR21

MRQRKGATYHKASPTYNLRKQITQTINNTGITFEDDRIKWHWIASVSVICFVIADVAMIIALFSVDDE
ERFFECFSVLCFCFVGFLKFLSLNCNHKQWSSILSNITALEKEQISYEPASVEYESDDENETFNFPQYIN
SYTAQFQAVSTFLFRMYITTLIIFAVSPFVEYAFLRILGEHVEWLRILPGWEPFANKSFVGHVINIIIEIIGS
CYCVILHVAFDLTSIGVMIFICGQFSLLRVYSENIGGKGRHWQLSKRRDERAHQRIIRCHKIHGLLMNT
SNELSKLLTNVLGVYIFLATLTLCSVAVQLNSELSSMQLVSLVQYTCATLTQLYLYCRYGDNVLHESSI
GMGQGPFGAAHWCLSPRTRRELALLGAGMMLPRYIRAGPFTRLDLPSLINVVRTAYSYYAVIRQ

>CpunOR22

MNGNEIKPDPMSLPYMRYTRLMLQSVGAWPGDAVAGRALRPRLYAYYLMLETAVLIAGELLFILNQF
HVLSFFVLGDVYIALSLSFLVMFRAVLSCFKRYGTIFRKFITEFHLMHFKHKSEYSNQMYEKVNKISH
YFTLFVIGLSVSGVVSFNVTPLYRNYRLGVFRDEPPENVSAEFSVHYLFPGFRQEDFYLPSTLANVFIS
YVCAFIICTMDLFLCLMIFQIIGHIKTLIHTLRNFPAPKKMKELQGFRQGSMNKAPIEVEIVKQFDDQE
NEVIKKIIIECVEHHLFIVSFTDDISNFFGPMLAFNYLYHMFSFSLLLVECMQGQVALMRYGPLTMITL
AQLTQLSVTFEIVGSESEKLKDEVYYIPWESMNKSNQKSVGIFLKRVQTPIRVTAMGMTTVGVQTMG
GILKTTFSYCAFLRSLNP

>CpunOR23

MMTKVKALGLVSDLMPNIKLMQAAGHFLFNYHSDNSGMAMLLRKIYASVHAFLIVIHYLCMAVNM
AQYSEEVNELTANTITVLFFAHSVIKLLFFALNSKSFYRTLAVWNQSNSHPLFTESDARYHQLSLTKM
RRLLYFICGVTVLAVVCWVTITFFGESVRMIANKETNETLTEPAPRLPLKAWYPFDAMGGTMYVVAF
VFQVYFLFFSMAIANLMDVMFCSWLIFACEQLQHLKAIMKPLMELSASLDTYRPNTAELFRASSTEK
SEKVPDPVDMDIRGIYSTQQDFGMTLRGAGGRLQNFGGNPTNNPNGLTQKQEMLARSAIKYWVER
HKHVVRLVASIGDTYGTALLFHMLVSTITLTLLAYQATKINGINVYAFSTIGYLSYTLGQVFHFCIFGN
RLIEESSSVMEAAYSCQWYDGSEEAKTFVQIVCQQCQKAMSISGAKFFTVSLDLFASVLGAVVTYFM
VLVQLK

>CpunOR24

MKAPCIIPELDTLKEVKINDPRYWPITFQRDRNWDQLKAFLEYTWCIDIKQRAGCLSYLDDPQGFLTI
LAQTLTLDKANIWSPTPDNIELFTNDDKVRNFVKHYLSKDTVTNVGANICADCLLVGKKRKGVKNE
ALLTKQCLCRRYSREEQEHVQALSMVTYECVVKDILCALPIWTTFLKIIKTMKTEPSTYHMWQIKLL
LSQIENHNRRAMDEMKVDGQEAQTEPLISTEFTLAIKQKVANTFDKWEIKIIPYLRKYLGLPSSRKISS
CEEDMKKILSAYLVYHDLPRGVLRHVVSENELGVLMDLESMNLSVDALSKIELLLR

>CpunOR25

MKIIKNAQLVNNKRENIDITPPRDQLMYKILANVLELYGLASMKLWNIPPYHVYLNKFYAINSLLIYT
SPLCILSQLLYLYVHFNDMSFYTLGVMFSVLPIGITVLIKIASTRTNAYKKIMVNFMNKIHVHNQHLEE
PDNEFIKSVVKQTENFTHLATYFLAFCLLTDWTAWTAIPVIDYLKNKELIERKEKKLETCLYLYTPFDY
SYDVGNWLIVHTFGSYLNFGGITIIIIFDTLCFIFVFNLVSHMKILQHKMERICKIDEMTNEDMRKKLIE
IIEYHAQIINYFDDIQAAFGLNITAVYAQNLFVDSLLLYQIMVGVNGEKTHVIIFGLMFVAYMGGLTFM

SFVLEEIRRQSETFSDLIYSLQWEDMSKSNQTTVIMILAKMQPPLTFTGAGGLQTGVRPLVSIIKSTFSY
YVMLNNRTATR

>CpunOR26

MDGSSEKKEDPLQRNHIRVLRAILMTHGAWPIGESFMRTYFTAAVVVSVIINVFAAGHYIIVNFAELDF
IDIGDHCMTECLAILTLMRSIFVRVKDYGTVTEEFLTKIHLDHFRHCGENYDKICRRVNQFSYYVTLL
MVVIAALGLNFFNIVPIYNNFRNRNDEDAVTQYSIYLSLPGVNQDMIYTFSTIYNYYISVMCAVLVCGI
DLLMFVMAFHVVGHIMTLRDDINNLPKPKGKKDPVHMKNNYQTNLHVDIYDNEENAKIREELLKCI
DHHATIVSFAGALSDLFGPILAISYVNHLVCGALMLLVCTTGDRNSVVRCIPVTIVVFSQLAQISVIFEI
MGSESAKLMDAVYNVQWECMGANNRKLVHMFLTKVQTPIRVTALGMVDVGVEAMVAILKTTFSY
YTLLQSLGE

>CpunOR27

MTISLGVCLVFRNKFRVFLTEMAFKDEMLEMPLIKYAFKFGEGKKLMELKNMVMESQEKLLKYTRV
LLKSYVISVWLVATLYLCSPIYEMFAKGDPTLRLLAFDMWFPWSLEKLGVYIASFIFHAYAGYLCCIA
YPGLQLTIILLIGQIIRQLRIVTFIMRNLNELVVEIIKDRESKWQICCTAVLAQCVDHYIKLKRFSNRLNV
ICQPFYLTLILVAIMLVCMCSVKIAISNKLSPDTIKYYVHEFCFIMVVLMFCLLGQQVDIECELLELAVT
ENWYIFDKEHKMNVRIFKMALSQRMPIFIFGSIKLSLPTFTWFIKTGMSFFTLVMSVLEE

>CpunOR28

MKFSIENYTHTVGNKNEMNGIMRIVLVLQRFFGQHILNPDWTWKRFCLYQLATFALFSYVLAGTFAI
LQETDDIKMIAEATYTVVVTGVSFVKYCIITSKRFVFRRLYVELKSSLYDIVRDDSEEKMKKVFDDGK
KSVYYIFVFTLCPIIMYIARVLWYNLHGQKVTLSLTTSILMPMETPYYQLGLILHSIYFIEVSFIVIQVD
MWLVLFVFFFCVTSDITLKILTVEKRKQGEDRIQYAVRLNDSLKRFYRLHVKQVNFLSMLNGTFKWI
TLVTQVNICICICIVLLLVRKGAESAFALNILPSIAELIGFSWLGEQIKTKTNNWKMALLDFDWINLQQ
KDKKNYYILMCYMNQEFGLQSAIGGDLVLITVSKVLKFSYQVYTVLQSM

>CpunOR30

MVDLPVDQSLKKIKFLYRLSGMNIEDRDKNTTEKAVYMFNFLWILTDIILATEWGIVGFVKGLDFIEIT
HVLPCLNMGMVAEMKTIFLVVYEEKLKQLLRDLRDLERIRLPNNPYAKKIADTDAKFLHSLIKMTFT
VNICLLILFNSGPLVLIAVKYYLTGKLELFLPFLDIYPFDSYDPKYWAWAYLHEVWTACIVLSEIFAVDF
LFYVCCTHIGIQFKMLKRELEHLITGKGLLADHDDQLKEKLADIVKWHQQIISCAEMLEVIYSKSTLF
NYISSSLIICLTGFNSMVIDDMAIVISFFMLAVVEVLQIFFLCFFGGKLIDSSTDVSSGAYNSKWYLADIP
TRKTILLIQTRAQNPCKLTAAGFADVHLRAFMKILSTSWSYFALLQTMYGSSKVSH

>CpunOR31

MPDNHAYSYNVMQKKMLHEIKFLSLLGVATFVYPFIGRSKIVLGCYSFIYLLVLLTATQLIITLYLKDFS
DWVEIINVVPNLAVVLMAVLKYSKVHHNQRFYKKLFDHFRNDLWDAVSDCEQHRKIVTKYTDISRY
VTRFLFYYSVVLVIFVFSFPRFIMYLQKIITGEECHLYPFDGWYPFDKVSWYYVAYIWECFMTFVVVC
IYGFAGIFTSAITIFICMELKVLGSSIQMLISPSDAAKLTKSPNDKKIHQDIRKRLRSIIIRHQVLAKLSAD
FDGVLGDIMLINYVFSSVFITLTIFTATVVENLYMRMRYFFMFCSLMVEVFQQCMIGQILSNHSEELSE
STYFADWTYADNNTKIMLLILMTRTQRPFEYTANNYLAMNLQSFSSICSMSYQFFNLLYTAYN

>CpunOR32

MIFGSKKNKQENDIEEQTEYTFRPFHETYRWIATTLTLGLMYPNPATDRTRLILIVASLLMMQPVIVFIL
IDMYMCWLKRDIFNIIRHSTIIGPFLGAFFKMLLMYYKRVQVKELIDEMNRDYSSYNQLPRHCRAVA
AAGVRASVTHTERLWAPLVGIAIMAFPGVAVVGTLCSHVFAAAPQRYMVHDLNRPFREPEARFDSPY
FETLFLLMFAGAIICVFNYTSYDGLFGLMTRHACLKMSIYCLRLHDGFRSDEPDELYRRLMEFIREQG

RMFRFGALIQDAFNIWLGTILISTMIQVGSLLFHISAGYGFDLRYMLFSVCSVVHILLPCKNAANLKM
MSMDTATLVYCCGWERTSCKRVRRMIPFLIARAQRPIRIMAFYMFQYDMELFVNIMRTSYSMFTLLR
S

>CpunOR33

MAFVFFVVTGIGMYMNQDDLISLLSDLDKTIVSYNFFFKIIMFLIKRKQIRILIDDILHSGDKINEERER
VMKIHVILVSVLITTIIGGFQMLSQIKGELVISSWMPFDAVKNQWTVFMAGQILDGLYAVPVMYRAIAI
QGIVCSIIMYMCDQLVELQQRLKTLTYNEENGHNTRKAFKRIIKKHIRLVGYSRSIKSVFKEYFFIQNV
AITAELCLNALMMTIMGLEQKKHLATFMAYLMIALFNAYIFCYLGQELMDQSAGIALAAYESDWTS
WPVDMQKDLLLLITVAQKSFTLSAGGIAVMCMETYALALYNGYSIFAVLRDVVD

CpunOR34

MWQSARKFGLEHCDLETMLQNVNLLLRTLTLNIDSQNKKPIPWVYYLVTLVLGLCYYYVFLFCMA
WVVAWRSRQTGDWLGATIVASLGITSEIGTTKLLYMILYIRQIRELVDLYRDCDKLVSPESRFADNLLK
TLKVVKKRAIFFWMVLVGNGAAYICRPFLRPGRHLLEDSFTIYGFEPKFESPNYEISNLLLSGGIVFVV
YLPSNISAFLIIITGYTEAQMIALSEEMLNLWDEAQRHYQHRAATFATSSVLIQSREAKWQEYTAKKEI
VNEYIRSHLREIIKMHRTNIFIVQKVENVYRNLIAAEFILLTFGITVELLGRLENTYLQIPFALMQVAMD
CYCGQKVMDASVVFENAVYACQWENFDVDNMKTVLVMLQMSQKTMRLSAGGVTMLSFTSLASVI
RIIYSGYTALRQLY

>CpunOR35

MLEKLKRFGLGHCDLPTLLWNVAFMLKGLMLNIDKRYMNRIPTILYIINVILAVSYLYTYFFSMIWFV
FVRCVQTKDLAAAMIVFSLGITSEIGVAKFINTYIYRDEIRNLLQDCLELDSTTVSGSRYSMNLLQTLR
TVKKRALTYWIIIISNGVLYILKPIVMPGRTAMEDVFILYKLEPEFETPNYELAYFLSATGSVLTCYLTSN
MAAFLIIVAGYLEAQLHALSEELYNLWDDAELEYLNRHFSADIIDVNDVIDEKDINRLIHIKLRDITIA
HTKTITLMLKLDDIFRISFAFEFFILSIGLIAELLGGLDKTYMQVPFAIIQVGMDCLTGQRVIDASNTFEN
AVYACNWERFDSSNMKTVLVILINAQKTLSLSAGGIAILSFATLMSVFKSIYSAFTTLQSMM

>CpunOR36

MLRTYSSANNGTRYYHKLCNIVYLAGLPNFWMEDLGYSETFVKYFDYFTKLVTGILYMFMVSEWAA
TFTQHNLTPKQSSDSLLFNYAHPLLSSYRLIMIYHQDQFRELIFNLFVKLKEVFNEKNVEQEMIRKSKT
FVVSFTIICSMTLVFYGFESFLQVLTTDATFTTVITAWPDVEDKSTAAGVGRVIAYLIWWMLMSRIFAS
YIVVMSLMVALEYQYKNLREYFRSLQEIFEEDLSQSEMEMKYEEGVKIGIRLHSETLNCARLAQETC
STIYCLQIILNITVLVSLMSQMLDSGRTVSTVFTIVVTAVGTLLSTGVFMWTAGDVTVEAALLATDMY
ASGWQNCYERAVSVRKLLTVAMAQAQRPVTIKGLGILEISYSAYLQIVKSSYSLFTMLY

>CpunOR37

MSKAGVSVRPHLRVLRRLGFCGLLPAQPGAPGRGPLARRLHKIYCWFTLCGTTLYVIQHLICIYQERH
NAARVVRVLYPMFSFCTCVSKQVSFRMGQPRVDDLIIGLNDELFNQEGERYAQLLKQTAQRSFYLMR
LFYSCGVSTVSLWTLFPVVQYFQGKEVELPFWTPLDIEKPSMFAIVVAHSSYMVLLLSMGNTTSDVFI
GAMLYQCSTQLRILRASFETLPERAKKLSEELNENYDMILHELLVKCILHFQKIADMAEVILQVYGWA
VLVLFGVGGWMMCTAAYTFITVDILSMEFASAVFFIICILIELYIYCYLGNEVTLESDLVVESIYSMHW
LGTSHRFQKALVLVMERAKRPLRPTAVGLIPLSLDTYVRILKSSYSILSVLRQTNK

>CpunOR38

MDLFEHVKNTFSNVKTRLKHNTYADLLWLVNIVPSFAGFSILGDTLWAPFWIAHLSLLMYIYGVGCA
VYQIRDAQNTGDFIKSFVNVSLFVLIANNSYWFMMKRPLLKSTLADISKSDEMATVNVIFREKHERY
LMRIKCILLIFYGFNVGNAAFVYLPHRVDVLNDYAMTPCYGMEPLSSSPNKEICMTLLCLQELSIMTV

VLNYQALLLLLLIAHTALLYHLLSDEIMTLNNFNKDELYNNPAAKEFLPVIIHRHSIILSVIFKLKALYSV
PIGVNFGSNAVCISLFFYLPLQEWLQFMPILVYCFLVFFLYCFLCQRLSNAAEVFEMAVYACGWENFDI
NERKAVYVMLRQAQKPVELLAADIIPVNISTFATTLQAMFKFVTVVKF

>CpunOR39

MVTLKHFWRKLTHTKALENSSGQLEMAFFETVYRVSYITGFSAADHDIPYMIYSTTVKLLITMLVCA
EMWYAFTETSSLDGIAACINTTLIQLITMYRYGKMLYHKDVYRKLAMSMESPYFDISTEQRRNLVDY
WAKKNDNYLKLLLFLGNCSLAFWFVYPLLDELEYNLFIAIRLPFDHSSPKNYVLAYFFAITTFAYMSH
FVMGNDLLMQAHLLHLVCQFTVLSDCFENIVEDCTKEFKDADINSLIRNEMFQRVYKNRLRDMVNQ
HRSILSHVMELRDALSGPMLGQLAASGILICFLGYQTATTGVGNVTKFLMSLLFLGYNLFEFYIICRW
CEEITVQSEKVGEAAYCSNWECGLTVIPGVKSCLLLVIARATKPVVMTAGGMYNLSLMSYSSLVKAS
YSALTVLLRTRQD

>CpunOR40

MSELTLAEAKQEIAESLTLNTFCMNRIGLSFEEPKNTTAYLLQKCMFVLSVMGICYHVFSEIVYIGLTL
SNSPRVEDVVPLFHTFGYGALSIAKVFVLWYKKDVFKQLIYELAGIWPMPPMAEEDTMIKNDSLAAL
RMAHRWYFAVNVLGVWFYNVTPIIVYFYRVYQGQDVTVGFVWVSWYPFDKHKPIAHVFVYIFEIFA
GQTCVWIMVGTDLLFSGMASHIGLLLKLLQRRLETLASAEQTEEQDYREILSNIKLHQRLIRYCNDLE
VAYSLSNLVNIVLSSVNICCVVFVIVLLEPFLAISNKLFLGSALIQIGMLCWYADQILHANADVAAAAY
NSSWYRTSARCRRALLFLIQRAQKPIAFTAMGFTDISLVTYSAILTRSYSYFALLYTMYNDK

>CpunOR41

MNTMFLFLTNTDCIYKLIVFWVKPEQIDEILSVFKGPIFNQEEPEHRSILMKTVRNSHLMYGILKNMA
LFTCFLWVAHPTVFHIQGKYIEFPVWLPFDANKNPQFYIAVFYVWLQTSWLAYCNTTSDIFITFLLEQ
CRTQISILRLDLENVVEKSKNEATALSMSFQKVLERRFKKILLHHKTIVNTADKIQDIFGGAEFYLFFV
SGWILCTSAYRLASANPASIEFVSMIMYVICILMEIFLYCYYGNEITHETDMLMESAYAMDWLEMPVK
LRLSLIIFMERIKIPIRPMAGSLIPLTNATFISIVRSSYTFFSLLKNSQH

>CpunOR42

MEQSESPFDSFRVIIKLLTITGFYEIESSRKYVRAIHQAYRIFVIFIMLAFAIQHYIYTFQVADQDDKLYG
LIIGSPQINFMVKILTVHFKYGRIRELHVLIKDPIFSSTNKKDLEYIQRNNVAMQMLGKSVYYCITLAA
VTWSLVFIFKRIEDPVNASVPSYVPFDTTSTIGYSISVVLEVTPIFWIGFGQSAADCAVACYYSQARTQL
KIIKHNLEHIFDNEDEESILMEHDTSSNGRPKYIDEVNGNIKTKFISFIERYKMVDWYVTEISSIFDTSIT
CQLISSTLATCLVSYMLSLLEIFSVMFLHLFVCLIYFKMQAFIFCLFGHLVEDESKSINDAMYFSDWLS
VSPRFRRYIIISMTRWTKPLTPRVSTIVPINFVTFASIVNSSYRLYTFMKSSHIA

>CpunOR43

MSLDMLKRSNVMFCGPEVSFTKKYWRFFYMIPLLVLHYISLSAYILKLFTEGLDPFEKADMLPLWLA
TVEYWINTVILIINQDEIRNFIVHLGSIWRITGLNEKQMTIKTGILKRLYYVGIAVNKVGLAMSWQYLL
APLCETVIRRLFLKQEVELQLPFDCVYPFETKDWPIYLAVYAFQVYCIFRTIYVYQGVGWLLVVLTTH
MHLQFLLLQEDLVQVKPEIKGRPIKSVSEDADEITQYVDRPYLRIDDFVRRHQVLISLADKLDSSCNK
LTFTIMLFATLIICFFAVAAKASKGAAYALNNYGAVVVILMSILILCYCCQLLSSSSSGIAMAAAKNLW
YKGDLRYQTNIRFIMMRSLKPCSLTLLGFSPIDLGTFNKVLKTTWSYFSLASQMYDERD

>CpunOR44

MKRKLDPMSLNHLRWVRMALKTVGGWPGHAIDGRPHKVLCFTLFVAIECALVIVGEILFIINRFHVV
SFFILGDVYISMALSFVLLVRASIPIFERYGIIMREFIRHFHLIHFKYTGGHWEIIFEKINKLSHYFALFNI
TLTAITAISFNIPPLYNSYTRGAFKKNRSENITLQFSVHYDWPGFEQEKHFIVASILNFWLSYACAFIICI

MDLLLCLMVFQIIGHIQVLKHSLRNFPKPQIQTNLQELSTGEMNETRILIEVMQPFSGEENECIENKIK
ECVDHHLFIVSFAEDMSQFFGPLLAVNYSYHLFGLSLLLVECMQGEEGAYTRYGPLTLITIAQLMLLSI
TFEIVASESEKLINEVYYVPWEYMSVSNQKSMCILLGRVQRPIHVTAMGMADVGVQTMGQILKTTLS
YYAFLRTLNN

>CpunOR45

MTIQGKKQWEFTSFPETFLITSFSMSIGMIYPNPATDKWRLLAIPFALLSVSPVSVFISIEMLKLWNNGE
ILELMRHTAMFLPFFAGFMKMCLMYYRRVEAKKIIDIINHDYAYYNNLPDDYKAMVAASIKSSHIYIN
IWAACVMVIFAVFVGTASLLNIVSHLFMQEPKRYMIYDINMPGRDPEERFNSPYFEIMYFYTIYVALM
YVLSFTGYDGLMIASVNHACLRQNIFTRQVQEAMGLRGERLRRKMAAAVKDQVEALRLIDYVQTT
FNIYLGYMYVIVLVEMVICIYLTNEDYNFDFQYTCLSFGTILHIYVPCMIADKLKCTCEDTAVRLYCC
GWENMYDLRVRRCLPFMIARAQCPVTLKAFGLLTYEMDLFVGTMKSAYSMYTLLKTQQ

>CpunOR46

MTVKMQKGQPLLLTHIRILRLSLMSCGAWPNEVLEGSSRRRFSLAFIIIIISCINTCGELNYLIKNYNILP
FFDLGDLYMTFFLTILTLIRAIIPTVKSYGDVVCTFFMEFHLEHYKQKGEYYEEICNKINKFSHYFTLLT
VANMTLGPIFFNLAPLYNNYRHGAFTNNREENYTLQFSVYFLYPWYDQEDHFITTSLINLYLSYFCAL
LVCCLDLLMSLMAFQIIGHIKTLIYDLRHVPRPKRHISMDISFRETNTKANIYADIYDEQENIVIRAKIID
FVNHHRQIVNFAGSMSELFGPMLAFTYLYQLISCALLLLECSQGEPAALARYGPLTVIIFYQLAQISFTF
EFIGSESDKLKDETYDIPWECMSVKNQRLVWMLLNRIQIPIRVTALGMVEVGVQTMVAILKTTFSYFA
LLKSINE

>OfurOR2

MMTKVKAQGLVSDLMPNIKLMQAAGHFLFNYHSDNSGMTTLLRKVYSSVHAFLIVINYLCMAAN
MAQYSEEVNELTANTITVLFFAHSVIKMLFFAVNSKSFYRTLAVWNQSNSHPLFTESDARYHQLALTK
MRRLLYFICGVTVLAVMSWITITFFGESVRMIANKETNETLTEPAPRLPLKTWYPFDAMSGTMYVVA
FVYQVYWLFFSMAIANLMDVMFCSWLIFACEQLQHLKAIMKPLMELSASLDTYRPNTAELFRASST
EKSEKMPDTVDMDIRGIYSTQQDFGMTLRGAGGRLQNFGQPNPNNPNGLTQKQEMLARSAIKYWV
ERHKHVVRLVASIGDTYGTALLFHMLVSTITLTLLAYQATKINGINVYAFSTIGYLSYTLGQVFHFCIFG
NRLIEESSSVMEAAYSCQWYDGSEEAKTFVQIVCQQCQKAMSISGAKFFTVSLDLFASVLGAVVTYF
MVLVQLK

>OfurOR3

MFKIGNENDINARHPMDLRYMKFLRMLLRMIDSWPHQQLRDSKPVRFRDSRYLFIEGAGVGIGGLF
YVRSHYKVVPFLEIGQTYLTIFLSVVATQRVTIAWFKSFREVITEFVLKIHLFYFRHKSNYTENVYQRI
NRLCSVFVAFVAVEVTIGIFLFNLMPFLNNYKKGMFNQELPANKVFEHSINYSLPYVDCYTNLIGYIV
MTLINIICSYDCGMFFSSVDVCIAVIVFHIWGHLKILDHRLRTFPTPVQMRGHQPGEPGNDLMYTKEE
NMKAAAMLRDIIEYHGMIMRFMTKTSEAFGPTLCLYYVFHQVSGCILLLECSSLDPESLGRYAGLTV
TLFQLLIQVSVIVELLGTQSETLKDAVYSMPWECMDTSNRRTVLFLLYNVQEPIRLKPMGIVSVGVQT
MATIIKTSFSYFMLLRTFT

>OfurOR4

MPAVHQNPSTLSYIITVKNALGPSGIWPSNIFEDKLQPLFFRIHRETLPYHTMLIVFGGLYYLSDNFRIM
SFLDMGHIILSTFLAMVTAMRSVVPNLKIYVALLTKLGREIHLMHFAHKGPYYEEINKTVDKASHIYT
KFIVVFMYMTMMMFNITPIYNISKNILSSKTENSTQEYALYYSFPGINPMNYYPTTTVYNFYLSYNCG
IMMCGLDLVLFLMIFQLIGHVYILRHNLENFPSPKNKVVLNIGDLPRYKNKENCIVEMFDAKENEEVR
VRLAECIEHHKIIIRFTDEISVVFGPILAFNYMFHMVGCCLLLLECSAGNQIIRYGPLTTVVFGQLIQISV
MFEMLGAETEKLKDSAYFVPWECMNISNRRTAQIMLHKMQDKISIKALGLAAVGVNTMMGILKTTF

SYYAFLQTMND

>OfurOR6

MQQESPLQLGYIKTIRFFLRPSGSWPSDVFEGYLPLPIRIHRATLPFHTTIIVMGGLYYITDNFHRLSFL
DMGHMIITTFLAMVTALRSILPNLQTYNSLLCKFIQEFHLMHHAYKGDYFEEVNKTVDKISSYCTKFS
TIIMYLAILFFNITPTYNNIRHTLISKTENYSMEYSVYFSFPGFNPLDHFASTTVYNIYLSYNCSTLFCGF
DLLLFLMIFQIIGHVYILRHNLENFQSPKNKITLNLRGDALITNNTCTYEVFDAQENEEVRLQLAECIE
HHKIIIGFTDDVSGLYGPLLAFNYFFHMIACCLLLLECTEGSYDAVLRYGPLTILVFGQLIQMSVMFEL
LGSETEKLKDSAYCLPWEAMNTSNQRTAFIMLHKMQYKISLKALGLAAVGVNTMVGILKTTFSYYA
FLQTMGDR

>OfurOR7

MVIIRSAFRVVGAWPSKFIGDVQTTSDVVVKYIQLVLNVVCQVAGILYLRENMDKLSFFELGHSYITV
LMSVVSMSRIITYCTEAYQEIFSLYVRKIHLFNVRNDSEYAMEMHTKINKLCYFLTFFIHAFMTLGILM
FNLIPMYSNYISGKFNRETGAFSGVSNATMEHAVYFLWPFNDTTDPIGYAIIVVFNWYISLVCSINYCT
FDLFVYHLVFHIWGHLKILIHNLETFPRPIGAINEEQNDYTEEESKQIYERLKKLVQHHNLIIDFIARISD
TFGLSLFVYLCYHQVCGCILLLECSTLELSALIRYGPLTAITFQLLIQVSLVFELLGSITESLMNAVYELP
WEYMEVRHRRTVHIMLRQSQVSLNTRALNMVDIGSRTMIAIIKTSLSYFVMLRTFATDD

>OfurOR8

MSNILKYFNTRNSYELSFFREGDPLALNYFKIIRIFMVAPGAWPADVFGEKLSLLVRVHRALMPYHTS
VIVIGELYYLYIHKEELDFLNMGHMIIFSFLGVLIAIRSILPQLRKYHLLLTKFVKVMHLMHFKNKGPY
YKQINETVDKISYYYTIFVALLVTTAMINFNIVPLFNNVTNVLIYKTENFTLEFALYYKYPGFDPLDYF
TSTTIYNVYLSYNCSIMVSGIDLILFLIIFQIIGHVYILRYNLENFPSPKIKVVFKLKEILKHKGNEDISSE
MFDAEENREVRLKLQECIEHHKLIIGFTDELSELFGPILAINYFFHLVCCSLLLLECSEGGAWIRYGPLT
VVIYGQLIQMSVIFEMLGSETEKLPDSAYFLPWECMDTSNRRTACIMLHKMQYKISLKALGLAAVGV
STMTGILKTTFSYYAFLQTMGE

>OfurOR9

MLYYSQLTVRRKVIKNIVDGYLACDAQTLKSDRFRQNLLKGLRIVKKRGLIFWMVIIGNGTIYIMKPI
VTPGRHIMEDLFIIYGLEPMFESPNYEIGFLLTAGGVICTCYLPANITALLTVLIGYTEATMLALSEELVH
LWSDAQEHYNKYLLETQVDNAGALVTPNDDIKNQIINKYIKQKLEEIVKIHTTNINLIQQIEHVFRGAI
AVEFLLLITGLISELLGGLENTYIEMPFALMQVAMDCLTGQRMMDACDKFENSVYDCKWENFNVAN
MRTVLLMLQNAQKTMVLSAGGMTQLSFTCLMTVIRSIYSAYTTLRSMMA

>OfurOR10

MFRLKEKDVIASNTQSQVFKPNIFFWKIFGWWPEVTSTIYYRCYYISFLSLTSVVYLFLFTLSLLYSPIE
LEIIIAQAMFYFTEISGLSKIFMIVIRREDIQKAFKMLDSEEFQGDDVIPREIINKNKVYYLKYYRACAT
FYYIGSFFLLFLPIIEYVAGHADLELPLCQYYFLSEHVRDKYFNVIFIYQFFGLFVLISGNVNIDTFICGL
LLMAIAQFRMLNWKMSNLKMNPLDLENESDDEETIMMRKLNKCLKHYDLILEYCDHIQDVLSAAIF
AQYGTAAATMCLSMCTVLMPMTSEDWLFMGCYIGAMTLEIFLPGLLGAELMNESQKLVAAAYSAD
WIPRSESFKRSLRLLVERANRPIVITGLKMFTLSLETFTSIIKLAYSFFTLLKNVQETEIA

>OfurOR11

MEFIKRNYREFRSRMQDYSYDSLLSIVNFVPSLVGFSILGNKISAPFWILHLSLLFYIYGVGCTVYQVK
YAGDARDFIKCYVNVTLLLLIANNSHWFLLKRPLLKSILQEISQSDALATANEAFRGKHKRAVQRIKR
ILFMFYGFNLTNAMFVYLPNRMDVKNSYSMTPCYGMEPLTASPNKEICSALLLIQEISIMMVVLNYQ
ALLVVLIAYTALLYRLLSEEIMTLNNYDRQTYFNNPIAKTMLHELIKRHVILLSIIDQLKSLYSGSIGINF

GSNAVCMSLFFYLPFQEWLQFMPVVVYCFLVFFLYCFLCQRLTNAAEYFEQCVYSCGWENFDVKEK
KAIYFMLRQAQRPVEILAADIIPVNISTFATTLQAMFKFVTVVKV

>OfurOR12

MIIKFLQSFEDPDKPFYGPNFWILTKTGLILPENKIAKALYILMHEIVAFFVFTQYMELYIIRSNLDLVLT
NLRISMLSVVCVVKANTFVFWQEKWNKIIDYLTEADSVERYSNDPERKKIIDKYTNYSRRVTYTYW
VLVFITLATTIGSPFIHFVSASYRESLRNGTELFPHILSSWMPIDKNHSPGIFITIVWHFTVTSYGALIMSS
YDTSIMVIMVFFGGKLDVLRERCKQMLGTGEVELSDDEVAARVRELHNTHVLIMKHLRLFDSVLSP
VMFVYVVMCSLMLCASAYQLTSATNAAQKLLMAEYLIFGIAQLFIFCWHSNDVLVKSENVMLGPYE
SRWWDANVRQRKSILLLAGQLRISKVFTAGPFTNLTLSTFITILKGAYSYFTLLRE

>OfurOR13

MLNVLKRLENPKRPLLGPNVKALKFWGLLLPENIYMKYFYLLMHVLVTIFTATEYVDIWFIKYDLNL
ILNNLKITMLATMSVLKITTFLYWQQHWKDIIEYVTRADLAQRTTDDVEKNVLITKSTRYCRKITLFY
WSLMYTTVVIVIFQPIFKYFLSRNYRENVKNGTDSYLQVVSSWVPWDKSTIPGYLIASAFQSYAAIYG
GGWITSFDSNAMVIMVFFKAELELLKIDCSNMFGTETKPVSDEVALKRLKDCHRRHVELLKYSRLFD
ACLSPIMLLYMFVCSVMLCVTAYQITSETSAMQQFLTTEYLVFGIAQLFIYCWHSNDVYYASLQLSQG
PYESLWWYRDVSHRKNLYILTAQFSRVVVFSAGPFTKLTVATFLSIIKGAYSYYTLLSKSQTK

>OfurOR14

MTILNSIWRKLTNTKALEKSSGCLETQFFETVYRVSYLTGISMADEDIPYLIYSSVVKLLIFLLIVGEFW
HLATEVTSFDEMADMVNITVIQYIAIFRYRSMLYHKDVYKKLAISMESQYFDISTKERRDVVDYWVK
RNANNVKLLLVLGNCTLIAWFLYPLVDDLEYNVFIGIRLPFPYYSPVCYAFVYLLLLIVFSYISHFVMA
NDLIMQAHLLHMVCQFDVLCNCFENLMEDCAKGFKGIDRESLLANANYREVFKARLGDMITQHRYI
LDHAMELRHTLSGPMLGQLAASGTLICFIGYQATTSGAYNITKCLMSLFYLCYNLLVFYIICRWCEEIS
VQSQRVGEAVYCSNWECGASNIPGVKVSLLMVITRANKPLTLTAGGVYDLSLMTFSSILKTSYSALTL
LLRLKSTE

>OfurOR15

MALMVYQKVLEEKITENDEEIFFKPFEETFTILIFSMVFGMIYPSDRMKNWQILGFFGFIIIMIPALSAV
YYDMYLAYLDRDMDTIFRHLIVIGPFNALYLKWVYMYYYRQQSKDAIEEMNRYFANLNFKPITHKR
IAKKWLIRSFFLEKSWAYCLIVGSFSFPVMAICKTTYSTLFDEEPRRYFIHELRSPQGPGMNYEFPFFEV
LFVNTCIASCMYFLNFSGYDGFFVQLILHTCMRMAICGEAVKDSFKIDDKAMRRSALHKVIDEHIAIC
HFMDNINCIFVQWMSLFTVALTIHVCICVFHLSEGTYQDMEFMFAFTAASIYLCMITSCGGLVEEESE
NLADAFYQSGWERVLDTHCNYLLVFMIARAQKTFRVRTLFHVHVNHELLIAIVKMAYTLLTFLKQT

>OfurOR16

MSLWSTIRKFGLGYCDLPTMLWNVSFMLRALTLNIDSRYKKRIPLIFYIIFALVAASYFYIYLISMAWF
VFWHSRETGDLVAAMVVASLGISSEIGTAKLIYMFLYRNKVRELVDMYLDCDALVKPGSRFANNLTK
TLRNVKKRAMIFWIVIMGNGVVYVLKPLLISGRHIMEDLFTPYGFDPVYESPNYEIVFLLMTAGVLFT
CYLPANITAFLIIITGYTEGQMLALSKEMLNLWSDAQQFYLDHRTFDLDTTRPVVTLDSEQITKKKIV
NEYVKKRLHEMIKIHTTNINLLNHVERVYRGAIAIEFGILVLGLIFELLGGLENTYLEVPFALMQVAM
DCLTGQRVMDASKAFEDAVYDCKWENFDVANMKTILLMLQNSQKTMRLSAGGVTTLSFSSLMMVF
RSVYSAYTTLRTTMNK

>OfurOR17

MEKSPNFEFGYALVAASVWFLCYVPANVTSVLIVFAGYIEAQMLALTQELLHIWADAEQHYANINLN
TLKRGTFVDAKYKKRVINEFITMRLHDIIRKHATNVHILHLLEEVFKGAIAFEFLFLIMGLIAELLGGL

QNTILEMPYAFVQVAMDCWTGQRVMDASAEFAAAVYACNWEMFDVPNMKIVLLMLASAQKTMKL
SAGGVTMLSFECLMSVVKNIYSAYTTLRSAFTINTHAH

>OfurOR18

MKFPWRRGGMRPKEPLSTLDSLPINNYTHFLEIPLKIVGCWDWYDHPKSEKEIIINNVYFCMVLFVLI
NVPATLYIHLHTEWVDVMTSLDKLADCLPFVVSIIIVVYFGLYRKELYELTKFMQRKFHYRSANGLTN
MTMLNSYKTARNFGYFYTACTMFSVSMYMIPEIVNRLKRQPLQSYMYMDVTRTPFFEFTLLRQCVA
QAFVGLAMGQFGVFFASNAILLCGQLDLLCCSLRNARYTALLRCGVSHRSVAAAHSDIQGDELYNYI
YNIAEMRQSIYHYDQRMSYEIMNKRSSFDIYSSEFDAATCEALRDCARACDVINTFKAKFESFVSPLL
ALRVVQVTMYLCMLLYAATLKLDMVTVEYLVAVALDIFVYCFYGNQIIIQADRVSTAAYQSAWPTMG
VRPRRLLLNILLANKRPVVVRAGNFLSMDLHTFVVIIKTSFSYYTLLVNVNEK

>OfurOR19

MEGLEAYPEEFVNSLKLSLEYYKRVNITFFGSKTSFWDKYRHFFVFGIPFAFFYYTVTMYMVKVVAE
GLDPFAKPDMIALWLISTQVIFKYILFTKNKEGVRLVIEHLGAVWRMTDLTKEQILIKNSSLKFLKYGL
YIYNKSCMTTAWQYFLYPFISMLFKHIFWGNEIEMVLPFPCEYPFAVDNWPVYLAVYALQIIGALQMV
HLYLAPNFLLTNLSIHISTQFRLLQDDLINIKPTNNKKTKYQYDMEITKYYEGKEYTIEDFVRRHQDIIL
LTRQLNDAFNKMVFVNLVISTVVVCFFAVAVKTTIDPAYKLTNGAALVAYMANLLIVCYCSEMLSISST
GIALSAAKNMWYDGDLRYQKIICIIIMRSQKPCTLLALNYYSISMKTFNKALKTTYSYFSLASHIYDG
RKERKYTTEY

>OfurOR20

MWNIRFLKEKRFTILNVFNFLEDPRYPLVGPHLRLLGLTGLWHPNLNSKTRFKQYLFFITIAFFFSQYV
KCAVKLEPSSLMLILQYAPFHLGIIKSCFFQKDHKKWESLIDYISGVERKEIANGNKDSNDIISEYISRS
RKVTYFFWALAFFSNFTIFTEPYQKNQINVNGTSVYLKIFDGYTPFSEVPPGYYASMLTQTVLGHIVSA
YVVGWDTLVCTIMIFFAGQLKISRLNCANVIDINNAERSHENIVNCHSFHTILVKNQKLFNSLISPAMF
VYLIVISVNLGVCIIGIVQLQDDLTTLISSCVFVMACLIQLLLFYWHSNEVTEESTLVSYGSFECDWVE
LDQRFKKEVALLGMATRTRLVFKAGPFNEMSLTTFIAILRLSCSFYTLLSKTM

>OfurOR21

PRLARAHALYCRFALAATSVYLAQECVYAYQVRNDMDKLARVMFLLLCHVTSITKQLVFYMSADKI
DEMINALDDPLYNQPAAWQRALLAATARSAGRLLRAYSGTAVVTCTLWIIFPILYYSQGLPVEFPFWT
NLDHSKPTFFVILLMYSYYVTTLVGIANTTMDAFMGTVLYQCKTQLRILRMNLENLIERATTVVKEN
SDEIFDKVLDRLFLECLEHYRQISETNRRLQDIFGTSILVQFGIGGWILCMAAYKMIGLNILSIEFASMT
LFITCILTELFLYCYYGNEVTVESDRMVEAVYAMEWLHAPLRFKRSLVLVMERAKRPLRPAAGHLIPL
SLDTFVTILKSSYSFYAVLRQTK

>OfurOR22

MLRNFLLSLENDNHPLLSPTLWGLQKWGLWQPNKVLNSNISNFIHFAATLFVISQYVELWLIRDNLNY
ALRNLSVTMLSTVCVVKAFTFVTWQDQWKDVIDNVSLLEKRQLSKKDKITDKIISEYTNYARRVTNF
YWTLVAATVFTVILAPLVCFLSSPDTRERIRDGYEPYPEIMSSWVPFDRSRGLGYWVTVLEHILICFYG
GGIVATYDSNAVVLITFFAGQMKLLSVNCSRLFDDEKEMTYEDDMEKIRACHYHHLMLIKYSKILNS
LLSPVMFLYVIICSLMICASGIQLTTEGTTTMQRIWIAEYLMALIAQLFLYCWHSNEALVMSNKVDDG
VYASAWWSRSIQVRRCVLLLAGQLRKSVVFTAGPFTKLNVPTFIAILKGSYSYYTLLNNKDD

>OfurOR23

MAEQPIDKSLRKIRFIFRYAGMNLEERPRTWCQSFIYVVNFLWIATDIIGEINWIFEGVSKGTSFVELTH
VAPCLSLGTMSEFKTAFVVAHEKSLFRLIGNVREMERKRLVGPIAHKIVKEESKFLYNLVFAMKMVN

WVLVVVFDFGPLVWIVVKYFIYGELELLLPIIDIYPFDCYDLRIWPFAYIHQIWTAWVVVTEILGVDCL
FYICCTHVMIQFKILNHEVTNVIAESRSAKRIEVTQLREKFNELVKWHQDIINSAGLLEDIYSKSTLVN
FLTSSLIICLTGFNMTALDNVRMTVAFAFFVVAMLQIYFLCFFGNMLMDASTDVSTAVYNSRWYLSD
AAFGKSALIMQIRAQKPCIVTAAGFAEVNLRAFMKIISTSWSYFALLRTVYQDI

>OfurOR24

MFSLSFVITHDLTLIKLCIFYFKNDQIQDIVRTLEIDLYNFYQNNRKNRATVRITRIMSASFVFFGWITIG
NTNVYGTIMDFRWKAEVAKLNASSIKPPRTLPQPIFIPWEYQTDQSYISTFVLETVGLLWTGHIVMAI
DTFIGSVILHMSSQFTILREAIVTAYDRTITKMYINAKHQYDSLEAVSSNDEENSSIDQSTQDGMEALV
LARFSKKEVELALQETLKNCFQQHQVLIRCVEKFAETYSYGFMTQLLSSMAAICVVMVQVSQDASS
FKSIRLVTSLAFFIAMIIQLAIQCFTGNELTLEAARIADAVMQCKWERMPPSLRRMLIMVMMRAQRPL
RLSAAGFAYMNNDCFLAIMKAAYSYYAVLSQKTK

>OfurOR25

MAKEAFENSLRLTKLFLLLSGIRITRRKWRKSVENFFDYYLYYISLSWLYTDVCGELNWLIEGILTGKS
FIDLSLTAPCITISMLATSKSIFLYWNRDVVAKIVDKLRDIHPEDKEFDEYKQLGLYQVESNEPDVEKEI
VEESRKFLSFVVHLLFYICAVVICAFPLMPVTSMAYDYYTTGSTECKYPYLVKYFFDPYTMKMWIAV
YFHHVVSTAIVGANVFGSDSLFYVVCIYIQMHFQTLCHRCECAVVSSREGTRRNVANAVKRHQELID
LVNQVELLYSKSTLFNIVTSSVLICLCSFIITVLDEIIVVVTFATFLVMNLSQISLLCYFGDILMRSSTEVS
SAVYNSLWYETDQSVKKSMLVILMRAQKPCKLTAWNFADLNLTAFTTILSRSWSYFALLKTMYK

>OfurOR26

MEEESLFDKSLKKITFAFRLTGLNIENDKRNLKQNCVYLFNFLWLNTDIVGALQWVLYGIASGKNFT
ELTYVAPCLALSILGDIKGVFMILNEKKVHILMDNLRSLELKAKEFENSEREDMIEPEIKFLNIITSVLN
VLNCLMIVVFDASPLILIAVKYFTTGQLELMLPFLDVYPFDSFDLRYWPFAYIHQIWSECIVLLEICATD
YFFFTCCTHIKIQFKLLQHQFQEIIPSRSVSAVDSIDQAAIRTKFQELIKWHQEIIRSANMLEGVYSKSTL
LNFCTSSLVICLTGFNVTTIDDKAFVMTFIIFLFMSLLQVFFLCFFGDILMSSSMDVSNAVYNSRWYLT
DVMMGRNVLLVQTRAQDPCKLTAAGFADVNLRAYMKILSTAWSYFALLQTIYC

>OfurOR27

MSDITLSEAKREIAESLTLNTFCMRRIGLSFEEPKNASSYFAQKFMLVLSVMSICYHVFSEIVYIGLTLS
NSPNVEDVVPLFHTFGYGALSIAKVFALWYKKDVFKQLISELAGIWPMSPLDDDATVIKAKSLTALRI
AHQWYFVINVLGVWFYNLTPIIVYAYRVWQGQDVEMGYVWVSWYPFDKHQPVAHVAVYIFEIFAG
QTCVWIMVGTDLLFSGMASHIGLLLRLLQRRLETLATMEQTEEDNYRDILASIKLHQRLIRYCNDLEV
AFSFSNLVNIILSSVNICCVVFTIVLLEPFLDISNKLFLGSALIQIGMLCWYADDILHANADVAAAAYTS
GWYRTSARCRRALLFLIQRAQKPIAFTAMGFTDISLVTYSSILTRSYSYFALLYTMYNDK

>OfurOR28

MIITYHQDLSTVVEFNTIPYNSMEDHTIRMEKEFGPFHDTYRLNMYSMSYGMIYPNPATNKWRLLAI
PILCFTTIPMTVLVFLDIRRYWIDGEILEVIRHVGLIGPFITGILKMCLLYYKEEPTSQILALINRDYASFN
QLPESYKPLVRGYVKNTRFYQNIWIACVLMILSTFVLTASVMNICETLFSSEPKRHMIYDVRLPIDRPG
AQFETPYFEILYIYMLYIAAVYTINFTGYDGFMIACVYHACLRIELFCKYVHDAMGYEGDELRRRLGE
AVNHHCETFKLIEKCESSFNFYLGLVYVVVTTELCLCLYLVMEGFEFDYKFSSFSIGTILHIYVPCLVAE
KLKCVCENASDLIYCCGWENNYDLSMRKFIPYMMARAQKPVAMKALGLITFEMSLFASTMKTAYS
MYTIIKTQ

>OfurOR29

MKWKKNKYGSRIVSTKKDNEFLQKNNLVTMITHRIKNIGLTFCVSDGIKIHWLAIFAIISFVLTQGLQII

GLFNAKDDIDKVFEYFSVMSFCGMGILKLLSLCRNHKQWKILLDNIKQLEKTQCQNETSNVEYESDG
ENDTFTFPSYIESYTKKFKIVSTVLSRMYGFTAIVYILSPFAEFTLLIMTGNEDYEKPHVLPGWAPFDSR
SFVGYLTNVAVEIISVTYCVLVHITFDLTSIGVMIFICGQFSLIRDYSSNIGGSGASCTLSKRREDRAHHR
IITCHKIHCLLMNTCDELGKQLQNILGVYFSVATLTLCSVAVRLNSELSRMELASLLQFMCATLTQLYL
FCHFGHNVLHQSSIGMGDGPFGAAYWCLSPRIRQELVILGMGMMMPRYFKAGPFISVDLPSFVQVLR
TAYSYYAVIRK

>OfurOR30

MATFNSEDLFLSRAKFVMKFLGVWMPPVDETLPRKLFKIFMLTLQYLFLIFQTIYITQIWGDLEAVSQ
PSYLLFTQACLCLKITIFHVNIDNLRELLKQMGSEVFLPQSRVHEEILKTQAARIKRFLLAFMISSQIVC
TIWVVHPMLQKTGPRKFPFDMWMPVSPDDSPQYEIGYAFQLLTICMSAYMYFGVDSVALSLVIFGCA
QVEIIKDKILSISPVQHRLKESERKIIFEKNHKILVECVIQHQAVVTFTQLVEDTYHWYLLFQLTGDVG
VTCMSALNILAQEVRSLQFVTILIYVIVMLSQLFICCWSGHELTATSEGLHTVLYQCIWYEQDLKFKR
DLRFVMMRMSRPMVLRAGHYIGMSRQTFVAVLRMSYSYFAVLNQANRVEQQ

>OfurOR31

MKFFVVNMYTDLKISLTILLYTGFWTRQKEVTNILSYCYPFLTFMFMAGISIIAQFVDLLHVWGDVSL
MTSSSFLLFTNVSFGLKMFNILWKREEVRAIIDDCDQVLRAVDTSWGYEIVKSGIRKSFLLFSIYTFLA
NISVFGWAISPEKGELPTRAWYPYNTTSSPGYELTYLHQVSAVLLGASVNASLDTVVISLMAQCTCRL
RLLAAALRMLGGDMLVTNMFEAEQERAIREELQRCVQQHRSVLQVAGLLQQYFSTSILAQFSVSLVI
ICVTAYQLAFVSSNVLTILGMTTYFMCMLMQVFLYCYQAHELSTVSSQVGDAVYESLWYEMSAPLR
KDLLVLMVRSQRVIKITAGGFTTLSLNTFMAIIKTSYTLFTVLQRED

>OfurOR32

MKIIPKGIIEKCTRTVGDRSEIDEVMRAILITQRAVGNQILDPYWSWMKSLPHQLVLGAFMFYVLIGT
WEFVGGTDDVKLIAEGSFTYIVAALIPSRYYFFLMARKDFQKLYIAFKTTVCKFITDDSEEKMEQLLK
KTRSLVKFMLFSSNFPMAIYFLAAMWHYVQGEKRTISKTTSILMPMRSPYHEIGLFAHSFFIFEAGFLI
LVPDMWFVVIMLFFCSACDSAAKFLIVEERRNESKLQYATRLNDSLRKFYVAHVKLIDFLDVLNSVF
KWLALLPLISVGVGICIILLLITQGVDWAFLSNILPVMGELFVYNWFGEQIINKAEKWSLALLNFDWI
NLSAKDKKCYYIMVSYMQKKFRMKTAIGSEFSLLTMSTCVKGGYQAFTVLQSATHKRE

>OfurOR33

MEDTIKVFHRVLSFAGILIYAEGNWDSKLWLAFQIFNFIIGSLSFIFTTGFVVVNCSDLLIFIQGACIWTT
GVIMTISLGVCLIFRKKFRIFLGEMVFKDEILEMPLIQFVLKLESGKKLIELKQMVNDSQEKLFRLTKV
LLKCYVTSVWLVATMYLCSPIYEMLSRGDKSLRLLAFDMWFPWSLENFKVYVISFVFHAYAGYLCC
VAYPGLQLTIILLIGQVIRQLRILTFVMENLDELVMEIIKEKGDRWQMCCTAVLSQCVDHYIKLKRFSN
RLNVICQPFYLTLILVAIMLVCMCSVKIAISEKLSPDTIKYYVHEFCFIMVVLMFCLLGQQVDNECANL
ERAVTENWYIFDKKHKIHVRIFKMALSQRMRVFIFGSIPLSLPTFTWFIKTGMSFFTLVMSVLED

>OfurOR34

MTSTQANGNRVYSRNDYDETYKLIITNILAKVGIRMTRKDSKYARLGWNVFFCFGFGNMVVTLFLD
LVTFQDVVRSGVGEDGYIVFMMLPCMGYMALAMLKTYKMVYKRDVFENLISELREMWPEGLVTE
EEHTIISRALNELNIIVKGYYWCNLGLGVSFMAPSFVVAIRRIFGADIPPSLPYFYWLPYDQSQPVAYEF
TLVMNTYHTLLTLWYMLAGDLLFCVFLSHITTQFDLMSVRITRLFQVPVDQQLIPEYPLGQQIKDFPE
NGHLPRLSNNEINSKQENELQKIIVRHNALIRLSGDVEDLFSFAIFINFFNSSIIICFCGFCCVMIEKWNS
LMYKTFLATSLSQTWLLCWHGQKLLESSERVADALYNSGWYTAANGIKKSILIMIHRSQKNVYVTTY
GFSIICLASYTAIIKTAWSYFTLLLNTYNP

>OfurOR35

MGIVMENVKKRLTILQPILPYGVIEPWDDLNPRLYHAIHIYWLKFYGMWYNNASPKTIVFWLQLIYT
ATVLWLVCFLPGIGEVVYLLKRRGNIGDVAEGLYLFLSEMYTYFKVAVFWLNKDKVINLLRYLSCEE
FKPVEMEHREIIRKSIKAARFVMTYYSTMCVGAVSVGIIMPLTENFDILPTNVEYPYFDVYKSPVYQT
LYIHHVYYKPATCIIDGVMDTILAAFVASAIGQIEILAFNLRNFDVVAERRRKRAVAENKPSAAWTQER
HIRAVLKDCILHHNSIIKYVSMIEGTFSLASALQFMLSVMVLCLVGIQFLSIENPSSHPMQIMWMAIYL
TCMLIEVFILCWFGDELIWKSTALRQAAFDGPWLETNHKTMVFIVIFLERCKRPLRVTAGKIFTLSLDT
YTILINWSYKAFAVVSNMKK

>OfurOR36

MKDYEILKKHCKRIYLIGSGDFWYEDGTIGDDKSWYYKVYSWSLLSVYGFMTILEIMAAMIGDYPE
DEKRDSVTFAVSHTIVMLKIFSVHSNKQMIKAMNKNMVYICEAHEEPTLMAEKYKIVKINVLAYFSI
VYGSGLFYVFEGIRKIFAGSHFVTIVTYPPSYEDDSLYSVAFRVSTTVILFMLLLTMIVSVDSLTMTYLI
MFKYKFITLRNYFERLTEDFYKMNDVNPREAADKLTNGLVEGIIMHKELLRMAKDIDQAFGTVIALQ
LCQSSGSAVSLLLQIALSDQLTFVASMKIIFFVAALFFLLGLFLCNAGEITYQASLLPDAVFYCGWHAC
ARQPPRRSARRIVLLACAQAQRPIVMKAFKMIQLSYSTFLQVLRGTYSVFALFYAQNK

>OfurOR37

MIVKNVTTSVSMSLTALRLVGFWMPEHFGGNKRILYDCYGFFSFMFLLGTYLIIQAVDMCMIWGDLP
LMTGVAFILFTNLAQATKIFFMVWRRKQVLTIIRGADEVLRAVESDEAKAIVKSCSRETTFLHIVYNCL
TLVTMVGWGTSAEKNQLPLRAWYPYNTSKSPAYELTYMHQIGALCVAAFLNVCKDSLVTSLIAQCR
CRLRLLGLSLRSLCKDLHATGKQYTAEQEAIVRARLCACVREHQAALVAAQQIQDVFSEQTFAQFNV
SLVIICVTAFQLVSQTGNLVRLMSMGTYLVNMMYQVFLYCYQGNQLSEESAMIAGSAYECPWYLMSI
SLRRSLLIVMIRTRRVSKITAGGFTTLSLASFMAIIKASYSLFTLLQQVEGKK

>OfurOR38

MENPGTSDETNKISRFFFQVCRLIYLLGLPNFWSKDFIFSKSFITFYDSFTKIMNVTTYVFVVTEWGAF
YTQQNLNEKQHSDRLVFCISHPILASYRLILTHHGEKLKELVYLLCLDLKEKANDEKIEKDTIRKAIIYA
TALVGLCIISIVSYGIDALANSISSDATFTTVVTAWPDVGDRSTLAGMARIMFYVIWWIFMSRVFGAFII
MISIIVALEHQYKNLGNYFRNLSKVFEQNLSPIEKEKKYEESLKVGIQLHAKTLKCIELAQASFGPIFG
AQIILNTYVIVLLLFQMVSSERTLGNVLATIFTGLAMLLSTGFLMWSGGDITIEAASLSTDMYWSGWQ
NCQASTTGTRKLLALAMLQAQRPVMIKGLGVITISYPAYLSIVKSAYSVFSVLY

>OfurOR39

MDELRLKVMVWSGIYKLHTKNRFLGICHDVYRVLMILYMSIYTVQHFVFIYMNVTRGDAINWQVAV
FSIGMLNMVVKGITIYMHPESIDEIHDLIKDPMFAATCKEDEDIIKKNEYHIGLFIKITYVTLTVCLFFW
VASIIVTRLVDDTAMPPSYFPFATNPWPQYIIATFVETVGSVLWFGYGHFSIDCSVACYYGRATAQLRII
RYHLEHFFDNGGAQGRFQYKDVVDRSLDEKFVYYVQCYQYVNRMIDNVSDAFNWGIAFHLCIVTT
GMGMCVFIISTRDIFSLDMLFTVTIFVLLLLQNFMYCYCGDLVKSESDQVCTSMYFSDWTAVSPRFRR
KMLIAMTRWARPIEPRVTIVPISLTTFASILRFSYTLYTMMKTRTM

>OfurOR40

MHCLLQMLNENIVRVIFIKVNSTYYPLSVKIPKTMNNYDILKNHCKKIFFSGSGDVWYEEGTIGDDKS
WYYRLYSWSLFSMYMFMTILEIMAAMFGDFPEEEMRDSVSLAVSHAIVMLKIYFLYSNKNLLKTMN
QNMVRICEAHEEPSLMAHKHRIVKITLRVYFGIVYGSTFCYVIEGIRKLFDGSHFVTVVTYYPSYEDD
SFWANGFRIFNTIVLLMLMMTMIVSVDSLTITYLIMFKYKFITLRHYFKTCSQDFFKLNDVDPRLAAD
KLTDGIVEGIVMHNELLRMVKDFDQAFGTVMALQLFLSSGTAVSLLLQIALADQLTLVASLKMIFFVT
ALVFILGLLLCNAGEITYQASLLPNALFYCGWHACVWQPPRRSVRRLVLLACAQAQQPLVIKAFKMF
ELSYGTYLQVLKGTYSLFTLFYGQNQ

>OfurOR41

MNLLNFFKKYTEDDLINIQEHHFESFNKTYQWIAFTLTLGIMFPNPATDRFRIISINVLLVCVFPLAMM
VLIDMYKCWMVKDIFNIIRHSTIVGPFLGAFFKMFLMYYKRAQAKEILDEINRDHASFNFLPRKQQDI
AFLNVKKGVFNVERLWAPIVSIAIMTFPGMAVVMTLYSYAFSDNPKRYMIHEVKPPNSRDPEDMLKS
PYFEILFVYETGSAIICVLNYTAYDGLFGIATNHACLKMSLCCMKLKEAFRCDSTEDMYKGILTFIEEQ
KKMFRFVDLIQDTFNIWLGTILTSTMIQIGSLLFHISAGYGFDLRYTLFSFTSVVHIFLPCKNAATLKD
MSTEMSTMIYSSGWERSRERRILRMIPFMVARAQVPNYITAFNLFIFDMELFVFILRTSYSMYTLIRS

>OfurOR42

NRLSNRLYRIYQYSLFIGSFIFLISTGIGTYMSKDDVIRLLSNVDKVTITYNYFFKIMIFLIKHEQIKSIISS
ILHSGDQIDVNRKYLMKIHVVMVTLLVTSITGAFQFLAQIKGELIMDAWFPFEPKKNKLTFLAATLIISI
LFVLPFMFRAIAIQGIVCSVVMYLCDQLVELQRRLKALKYSVESETYLREEFKDILKKHIRLMEYSKSI
KSAFNEFFLVQNLAITAELCLNALMMSLIGLEQKNHLVSFMAFLMMALFNAFIFCHLGNNLIDESAGI
SLAAYESTWTSWPVDLQRDLLIVITVAQKSLSLTAGGIADMSMQTYAQALYNGYSIFAVLRDVVN

>OfurOR44

MNIVLVTTTNVYRTSCTKFVQLNTVAPSHSKIDSILNESSLFHVTMTHTFLHRPKTALTMLGLWLLPE
NYKVPYLIYRSFQLSIQFTFLLFNFIYMGVVWGDLEESSEGFYLLFTQATLCLKSTTFVMNRTRLIRLL
RFMESDIFATNTPKHKRILAVQAVKMWQVYMFFMTCATCNVLEWAVVPLLESRGPRVFPFKIWMPA
DPAMCPDYVYTICYVYQAVTVYLSATTFLTIDFMTVSMITFASAQLEIIAEKIKQIPPVATSSENLKAEE
VKSRVQHNNKILNECIQQHQAVIRFVGLVENMFNVNIFFQMSGTVAIICIIGFRITIEPPNSFHFYSTLNY
LVTMVAQLYLYCWCGNELTERSQVLRDTLYTSQWYEQDRRFGSTLGIAMECMKRPIIFRAGYYIPLS
RPTFVSILRCSYSYFAVLNQANNK

>OfurOR45

MDIPTFEELFKQIKLNLWFFGIPFNGRKIELRFYFMVVVVIIMLIGEISFFVSRYAPENFMELTQLASCIC
VGALSLLKILPIAHKKQKIFELTESLDGLYNTILENPKKKAIIRRQMILVKILMKYLFIVNIALFVIYNISP
LIFMTYNYIATNEVEFILPFALGVPFSIESMATWFPVYAYSVFSSFVSVSYFVTVDALYCILTTHICSNLS
MVSEELQNVDTSNEDELKELVKNHQYILKLSENLEEIFSLPNLFNVMMSSLEICAVGFNLTMGPVSEIP
RSVVFLSSVLLQILMLSVFGEKLIEESTKVGDAAYNSKWYEVDQKTKKTILIIMTRSSKPQQLTAYKFS
VISYGSFTKIISTSWSYFTILKTVYKPPE

>OfurOR46

MLLKKWKEFYNKEDFDYSTGYVDPYQFHRTFYFVQSAFQVTDEPFKPWTYVSKTITLICGIGVLTDA
CFSFYHAIDIFDMGLITEAGTYVLMLLYKMMTLTITKVNLPSYIHFMKCMKDDFGYICTKSEKYRKV
FFETQLATWKLCVSTCLFMFCLANSLVLFAIGSLFFYLATHEPGDGTNRPLVFPFWAPGIDYTSSPAYDI
AFNFANIGVLACTYNYTFVLQTNIVWVRQIASKAEMIEMCITDLLEGIQPANNEEEKRHYANLINYR
MREIISQHQKMLLDSYASVFKKCLMFEQLVSSPVICMLAYCSAEKLDAGEIHTVMILLCVGAILILFLP
CYLCTYLRMKVTRICDACWEIRFWDAGPNIRPYLILIMQRCLRPLPLQAPGFQEVSIKTFSSKMTSAY
SLFNMLRQADLDL

>OfurOR49

MFVYHGIEVEDYSFATECFCYFVMLSVIPILYGSVLMNNRNVVLLLDKMDKDFKFICKLSFKYRDHF
LKRQLLIWQLCFTWLGFLCCVAVLYVLMTLAPLTYQSLFATQDEHMIRPLIFPMWLPKDDPYRTPNY
EVFLFLQMNFLLIFIQSFGVYVYIQFHVLLHNFILLELVILDFDVIFEGLDESVVGLSRYDPRRASIQHV
FNKRIERIVTWHDSVFKSIATLSTVQGPVIVYQVMFSSLGICLMMYQVADKLDSGTFDILFMLLTVATT
LQLWIPCYLGTLLRNKAFDVGDACWNCGWHETSLGRMIRNEILIIIMRAQHPISIKFTGLPNLSLETFS
SIMSSAYSYFNMLRQYNK

>OfurOR50

VFVTLTTALHFVFYTLNLIYTPRKIEIFATQAVYYFSSVSGLFKIGTVLTKQQEILSTFEIIDCKEFLGNCS
ETKKYLQQFRRTYFRYFRLYLTFCMCCAVVFLCGLPLVNYFFRHEDLKLPVCEYYFLTDEVRKNYIFY
WFDYQILGLIVTIVYNSTTHTFLCGLILMGITQFKILNFNIANIRLDEDIEINNQEEREQALINKLNQCL
KHYDIILKYCENVQNIADVFFFVQFSLAAITICFCMYMLILDLSDKDKVFTTCFISAMLLENYTPSFLG
SHLTAESDNLRIAAYSCNWTPRSHSFKKSLILLMERAHRPVVIVALKMVPINLETFASMVKTAYSFFTL
LSGAQE

>OfurOR53

MFPKTLAPTKINRNNFKFDNMFVVTAMAMFINRSHPSIPRNLFWVFQFLIILTLSTTTFLFLGNSVLLY
DIPAGRYAEASKNGTMAIVAFTITIKYSFLLYFQKYMKNLISVVDRDYKLAMDFEEEEKEIVIMYAKK
GAKVSWYWLLAALSTSIAFPLKAIFKMGYSYWQGDFKYIPMFDMRYPDRLDILKDIPAMFILLFVLC
LMFGCYATTMYIGFDPLVPIFLLHICGQLDILSKRILKIFSENYNEEEINEKLKNVNIKLQDLYGLIENIK
NKFTVLFEYNMKTTTFLLPLSLFQVVEDLKRSQLNMEFLSFFVATILHFYMPCYYSDNLLERSYYLRE
AIYSCGWETHPNTRARKTVLLMMTRTTAPLVLSTIFYTICLDTFAEMCRQSYAIFNIMNAACA

Supplementary Table 6.

>OfurIR21a

MTRLKCLIANIFIFVSFVLCEDVEYYPSQAALNSYSNIAKRSVNEPELEKGKVAIKWRHFNENKNETQ
EVKTKRAVDPIFHGHPKTREELWNERFLNKSSAFDQTPSLIKLIHNITLRYLNDCIPVILYDSQIKTRES
YLFQNLLKDFPVSYVHGYIDDNNKLKEPELLIPVKQCLHFIVFLTEVKSSAKVLGKQSESKVVVVARS
SQWAVQEFLASSYSRVFINLLVIGQSFKDDDDNSLEAPYILYTHKLYTDGLGASQPKVLSSWTHGKYS
RDVNLFPPKMTEGYAGHRFIVAASNQPPFVFRKIKTDLDGGNPRVIWDGIEMRLLHLLAERNNFSIEIL
EPQEPHLGSGDAVTKEIAMGRADIGVAGMYLTVDRTKSMDMSFSHSQDCAVFITLMSTALPRYRAIL
GPFHWHVWVALTFTYLIGILPLAFSDKHTLRHLLHNSGEIENMFWYVFGTFTNCFTFVGKNSWSKTT
KITTRLLIGWYWIFTIIITSCYTGSIIAFVTLPVFPETVDTIEQLIAGFYRVGTLDRGGWERWFFNSSDAK
TNKLFKKLELVPNVESGIRNTTKAFFWPYAFLGSQAELEYIVQSNFTATKSKRAMLHISNECFVPFGV
SMGFPTNSLYSAKLSGDLRRMFQSGIVDKIVDEVRWEMQRSATGKLLSAGSGSLKITSAEEKGLTLD
DTQGMFLLLAAGFLMGASALVSEWMGGITRRCRIGRKKPSSANSKEELIATPELESEIKVISDCTESRL
NFDTRCSSACSRDTLEGQVINVTEENIVVHETLDAATWDSRRSSSVDLDREVQEIFEKDLRRRRIVTG
DIEEAAEVKRELTASNGAFGDHLN

>OfurIR25a

MKPKESRFSLKLLLLFSFVRVAIFQTTQNINVLLINEENNALAEKAFEVAKEYVRRNPSLGLAVDPVIV
VGNRSDAKVFLENVCRKYNDMLSAKKTPHVVLDFTMTGVGSETIKSFTAALALPTMSSSFGQAGDL
RQWRSLDANQTRFLLQVMPPADILPESIRAIVTKQDITNAAIIFDELFVMDHKYKSLLQNIPTRHVITP
VKSFNKDEIKTQLRSLRELDIVNFFVVGSLRTIKNVLDAADENQYFGRKTAWFALSLDKGDITCGCK
DATIVYMRPTPDAKSRDRLGKIKTTYSMNGEPEITSAFYFDLSLRTFLAVKSLLDSGKWPNDMKYITC
DDYDGKNTPNRTLDLKAAFQEIKETPTYAPFYIPEDDPMNGRSYMEFNTDITAVTVKDGASIGSRVLG
SWKAGLSNPLSLTDPDNMSDYSAQLVYRVVTVEQEPFIIRDDEAPKGFKGYCIDLIEEIRQIVKFDYEI
VLSPDGNFGTMDENGNWNGIIKELIDKRADIGLTSLSVMAERENVVDFTVPYYDLVGITIMMKLPRT
ATSLFKFLTVLENDVWLSILAAYFFTSFLMWVFDKWSPYSYQNNREKYKDDEEKREFTLKECLWFC
MTSLTPQGGGEAPKNLSGRLLAATWWLFGFIIIASYTANLAAFLTVSRLDTPIESLDDLSKQYKIQYAP
LNGSAAMTYFERMAHIEVKFYEIWKEMSLNDSLSDVERAKLAVWDYPVSDKYSKMWQAMKEAGL
PNSIEEALQRVRDSKSSSEGFAWLGDATDVRYHVLTSCDLQMVGDEFSRKPYAIAVQQGSPLKDQFN
NAILQLLNKRKLEKLKENWWTNNPNAMKCEKQDDQSDGISIQNIGGVFIVIFMGIGLACITLGVEYW
WYKIRKRSTIGDITQVEPAKSSRINTDFKGEGFTFRSRNLGLSNLKPKF

>OfurIR40a

MKFVPFFLFLNTAHCFFDIQDIISQTMTKLPKDFAVAIKDIAEGLPAKTITVVRGESTKIRSQDIFQLLCL
LSEHNIQVINLDITTKQNKDKYYSFVKQALDISEDRTSLILCEPFECEKILTELTDNNLIHRTILYIFYWP
YGTVSDKFLNTMKEAMRVAVLTNPRESVFRVYYNQATPDRLHHLSLVNWWSGRLYKSPVLPPAGKI
YQDFKGRMFDVPVLHAPPWHFVRYNNDSSVNVTGGRDDKLLSLISKKLNFRYQYYDPPDRSQGSSI
SGNGTFKGTLGLLWKRKADFFIGDVTMTWERLQAVEFSFLTLADSGAFLTHAPAKLSETLAIIRPFRW
EVWPLVCATVLVTGPALWVVIAAPSLWQRRQRDQLRLLNNCCWFTTTLFLRQSSSKEPSKTHKARLV
SVLVSLGATYVIGDMYSANLTSLLARPARERPIGTLQALEEAMRDRGYELVVERHSSSLTILENGTGV
YGRLARLMRRQRVQRVRSVEVGVRLVLTRRHVAILGGRETLYYDTERFGSHNFHLSEKLYTRYSAIA
LQIGCPYLETFNNVVMTLFEAGILAKMTTDEYKNLPEQSRRSEPVTESDKPNNDITGDSPAASQGGTT
PGESTKALEPVSLRMLRGAFCLLGIGHLLAAIALGVEIQIHRRSKKFIKIVEPNGGKNVPGKRALRKA
NKFIRQGIGRMVRAFCRSVDRALGPGNQ

>OfurIR8a

MMEIPLLLLFLINLGCVLSEISLRFVFITEVHDSDLAHQIGRALRNAEEQRSGVKISDYMVQLDRENE
DESYRRLCSGVSKGASLVIDLSWAPWDMAEQLCAESGLPLVRTLLGSQQLVAALDEYLESRNATDAA
ILLESESDVDKTLYELLGRSNVRLWVHAGLTRDSAKALKSMRPEPSFFVIVGESGFLMDTYRRAVKE
KLVRRDYRWNLVLTDYSGDSIDVTQLPLPTMILHVDQVECCRLLGLREECSCPSDLKRKQLIISALVL
YLSETYSKLERELPVLSTKVDCDNVLASEMNVTRDRLVRQFGEDVEINNDTLFYWDDDRSGLFLRSS
FVLSVYRPDSGLETVASWSANDEYKLLPGVTLDPLKLFFRIGTSPAVPWTLPKLDPETGEPEVNEDGQ
PVYEGYCIDLISKLAETMEFDYEIITPKSGSFGKKLPNGSWDGVVGDLMRGETDLAVAALTMTAERE
EVIDFVAPYFEQTGILIAIRKPIRKTSLFKFMTVLRTEVWLSIVAALVLTGLMIWLLDKYSPYSARNNPQ
AHPYPCREFTLKESFWFALTSFTPQGGGEAPKALSGRTLVAAYWLFVVLMLATFTANLAAFLTVERM
QTPVSSLEQLARQSRINYTVVEGSTIHQYFINMKFAEDTLYRVWKEITLNATSDQAQYRVWDYPIREQ
YGHILLAINASGPVPDAKTGFEQVNEHTDADFAFIHDSAEIKYEVTRNCNLTEVGEVFAEQPYAIAVQ
QGSRLQEHLSRALLDLQKERFLEQLASKYWNESARQACPDADESEGITLESLGGVFIATLFGLGLAMI
TLAWEVFYYKRKERNKIQGIDAKVEKAAFVDPKKKDKLGVRLRKGKSKVAKLDVVGKGKGVTIGD
TFKPAAEKMGVSYISVYPKGEYRP

>OfurIR41a

MLQDTVLFFPVEILLTSIVNLYLNSSYCLTIVSEKSLDLSISNSFTSMVPEDGDLLVNQLLQVSEMGCS
DYIVKMRDPARFMAAFERVNHLGNVRRSDRKVVFLPYEDNNITRTDLLQLLTLKETSFLANILLILPS
LESGLCSIYDLATHKYTGPDDQVDQPYYMDRWNSCSLKFEKDANLFPHDMTNLHGKTVKVACFTY
KPYALLDLDPLEEPLGRDGTEVRIVDEFCRWINCTIEVVRDDEHEWGELYDNQTGVGVLGNVVKDR
ADLGITALYSWYEEYLELDFSGSGIRTAITCVAPSPRLLASWEMPLLPFSWYMWMALGFTFVYASFAL
AIAKGCSTDKVFLATFGMMVTQSQADVGATWRVRSITGWMLLTGLVLDNAYGGGLASVFTVPKYE
KSIDTVQDIVDRGMEWGATHDAWVFSLTLSPEPLVKQLVSLFRVSSAEDMKIKSMQRSMAFSVERLP
AGYFAVGDYITKDAMLGLTLMQEDFYYEQCVVMMRKSSPYTQKVSKLIGRLHESGLMLAWETQVA
LKYLNYEVQLEVRLSRFHKDVDNVEPLKLRHVVGVFIIYIIGVIISTMLFILEIIHKHKKRVY

>OfurIR64a

MDINYFLNFISIAEISLVIDLLKLKEIQNVVNINCDGQKSIFHHKILNDNNIHASYWSLNSTDQNMMQ
MSYHKTGVILDASCSNWEQALNNFDNSMFRNEFIWLIITEDLLSTARSLTNCPIEIDSDVTVALKTNGI
FMLYEVFHTNYSSGVLSIRNVGYWDTTLHIATSSRRDLQGLKMRCPVVVTDKVVHQTFEEYLSKHQ
VFQVDSLHKLKFVALLNYIRDMYNMSYELQRTNSWGYMRNGSFDGVVGSLQRQHADFGGSPLFFR
ADRAELIDYIAETWQSRQCFILRHPKHPGGYYTIYTRPLTAKVWYCILAMLIFSGVILCLMLKTKVTQ
SHEKSTDSSFSLALLFAWSAICQQGMTVNRSSTSVKIVVIVTFVYAVTLYQYYNATVVSTLLREPPKNI
RTLEDLLQSNLKAGAENVLYTKDYFKRTTDPVALRMYHKKITPKHQYNFYSPEYGMSLVKQGGFAF
HVDSVVAYRIMRKTFTEREICEAHEVLLYPPQKMGMVVRKASPYKEHFTYGIRKIYEAGLMDRLQS
VWDEPKPSCVHTPDSSVFSVSIVEFSTALLALVAGNVAAILVLFAEIVLHRCEMKKRIAFTH

>OfurIR68a

MWIAVLVVLLISGSIFYGLARHYMNLQEYIKTHENRNTNEKQLDTAEKPVGLYLFGEIINSILYTYAML
LVVSLPKLPTGWSIRLLTGWYWLYCVLLVVSYRASMTAILANPAPRVTIDTLKELVESKIACGGWGM
ETKKFFQESSDDIQTIGQRFETINDPFVAANKVAKGVYAYYDNENFLKYIRVKRKNIDMNIQSDMVNA
TSNTTDVFAGEMERNLHIMSDCVVNTPISIGFHKNSPLKPLADIYLRRIVEVGLVEKWLNDAMHPIKS
LETNEDEVKALMNLKKLYGAFIALAIGYSISTIGLIGELIHWHLIVKRDPKFDKYAIDLYYLSKNKKQ

>OfurIR75

MNLRDLLLVFILVYFVCTFSKSSDEISIITDLIQSSDKLTSVVAHACWKPSKQIQLASRLGNKNRPMTV
RFVNKNWAGIVEPQHRERLLIVADLNCPSTKVFFKLANTTNKFSFPYRWIVIGKAVNESVAVTSNFQNI

PLLPDSDVIIAQKNDKNSYILTTIYKIQIKGKWIVEQFGIWTSANGLKKFEAVKHPISTRRKNFQRAPIK
MAMVILDNRTISNPYDLSDILTDTVSKSSFRQTDPIFGYLNASRTLIYSPTWGYYRNGSYGGMIADMT
IGDAELAGTVLIATQDRMEVVEYLSCPTPISIKFVFRQPPLSYQNNLFLLPFKSTVWYCIGAFVLVLAFI
LYINALWENKKLESSEQNFEDPTVLRPNVGDIAILVISAISQQGSSTELKGTLGRIVMFILFLAFLLLYSS
YSASIVALLQSSSNQIRTLSDLLNSKLELGVEDTPYNRYFFPIATEPVRRAIYQTKIAPKGTKPKFMSLE
DGVKKLQKEPFAFNMNKGIGYRLVERYFHEHEKCGLQEIPYLYATKTYITCRKNSPYKEIFKIGLFRIQ
EHGLSDRENRLIYARKPPCQARGGSFGSVNMVDFHPILLMYLYGILLAFFFFFVEILAHKKLHPRQSQ
RR

>OfurIR87a

MCTIIFLPLFFALHVSATINENSLLTTTGNSEQTAKTAECVLKLSAKYFVEKKALSGSIVIININSYASTT
QVLLLQTIHGGIKYSVMVKDSFYPHANASHFPEKAKNYMLILEEKSELTRNILQLNKLPTWNPLAKA
IVFYQLNKTEDAEQTAIEFINELRHYKLFKSIIFIYSPEEKEVISYTWTPYSDTNCGGKCDSVYILDTCK
DNVIHELATQKEMFPLDMKGCPLVTYAIVSEPYVLPPAMKLSNTSYNDAYVFQKGGEINLVKIITQFT
NMSLVMRTSDVPENWGNVYWNGTATGAYGVLRNDEVDMVIGNIEVTRTIRRWFHPTVSYTQDEMT
WCVPKAGQASTWNNLVIIFQWSTWVATFGSLFVMGLLFHYMYYRENNQKVTKWPTNSLLMTFSML
LGWGASFEPKSATFRILIFGWLCFSVNMGISYESFLRSFLMHPRFEKQISTETDLIQSRIPLGGREIYRS
YFETNNASSFYLYRKYNSTTFAEGIKRAAKDRNFAVVSSRRQAAYADQRLGKGKPLIYCFPESDNLYK
YGVVLLARKWFPMLERFNTIIRSVSENGLIDKWNQELLIHTANAEGASEIEPLSIQHLLGAFMFIGFMY
AASIAVFIAEICIGVFQKWKARKNWQSLYKHIRFRNAK

>OfurIR93a

MRICLFVSLYLLRVSGEEFPSLITANASIAVVLDRQFLGEQYQATLDELKDYIKELARVELKHGGVVV
HYFSWTTISLKKGFLAVFSVASCEDTWSLFSRTEEEELLLFALTEVDCPRLPTDSAITVTNVMPGEELP
QILLDMRTEMAFKWKSAVILHDDTLSRDMVSRVVQSLTVQIDEGASTSPVSVSVYKMKHEINEYLRR
KEITRVLSKLPVKYIGENFMAIVTTEVMTTMAEIARDLVMSHTLAQWLYVISDTDAQNGNLSSLINAL
YEGENVAFMYNITESNPECKNGLMCYCQEMMNAFISALDAAVQDEFDVAAQVSDEEWEAIRPNKIQ
RRGMLLKHMQQHISTKSSCGNCSTWRALAADTWGATYRSYGDSDQLFKEPDNATTKGVIEHVDLL
QVGYWRPIDALRFEDVLFPHVEHGFRGKALPIITYHNPPWTILQVNESGSVVSCSGLIFDIVNQLAKN
KNFTVKVILPSHVKNLLSNDTTADMMHSQDAALTLIAVAKGQAAIAAVAFTVLSDPPSGINYTLAVST
QPYSFMIARPRELSRALLFLLPFTTDTWLCLGLGVILMGPTLYIIHRLSPYYEAKEITRQGGLSTIHNCL
WYVYGALLQQGGMYLPRADSGRLVVGTWWLVVLVVVTTYSGNLVAFLTFPKQEIPVTTIGELLENQ
LTYTWSIQKGSYLEMELKNSDEPKYAALLKGAELSGAGASGNLSSWKKQLIRIREQRHVIFDWKLRL
SYLMRAEHMLTDTCDFALSAEEFMDEQLAMVLPAGSPYLPVINKEINRMQKAGLISKWLFAYLPKR
DRCWKTSSIAQEVNNHTVNLRDMQGSFFVLFLGFFSASVVLLLEWFCNRRRRRSEDVIIKPYVE

>OfurIR1

AAATSLYHKHQDITPILRWENVIDKVDLVHPPVTSIETRYFYRIPTYGAGKFENQFLRPLSYGAWISVV
IVITLCACVLLISAKLERRRSAGQYAIFSVLASMCQQFFEDNTSFTPRVSAARQLTIFVTGISCVLIYNY
YTSSVVSWLLNGPPPSINSLQELLESPLELIFEDIGYTRSWLQKKSYYYNIRNIEIEDELRKKKVFNKKP
NAPLLVPVEEGIKLVKAGGYAYHTDTNNANRLISQTFTQSELCELGSLQSMAKAELYPALQRNSPYKE
FFVWGSIRLYERGVVKFVQRRISSPAVECEGSSPRALALGGAAPAFLLLAAGYLLATVIMLVERAIWR
RKYKSKVVLKLKK

>OfurIR2

RVLVPAKLWYSTQDVLRSESADLNAGVLRIMETRMEYLDYIMPIWLFSVGFTYLAERESSSNMYVEP
FTAACWWTCLGIGVTLALAQRVAARGKQEKEGAFMAVLATWLQQDAAAVPDGVAGRWTFTVMSIC

AMLVHAYYTSAIVSALMSTGRGGPNTLRELADSRYAIASEDHDFIRNGMFNVETDWEELEYLKKKK
MTSKLFQDMEYGVQLIQQGYTAYHAEYHQLYHFLEKFSDDEICKMQHVDTIPEIMTWVSASARGQ
WTEIFRSTGAWLYETGLARQLLSSIQTHPPPCRAAMLAERVTFWDVAPLLGLTTIGAIASIGLLGLEIV
LHRWTEKNRREKKWGSSEASLVALE

>SlitIR21a

MAVPWFYVVFLAYHVVFGAEVIIEYYPSQSVLDMNNKVVRKREVNNTDDPNLNINGSDSYWRHFN
NDTDDGDIHKRALDPVFYGHPKTREELWNERFLNETTSFDQTPSLVKLLHNITLTYLKDCTPVILYDN
QVMSKESYLVQNLLKGFPTTFIHGYINDDGELVEPELIHATIECQNYILFLSDIKISAKILGKQPENKIIII
ARSSQWAVQEFLASVTSRNFVNLLVVGQSFKEGDDAKLESPYILYTHKLYTDGLGASQPLVLNSWTH
GKFSREVNLFPLKMTEGYAGHRFVVAAANQPPFVFRRIKSDLDGGNPRVVWDGIELRLIKLLAERNN
FSIEIIEPREPNLGPGDAVAKEIVTGRADIAIAGMYLTNDRIREMDMSLAHSHDCAVFVTLMSTALPRY
RAILGPFHWHVWVALTFTYLFGMFPLAFSDKHTLRHLIHNSGEIENMFWYVFGTFTNCFTFLGKNSW
SKTNKITTRLLIGWYWIFTIITTSCYTGSIIAFVTLPVFPETVDTIKQLLAGFYRVGTLDRGGWEKWFL
NSSDPQTNKLLRKLELVPSVEAGIRNTTKAFFWPHAFLGSKAELEYIVQANFTATKSKRAVLHISNKC
FVPFGITIGFPNNSVYSAKMNLDISKMIQSGLIDKITNEVRFEMQRSPTGSLLAAGSGTINIPSAEEKGL
TLEDTQGMFLLLAAGFTIAATALVSEWMGGFTRRCRFQKKSETPTSANSRDNLIITPKTDVDSEIRIIE
DTERRLHFEERPSSSVSVDTLEGQVIHVTESSIDVHNTFNVDRFDSRRSSSLDLDREVREIFEKDQKRR
RIFSRDMESLDENGSTVSRAAFGDSVKNDI

>SlitIR25a

MNGEPEITSAFYFDLSLRTFLTIKSLLDSGKWPNDMKYITCDDYDGKNTPNRTLDLKTAFQEIKETPT
YAPFYIPQDDPMNGRSYMEFSTDLLAITVKDGASISSHSLGSWKAGLSSNLTLTDPNNMSNYSAQLV
YRIVTVEQKPFIIRDDQAPKGFKGYCIDLIEEIRAIVKFDYEISLAPDGNFGTMDENGNWDGIIKELVD
KKADIGLSSLSVMAERENVVDFTVPYYDLVGITIMMKLPRTPTSLFKFLTVLENDVWLSILAAYFFTS
FLMWVFDKWSPYSYQNNREKYKEDEEKREFTLKECLWFCMTSLTPQGGGEAPKNLSGRLLAATWW
LFGFIIIASYTANLAAFLTVSRLDTPIESLDDLSKQYNIPSATVSMDLRLMTCFQRGGYLGGSFLKFGGS
WAKRPFKGGGTAQNLRYGIIPVRDKYIKFWRAMEEAVLPLPFLGKLYRGVRDSKSFSEGFAWLGDAT
DVKYHVMTSCDLQSVGDEFSRKPYAIAVQQVSPLKDHFNNAILQLLNKRKLEKLKEIWWNNNPESM
KCEKQDDQSDGISIQNIGGVFIVIFMGIGLACVTLGVEYWWYKWRKRPAVGDVTQVEPAKLTRNNV
DKQGEGFNFRGRNLGLNFKPKF

>SlitIR40a

MRRQRVQRVRNVEVGVRLVLSHKRVAVLGGRETLYYDTERFGSHNFHLSEKLYTRYSAIALQIGCPY
LETFNNVVMTLFEAGIVAKMTTDEYKNLPEHARRSDPVTESDKQGGEVMGESAATSSQTPQGESTK
GLQPVSLRMLRGAFCLLGIGHLLAAISLAVEIQLHRRSKRRRKPEHNEHRKAQKLLVLGKSVMLFKR
GCKKVCTSVFTSIDKALGSDNKDYFDKMAPDLGNLFLIVVCLLTSRVFLVHTGVLCRIFSSLQPVLYLI
EQNSYFAVSIKIFTRVSENARLPVCTRGAREGNSNFVFKCFFYLSRLKVVATVIVK

>SlitIR41a

MLLPTISLPLEILLNTIITQYLDSSYCVTVFSDKPLSPIISTSFIYLIPDEENLVEQIYNVSERGCSDYIVRM
RDPQNFMTAFERVVHIGNVRRSDRKIIILPYNEEYNDNNDENLPSLIFSMKGSEYLANMLMVVNHNS
SNSDCKEFDLITHQYVGPDDVSNLPKYLDRWDSCSQQFENNANLFPHDMTNLFGKTLRVACFTYKP
YALLDIDTAIEPLGRDGVEIRIVDEFCRWVNCTVEVVREDVDQWGEIYKNESGGIGVIGSVVKDRAD
LGITALYSWYEEYRVMDFSVAGVRTAITCIAPAPRLLSSWEMPLMPFTWYMWLAVVFTYFICLNWDF
NSTGIWFIIVSILNAFGMMIGQSQYEGKPSWKIRSVTGWLLIAGLILSSAYGAGLASTFTVPRYEPSIDT
VQDIVDRKMEWGATHDAWIFSLTLSTEPLVKELVSQFRIYSFDELKRKSFTRSMAYSIEKLPAGNFAIG

EYVTQEAILDMMVMLEDFYYEQCVVMMRKSSPYTEKVSQLIGRLHQSGLLLAWETQVALKHLNYK
VQVEVRLSRSKNDVGTTKALNLGNVMGIFIVYAIGLMLSIATFLGELYVHHHKQKKERIHVD

>SlitIR68a

MLRILIIFVITSTYNSRLQVESFPIIKDLHERRDLEFVLIDLLNVLTRDYEVTCIAIICDEVYLNVFGGPLF
KRTASVPYVMTVVEDYEDLLSPNFVTLESLRAARKEGCNVYVILLANGLQASRLLRFGDRHRILDTR
AKYIMLHDFRLFRSELHYIWRRIVNIIFVKYHKKILGVSKSRPWFELSTVPFPNPIKGVFVPRRVDIWK
NENFYYKRPLFADKTSNLNGEVLNVVYLDHVPSVVVVKNNGSNKIGGVEVEILHTLAEKMNFKPKP
YQAINAELHKWGQKQPNGSFSGLLGEMVNGRADVALGNLQYTPYHLELTDLSIPYTSQCWTFLTPE
ALTDNSWKTLILPFKLYMWIAVLLVLLITGTIFYGLAKNHMNLQEYKKLRPIQTKDDEGIDAKPGLYL
FGEIINSILYTYGMLLVVSLPRLPTGWSIRLLTGWYWLHCILLVVSYRASMTAILANPAPRVTIDTLREL
VDSKVTCGGWGTQSKKFFQESLDENTQKIGDKFETIDDPMKAASKVAQGVYAYYANSDFLKYISVT
RKDALKGSKGNSTNTTDIAPKIDSQRNLHIMSDCVVNIPISIGFHKNSPLKPLADVYMWRVVEVGLVE
KWLNDVMHPIHSLETNEDELKALMNLKKLYGAFIALAIGYTLSALCLAGELTHWHFIVKRDPNFDK
YALHLYYRNKNKKDY

>SlitIR75d

MELISFILSYFITKDLSMMTAFICWPSEQALELQRSARVAGVRLTVVSELRHSAPMTTSGYFREAMLL
DLNCPDTHFVLEKASRSRVLNKRHSWLLLHNSSAEPALVEETLYAYEILPDADVVWSSPNSLVDVYK
TKPNQPLLQVQLGLSRNSSHQELLSLWGALPTAVTRRRDLRNVSLKGISVVTEPDNFKGWADLRNRQ
IDTFPKFTYPLMMLLAQDLHFRFDLRQVDFYGVSHNGSFDGLVGHLQRREAEVGLASLFMRHDRM
QVADFFSETCVLACAFIFRQPSRSAVSNVFLAPFSAGVWGASACVAASAALLLVALRRLRQHTRASTD
LQLFTLLEAVTFALGSMCQQGFHRTPPVTSVRLVMFSTLLTSLFVFTAYSAKIVAILQTPSTALQTIDDL
VRSPMTIGVQDTTYKTVYFLESPEKSTQQLYRHKILPQGERAYHSVVDGIARVRTGFFAFQVEKSSGY
DIIKQTFTEREKCSLSEIEAFKPPLVAVPMKKHSGYRELFASRLRWQREVGLMDRARHVWLVS

>SlitIR75p

MTGISIVLFFLVTQCSVLIQSKDMENINFIKLFILNDQKPTHLIYGGLCWKKELINKLVVEMSNIGVRTS
ASFKPRSKYQDHAIMYLTDLDCAQSRTVLSYASSKELFQFTYRWLILVTSPQLQQSKISLLENGPVLV
DSDVVLAERVGNMFKMTELHRPGPNGSMISTPRGYYNGSVVDVRAHRELYRRRRNMRGHAITMSN
VIQDSNTTRLHLPREDRLKLQYDSITKACWSAAKIGFEMINATPRYIFSYRYGYKVNGQWSGMIADL
YANKADMGTNCVIFRDRFDVVTYTDLVAPMRMLFIFRQPPLAYVANVFYLPFSTRVWVTIAVCTAIAT
VTLFLASKVEIVITKTTTQQQLDGGICDVLLLTMSAVTQQGCYIEPRRAPGRMMVFVLFTALMALYA
AYSANIVVLLQAPSDSIRSLPQLANAKITLAANDVDYNHFVFNQSREPLYISIRDRVFPENGKAKLYSL
ADGVERIRQGLFALHSVAEPVYRQIEATFLESEKCDIATVDYLVTFDSFTPVRKGSPYLELIRVVHKQI
RESGIQSAIRRRYLVSKPHCTTKMSSFSSVGLMDMRPVLILMLYGVAVSVIIVFGEIIVHKLINRYYKQ
KSKVQMVKTIHY

>SlitIR75q1

MKYLTFVLNIICLDYCVTFNTNTELQIIVDVAKSYDKPTSVIAKMCWETSKRSKYAPLEAKLAKMLA
NLDRPMNIRYLRQNETIDNDNYPNNHLLFILNRTCDDANAFLRWASANHKFRKSHRWLILGKSLIIKD
ETFNVSPEFDDIRISVDSEVIIIGKINNSEEVSLHTFYKLKPHTKWIIEDYGTWSFDTGFTKSTTRIESNV
IRRKDFMGETLITSVAISDNRTKTDLLGLGNIFIDTPAKSSFRTIVPLFDFLNATKVVKSLILGVLINGSG
MEGLVILSGRSRLVWNCNVHNERTYDNFRIFDPSYTHHTEVCLQTTALVVPEQFIPSAIFHWCLAVHW
CIHCYINCHIVRQHDMGFKEIQRLNKQKIDQTCLPPTWSDITIFVLSAISQQGSSNELKGTLGRLVMFL
VFLAFVFLYTSYSANIVVLLQSTSNQIRTLSDLLHSRLELGLEHAPFNKFYFSSAYTADDPIKKALVDT
KIAPKGVLTNVMNIEQGVRIMQKKPFAFNMNTGXRVQNCFSNLKNHGKVSGLQEIEYIPNSNPWLCS

RRLSPYGELFKVGYIRIQEHGLSDRENRLIYAKKPACTVMGGSFGSVNMVDLHPVCLVLLYGMILAF
LLLGVEILVHRKQMKMRNQARVECNVKLCSKVASCEIRFWFCIAYKMCSNDAFIAKILLIFFTVNRKL
LMASPPLTSVTGDPMAFNVLIDDFH

>SlitIR76b

MAGIELIISSICNATFCEVPYNETYQAPDSLAEKDTNFMSLMKEVNGKNIKVTTYNNTPLSSTELENG
TVVGKGVAFTILNILRKKFNFTYEVVLPTKNFELGAKISDDSIIGLLNSSKVDMAVAFIPTLLPYREWV
SFSIDLDEGVWVMMLKRPKESAAGSGLLAPFNDLVWYLVLAAVLTFGPCITFFTRVRSKLITDDEGVL
PLKPSFWFVYSAFLKQGTNLSPEAHTTRVLFVTWWLFMILLSAFYTANLTAFLTLSKFTLAIETPKDLY
QKNNRWVASAGSSVEHVVKTEGEDLYFLNAMISSGKARFLSVAGDKDFLDFVKKGAVLVKEQTVV
DHLMYNDYISKKDVEESEKCTYVVAPSAFMKKQRAFAYPVGSKLKGLFDPVLTQIFQAGILDFLKRS
DLPSTKICPLDLQSKDRKLRNSDLIMRYMVMVAGSATAVAVFGAEVFIKRYVSGKLNKNKKSKRKKS
KTGKSLKSHDDSRPPPYDSLFGKNPKFNVETTRMKMINGREYYVFETSNGDKKLIPARAPSSFLYRSD
K

>SlitIR1

MLAQKLTKENVRVSVRRLNGDNVDVVRVAHQTTVPVGVLVDGHCDQTQTLMNQASFNKLFDAVH
SWLILTDFEDDNCTEYVMQTFQWLNLSVNADVAVVANRGDSFAIIDVYNFGKIQGNHLETALLGTW
QPDQGLEIILKGYKYYNRWDFHNLTLRAISVIVDQPKVFYPEMLSEMTYTSGVAAMTKITTQMLNTI
KERHNFRFNYSIASRWIGSPERNSTMAVTNTLFWEEQDLSSTCARIFPKWLNWVDIYHPPTTNLQTKF
YYSLIPETGCRGQYEGTGFLTLGCSHGGLGGCVLLSPGIPRTRGSWQPRLRMEKQTQARDCMPSFRV
FAAGLSTRLGRRRSGCWSQTLSSQGRRTNPASDRTDEHAAVQLTTPAVWCPGCWTLRAPFPSANLEG
LINSDFELVLEDIGYTRGWLDNPGFFYYSGFKNVKEDELRDKKVTKAKRTVSVLQNVNKGVELLRT
GKYAFHTEPYTASQVISKTYEDKELCNLGALQMMLPAHVYIMAQKKSPYKEFFDWSLLRLLERGHV
KAIRARFAGTMSACSGAQPRALALGQAAPAFLMLASFAVLSCFILVLEVLWKRVQLKNRGQ

>SlitIR87a

MTLITRTSEILENSGVVYRNGTATGAFEVLRNETADLVIGNVEVTRVLRKWFHPTVNYLQDEMTFCL
PKAQQAPTWDNLVIIFQWSTWVATFLSLVIMGLVFHFFYYREHSNATKWPTNSLLMTFSMLLGWGAT
FEPKSPTFRILIFAWLCFSINMGISYESFLRSFLMHPRFEKQISSEADLIQSGIPLGGREIYRSYFETNNAS
SFYLYRKYNSTTFSEGVRRAALQRNFAVVSSRRQAVYQDQKLGKGAPLIYCFPESNNMYKYGVAILT
RRWFPMLERFNNIIRSVTENGLIDKWMNELLIHTVSSEEASTIVPLSIQNLLGAFMFIGFMYACSIVIFL
GEVIMGVIGKRRRVKKFKCKCSW

>HarmIR64a

INRESTSTKLLIFITFMYAVTLYQYYNATIVSSLLLEAPRNIRTLKDLLDSDLKAGAHDIVYNYDYFKRT
TDPVAIELYHKKVVTATQHNYFPAEKCMDLVRRGGYAIHIDTSVAFPLIKATFNEREICDTTLVQMYPL
QRMGVVMRKNTQYREHVANAIRRFSEAGLPQRLRSDVDEPMPECAHTPDSSVFCVGIREFSTPLLVL
ALGMLLSVMLLLCEIILHRVVQRAGLRDFVH

>HarmIR68a

GMLLVVSLPRLPMGWSIRLLTGWYWLYCILLVVSYRASMTAILANPAPRVTIDTLKELVDSKVTCGG
WGTQSKKFFEQSLDEYSQRIGDKFETIDDPMEAANKVAQGVYAYYDNSDFLKYISVVRKNSFMDPK
QNNTANNTEVTGRKDTQRNLHIMTDCVVNIPISIGFHKNSPLKPLADIYMWRVVEVGLVEKWLNDA
MYQIRTLETSEDEVKALMNLKKLYGAFIALAIGYSLSAICLIGELIHWHIIVKRDPNFDKYALHLYYLH
KNKKH

>HarmIR60a

KMKARYNFIDGYRGERENVGEWNGGLKKLASKSGHLLLGGIFPDFDVHEDFETSVTYLADAYTWV
VPRAHKSAAWVALVIIFKSLVWYSVIAGFFLCGITWKIIAELSEDSDYNRSFRHCFLNTWITVLGFVSY
LHPVKESLRVFFVFLNIYCMLFSTAYQTKLFEVLTNPSYEYQIQTVEELVESGLKFGGFEELHDLFYNS
TDPFDYRIGDQWTDITNITEAMIDVAVHRNFSLLCSRLELAHISGITPELSDSVGNYKYYTFTDNVFSV
PIETIALRGFPFMMEFSTTITIFKQSGLNEGLRQHFAHFNERRRARQLRALLKEKSDVNPLSSEHLQGG
FLALALGYVSGTLALIVEVILNCNYVQNKFENFKRRVNPLS

>HarmIR15

MAYWYYLLNAVMTTMIGCSPMKNRSMIIIRSACLSGSILFLAVYQGHTSRVYTTLKHFERISTLDDLY
AFGAILYTTPGMRQFTRQLQRPGNKLEEDFFNRSRLILNERIGAGITLEIPRATTLDRKSDAEMKILEH
FSDREGRPLIDIVDECFMNYFLSYITRSGFPFFEEIQIFTQRLLEAGLPTKYYKWTQQMLNIPTSLPETR
SEPRPFSKIKLKDQRVAFFVLFVGSALSIIVFAVEIFKGPPVEF

>HarmIR75p.1

EADLGTLTIFTQERMMAVDYIAMVGSTAVRFVFREPPLSYISNIFALPFSGAVWLAIFICVLGCSIFLYIA
SKWEASMGMHPLQLDGSWADVLILMIGAVLQQGCTLEPRYAAGRCVTLILFLALTILYAAYSANIVV
LLRAPSSSVRSLPDLLNSPLKLGASDFEYNRYFFKKLNDPIRKSIYEKKIAPKGKKANYYSMKEGVER
IRKGLFAFHMELNPGYRLIQETYQEDEKCDLVEIDYINEIDPWVPGQKRSPFKDLFKINFLKIRESGVQ
ANIHQRLTVPRPRCSGHVSTFSSVGITDMYPAMLMTLYGMLLAPAVLLMEIMYHRLMIARQQKRGTS
DYDHIPFRH

>HarmIR93a

NPDCKNGIMCFCQELMDAFISALDAATQDEFDVAAQVSDEEWEAIRPSKIQRRNMLLKHMQQHLAA
KSRCGNCSTWRALAADTWGATYRGLSDASDLSNVNTNGSSGVIDKIDLLKVGFWRPIDAVRFDDVL
FPHIHHGFRGKELPIITFHNPPWTILERNESGAIVKYSGLIFDIVNQLAINKNFTLKIILASVLKKELAND
TLADTMHGMDAKLTIAAISKGQGALAAASFTVLADPMPGINYTMPVSIQPYAFMIARPRELSRALLF
LLPFTTDTWLCLGLAVILMGPTLYIIHRMSPYYEAMEITRQGGLATIHNCLWYIYGALLQQGGMYLPR
ADSGRLVVGTWWLVVLVVVTTYSGNLVAFLTFPKQEVPVTTVAELLENRALYTWSITKGSYMEMEL
KNSDEPKYIALLKGAEMVTTSVGMGGTMTSGSALLQRVRFHRHVIIDWKLRLSYLMRADRLESDNC
DFALSAEEFFDEKVAMIVPAGSPYLPVINKELDRMHKAGLITRWLEAYLPKKDRCWKASSMMQEVN
NHTVNLSDMQGSFFVLFMGFFSASTVLVLEFLYHRRKRRSELTVIKPYVE

>HarmIR7d.1

SVIVKLGRFLPARRAAVPQMIIFGEDASEISSTIRWTVRAKYDSNGKFIIICAHLEQECDELKIFQTLQSL
YMFNAVVLKTSNKTKESLAYSYDFLSEGKCKNSIPYKVNLTTDCFNDNCFKNLYPERLSNFRKCPLI
MSTIEQPPFMYLHNLTSKPTGIDGDIMRLVADMLNATLHLKPPYDGADSGHFANNNWTGSLGDIYNN
HSHASVCSAPITSGKYGNFQISFTYYSMDIVWATRLPAQQAPWQKLLHPLNIYIRIILLLMFICIIFMN

>CpunIR1

MKKIILLCVLLCANTLADLTPILKGLNERKDLEKILVDLLNEMTGSDDEITCMTFVCDAVYLNVFDGD
LFKRTMAVPIVMIVVEDYEDLLSPNFDTLESLREARKDGCNVYVILLANGLQTSRLLRFGDRYRILDT
RAKYIMLHDYRLFHSDLHYLWKRIINVLFLRYHSISVGAPKSKAWFDLSTVPFPNPIKSVFVSRRVDI
WNNGRFHYNRTLFADKTSNLNNEVLNVVYLDHVPSVVVTKTNETNKVGGVEIEILNTLAKQMNFQ
PKLYQPINADLYKWGQVQANGSFSGLLGEMVNGQADVALGNLQYNPYHLELTDLSIPYTSQCFTFLT
PEALTDNSWKTLILPFKVREAGGPVSVWRDNKQHSLHLRNAARRVFTQATHRMVPETSNWVVLVIL
HSGRRILQSQHDRYSRKPCPQSHN

>CpunIR2

MEIPLFLLIFFINLGCVVSELSLRFVFIIENHEPELGQLVGRALKVAEEQQDVRVDDSIVLLDRENEEES
YGRFCSAISKGVSLIVDLSWSPWELAESVASGGGVPLVRTALSMQRLLSAVASHCASRNATDAALITE
SEADVDRALYELLGRSNIRLWVHAGLTRDSARALKNMRPDPSFYIIVGESGFVMDTYRRAVKEKLVR
RDFRWYLLLTDYSGDSFDTSQLVLPTMMLHVDANECCKLLGSKDDCSCPSDLKRKQYILSGLMTYL
AETYSKLENDLSVVTAKMDCDNIQASEMNVTRERVVRQFADDEQISNDTLFYWDGERSALYLRSTF
VLSTFKPDSGLETVASWSANEEYKLLPGVTLEPLRPFFRVGTSPAVPWTLPKLDPDTGEQVYNEDGQP
EYEGYCIDLIARIAETMEFDYEIITPKSGTFGKKLPNGSWDGVVGDLMRGETDLAVAALTMTAEREE
VIDFVAPYFEQTGILIAIRKPIRKTSLFKFMTVLRTEVWLSIVAALVLTGLMIWLLDKYSPYSARNNPTA
YPYPCREFTLKESFWFALTSFTPQGGGEAPKALSGRTLVAAYWLFVVLMLATFTANLAAFLTVERMQ
TPVSSLEQLARQSRINYTVVEGSTIHQYFINMKFAEDTLYRVWKEITLNATSDQAQYRVWDYPIREQY
GHILLAINASGPVPDAKTGFEQVNEHTDADFAFIHDSAEIKYEVTRNCNLTEVGEVFAEQPYALAVQQ
GSRLQEQLSRALLDLQKERFLEQLTSKYWNESARQACPDADESEGITLESLGGVFIATLFGLGLAMIT
LAWEVFYYKRKEKNKVQTIDAKMEKAAFTEPKNAEKTGVRFRKKEKKSKLGKISKLGKVEEGKLG
KRVTIGDSFKPASEGAGVSYISVFPKGEYRP

>CpunIR3

MINILRIIFTIQIDWVNKFILFFKYKNFISYLFTMMLDSVLSLMPIEILLQTIFNEYLSNSYCLTVVSEKPL
DLHVNISYAYISVENGELSPDQMLKLSENGCSDYIVQVKNPQKFMGAFETVNLLGNVRRGDRKIVFL
PYREDNATTTLLLEILTLKETSFIANILLILPSPEQSTCSYYDLVTHKYVGQDNEINQPYYIDRWNACTL
NFEKNVSLFPHDMTNLYGKTLKVACFTYMPYVLLDLSEAQEPFGRTGTEIKIVDEFCRWVNCTVELV
REDEHMWGEIYDNLTGVGVIGNLVEDRADIGITALYSWYEEYVVLDFSAPGVRTAVTCIAPSPRLLAS
WEEPLLPFSWYMWLALIFTFVYASLALTIAQGFTTDNAFLTTFGIMIAQSQHDVGASWRVRSVTGW
MLLTGLVIGNAYGGGLASVFTVPKYEKSIDTVQDIVDRKMEWGATHDAWVFSLTSSNEPLIKKLVNQ
FKVYPADVLKKKSLDRSMAFSIERLPSGYYAIGDYITKEAMLDLTVMLEDFYFEQCVAMLRKSSPYT
KKISQLIGRLHESGLLLVWETQMALKYLNYEVQLEVRLSRSQKDINTEALSLRHVVGVFILYLIGMIF
SVIIFTLEVMNVNKKRNTSSF

>CpunIR4

MLNKMQDMKTYEVLFILLMSCFSLIIANPINEFRMIADVIKDSNKSTSVVAHLCWNPSKQIQMASYL
HNSELTQLVLLVNESWADIKEPQHRERLLLIADIDCPSTTAFFKMANETKKFSLPYRWLIIGKAVNKST
DVTADFDGLHLLPDSDVIIAQKNDSNSFYMNMIYKIKIKSKWIIEDFGTWTTNTGLIKSDLAQYSTST
RRKNFHGESFTTAMVIFDNKTISNLFDLSDILTDVVTKSSFRQIVPLYGYMNASQQHIYSKTWGYYRN
GTFDGMIAELTVGDADLGGTVLIVTWDRMQVVDYLSKPGSITVKFVFREPPLSYQNNLYLLPFKVTV
WYCMGAFVLVMGFILYITALWENKKMGENQEISNDPTVLKPNVSDIAILIISAVSQQGTTLELKGTLG
RIVMIIQFIAFLLLYASYSASIVALLQSSSNQIRTFSDLLNSKLELGIEDTPYNRYFFPIAVEQVKKEIYKS
KTPPRWTEPKFMSLEDGVKKLQKKPFAFNMLQGIGYKLVERYFHEHEKCGLQEIELQYGTKTYIASR
KNSPYKEIFKIGLFRIQEHGISDREFRLLYARKPTCQVRGGNFDSVNMVDFHPVLLMYLYGILLAIALL
VIEILVFKKQQLMCSAASRRQRSGSC

>CpunIR5

MKATVLLLFCLKYLNVKSHANTNSVMHMVGDIIRAMEKPSSVVATLCWLTDEKVQFYYAVTASDRF
SRVNTAQFVDMRHVSEDHGQEQHIVFVADLSCPNISAYFDEKRAQNYFRGPFRWILIGNVVEEDIVPN
SIAHIDALPDSQVIVARQIDEESYDLYTIYKINANDDWRTKLYGKWNQQTRFTITNPHMESIALERLDL
LGSEISVCYVLTDKDSINHLTDEVNDHIDTITKVNFPTTNHLLDIVNASRKYIFADTWGYRVNGTWNG
MTGYLIREEVEIGGSPMFFTSERISVVDYIASPTPTRSKFVFQQPKLSYENNLFLLSFRTSVWYSSTGLI
FLLLLALFVVAAWEWKKHTNDNQVSFQRENDAGTLRPNFVDVIVLIFGAICQQGSPVELKGSLGRVV

MLILFLALMFLYTSYSANIVALLQSSSTKIRNLDDLLHSRLKFGVHDTVFNRYYFSTATEPVRKAIYEK
KVAPPGTTPRFISMEEGVKKMRKGLFAFHMETGVGYKFVGKYFDEGEKCGLQEIQYLQVIDPWLAV
RKHTPYKEMFKIGMKRIQEHGLQSRENLLLYEKRPKCSGRESNFVSVSMVDCYPALLILSYGILVALF
FLAFELLIHKRQTIVHRLSHCRRNSIDSRFI

>CpunIR6

MSHSMIHLRCIVAYTLLLYYTVWCEDVEYYPSQYTIDNHNIAKRSSNYLQSTDNKWNIKEKVTQMK
LRYFNDNDIKAKDNKTKRAVDPVFHGHPKTREQLWHEHFLNKSTAFDQNPSLIKLIHKITLKYLNDCI
PVILYDSQVKSKESYLFQNLLKDFPVSYVHGYIDDSNNLKEPELLVPVKQCLHYIIFSTEVKSSAKVLG
KQSESKVVVVARSSQWAVQEFLASPESRMFINLLVIGQSFKDDDDETMEAPYILYTHKLYTDGLGAS
KPVVLTSWTHGKYSREVNLFPAKMTEGYAGHRFIVSASNQPPFVFRRIKSDLDGGNPRVVWDGVEL
RLLSMLAERNNFSIEIKEPQEPSLGPGDAVSKEVAMGRADIGVAGMYFTSERTYGLDMSFSHSQDCAV
FITLMSTALPRYRAILGPFHWHVWVALTFTYLIGILPLAFSDKHTLRHLLHNSGEIENMFWYVFGTFT
NCFTFLGKNSWSKTTKITTRLLIGWYWIFTIIITSCYTGSIIAFVTLPVFPETVDTIQQLLAGFYRVGTLD
RGGWERWFFNSSDPNTNKLFKKLELVPNVEAGIRNTTKAFFWPYAFLGSQAELEYIVQANFSMAKS
KRAMLHISDECFVPFGVSMAFPSNSLYSSKLSGDLRRMFQSGLIYKIVDEVRWEMQRSSSGKLLSAG
AGSLKIVSAEEKGLTLEDTQGMFLLLAAGFLLAASALISEWMGGIGRRCRQLRNKLPSSANSKEQLVI
SSPDLESEVNDGTESRLQFGTRSTSAGSRDTLDGQVINVTEENIIVHELMVEGLDSRRSSSVDLDREV
QEIFERDLRRRKIVTGDSIEVSEEKREPTASKGAFGDPLS

>CpunIR7

MATGLELILSSICNATFCEPIFDNPLLGRQDSPKDVKYNDMVNEINGKHLKIATYDNRPMSWVEKGE
NGTIIGKGVAFVIVNILQKKYNFTYEVVVPEKNFEMGGDNPQDSLVGLANSSLVDMVAAFLPKVNKY
REKVSFSYDLDEGVWMMMLKRPKESAAGSGLLAPFDNAVWYLILIAVLSFGPCITLLTRLRNKMVP
DGEKFIPLSPSFWFVYGAFIKQGTNLAPEANTTRVLFTTWWIFIILLSAFYTANLTAFLTLSKFTLDIETP
QDLYKKNYRWVSPEGSAVQYVVNSPNEDLYYLSRMIGTGRAEFRTVPNSQDYLPLVDGGAVLVREQI
GIDELMYGDYLKKAREGVAEADRCTYVVAPNNFMTKLRGFAYPRDSKLQYFFDSILTYILQAGIIDFL
EKKDLPSTKICPLDLQSKDRQLRNSDLMMTYMIMVTGLAAAVAVFIGELFIKRYICKTKDEVTKPKR
KKTKFEKRLRIHTYDDSQPPPYDAIFGRNPKIKVTERAQRKIINGREYLVIDVSNGETRLIPVRTPSALL
YQLDK
